# Carba Analogues of Flupirtine and Retigabine with Improved Oxidation Resistance and Reduced Risk of Quinoid Metabolite Formation

**DOI:** 10.1002/cmdc.202200262

**Published:** 2022-07-07

**Authors:** Konrad W. Wurm, Frieda‐Marie Bartz, Lukas Schulig, Anja Bodtke, Patrick J. Bednarski, Andreas Link

**Affiliations:** ^1^ Institute of Pharmacy University of Greifswald Friedrich-Ludwig-Jahn-Str. 17 17489 Greifswald Germany

**Keywords:** drug design, flupirtine, ion channels, K_V_7, retigabine

## Abstract

The K_V_7 potassium channel openers flupirtine and retigabine have been valuable options in the therapy of pain and epilepsy. However, as a result of adverse reactions, both drugs are currently no longer in therapeutic use. The flupirtine‐induced liver injury and the retigabine linked tissue discolouration do not appear related at first glance; nevertheless, both events can be attributed to the triaminoaryl scaffold, which is affected by oxidation leading to elusive reactive quinone diimine or azaquinone diimine metabolites. Since the mechanism of action, i. e. K_V_7 channel opening, seems not to be involved in toxicity, this study aimed to further develop safer replacements for flupirtine and retigabine. In a ligand‐based design strategy, replacing amino substituents of the triaminoaryl core with alkyl substituents led to carba analogues with improved oxidation resistance and negligible risk of quinoid metabolite formation. In addition to these improved safety features, some of the novel analogues exhibited significantly improved K_V_7.2/3 channel opening activity, indicated by an up to 13‐fold increase in potency and an efficacy of up to 176 % compared to flupirtine, thus being attractive candidates for further development.

## Graphical abstract

**With the aim of providing** safer replacements for the K_V_7 potassium channel openers flupirtine and retigabine, the oxidation‐sensitive triaminoaryl scaffold was modified in a ligand‐based approach in order to obtain oxidation‐resistant carba derivatives. Some of these novel analogues have a negligible risk for the formation of quinoid metabolites and possess potent K_V_7.2/3 opening activity.

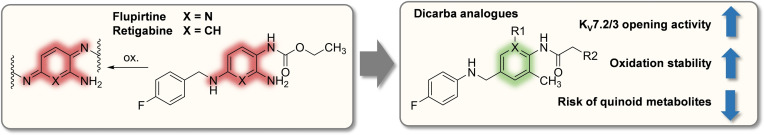

## Introduction

K_V_7 channels (KCNQ channels) are homo‐ or heterotetrameric, non‐inactivating, voltage‐dependent potassium channels with slow gating kinetics.^[1]^ Their activation causes an outward‐directed potassium flow, which in turn leads to a more negative cell membrane potential and thus increases the threshold for new action potentials.^[2]^ The resulting current is commonly referred to as the M‐current, which is fundamentally involved in controlling neuronal excitability in both the central and peripheral nervous systems.^[3]^ The possibility of slowing excessive neuronal excitation by activating K_V_7.2/3 sub‐type channels makes them an attractive target for drug development in some areas with unmet medical needs, including pharmacoresistant epilepsy,^[4]^ different types of pain,^[5]^ and depression.^[6]^ While strong evidence exists for the clinical value as analgesic and antiepileptic, only preclinical data suggest an additional antidepressant potential to date.

**Figure 1:**
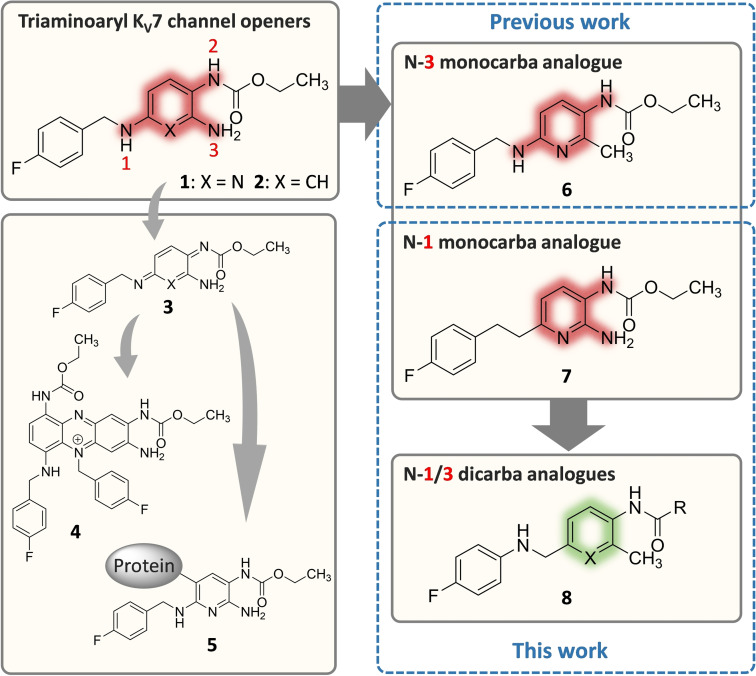
Structures of flupirtine (**1**) and retigabine (**2**), the proposed toxification pathway to phenazinium salt **4** in the case of retigabine, and hapten‐protein adducts **5** in the case of flupirtine via azaquinone diimines or quinone diimines **3**, and selected structural modifications carried out in previous work (**6**) and this work (**7**, **8**).

Flupirtine (**1**, Figure 1) was the first approved drug to act as an opener of K_V_7.2/3 channels, although it was only identified as such several years after its discovery as a non‐opioid and non‐steroidal analgesic.^[7]^ It has been in therapeutic use in humans for more than 30 years, and over this time, the drug was considered a well‐tolerated analgesic with relatively mild side effects until the first reports of hepatotoxic reactions under flupirtine treatment were published in 2011 and 2012, respectively.[^8^, ^9^] The severe but sporadic hepatotoxic events (reporting rate 1.68 cases/100,000 patient‐years) initially forced the responsible authorities to impose restrictions on use and, after these proved insufficient, to withdraw the authorization of flupirtine‐containing medications.^[10]^ As an alarming consequence of this withdrawal, metamizole is being increasingly used to replace flupirtine in Germany despite its known association with blood dyscrasias, yielding a growing number of reports of metamizole‐induced neutropenia.^[11]^

Retigabine (**2**, USAN: ezogabine), a close structural analogue of flupirtine, was approved in 2011 for adjunctive treatment of partial‐onset seizures in adult patients with epilepsy where other appropriate medicines were insufficiently effective.^[12]^ However, shortly after approval, it became clear that retigabine treatment occasionally resulted in blue discolouration of the skin and ocular tissues.^[13]^ Since the consequences and the reversibility of these discolourations were initially unclear, the FDA issued a boxed warning.^[14]^ Although additional investigations did not indicate that the pigment changes in the eyes affect vision, the manufacturer discontinued retigabine in 2017 because of limited and declining use.^[15]^

However, the side effects of flupirtine and retigabine responsible for failure seem to be no class effects of K_V_7.2/3 channel openers in general. In both cases, the adverse drug reactions are likely resulting from the drug‐specific metabolism, which affects especially the oxidation‐sensitive triaminoaryl metabophore. Concerning flupirtine, an *in vitro* trapping study using peroxidase mediated oxidation was able to detect glutathione conjugates, which are suggested to indicate the formation of reactive azaquinone diimine metabolites such as **3**.^[16]^ Additional evidence for the formation of reactive electrophilic metabolites of flupirtine was provided by a clinical study; after administration of flupirtine to healthy human subjects, mercapturic acid derivatives of the drug were detected in the urine, which are also likely to result from azaquinone diimine metabolites.[^17^, ^18^] Since a connection between the formation of reactive quinoid metabolites and hepatotoxicity is well known and has been demonstrated for several drugs in the past,^[19]^ the supposed reactive azaquinone diimine metabolites are very likely to be involved in flupirtine‐induced liver injury. Furthermore, the rarity of the reported hepatotoxic reactions and the lack of a dose‐dependency suggest the contribution of an additional component to the pathomechanism of flupirtine‐associated hepatotoxicity. Since genetic polymorphisms of metabolizing enzymes could be largely ruled out as a cause,^[18]^ the theory of a possible involvement of the adaptive immune system came into focus, supported by two case studies. A histological investigation concluded that clinical and histological features raise the possibility of immune‐mediated toxicity, and a genome‐wide association study identified a certain human leukocyte antigen gene as a genetic risk factor for flupirtine‐induced hepatotoxicity.[^9^, ^20^] Considering both the formation of reactive azaquinone diimine metabolites and the possible involvement of the immune system, a hapten mechanism becomes likely. After binding to an endogenous carrier protein, azaquinone diimine metabolites of flupirtine may form hapten‐protein adducts such as **5** that are able to trigger toxic autoimmune responses. The resulting immune‐mediated idiosyncratic reactions are typically very rare, not dose‐related, and often occur with a delay,^[21]^ as observed in the case of flupirtine‐associated hepatotoxicity.

In contrast, the hepatic metabolism of retigabine is not dominated by oxidation but by phase II reactions, particularly N‐glucuronidation. In fact, there is no evidence of hepatic metabolism to reactive quinone diimine metabolites or any possible hepatotoxic effects of retigabine at therapeutic doses.[^12^, ^22^] However, in a reaction associated with the presence of melanin, retigabine appears to be oxidized in other tissue types, such as ocular tissues, yielding quinone diimines **3**, which subsequently tend to dimerize. The resulting dimers can be oxidized further and thus form phenazinium structures such as **4**, identified by MALDI imaging mass spectrometry in rat eyes (but not albino rats lacking melanin) after retigabine treatment. These compounds are presumed to be responsible for the blue tissue discolouration caused by retigabine.^[23]^

Since the oxidation to reactive metabolites is likely to be the reason for the adverse drug reactions in the cases of flupirtine and retigabine, this work aimed to modify the oxidation labile triaminoaryl metabophore of the two failed K_V_7 channel openers to prevent the formation of quinone diimine or azaquinone diimine metabolites. The starting point for the structural modifications carried out in the present study was the N‐3 monocarba analogue **6** synthesized in our previous work (for the numbering of the nitrogen atoms, see Figure 1).^[24]^ Other analogues with methyl substitution at the primary amine site have also been described elsewhere (not shown).[^25^, ^26^, ^27^] In the case of analogue **6**, the removal of the primary amino function from the central pyridine ring and its replacement with a methyl group resulted in a compound that was equipotent to flupirtine, had a slightly attenuated liver cell toxicity *in vitro*, and was less sensitive to oxidation, indicated by an increased anodic peak potential in cyclovoltammetric measurements. In this work, following a ligand‐based design strategy, the different N‐1 monocarba analogue **7** was prepared, in which a methylene group replaces the secondary amino function of flupirtine to investigate possible advantageous properties in analogy to **6**. In the next step, the N‐1/3 dicarba analogues **8** and related compounds with an inverted secondary amino function and a methyl group instead of the primary amino moiety were synthesized since the N‐1 or N‐3 monocarba analogues **6** and **7**, despite increased oxidation stability of **6**, still bear the risk of azaquinone diimine formation. In contrast, the N‐1/3 dicarba analogues **8** only have one nitrogen atom directly attached to the central aromatic ring. Thus, metabolism to quinone diimines or azaquinone diimines can be excluded by design. In particular, similar retigabine analogues with scaffold **8** (X=CH) have already been described in patent literature,[^25^, ^28^] as have structurally related tetrahydroquinoline and tetrahydroisoquinoline derivatives.^[29]^ Nevertheless, this work provides a comprehensive investigation of the underlying structure‐activity relationships and further safety relevant properties such as oxidation behaviour or *in vitro* hepatotoxicity, which are currently missing.

## Results and Discussion

### Design and synthesis of N‐1 monocarba analogues

**Scheme 1:**
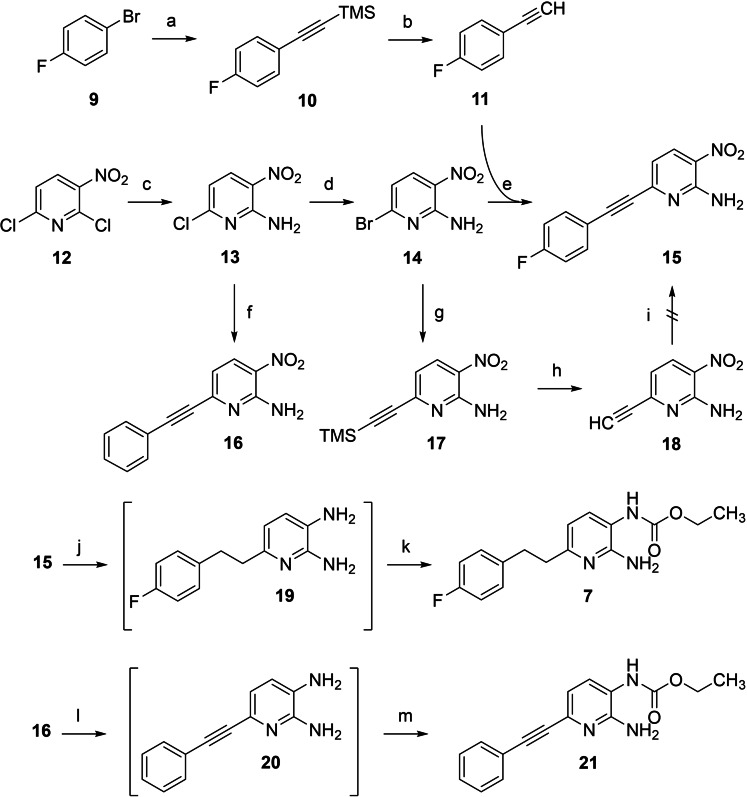
Synthesis of N‐1 monocarba analogues **7** and **21**: a) trimethylsilylacetylene, CuI, Pd(PPh_3_)_4_, TEA, 60 °C, 16 h, 99 %; b) K_2_CO_3_, MeOH, RT, 5 h, 85 %; c) NH_3_, EtOH, 0 °C–RT, 21 h, 83 %; d) HBr, AcOH, 100 °C, 8 h, 93 %; e) CuI, Pd(PPh_3_)_4_, TEA, DMF, 60 °C, 6 h, 80 %; f) phenylacetylene, CuI, Pd(PPh_3_)_4_, TEA, DMF, 60 °C, 1 h, 38 %; g) trimethylsilylacetylene, CuI, Pd(PPh_3_)_4_, TEA, DMF, 60 °C, 0.5 h, 74 %; h) K_2_CO_3_, MeOH, RT, 0.5 h, 75 %; i) 1‐bromo‐4‐fluorobenzene, CuI, Pd(PPh_3_)_4_, TEA, DMF, 60 °C; j) H_2_, Pd/C, EtOAc, 40 °C, 40 h; k) ethyl chloroformate, THF, TEA, RT, 4 h, 25 %; l) Fe, AcOH, 50 °C, 1 h; m) ethyl chloroformate, THF, TEA, RT, 7 h, 30 %.

As depicted in Scheme 1, the starting material for the synthesis of the N‐1 monocarba analogue **7** was commercially available 2,6‐dichloro‐3‐nitropyridine (**12**). The initial step introduced a primary amino function through a nucleophilic substitution reaction with ammonia, yielding aminopyridine **13**. The reaction proceeded regioselectively at the 2‐position as previously reported by Kinarivala et al.^[30]^ A halogen exchange was then carried out to obtain the aryl bromide **14** as a more reactive starting material for the subsequent Sonogashira reaction to synthesize the coupling product **15**. 4‐Fluorophenylacetylene (**11**) used for the coupling reaction was synthesized starting from 1‐bromo‐4‐fluorobenzene (**9**) also via a Sonogashira reaction by using trimethylsilylacetylene as an alkyne component to obtain the corresponding coupling product **10**. The subsequent cleavage of the trimethylsilyl protective group yielded the free terminal alkyne **11**. An alternative synthetic strategy for the preparation of **15** via intermediates **17** and **18**, investigated in a first attempt, failed in the last step since no product formation took place under standard Sonogashira coupling conditions. After the successfully conducted cross‐coupling reaction for C−C bond formation, a reduction of **15** was carried out by catalytic hydrogenation with Pd/C as a catalyst, in which both the triple bond and the nitro group were reduced simultaneously. The resulting *ortho*‐diamine **19** was not isolated but reacted directly with ethyl chloroformate under an inert atmosphere to obtain the final compound **7**.

In addition to the envisioned N‐1 monocarba analogue **7**, the synthetic route also offered the opportunity to synthesize a more linear and less flexible alkyne derivative **21**, enabling further exploration of the binding site. In this case, the 4‐fluoro substituent was omitted to simplify the synthesis, using commercially available phenylacetylene instead of 4‐fluorophenylacetylene. However, this is not expected to negatively affect the activity since a flupirtine derivative without a 4‐fluoro substituent was approximately as active as the parent compound itself in the maximum electroshock seizure test, suggesting that the 4‐fluoro substituent is not relevant for K_V_7.2/3 opening.^[31]^ To further shorten the synthesis by one step, the Sonogashira coupling was carried out starting from the aryl chloride **13**, which, however, led to a significantly reduced yield of 38 % compared to previously performed Sonogashira couplings (74–99 % yield), demonstrating the superiority of a preceding chloride to bromide exchange in the reactant as it was done for the synthesis of carba analogue **7**. The subsequent reduction of **16** was performed with iron in acetic acid as a reducing agent instead of catalytic hydrogenation with Pd/C since only the nitro group and not the triple bond should be reduced selectively. The final step in preparing the alkyne carba analogue **21** was the carbamate formation, which was carried out as described for the synthesis of **7** without prior isolation of the diamino intermediate **20**.

### Design and synthesis of N‐1/3 dicarba analogues

**Scheme 2:**
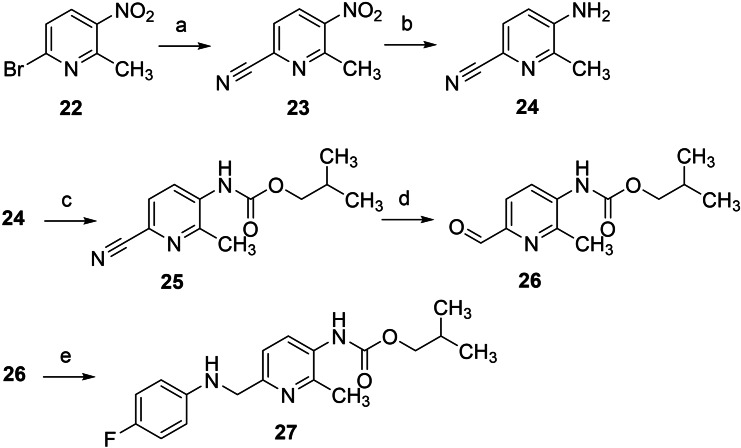
Synthesis of N‐1/3 dicarba analogue **27**: a) Zn(CN)_2_, Pd(PPh_3_)_4_, DMF, 70 °C, 24 h, 96 %; b) Fe, AcOH, CaCl_2_, EtOH, RT, 2 h, 71 %; c) isobutyl chloroformate, TEA, 4‐DMAP, DCM, RT, 4 d, 34 %; d) DIBAL−H, DCM, −85 °C, 4 h, 23 %; e) 1. 4‐fluoroaniline, DCM, RT, 5 h, 2. NaBH_4_, MeOH, RT, 1 h, 82 %.

Since the N‐1 and N‐3 monocarba analogues **6**, **7** and **21** may have beneficial properties but still bear the risk of oxidative azaquinone diimine formation, the next step, as mentioned in the introduction, was the synthesis of N‐1/3 dicarba analogues that only have one nitrogen atom left attached to the central aromatic ring. Designing these N‐1/3 dicarba analogues, the secondary amino function was not entirely replaced by a hydrocarbon linker but inverted to retain drug‐like physicochemical properties in terms of avoiding an increase in lipophilicity. Furthermore, in the case of the first compound of this series (**27**, Scheme 2), the ethyl carbamate of flupirtine and retigabine has been replaced by a 2‐methylpropyl carbamate since docking suggested space for more bulky carbamate side chains and the 2‐methylpropyl moiety in this position proved to be slightly superior to the ethyl residue in a series of retigabine analogues described in the literature.^[32]^

The synthesis of N‐1/3 dicarba analogue **27** was carried out starting from the commercially available pyridine derivative **22**. In the first step, the nitrile function was introduced via a modified version of the typically copper‐catalyzed Rosenmund‐von Braun reaction by using a palladium catalyst and Zn(CN)_2_ instead of CuCN. The nitro group of the resulting compound **23** was reduced with SnCl_2_ to obtain the aminopyridine **24**, which was subsequently reacted with 2‐methylpropyl chloroformate, yielding the carbamate **25**. In the following reaction step, the nitrile group of **25** was reduced with diisobutylaluminum hydride (DIBAL−H), which initially led to an imine intermediate (not shown) and, after acidic workup, provided the corresponding aldehyde **26**. Finally, reductive amination with 4‐fluoroaniline and NaBH_4_ was performed to obtain the desired N‐1/3 dicarba analogue **27**.

**Scheme 3:**
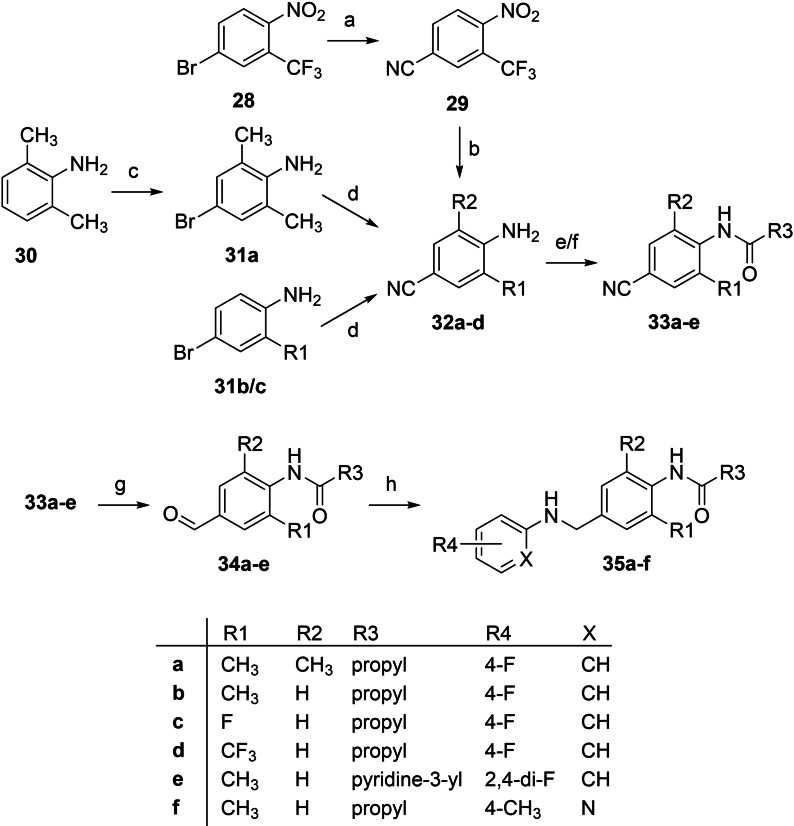
Synthesis of N‐1/3 dicarba analogues **35 a**‐**f**: a) Zn(CN)_2_, Pd(OAc)_2_, PPh_3_, DMF, 100 °C, 1.5 h, 83 %; b) SnCl_2_, EtOAc, 70 °C, 0.5 h, 99 %; c) Br_2_, DCM, −78 °C–RT, 1 h, 96 %; d) CuCN, NMP, 170 °C, 7–8 h, 42–47 %; e) 1. acyl chloride, TEA, 4‐DMAP, THF, 0 °C, 0.5 h, 2. RT/70 °C, 16 h–7 d, 56–74 % (**33 a**,**d**); f) 1. acyl chloride, TEA, DCM, RT, 1 h, 2. RT/40 °C, 3–89 h, 67–88 % (**33 b**,**c**,**e**); g) Ni, HCOOH, 80 °C, 6–8 h, 37–88 %; h) 1. amine, molecular sieves, toluene, 120 °C, 4–8 h, 2. NaBH_4_, MeOH, 1,4‐dioxane, 0 °C–RT, 17 h, 42–73 %.

In previous work, several amide derivatives of flupirtine and retigabine were found to be superior compared to carbamate analogues in terms of channel‐opening activity.[^24^, ^33^] In addition, it was shown that the carbamate function of flupirtine is affected by esterase‐mediated cleavage, which in turn means that amide derivatives could have improved metabolic stability.[^16^, ^34^] For this reason, amide derivatives of N‐1/3 dicarba analogues were intended to be synthesized in the next step. Simultaneously with the introduction of an amide group, another structural change affecting the central aromatic ring was made. Since retigabine has a 7‐fold increased potency compared to its pyridine congener flupirtine, the following N‐1/3 dicarba amide analogues have a central phenyl ring to aim for improved biological activity (Scheme 3). In the course of these structural modifications, in addition to various N‐1/3 dicarba analogues, a derivative was synthesized that bears a fluoro substituent instead of the methyl group (**35 c**). Technically speaking, this compound cannot be referred to as an N‐1/3 dicarba analogue but serves the same purpose of avoiding quinone diimine formation and should therefore be included as a related dideaza derivative here, too.

The syntheses of N‐1/3 dicarba amide analogues containing a central phenyl ring usually began with 4‐bromoanilines **31 a**‐**c**. In the case of a 2,6‐dimethylated derivative, the bromoaniline **31 a** was prepared by bromination of the corresponding aniline **30**. The bromoanilines were then converted into the nitriles **32 a**‐**c** in a Rosenmund‐von Braun reaction using CuCN in a dual function as a catalyst and cyanide source. In the case of a trifluoromethylated analogue, the synthesis was carried out differently, starting from the nitrobenzene **28**, which was first converted into the nitrile **29** and then reduced to the aniline **32 d** with SnCl_2_. In this case, the Rosenmund‐von Braun reaction was again performed in a palladium‐catalyzed version providing a better yield (83 %) compared to the copper‐catalyzed procedure used before (43–47 %). The next step was an amide coupling for all derivatives. Since the 4‐aminobenzonitriles **32 a**‐**d** used as reactants proved to be unreactive amine components due to the electron‐withdrawing properties of the cyano group and the steric hindrance by ortho substituents, it was partially necessary to apply elevated temperatures of up to 70 °C, long reaction times of up to seven days, or additional use of a catalyst (4‐DMAP). The nitrile function of the amides **33 a**‐**e** obtained in this way was subsequently reduced to obtain the corresponding aldehydes **34 a**‐**e** by using nickel in formic acid. Finally, reductive amination with sodium borohydride and different aniline reactants was carried out to prepare the desired N‐1/3 dicarba analogues **35 a**‐**f**.

A total of six derivatives (**35 a**‐**f**) were synthesized in this series of N‐1/3 dicarba analogues with amide substituent and a central phenyl ring, some of which have additional structural changes. Instead of the methyl group, a trifluoromethyl group or, as already mentioned, a fluoro substituent was introduced in compounds **35 c** and **35 d**, respectively. In addition, a pyridine ring was evaluated both as an alternative bulkier amide substituent in the case of **35 e** and a more hydrophilic replacement of the 4‐fluorophenyl ring in analogue **35 f**. In the case of **35 f**, the putatively metabolically most labile position of the pyridine ring was substituted with a methyl group. Moreover, a second ortho methyl group has already proven to be beneficial for the activity of some analogues of flupirtine and retigabine known from the literature and was therefore attached to the central ring of **35 a**.[^27^, ^35^]

Since it was expected that the N‐1/3 dicarba analogues **35 a**–**e** with clog*S* values in the range of −3.94 to −4.63 are considerably less soluble in water than flupirtine and retigabine (clog*S*=−3.16/−3.41),^[36]^ it was investigated whether the secondary amino group allows salt formation. With compound **35 d** as an example, it was successfully shown that a stable hydrochloride salt could be prepared. However, it was not verified whether salts of weaker acids, such as maleic acid, which was used for salt formation in the case of flupirtine, are also possible.

**Scheme 4:**
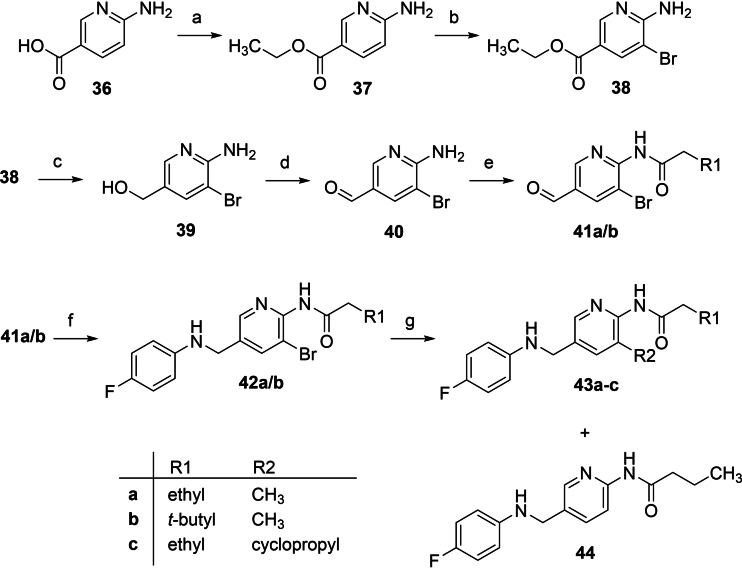
Synthesis of N‐1/3 dicarba analogues **43 a**‐**c**: a) EtOH, SOCl_2_, 90 °C, 18 h, 88 %; b) NBS, THF, 0 °C–RT, 17 h, 77 %; c) LiAlH_4_, THF, RT, 5 h, 54 %; d) MnO_2_, toluene, 80 °C, 2 h, 41 %; e) acyl chloride, DIPEA, DCM, 0 °C–RT, 16.5–24.5 h 48–50 %; f) 1. 4‐fluoroaniline, toluene, molecular sieves, 120 °C, 5 h, 2. NaBH_4_, MeOH, 1,4‐dioxane, 0 °C, 17 h, 57–74 %; g) trimethylboroxine or cyclopropylboronic acid, Pd(PPh_3_)_4_, Na_2_CO_3_, 1,4‐dioxane, H_2_O, 140 °C, μW irradiation, 75 min, 15–43 %.

While it was an obvious choice to replace the basic heterocycle of flupirtine with a phenyl ring, as in the case of N‐1/3 dicarba analogues **35 a**–**f**, the pyridine ring has attractive physicochemical features. For this reason, it was investigated whether an inverted pyridine ring, which is rotated by 180° relative to the substitution pattern of flupirtine, might be favourable. In the following, a synthetic route is presented, leading to these novel N‐1/3 dicarba analogues with amide substituent and inverted pyridine ring (**43 a**–**c**, Scheme 4). Since the methyl group is introduced in the last reaction step, the synthetic pathway also offered the possibility of replacing the methyl group with variable hydrocarbon residues.

The syntheses of the N‐1/3 dicarba analogues with an inverted pyridine ring were carried out starting from 6‐aminonicotinic acid (**36**), which was esterified in the first reaction step to obtain the ethyl ester **37**. The subsequent bromination was performed by reacting **37** with *N*‐bromosuccinimide, yielding compound **38**. To obtain the aldehyde **40**, a two‐step procedure was carried out. In the first step, LiAlH_4_ was used to reduce the ethyl ester **38** to the corresponding alcohol **39**, which was then reoxidized in the second step by using activated MnO_2_, yielding the desired aldehyde **40**. This reduction‐oxidation procedure was followed by the synthesis of the amides **41 a**/**b** carried out by employing the corresponding acyl chlorides. Subsequently, the aldehyde group of the resulting amides was coupled with 4‐fluoroaniline in a reductive amination procedure to obtain the secondary amines **42 a**/**b**. Due to their structural similarity to the N‐1/3 dicarba analogues, which also prevents oxidation to azaquinone diimines, one of the bromo intermediates (**42 a**) was also subjected to biological testing. In the last step, the methyl group was introduced via a Suzuki reaction with trimethylboroxine as the methyl source, a cheaper and fairly soluble anhydride alternative for methylboronic acid, thus yielding the final compounds **43 a**/**b**. In the case of compound **43 b**, a bulkier 3,3‐dimethylbutanamide substituent was introduced as an amide sidechain. This 3,3‐dimethylbutanamide moiety is contained, for example, as a structural element in Lu AA41178, a K_V_7 channel opener that demonstrated efficacy in preclinical models of epileptic seizures and psychiatric disorders but is not devoid of the risk of azaquinone diimine formation.^[35]^ In addition, compound **43 c** with a cyclopropyl substituent instead of the methyl group was synthesized to evaluate possible space for a sterically more demanding hydrocarbon moiety in this part of the scaffold. The synthesis was conducted analogously to the methyl compounds by using cyclopropylboronic acid. The cyclopropyl residue in this particular case has the advantage of not increasing the lipophilicity as much as other branched hydrocarbon residues, such as a 2‐propyl group, because of the increased π‐character of its C−C bonds.^[37]^ Unfortunately, all Suzuki couplings carried out resulted in only poor to moderate yields (15–43 %). This was partly due to the formation of various by‐products. The debrominated compound **44** could be isolated and characterized as one of these by‐products in the synthesis of **43 a** and **43 b**, respectively.

**Scheme 5:**
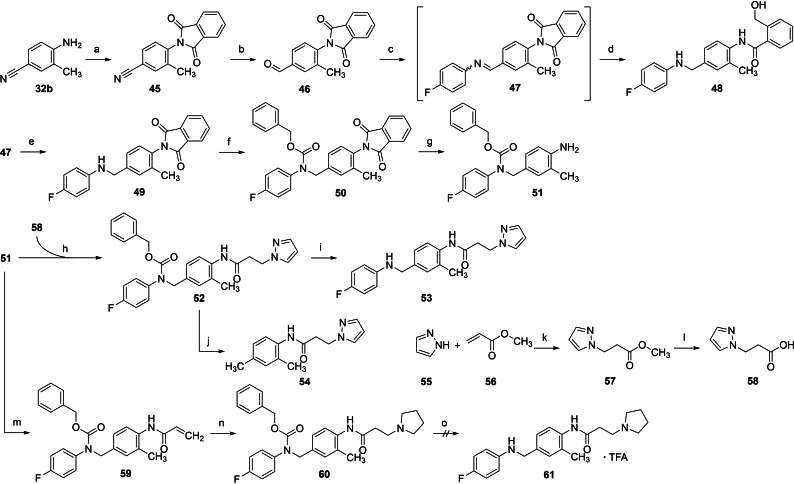
Synthesis of N‐1/3 dicarba analogues **48** and **53**: a) Phthalic anhydride, AcOH, 130 °C, 5 h, 83 %; b) Ni, HCOOH, 80 °C, 6 h, 81 %; c) 4‐fluoroaniline, molecular sieves, toluene, 120 °C, 6 h; d) NaBH_4_, MeOH, 1,4‐dioxane, 0 °C–RT, 17 h, 68 % (c+d); e) H_2_, Pd/C, EtOAc, RT, 6 h, 67 % (c+e); f) Cbz‐Cl, DIPEA, DCM, RT, 4 h, 84 %; g) N_2_H_4_, H_2_O, THF, RT, 16 h, 98 %; h) DIC, HOBt, DMF, RT, 16 h, 69 %; i) HBr, AcOH, RT, 2 h, 89 %; j) H_2_, Pd/C, EtOAc, RT, 20 min, 61 %; k) DBU, MeCN, RT, 16 h, 76 %; l) KOH, H_2_O, MeOH, RT, 16 h, 74 %; m) acyl chloride, DIPEA, DCM, 0 °C, 2.5 h, 94 %; n) pyrrolidine, EtOH, 60 °C, 7 h, 87 %; o) H_2_, Pd/C, MeOH, RT, 2 h, 50 %.

Sterically demanding amide sidechains, such as the already mentioned 3,3‐dimethylbutanamide moiety but also benzamides, or phenylacetic acid amides, have proven advantageous in several amide‐type K_V_7.2/3 channel openers derived from flupirtine and retigabine.[^24^, ^35^, ^38^] Consequently, these amide residues are also of interest for the N‐1/3 dicarba analogues. However, an increase in lipophilicity, which is associated with these bulky amide moieties, should be avoided as far as possible to ensure at least a moderate aqueous solubility of the relatively lipophilic N‐1/3 dicarba analogues. For this reason, it was investigated whether less lipophilic but still bulky heterocycles are tolerated as part of the amide sidechain. A first attempt to introduce a heteroaromatic amide substituent was compound **35 e**, which has a pyridine ring incorporated in the nicotinamide structure. In the following, a modified synthesis strategy is presented, which was established to couple other aromatic and non‐aromatic heterocycles to the amide sidechain (Scheme 5).

The starting material was the above‐mentioned compound **32 b**, the amino group of which was protected in the first step by reaction with phthalic anhydride, forming the phthalimide **45**. Afterwards, the corresponding aldehyde **46** was synthesized analogously to compounds **34 a**–**e** by treating the nitrile with Raney nickel in formic acid. The reductive amination procedure with sodium borohydride, which had previously been used, was unsuccessful in this case since it led to a reductive ring‐opening of the phthalimide structure yielding **48**. Alternatively, a catalytic hydrogenation procedure with Pd/C as a catalyst was applied for the reductive amination to obtain the desired product **49**. Reductive cleavage of the benzylamine partial structure, as initially feared, was not observed under these conditions. The resulting secondary amine **49** was then protected by reacting the amino group with benzyl chloroformate, forming the benzyl carbamate **50**. Subsequently, the phthalimide protective group was cleaved by hydrazine. The resulting aniline **51** could then be modified by the attachment of different amide sidechains. Compound **52** with a 3‐(1*H*‐pyrazol‐1‐yl)propanamide sidechain was realized by coupling the corresponding carboxylic acid **58** with DIC and HOBt as coupling reagents. The required carboxylic acid **58** was synthesized from 1*H*‐pyrazole and methyl acrylate via an aza‐Michael addition and subsequent hydrolysis of the resulting ester **57**. Finally, the Cbz group of **52** had to be removed to release the secondary amino group. The first attempt of a reductive cleavage of the protecting group failed because the cleavage also affected the benzylamine partial structure, yielding **54**. Alternatively, the deprotection was realized by applying an acidic cleavage protocol utilising HBr in acetic acid to obtain the final compound **53**. The undesired reaction product **48**, which arose as a result of the reductive ring‐opening of the phthalimide structure, was also subjected to biological testing since it represents an N‐1/3 dicarba analogue with a bulky but not too lipophilic amide sidechain.

As a second heterocycle‐containing amide sidechain, a 3‐(pyrrolidin‐1‐yl)propanamide moiety was considered. For this purpose, compound **51** was reacted with acryloyl chloride to give the corresponding acrylamide **59**. The pyrrolidine moiety was then introduced again via an aza‐Michael addition, yielding **60**. Finally, the Cbz group was cleaved hydrogenolytically by using Pd/C as a catalyst. The resulting product had to be purified by preparative RP‐HPLC and was obtained as the trifluoroacetic acid salt after lyophilization of the product containing fraction. In contrast to the undesired conversion of **52** to **54**, ^1^H‐NMR spectroscopic data suggest the benzylamine partial structure was not or, considering the moderate yield, at least only partially cleaved under these conditions. Unfortunately, the resulting trifluoroacetic acid salt was found to be highly hygroscopic, transforming from an amorphous solid to a sticky resin within minutes after contact with air. A hydrochloride salt, which was stable and non‐hygroscopic in the case of analogue **35 d**, was also evaluated here but proved to be similarly hygroscopic as the trifluoroacetic acid salt, and the free base turned out to be a viscous oil that did not solidify. Therefore, compound **61** was excluded from further characterization and biological testing.

### Evaluation of K_V_7.2/3 opening activity

The K_V_7 channel opening activity was tested on HEK‐293 cells overexpressing the K_V_7.2/3 channel. For this purpose, a commercially available assay was applied, which uses the permeability of potassium channels for thallium ions. In this assay, a thallium‐sensitive fluorescent dye is used, which, when bound to Tl^+^ passed through potassium channels, produces a fluorescent signal. The intensity of the signal is proportional to the number of open potassium channels and therefore provides information about the functional activity of the K_V_7.2/3 channels. The corrected fluorescence intensity as a function of the compound concentration was used to determine the EC_50_ values, which express the concentration required to reach the half‐maximal fluorescence signal. Moreover, the efficacy was determined, which is the percentage of the maximal fluorescence signal induced by a compound relative to the maximal fluorescence signal induced by flupirtine. A representative dose‐response curve is shown in Figure 4. For a summary of the activity data, see Table 1.

**Table 1:** K_V_7.2/3 channel opening activity, *in vitro* toxicity, and log*D*_7.4_ values of the synthesized analogues **7–53** in comparison to flupirtine and retigabine.^[a]^

		HEK‐293		TAMH		HEP‐G2	
Entry	Log*D*_7.4_	EC_50_^[b]^ [μM]	Efficacy [%]	LD_50_^[c]^ [μM]	LD_25_^[d]^ [μM]	LD_50_^[c]^ [μM]	LD_25_^[d]^ [μM]
Flu	2.1	1.837±0.844	100	487±51	103±47	547±111	74±40
Ret	2.1	0.249±0.052	119±7	>400	>400	>400	269±166
7	3.0	>10.000	–	>63	25±7	>63	27±16
21	3.0	–^[e]^	–^[e]^	>125	63±12	>125	>125
27	3.4	4.123±3.945	94±15	>250	76±15	>250	136±38
35 a	3.0	0.143±0.020	143±7	>125	>125	>125	>125
35 b	2.9	0.675±0.276	176±14	>125	>125	>125	88±9
35 c	3.0	1.505±0.364	167±16	>63	>63	>63	>63
35 d	3.0	0.362±0.093	156±14	>125	>125	>125	75±17
35 e	3.0	–^[e]^	–^[e]^	>125	41±11	107±16	61±7
35 f	2.3	7.858±2.167	70±9	>125	>125	>125	>125
42 a	2.7	5.454±1.696	163±23	>63	>63	>63	>63
43 a	2.3	4.840±2.907	89±32	>125	110±54	>125	46±24
43 b	3.0	0.677±0.419	153±12	>125	>125	>125	66±27
43 c	2.8	2.015±1.195	138±20	>125	76±52	>125	55±17
48	2.9	–^[e]^	–^[e]^	>125	70±10	84±6	80±7
53	2.9	2.361±0.543	144±42	>63	>63	>63	>63

[a] Log*D*_7.4_ values were estimated by applying an HPLC‐based method. EC_50_ values were determined in HEK‐293 cells overexpressing the K_V_7.2/3 channel. LD values were obtained by MTT assay after 24 h of exposure. EC_50_ and LD values are means with corresponding standard deviations of at least three independent determinations. [b] Required concentration to reach half‐maximal K_V_7.2/3 channel opening activity. [c] Required concentration to reduce cell viability by 50 % compared to untreated controls. [d] Required concentration to reduce cell viability by 25 % compared to untreated controls. [e] Inactive up to a concentration of 20 μM.

**Figure 2:**
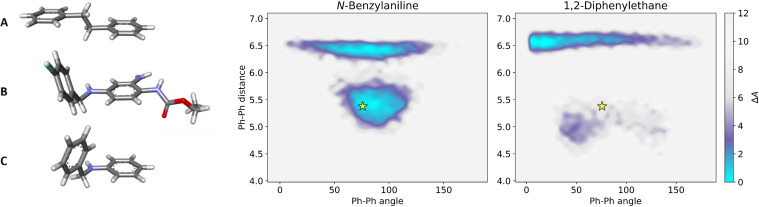
Left: Crystal structures of 1,2‐diphenylethane (A) and *N*‐benzylaniline (C) in comparison to the conformation of retigabine in the bound state (B) obtained from a cryo‐EM structure of K_V_7.2 in complex with retigabine. Right: Helmholtz free energy landscapes for the conformational space analysis of *N*‐benzylaniline and 1,2‐diphenylethane in octan‐1‐ol, as representative scaffolds for the investigated ligand structures. The star denotes the corresponding ring angle and ring distance in retigabine bound to K_V_7.2.

In contrast to the N‐1 monocarba analogue **6**, which served as the initial inspiration for synthesizing other carba analogues, the N‐1 monocarba analogue **7** proved considerably less potent than flupirtine; up to a concentration of 20 μM, the concentration‐response curve of **7** (not shown) rose slightly without reaching a maximum and always remained below the curve of flupirtine. An exact EC_50_ value could not be determined. However, the compound was clearly less active than flupirtine. The alkyne derivative **21** was completely inactive even up to a concentration of 20 μM. Both results were not necessarily to be expected since a recently published cryogenic electron microscopy (cryo‐EM) structure of K_V_7.2 in complex with retigabine does not indicate any specific interactions of the secondary amino group of retigabine with the binding site.^[39]^ However, the *N*‐benzylaniline partial structure of retigabine appears to be in an angled, synclinal conformation in the bound state (Figure 2B), which suggests conformational causes may be responsible for the drastic loss in activity of the N‐1 monocarba analogues **7** and **21**. This is obvious in the case of the alkyne derivative **21**, as it is built linearly due to the acetylene linker and consequently differs significantly from the angled conformation described for retigabine in the bound state. In contrast to **21**, a synclinal conformation similar to retigabine would theoretically be conceivable for compound **7**. However, the crystal structure of 1,2‐diphenylethane (Figure 2A),^[40]^ as a simplified model for the ethylene linker of **7**, suggests that a straight, antiperiplanar conformation might be energetically preferred. In contrast, the crystal structure of *N*‐benzylaniline (Figure 2C) reveals an angled, synclinal conformation due to the aniline nitrogen atom geometry,^[41]^ thus resembling the conformation of retigabine in the bound state (Figure 2B). These assumptions regarding the conformation were supported by molecular dynamics simulations, which more closely reflect the conformational states in solution. As can be seen in Figure 2, the Helmholtz free energy landscape of *N*‐benzylaniline shows a minimum in the region matching the ring angle and ring distance of retigabine bound to K_V_7.2, whereas this specific conformation is clearly energetically unfavourable for 1,2‐diphenylethane. Applied to the current carba analogues, these findings imply that methyleneamino linked compounds such as flupirtine and retigabine are conformationally preorganized. In contrast, derivatives with an ethylene linker such as carba analogue **7** bind rather poorly to the binding site since the entropic costs of conformational reordering may be higher than for methyleneamino linked compounds.

In contrast to the N‐1 monocarba analogues **7** and **21**, several N‐1/3 dicarba analogues and structurally related compounds showed a significantly increased K_V_7.2/3 opening activity compared to flupirtine, with analogue **35 a** exceeding retigabine in both terms of potency and efficacy. Overall, the small series of N‐1/3 dicarba analogues synthesized in the present study provided valuable insights into the structure‐activity relationships of this substance class, which will be discussed below.

The inverted methyleneamino linker of the N‐1/3 dicarba analogues, instead of a possible ethylene linker as investigated in the case of N‐1 monocarba analogue **7**, was originally chosen in order to retain balanced and drug‐like lipophilicity. In retrospect, this turned out to be positive in two ways since, in addition to the lipophilicity, the biological activity was also not negatively affected, as in the case of the ethylene linker of **7**. Presumably, the inverted methyleneamino linker of the N‐1/3 dicarba analogues also favours an angled conformation like the original linker of flupirtine and retigabine since it is still an *N*‐benzylaniline partial structure, albeit inverted.

**Figure 3:**
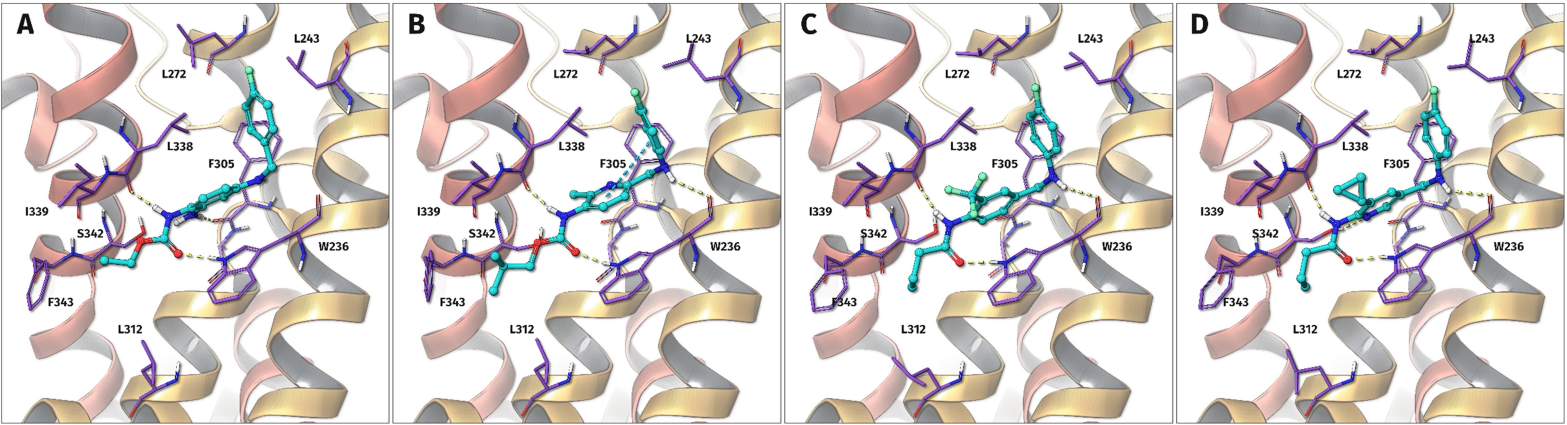
Predicted induced‐fit docking poses of retigabine (A), **27** (B), **35 d** (C), and **43 c** (D) in the binding pocket of the heterotetrameric K_V_7.2/3 potassium channel. The hydrogen bonds (yellow dashed lines) of the carbamate group are mostly preserved for all compounds, while the alkyl chain binds to a larger hydrophobic pocket. Inverting the secondary amine nitrogen atom position results in a hydrogen bond interaction with the backbone carbonyl oxygen atom of W236. The hydrogen bond interactions to S342 and F305, formed by flupirtine and retigabine, are not mandatory for activity. The primary amino group can therefore be replaced by hydrophobic substituents to form interactions with a small pocket between I339 and L338. An inversion of the pyridine ring realized in **43 c** enables the pyridine nitrogen atom to act as a hydrogen bond acceptor for S342.

In order to investigate the binding mode of the N‐1/3 dicarba analogues and to shed light on the possible role of the inverted methyleneamino linker, a molecular docking study was performed. Figure 3 shows the docking poses of retigabine compared to selected analogues (**27**, **35 d**, and **43 c**). As can be seen, the general orientation in the binding pocket, as well as the conformation of the N‐1/3 dicarba analogues, is roughly comparable to retigabine. However, a significant difference is a newly occurring hydrogen bond interaction with the backbone carbonyl oxygen atom of W236, which is enabled by the inversion of the secondary amino group.

In line with our previous findings concerning N‐3 monocarba analogue **6**, the methyl group as a replacement for the primary amino function of flupirtine and retigabine is also well‐tolerated in the N‐1/3 dicarba analogues, although the above‐mentioned cryo‐EM structure of K_V_7.2 in complex with retigabine suggests the primary amino group may be involved in hydrogen bonds to S342 and F305. Possibly, these interactions are not mandatory for the activity, or alternatively, their loss is compensated by other effects, such as the increased compound lipophilicity. A replacement of the primary amino function with a methyl group or other non‐polar substituents along with additional structural changes generally led to more lipophilic compounds as reflected by higher log*D*_7.4_ values throughout all N‐1/3 dicarba analogues compared to flupirtine and retigabine (Table 1). A possible correlation between increased lipophilicity and improved activity was already observed in our previous work but could not be clearly proven since other structural features aside from the compound lipophilicity also affect the activity.^[33]^ Nevertheless, lipophilicity seems to be a relevant variable influencing activity, and a possible reason might be the location of the K_V_7.2/3 binding site, which is situated in the area where the channel is embedded in the cell membrane. It has not yet been clarified how ligands find their way to the binding pocket. However, a plausible possibility is that potential K_V_7 openers first have to diffuse into the lipid double layer in order to reach their site of action, which requires a certain degree of lipophilicity.

Halogen substituents to replace the methyl group were also tolerated. However, as revealed by comparing the activity data of **35 b** and **35 c**, a smaller fluoro substituent was inferior to a methyl group. In contrast, the comparison of **42 a** and **43 a** suggests a larger bromo substituent has a similar to slightly superior effect on the K_V_7.2/3 opening activity compared to a methyl group. The likewise electron‐withdrawing trifluoromethyl residue was clearly superior to both the fluoro substituent and the methyl group, as the comparison of **35 b**, **35 c**, and **35 d** demonstrates. Furthermore, the binding site appears to offer space for bulkier hydrocarbon residues than a methyl group. The introduction of a cyclopropyl moiety in compound **43 c** led to a 2.4‐fold increase in potency and an increase in efficiency of 49 % compared to the corresponding methyl analogue **43 a**. In this case, too, the improvement in activity is possibly related to an increase in lipophilicity since the log*D*_7.4_ value increased at the same time from 2.3 to 2.8 when a cyclopropyl residue replaced the methyl group. Moreover, molecular docking reveals a slightly different binding pose of **43 c**. As shown in Figures 3C and 3D, larger lipophilic substituents than a methyl group such as the cyclopropyl moiety as well as the trifluoromethyl group, which is suggested to be about as bulky as an ethyl residue,^[42]^ face away from the binding site. This is contrary to a methyl group (Figure 3B), which is oriented in the opposite direction, thus occupying a lipophilic pocket between I339 and L338 that might be too small for larger substituents. However, the orientation of sterically more demanding substituents facing away from the binding pocket and the resulting marginal rotation of the central aromatic ring may be advantageous, as suggested by the increased potency of **35 d** and **43 c** compared to **35 b** and **43 a**.

**Figure 4:**
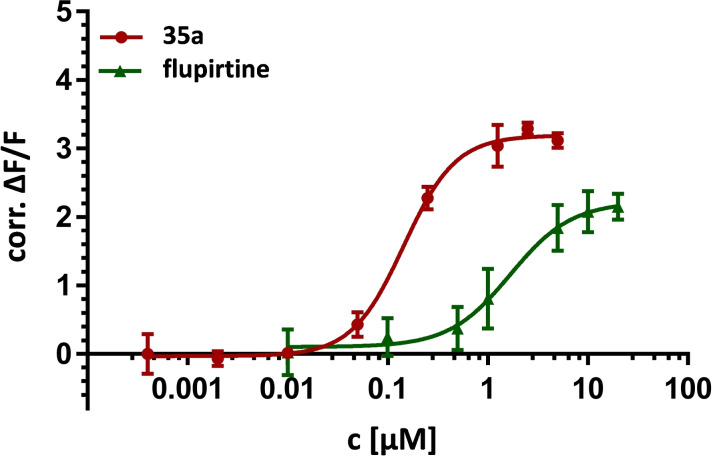
Comparison of the concentration‐activity curves of **35 a** and flupirtine.

As with several analogues known from the literature, an additional methyl group in ortho position to the amide function proved advantageous.[^27^, ^35^] The resulting compound **35 a**, with an EC_50_ value of 0.143 μM, was the most active in the present set of substances, clearly reflected by the left‐shifted concentration‐activity curve in comparison to flupirtine displayed in Figure 4. This is in analogy with our recently published series of inverted amide derivatives of flupirtine and retigabine with benzamide/nicotinamide scaffold, where an additional ortho methyl group even made the difference from complete inactivity to submicromolar activity (not shown).^[43]^ As we have demonstrated, the positive effect of the methyl group and the resulting ortho disubstitution is probably attributed to steric interactions, which force the amide group to rotate out of the plane, favouring a dihedral angle that is in good agreement with the predicted dihedral angle in the bound state. However, the positive effect on the activity is not quite as pronounced for anilide **35 a** as for the previously published benzamide/nicotinamide series. Nevertheless, the methyl group in **35 a** led to a 4.7‐fold increase in potency compared to **35 b**, lacking the additional ortho methyl group.

In agreement with experience made with previously prepared analogues of flupirtine and retigabine bearing different scaffolds, an exchange of the carbamate function for an amide group was possible without drastically impairing the activity.[^24^, ^33^] This was confirmed by molecular docking since the hydrogen bonds of the carbamate group of flupirtine and retigabine are preserved for amide derivatives (Figure 3C/D). Similar to the ethoxycarbonylamino sidechain, the butanoylamino side chain of **35 d** and **43 c** binds to a larger hydrophobic pocket, which apparently offers additional space for sterically more demanding amide residues. In line with this observation, a bulky and lipophilic side chain was found to increase the activity of the N‐1/3 dicarba analogues further since it presumably enables a better fit in the corresponding hydrophobic cavity. This becomes evident when comparing **43 a** and **43 b**, where a 3,3‐dimethylbutanoylamino side‐chain caused a 7.2‐fold increase in potency compared to a butanoylamino sidechain and, at the same time, increased the log*D*_7.4_ value from 2.3 to 3.0. In contrast, a bulky but less lipophilic amide moiety such as the 3‐(1*H*‐pyrazol‐1‐yl)propanoylamino residue of **53**, which left the log*D*_7.4_ value unchanged compared to a butanoylamino sidechain (**35 a**), seems not to be an adequate amide residue to occupy the hydrophobic cavity since it led to a 3.5‐fold decrease in potency.

The incorporation of a pyridine ring or a benzyl alcohol moiety into the lateral molecule parts also proved problematic and led to inactive (**35 e**, **48**) or weakly active substances (**35 f**). While docking suggests the benzyl alcohol moiety of **48** does not fit the binding pocket at all, the pyridine rings of **35 e** and **35 f** principally fit but may weaken hydrophobic interactions in the area of the lateral lipophilic pockets, which seem vital for a sufficient binding. Possibly, the same applies to **53**, where a 3‐(1*H*‐pyrazol‐1‐yl)propanoylamino side chain had a detrimental effect on the activity, too, as mentioned above. However, in the case of **53**, at least a fair K_V_7.2/3 opening activity was retained.

If at all, a pyridine ring is most likely to be tolerated in the central part of the molecule (**27**, **42 a**, **43 a**–**c**), although it became clear that a more lipophilic phenyl ring is superior here, too, leading to substances with increased potency (**35 a**–**d**). However, through further structural modifications, such as a bulky, lipophilic amide sidechain, the potent compound **43 b** with an EC_50_ value of 0.677 μM could be obtained despite the presence of a pyridine ring as the central molecule part. Interestingly, the orientation of the pyridine nitrogen atom relative to the substitution pattern may be of certain importance here. An inverted pyridine ring as in **43 a**–**c** seems to be advantageous for binding since docking suggests the nitrogen atom can act as a hydrogen bond acceptor for S342 in this type of compounds (Figure 3B vs 3D). However, this observation could not be clearly confirmed by activity data since direct comparison of **27** and **43 a** with regard to the influence of the inverted pyridine ring is not valid due to the different carbamate/amide side chains.

### Evaluation of in vitro hepatotoxicity

Idiosyncratic toxicity, as suspected for flupirtine‐induced liver injury, is an ongoing challenge in drug discovery since it is still very difficult to predict in an *in vitro* assay due to the multifactorial causes.^[44]^ Some experimental assays have been developed to evaluate idiosyncratic hepatotoxicity, but they include complex procedures and are partly not sufficiently validated.^[45]^ Furthermore, too little is known for sure about the pathomechanism of flupirtine‐induced liver injury to select an appropriate model for idiosyncratic hepatotoxicity. For these reasons, the toxicity evaluation of the newly synthesized compounds was limited to determining the general toxicity in cultured liver cells by using a standard MTT based cell viability assay, which, however, does not consider the possible involvement of an immunological component in the flupirtine‐induced hepatotoxicity as mentioned in the introduction. Nevertheless, the resulting toxicity data are highly relevant since the focus of our work is on the development of safe K_V_7.2/3 openers, and consequently, the new carba scaffolds have to be examined for potential toxicological risks. The human and mouse cell lines Hep‐G2 and TAMH, respectively, that were used for the assay are of hepatic origin and well established for *in vitro* hepatotoxicity testing.^[46]^ The detection of cell viability with the MTT assay is based on the mitochondrial reduction of the yellow, water‐soluble 3‐(4,5‐dimethylthiazol‐2‐yl)‐2,5‐diphenyltetrazolium bromide (MTT) to a blue‐violet, water‐insoluble formazan in living cells, which can be quantified by colourimetric measurement.^[47]^ A limiting factor in the toxicity testing was the aqueous solubility of the substances; most of the compounds proved to have only low to moderate toxicity, and as a result, LD_50_ values could only be determined in some cases since the required high concentrations could not be achieved without the substances precipitating. For this reason, LD_25_ values were calculated that indicate the concentration at which cell viability was reduced to 75 % (Table 1).

To our knowledge, no hepatotoxic effect is known for retigabine, and this was also observed in the MTT assay. LD_50_ values could not be determined but are above 400 μM and therefore considered as uncritical. The same applies to flupirtine with LD_50_ values of around 500 μM. Although LD_25_ values in the range of 74–103 μM were calculated, these should not be assessed as critical either since the peak plasma concentration after oral administration of 400 mg flupirtine was determined as 1.6 μM (modified release dosage form once daily for 8 days).^[18]^ Considering this value as a relevant physiological concentration range, it becomes clear that flupirtine itself may exhibit only very mild, if any, acute hepatotoxicity at therapeutic dosing; otherwise, it would have hardly been approved as an analgesic. Despite this apparent lack of *in vitro* toxicity, flupirtine proved to be severely hepatotoxic for a very small number of patients. However, as already mentioned, other influencing factors that are not considered in an artificial *in vitro* toxicity assay may contribute to the pathomechanism.

Similar to flupirtine and retigabine, most of the novel carba and N‐1/3 dicarba analogues demonstrated a rather uncritical level of *in vitro* hepatotoxicity. When comparing the LD_25_ values, predominantly, some of the less active or inactive substances performed slightly worse than flupirtine. This particularly affected the N‐1 monocarba analogue **7** and the inactive N‐1/3 dicarba analogues **35 e** and **48**, which both share an aromatic amide sidechain as a common structural feature. However, most of the remaining N‐1/3 dicarba analogues, including the submicromolar active compounds **35 b**, **d**, and **43 b**, exhibited a weakly pronounced and uncritical level of liver cell toxicity comparable to flupirtine. The most active compound **35 a**, with LD_25_ values above 125 μM in both cell lines, tends to be slightly less toxic than flupirtine. It must also be taken into account that the last‐mentioned substances demonstrated superior potency and efficacy compared to flupirtine. Consequently, these analogues may have a wider therapeutic safety window when considering the similar uncritical level of toxicity. In addition, they could potentially be effective at reduced doses, thus further minimizing the risk of toxic reactions since a high daily dose is generally considered a risk factor for oral drugs to induce hepatotoxicity.^[48]^ In conclusion, the *in vitro* toxicity data confirmed our initial design strategy to develop safer K_V_7.2/3 channel openers. In addition to ortho or para nitrogen atoms, an aniline moiety was also avoided, which is generally considered harmful since it can potentially be metabolized to toxic *N*‐hydroxylamine and nitroso metabolites.^[49]^ Therefore, two structural alerts were simultaneously removed by the redesign of flupirtine and retigabine to N‐1/3 dicarba analogues.

### Evaluation of oxidizability

Oxidation stability is a key characteristic for new analogues of flupirtine and retigabine since the oxidation‐sensitive triaminoaryl scaffold seems to be the main weak point of both drugs. For this reason, all newly synthesized compounds were examined for their oxidizability. While flupirtine is probably oxidized enzymatically, the oxidation of retigabine is supposed to take place via a melanin‐associated reaction, or at least melanin may function as an adsorbent for oxidation products.^[50]^ Since two different oxidation mechanisms have to be considered, cyclic voltammetry seemed a practicable solution to evaluate the general oxidizability of the novel analogues. This method has already been used to investigate the electrochemical behaviour of other oxidatively toxified drugs such as paracetamol or diclofenac.^[51]^ Furthermore, a recent study concluded that electrochemical oxidation is a suitable approach for a preliminary investigation of the oxidative behaviour of drug candidates since the profile of generated oxidation products is roughly comparable to *in vitro* microsomal incubation.^[52]^

**Figure 5:**
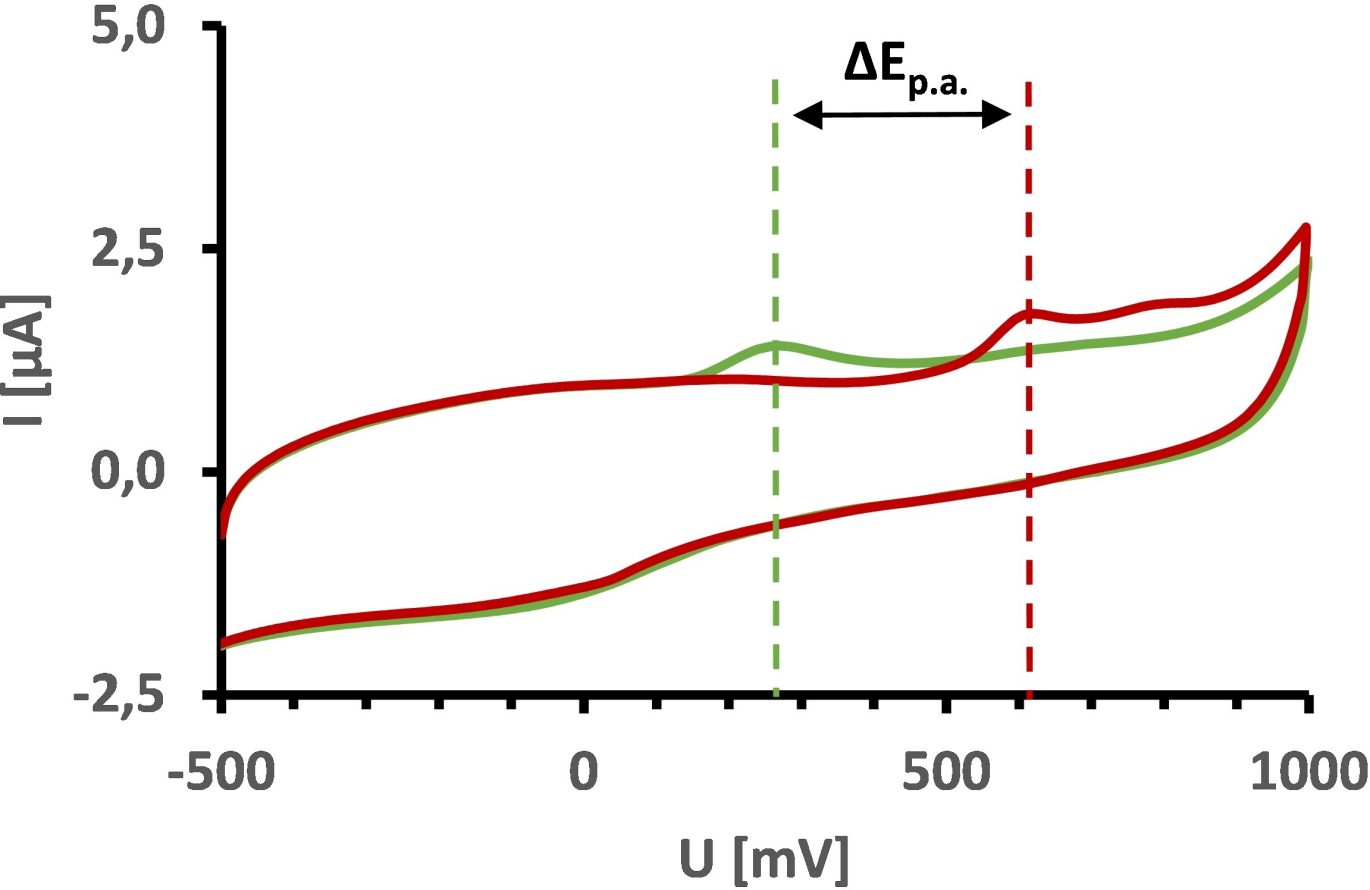
Cyclic voltammograms of retigabine (green) and the N‐1/3 dicarba analogue **35 a** (red) in 0.1 M Tris‐HCl buffer (pH 7.4) with anodic peak potential values (E_p.a._) indicated by dashed lines.

Figure 5 shows a representative cyclic voltammogram of compound **35 a** versus retigabine. The anodic peak potentials, which are marked by dashed lines, indicate that **35 a** with an oxidation potential of 633 mV is significantly more oxidation‐stable compared to retigabine (E_p.a._ 260 mV) and flupirtine (E_p.a._ 306 mV, not shown), respectively. This trend is consistent for all carba analogues (Table 2), confirming the initial design strategy of replacing amino substituents with alkyl substituents to reduce oxidation sensitivity. For three derivatives (**35 e**, **48**, **53**), however, no anodic peak potential could be determined. In contrast to the other analogues, these compounds have an aromatic amide sidechain; this structural feature might be the reason for the apparent lack of anodic peak potentials since the large planar molecular fraction may lead to reduced aqueous solubility, hence making it impossible to obtain a cyclic voltammogram.

**Table 2:** Investigations on the oxidizability of flupirtine, retigabine, and the synthesized carba analogues **7–53**, including anodic peak potentials (E_p.a._) determined by cyclic voltammetry and *in silico* molecule quinone formation scores (MQS) predicted by XenoSite.

Entry	E_p.a._ [mV]^[a]^	MQS	Entry	E_p.a._ [mV]^[a]^	MQS
Flu	306	0.91	35 e	–^[b]^	0.91
Ret	260	0.94	35 f	850	0.51
7	628	0.93	42 a	668	0.28
21	553	0.93	43 a	678	0.42
27	648	0.87	43 b	663	0.45
35 a	633	0.50	43 c	678	0.34
35 b	618	0.57	48	–^[b]^	0.33
35 c	643	0.73	53	–^[b]^	0.55
35 d	663	0.56

[a] The cyclic voltammetry measurements were carried out in a 0.1 M Tris‐HCl buffer (pH 7.4) in a potential range from −0.5 to 1.0 V with 5 mV voltage steps and a sweep rate of 0.1 V/s. [b] No oxidation was observed in the specified potential range.

A very early hypothesis of ours, according to which low oxidation potentials correlate with good K_V_7.2/3 opening activity and vice versa, could not be confirmed since all N‐1/3 dicarba analogues showed improved oxidation stability, and at the same time, some derivatives were significantly more potent than flupirtine.^[53]^

A reduced tendency towards oxidation, indicated by higher anodic peak potentials in cyclic voltammetry, does not inevitably exclude the formation of quinoid metabolites, as it does not allow any conclusions to be drawn about the type of oxidation products formed. Therefore, the propensity of the carba analogues to form quinoid metabolites was investigated in more detail. Considering the N‐1/3 dicarba scaffold of analogues **27**–**53**, oxidation to quinone diimines or azaquinone diimines is impossible due to the lack of ortho or para amino substituents. As a result, no phenazinium‐like dimers can be formed, which are suspected of being responsible for the blue tissue discolouration in the case of retigabine. However, the formation of different quinoid metabolites such as quinone imine methides would still be theoretically conceivable, which, although they cannot form coloured phenazinium structures, can possibly trigger hepatotoxic reactions similar to the azaquinone diimine oxidation products of flupirtine. To evaluate the possibility of quinoid metabolite formation in general, XenoSite was used, which is an *in silico* method based on a deep learning approach that allows for predicting the formation of different quinone species in drug metabolism.^[54]^ The predicted molecule quinone formation scores (MQS) are included in Table 2, whereby the developer of XenoSite defined a value of 0.52 as the cut‐off above a molecule is likely to form quinoid metabolites.

As can be seen, flupirtine and retigabine have high molecule quinone formation scores of 0.91 and 0.94, respectively. Together with the low oxidation potentials determined by cyclic voltammetry, these results support the initial hypothesis that quinoid metabolites may be a factor in the adverse drug reactions of flupirtine and retigabine. While cyclic voltammetry revealed a reduction in oxidation sensitivity to comparable levels for all carba analogues except **21** and **35 f**, *in silico* risk prediction for quinoid metabolite formation yielded more differentiated results. The N‐1 monocarba analogues **7** and **21** demonstrated MQS values above 0.9 and were thus in the range of flupirtine and retigabine. Therefore, they are also likely to form quinone diimine metabolites despite an overall reduced sensitivity to oxidation indicated by higher anodic peak potentials. In contrast, all N‐1/3 dicarba analogues have significantly reduced MQS values except compound **35 e**, whereby a visual inspection of the graphical output of XenoSite showed that the high MQS value of **35 e** is not associated with the central phenyl ring. Surprisingly, XenoSite predicted oxidation of the 2,4‐difluoroaniline ring of **35 e**, which was actually assumed to be metabolically stable. However, the literature shows that in some cases, fluorinated aromatic compounds can also be metabolized to quinoid structures by oxidative ipso substitution.^[55]^ The data of the other N‐1/3 dicarba analogues indicate that the central aromatic ring itself has a crucial impact on the risk for quinoid metabolite formation. A central pyridine ring, as in flupirtine derivative **27**, is still associated with a relatively high risk (MQS 0.87), whereas a phenyl ring significantly reduces the molecule quinone formation score to 0.51–0.73. An inverted pyridine ring seems to be the best choice since the MQS values of the concerning compounds **42 a**–**43 c** are, without exception, well below the cut‐off of 0.52. Despite the slightly less favourable central phenyl ring, the MQS of the most active compound **35 a** is also below the cut‐off value. In conclusion, the formation of quinoid metabolites is improbable in the latter cases, including the submicromolar active compounds **35 a** and **43 b**.

## Conclusion

With the aim of developing safer replacements for flupirtine and retigabine, the oxidation‐sensitive triaminoaryl metabophore was successfully transformed into an oxidation‐stable carba scaffold by replacing amino substituents with alkyl groups while maintaining good K_V_7.2/3 opening activity. Particularly, the N‐1/3 dicarba analogues **35 a** and **43 b** exhibited submicromolar activity and no critical toxicity *in vitro*. In addition, they showed improved oxidation stability and *in silico* predictions indicate a negligible risk for the formation of quinoid metabolites. Consequently, **35 a** and **43 b** represent excellent candidates for further development and characterization. This includes an *in vitro* metabolism study to confirm the absence of reactive metabolites and an investigation of channel selectivity, both of which are subject to future research.

## Experimental Section

### Chemistry

Unless otherwise stated, all solvents and reagents were purchased from commercial suppliers (Sigma Aldrich, VWR, TCI and ABCR) and used without further purification. All anhydrous solvents were obtained from Acros Organics, except THF, which was dried by refluxing over sodium and benzophenone (until a permanent dark blue colouration was evident), followed by distillation under anhydrous conditions. Microwave‐assisted syntheses were conducted using an Anton Paar Monowave 300 reactor operated in closed vessel mode. An integrated IR sensor was used for temperature control, and the stirring speed was set to 600 rpm. A Bruker Avance III device was used to record ^1^H‐NMR (400 MHz) and ^13^C‐NMR (100 MHz) spectra. The chemical shifts were measured using the internal standard tetramethylsilane (TMS) in deuterated solvents and reported in parts per million (ppm). The coupling constants (*J*) are reported in Hz, and the splitting patterns are abbreviated as follows: br=broad, s=singlet, d=doublet, t=triplet, q=quartet, m=multiplet, and combinations thereof. MIR spectra were recorded with an ALPHA FT‐IR instrument from Bruker Optics coupled with a diamond ATR accessory unit. A Bruker Elute UHPLC with Bruker compact QTOF‐MS, a Bruker maXis LC‐QTOF‐MS or a Shimadzu LCMS‐IT‐TOF system, each operated with ESI ionization, were used to obtain the HRAM‐MS data. The melting points were measured with an automated Büchi Melting Point M‐565 apparatus. HPLC analysis (at 220 nm, using the 100 % method) determined the purity of all final compounds to be >95 %. Preparative and analytical HPLC procedures were conducted using Shimadzu devices CBM‐20 A, LC‐20AP, SIL‐20 A, FRC‐10 A with SPD 20 A UV/Vis detector. For analytical separation, a LiChroCART (250×4 mm) and for preparative scale separation, a Hibar RT (250×25 mm) HPLC column was used, both containing LiChrospher 100 RP‐18e (5 μm). A mobile phase consisting of methanol/water (75 : 25) with 0.1 % formic acid was used unless stated otherwise. Thin‐layer chromatography (TLC) was performed on silica gel 60 F_254_ aluminium plates obtained from Merck. Column chromatography was carried out using a glass column and silica gel 60 from Carl Roth with a particle size of 20–45 μm. Purification by flash chromatography was conducted using 25 g or 50 g Biotage SNAP KP‐SIL columns, which were run with the Sepacore system from Büchi. Alternatively, an Interchim puriFlash XS 520Plus system in combination with 80 g puriFlash 30SI‐HP or 25 g puriFlash 15SI‐HP columns was used.

**General procedure** (**Sonogashira reaction**): Pd(PPh_3_)_4_ (0.05 equiv.) and CuI (0.05 equiv.) were set under an argon atmosphere in a three‐necked round bottom flask. Aryl halide (2.0–3.6 mmol, 0.1 mmol/mL) and alkyne (1.5–2.7 equiv., see individual procedures) were dissolved in a mixture of triethylamine and DMF (1 : 2.5, v/v). The resulting solution was degassed using the freeze pump thaw method and added to the mixture of Pd(PPh_3_)_4_ and CuI. The resulting solution was stirred at 60 °C. After complete conversion (0.5–6 h), the reaction was quenched by the addition of water (100 mL), and the mixture was extracted with DCM (2×100 mL). The combined organic phases were extracted with water (3×100 mL), dried over Na_2_SO_4_, filtrated, and concentrated under reduced pressure to obtain the crude product.

**General procedure** (**Rosenmund**‐**von Braun reaction**): The aryl bromide reactant (32.0–62.0 mmol, 0.8 mmol/mL) and CuCN (2.0 equiv.) were dissolved in NMP. The reaction mixture was stirred at 170 °C. After complete conversion (7–8 h), it was allowed to cool to room temperature. Ethyl acetate (600 mL) was added, and the resulting suspension was filtered through a pad of celite. The filtrate was extracted with an aq. 3.5 % NH_3_ solution (2×300 mL), washed with brine, dried over Na_2_SO_4_, filtrated, and concentrated under reduced pressure to obtain the crude product.

**General procedure (nitrile reduction**): The nitrile reactant (2.6–10.0 mmol, 0.1 mmol/mL) was dissolved in 98 % formic acid, a Raney nickel suspension in water (50 %, 0.8 mL per mmol nitrile reactant) was added, and the mixture was stirred at 80 °C. After complete conversion (6–8 h), it was filtered through a pad of celite, which was subsequently rinsed with ethyl acetate (100 mL). The combined filtrates were concentrated under reduced pressure. The resulting residue was suspended in ethyl acetate (50–190 mL) and filtrated. The filtrate was extracted with an equal volume of a saturated aq. NaHCO_3_ solution. Thereafter, the organic phase was washed with brine, dried over Na_2_SO_4_, filtrated, and concentrated under reduced pressure to obtain the crude product.

**General procedure (reductive amination**): The aldehyde reactant (1.1–4.8 mmol, 0.1 mmol/mL) was suspended in dry toluene. 4 Å molecular sieves (1.0 g/mL) and the amine reactant (1.1–1.2 equiv., see individual procedures) were added. The resulting reaction mixture was stirred at 120 °C. After complete conversion (4–8 h), it was filtered hot to remove the molecular sieves. The filtrate was cooled to room temperature and concentrated under reduced pressure. The residue was dissolved in a mixture of 1,4‐dioxane and methanol (4 : 1, v/v) to a concentration of 0.1 mmol/mL. The mixture was cooled to 0 °C, and sodium borohydride (5.0 equiv.) was added in portions over a period of 1 h under stirring. Afterwards, the mixture was allowed to warm to room temperature and stirred for 16 h. The reaction was quenched by adding 50–220 mL of water, and the mixture was extracted with an equal volume of ethyl acetate. The organic phase was washed with brine, dried over Na_2_SO_4_, filtrated, and concentrated under reduced pressure to obtain the crude product.

**General procedure (Suzuki reaction**): In a microwave vessel, the aryl bromide reactant (1.2–1.3 mmol, 0.3 mmol/mL) was dissolved in 1,4‐dioxane. A 2 M aq. solution of Na_2_CO_3_ (2.8 equiv.), Pd(PPh_3_)_4_ (0.1 equiv.), and boronic acid or boronic acid anhydride (2.0 equiv.) were added. Argon was passed through the reaction mixture for 30 min. Afterwards, the mixture was heated in a microwave reactor at 140 °C in a closed vessel under an atmosphere of argon. After 75 min, water (100 mL) was added, and the aq. mixture was extracted with ethyl acetate (100 mL). The organic phase was washed with brine, dried over Na_2_SO_4_, filtrated, and concentrated under reduced pressure to obtain the crude product.

**Ethyl** [**2**‐**amino**‐**6**‐(**4**‐**fluorophenethyl**)**pyridin**‐**3**‐**yl**]**carbamate** (**7**): Compound **15** (411 mg, 1.60 mmol) and Pd/C (10 % Pd, 50 % water wet, 200 mg) were suspended in ethyl acetate (40 mL). The suspension was carefully set under a hydrogen atmosphere (balloon pressure) and stirred at 40 °C. After 16 h, another 200 mg of Pd/C were added. Afterwards, stirring was continued for 24 h at 40 °C under a hydrogen atmosphere. After completion of the reaction, the catalyst was filtered off, and the filtrate was concentrated under reduced pressure. The resulting residue was dissolved in 20 mL of THF, and triethylamine (445 μL, 3.20 mmol, 2.0 equiv.) was added. The reaction was set under an argon atmosphere, and a solution of ethyl chloroformate (304 μL, 3.20 mmol, 2.0 equiv.) in 2 mL of THF was added dropwise over 30 min. After stirring at room temperature for an additional 4 h, the reaction mixture was partitioned between 100 mL of ethyl acetate and 100 mL of water. The organic phase was extracted with water (2×100 mL), washed with brine, dried over Na_2_SO_4_, filtrated, and concentrated under reduced pressure. The crude residue was purified by silica gel column chromatography (ethyl acetate/*n*‐hexane 3 : 2) and subsequent recrystallization (methanol/water) to obtain **7** as a colourless solid (120 mg, 0.40 mmol, 25 %): *R*_f_=0.23 (ethyl acetate/*n*‐hexane 1 : 1); mp: 154 °C; ^1^H‐NMR (400 MHz, DMSO‐d_6_): δ(ppm)=8.57 (s, 1H), 7.44 (d, *J*=6.6 Hz, 1H), 7.28–7.18 (m, 2H), 7.12–7.02 (m, 2H), 6.41 (d, *J*=7.8 Hz, 1H), 5.70 (s, 2H), 4.10 (q, *J*=7.1 Hz, 2H), 2.94–2.85 (m, 2H), 2.80–2.71 (m, 2H),1.23 (t, *J*=7.1 Hz, 3H); ^13^C‐NMR (100 MHz, DMSO‐d_6_): δ(ppm)=160.6 (d, *J*=241.0 Hz), 154.5, 152.3, 137.8 (d, *J*=3.0 Hz), 131.0, 130.0, 130.0 (d, *J*=7.9 Hz), 116.4, 114.8 (d, *J*=20.9 Hz), 110.9, 60.3, 38.7, 34.2, 14.5; IR (ATR): $\tilde{\nu }$ =3417, 3329 (m, ν_N−H_), 3036 (w, ν_C−H_), 1686 (s, ν_C=O_) 1652 (m, δ_N−H_); ESI‐HRMS (*m/z*): calcd. for [C_16_H_18_N_3_O_2_F+H]^+^ 304.1456, found 304.1447; cpd purity (220 nm): 100 %.

[(**4**‐**Fluorophenyl**)**ethynyl**]**trimethylsilane** (**10**): Pd(PPh_3_)_4_ (347 mg, 0.30 mmol, 0.05 equiv.) and CuI (57 mg, 0.30 mmol, 0.05 equiv.) were set under an argon atmosphere in a three‐necked round bottom flask. 1‐Bromo‐4‐fluorobenzene (660 μL, 6.00 mmol) and ethynyl(trimethyl)silane (1020 μL, 7.20 mmol, 1.2 equiv.) were dissolved in 18 mL of triethylamine. The resulting solution was degassed using the freeze pump thaw method and added to the mixture of Pd(PPh_3_)_4_ and CuI. The resulting solution was stirred at 60 °C for 16 h under an argon atmosphere. Subsequently, the reaction was quenched by adding water (100 mL), and the mixture was extracted with *n*‐hexane (100 mL). The organic phase was washed with brine, dried over Na_2_SO_4_, filtrated, and concentrated under reduced pressure. The crude residue was purified by silica gel column chromatography with *n*‐hexane as mobile phase, which yielded **10** as a colourless liquid (1.14 g, 6.0 mmol, 99 %): *R*_f_=0.83 (*n*‐hexane); ^1^H‐NMR (400 MHz, DMSO‐d_6_): δ(ppm)=7.56–7.47 (m, 2H), 7.27–7.16 (m, 2H), 0.23 (s, 9H); ^13^C‐NMR (100 MHz, DMSO‐d_6_): δ(ppm)=162.1 (d, *J*=248.1 Hz), 133.9 (d, *J*=8.6 Hz), 118.6 (d, *J*=3.4 Hz), 115.9 (d, *J*=22.1 Hz), 104.0, 93.8, −0.2; IR (ATR): $\tilde{\nu }$ =2161 (w, ν_C≡C_).

**1**‐**Ethynyl**‐**4**‐**fluorobenzene** (**11**): Compound **10** (1.22 g, 6.3 mmol) and K_2_CO_3_ (1.76 g, 12.7 mmol, 2.0 equiv.) were dissolved in 19 mL of methanol. The reaction mixture was set under an argon atmosphere and stirred at room temperature. After 5 h, 50 mL of water were added, and the pH of the solution was adjusted to 4–5 by the addition of a 1 M aq. HCl solution. Afterwards, the resulting solution was extracted with diethyl ether (3×50 mL). The combined organic phases were washed with brine, dried over Na_2_SO_4_, filtrated, and concentrated under reduced pressure. The resulting orange liquid (645 mg, 5.37 mmol, 85 %) was used for the following reaction without characterization or further purification.

**6**‐**Chloro**‐**3**‐**nitropyridin**‐**2**‐**amine** (**13**): 2,6‐Dichloro‐3‐nitropyridine (19.30 g, 100.0 mmol) was dissolved in ethanol (200 mL). The reaction mixture was cooled to 0 °C, and ammonia gas was passed through the solution under stirring for 3 h. Afterwards, the reaction mixture was sealed and continued to stir at room temperature. After 18 h, the solution was poured into water (400 mL). The resulting precipitate was collected by filtration and washed with additional water (200 mL) and *n*‐hexane (200 mL). The title compound was obtained as a yellow solid (14.41 g, 83.0 mmol, 83 %): *R*_f_=0.86 (ethyl acetate/*n*‐hexane 2 : 3); mp: 190 °C; ^1^H‐NMR (400 MHz, DMSO‐d_6_): δ(ppm)=8.39 (d, *J*=8.6 Hz, 1H), 8.25 (s, 2H), 6.77 (d, *J*=8.7 Hz, 1H); ^13^C‐NMR (100 MHz, DMSO‐d_6_): δ(ppm)=155.0, 153.4, 138.3, 126.1, 112.0; MIR (ATR): $\tilde{\nu }$ =3442 (w, ν_N−H_), 3157 (w, ν_C−H_), 1552 (m, δ_N−H_), 1552 (m, ν_N−O_).

**6**‐**Bromo**‐**3**‐**nitropyridin**‐**2**‐**amine** (**14**): Compound **13** (750 mg, 4.32 mmol) was suspended in 10 mL of an HBr solution in acetic acid (33 %). The resulting reaction mixture was stirred for 8 h at 100 °C. The reaction was cooled to 0 °C and adjusted to pH 7 with an aq. NaOH solution (10 %). The resulting precipitate was filtered off and washed with water to obtain the title compound as a yellow solid (878 mg, 4.03 mmol, 93 %): *R*_f_=0.70 (ethyl acetate/*n*‐hexane 1 : 4); mp: 242 °C; ^1^H‐NMR (400 MHz, DMSO‐d_6_): δ(ppm)=8.26 (d, *J*=8.6 Hz, 1H), 8.26 (s, 2H), 6.90 (d, *J*=8.5 Hz, 1H); ^13^C‐NMR (100 MHz, DMSO‐d_6_): δ(ppm)=153.4, 146.9, 137.7, 126.4, 115.8; IR (ATR): $\tilde{\nu }$ =3444 (w, ν_N−H_), 3147 (w, ν_C−H_), 1629 (m, δ_N−H_), 1552 (m, ν_N−O_).

**6**‐[(**4**‐**Fluorophenyl**)**ethynyl**]‐**3**‐**nitropyridin**‐**2**‐**amine** (**15**): The synthesis was conducted from **14** (436 mg, 2.00 mmol) and **11** (645 mg, 5.37 mmol, 2.7 equiv.) according to the general procedure (Sonogashira reaction). The crude residue was further purified by silica gel column chromatography (ethyl acetate/*n*‐hexane 1 : 4) and subsequent recrystallization (ethyl acetate) to obtain **15** as a gold‐coloured solid (411 mg, 1.60 mmol, 80 %): *R*_f_=0.66 (ethyl acetate/*n*‐hexane 1 : 3); mp: 224 °C; ^1^H‐NMR (400 MHz, DMSO‐d_6_): δ(ppm)=8.42 (d, *J*=8.5 Hz, 1H), 8.03 (s, 2H), 7.75–7.67 (m, 2H), 7.38–7.29 (m, 2H), 6.96 (d, *J*=8.5 Hz, 1H); ^13^C‐NMR (100 MHz, DMSO‐d_6_): δ(ppm)=162.9 (d, *J*=250.0 Hz), 153.5, 147.6, 135.8, 134.6 (d, *J*=8.9 Hz), 126.3, 117.1 (d, *J*=3.3 Hz), 116.3 (d, *J*=22.3 Hz), 115.7, 90.7, 87.9; IR (ATR): $\tilde{\nu }$ =3469 (w, ν_N−H_), 3097 (w, ν_C−H_), 2217 (w, ν_C≡C_), 1613 (m, δ_N−H_), 1598 (m, ν_N−O_).

**3**‐**Nitro**‐**6**‐(**phenylethynyl**)**pyridin**‐**2**‐**amine** (**16**): The synthesis was carried out from **13** (521 mg, 3.00 mmol) and ethynylbenzene (659 μL, 6.00 mmol, 2.0 equiv.) according to the general procedure (Sonogashira reaction). The crude residue was further purified by silica gel column chromatography (ethyl acetate/*n*‐hexane 3 : 7) and subsequent recrystallization (ethyl acetate) to obtained **16** as an orange solid (274 mg, 1.15 mmol, 38 %): *R*_f_=0.70 (ethyl acetate/*n*‐hexane 1 : 3); mp: decomp.; ^1^H‐NMR (400 MHz, DMSO‐d_6_): δ(ppm)=8.42 (d, *J*=8.5 Hz, 1H), 8.04 (s, 2H), 7.67–7.60 (m, 2H), 7.53–7.46 (m, 3H,), 6.97 (d, *J*=8.5 Hz, 1H); ^13^C‐NMR (100 MHz, DMSO‐d_6_): δ(ppm)=153.7, 147.7, 135.7, 132.0, 130.2, 129.0, 126.3, 120.6, 115.7, 91.7, 88.1; IR (ATR): $\tilde{\nu }$ =3467 (w, ν_N−H_), 3099 (w, ν_C−H_), 2209 (w, ν_C≡C_), 1626 (m, δ_N−H_), 1565 (m, ν_N−O_).

**3**‐**Nitro**‐**6**‐[(**trimethylsilyl**)**ethynyl**]**pyridin**‐**2**‐**amine** (**17**): The synthesis was conducted from **14** (630 mg, 3.63 mmol) and ethynyl(trimethyl)silane (775 μL, 5.45 mmol, 1.5 equiv.) according to the general procedure (Sonogashira reaction). The purification was carried out by silica gel column chromatography (ethyl acetate/*n*‐hexane 1 : 9) and subsequent recrystallization (ethyl acetate) to obtain **17** as a gold‐coloured solid (634 mg, 2.69 mmol, 74 %): *R*_f_=0.62 (ethyl acetate/*n*‐hexane 1 : 9); mp: 164 °C; ^1^H‐NMR (400 MHz, DMSO‐d_6_): δ(ppm)=8.36 (d, *J*=8.5 Hz, 1H), 7.99 (s, 2H), 6.83 (d, *J*=8.5 Hz, 1H), 0.25 (s, 9H); ^13^C‐NMR (100 MHz, DMSO‐d_6_): δ(ppm)=153.4, 147.0, 135.8, 126.5, 115.5, 103.1, 97.9, −0.61; IR (ATR): $\tilde{\nu }$ =3472 (m, ν_N−H_), 3133, 2962 (w, ν_C−H_), 1617 (m, δ_N−H_), 1563 (m, ν_N−O_).

**6**‐**Ethynyl**‐**3**‐**nitropyridin**‐**2**‐**amine** (**18**): Compound **17** (574 mg, 2.44 mmol) and K_2_CO_3_ (674 mg, 4.88 mmol, 2.0 equiv.) were dissolved in 20 mL of methanol. The reaction was stirred at room temperature. After 30 min, water (50 mL) was added. The pH of the resulting solution was adjusted to 7 by the addition of a 1 M aq. HCl solution. Subsequently, the neutralized solution was extracted with ethyl acetate (3×50 mL). The combined organic phases were washed with brine, dried over Na_2_SO_4_, filtrated, and concentrated to dryness under reduced pressure. The title compound was obtained as a brown solid (299 mg, 1.83 mmol, 75 %): *R*_f_=0.54 (ethyl acetate/*n*‐hexane 1 : 3); mp: decomp.; ^1^H‐NMR (400 MHz, DMSO‐d_6_): δ(ppm)=8.38 (d, *J*=8.5 Hz, 1H), 7.99 (s, 2H), 6.87 (d, *J*=8.5 Hz, 1H), 4.63 (s, 1H); ^13^C‐NMR (100 MHz, DMSO‐d_6_): δ(ppm)=153.4, 147.0, 135.8, 126.7, 115.7, 83.5, 82.0; IR (ATR): $\tilde{\nu }$ =3464 (w, ν_N−H_), 3087 (w, ν_C−H_), 2106 (w, ν_C≡C_), 1624 (m, δ_N−H_), 1568 (m, ν_N−O_).

**Ethyl** [**2**‐**amino**‐**6**‐(**phenylethynyl**)**pyridin**‐**3**‐**yl**]**carbamate** (**21**): Compound **16** (239 mg, 1.00 mmol) and iron powder (670 mg, 12.00 mmol, 12.0 equiv.) were suspended in acetic acid (20 mL). The reaction mixture was set under an argon atmosphere and stirred for 1 h at 50 °C. Subsequently, the reaction was cooled to 0 °C and adjusted to pH 7 with an aq. NaOH solution (10 %). The resulting aqueous mixture was extracted with DCM (3×50 mL). The combined organic phases were dried over Na_2_SO_4_, filtrated, and concentrated under reduced pressure. The resulting residue was dissolved in 20 mL of THF, and triethylamine (333 μL, 2.40 mmol, 2.4 equiv.) was added. The reaction was again set under an argon atmosphere, and a solution of ethyl chloroformate (115 μL, 1.20 mmol, 1.2 equiv.) in 2 mL of THF was added dropwise over 30 min. After stirring at room temperature for an additional 6.5 h, the reaction mixture was partitioned between 100 mL of ethyl acetate and 100 mL of water. The organic phase was extracted with water (2×100 mL), washed with brine, dried over Na_2_SO_4_, filtrated, and concentrated under reduced pressure. The crude residue was purified by silica gel column chromatography (ethyl acetate/*n*‐hexane 1 : 1) to obtain **21** as an off‐white solid (85 mg, 0.30 mmol, 30 %): *R*_f_=0.45 (ethyl acetate/*n*‐hexane 1 : 1); mp: 152 °C; ^1^H‐NMR (400 MHz, DMSO‐d_6_): δ(ppm)=8.83 (s, 1H), 7.75 (d, *J*=7.9 Hz, 1H), 7.58–7.49 (m, 2H), 7.48–7.38 (m, 3H), 6.86 (d, *J*=7.9 Hz, 1H), 6.10 (s, 2H), 4.14 (q, *J*=7.1 Hz, 2H), 1.26 (t, *J*=7.1 Hz, 3H); ^13^C‐NMR (100 MHz, DMSO‐d_6_): δ(ppm)=154.1, 151.9, 134.6, 131.4, 128.9, 128.8, 128.5, 122.0, 119.5, 116.4, 89.7, 86.2, 60.6, 14.5; IR (ATR): $\tilde{\nu }$ =3457, 3281 (m, ν_N−H_), 3142, 2978 (w, ν_C−H_), 1690 (s, ν_C=O_), 1620 (m, δ_N−H_); ESI‐HRMS (*m/z*): calcd. for [C_16_H_15_N_3_O_2_+H]^+^ 282.1237, found 282.1235; cpd purity (220 nm): 100 %.

**6**‐**Methyl**‐**5**‐**nitropicolinonitrile** (**23**): 6‐Bromo‐2‐methyl‐3‐nitropyridine (2.82 g, 13.0 mmol), Pd(PPh_3_)_4_ (451 mg, 0.39 mmol, 0.03 equiv.), and zinc cyanide (2.14 g, 18.2 mmol, 1.4 equiv.) were dissolved in DMF (20 mL). The reaction mixture was set under an argon atmosphere and stirred at 70 °C. After 24 h, it was cooled to room temperature and quenched by adding water (100 mL). The resulting aq. suspension was extracted with ethyl acetate (100 mL). The organic phase was extracted with water (2×100 mL), washed with brine, dried over Na_2_SO_4_, filtrated, and concentrated under reduced pressure. The crude residue was purified by silica gel column chromatography (ethyl acetate/*n*‐hexane 1 : 4) to obtain **23** as a yellow oil (2.03 g, 12.4 mmol, 96 %): *R*_f_=0.74 (ethyl acetate/*n*‐hexane 1 : 3); ^1^H‐NMR (400 MHz, DMSO‐d_6_): δ(ppm)=8.66 (d, *J*=8.3 Hz, 1H), 8.22 (dd, *J*=8.3, 0.7 Hz, 1H), 2.77 (s, 3H); ^13^C‐NMR (100 MHz, DMSO‐d_6_): δ(ppm)=154.4, 147.3, 134.6, 134.6, 127.9, 116.2, 22.8; IR (ATR): $\tilde{\nu }$ =3085, 2991 (w, ν_C−H_), 1593 (m, ν_N−O_).

**5**‐**Amino**‐**6**‐**methylpicolinonitrile** (**24**): Compound **23** (163 mg, 1.00 mmol), iron powder (670 mg, 12.00 mmol, 12.0 equiv.), and calcium chloride (1.33 g, 12.0 mmol, 12.0 equiv.) were suspended in acetic acid (1 mL) and ethanol (6 mL). The reaction mixture was stirred for 2 h at room temperature. Afterwards, the suspension was filtered through a pad of celite, which was subsequently rinsed with ethanol. The combined filtrates were concentrated under reduced pressure. The crude residue was purified by silica gel column chromatography (ethyl acetate/*n*‐hexane 1 : 1) to obtain **24** as a yellow solid (95 mg, 0.71 mmol, 71 %): *R*_f_=0.34 (ethyl acetate/*n*‐hexane 1 : 1); mp: 197 °C; ^1^H‐NMR (400 MHz, DMSO‐d_6_): δ(ppm)=7.48 (d, *J*=8.3 Hz, 1H), 6.92 (d, *J*=8.3 Hz, 1H), 6.14 (s, 2H), 2.28 (s, 3H); ^13^C‐NMR (100 MHz, DMSO‐d_6_): δ(ppm)=146.1, 144.5, 128.1, 119.2, 117.8, 116.7, 20.4; IR (ATR): $\tilde{\nu }$ =3445 (m, ν_N−H_), 3191, 2941 (w, ν_C−H_), 2215 (m, ν_C≡N_), 1646 (m, δ_N−H_).

**Isobutyl** (**6**‐**cyano**‐**2**‐**methylpyridin**‐**3**‐**yl**)**carbamate** (**25**): Compound **24** (1.94 g, 14.6 mmol) and *N,N*‐dimethylpyridin‐4‐amine (89 mg, 0.73 mmol, 0.05 equiv.) were dissolved in DCM (100 mL). Triethylamine (4.08 mL, 29.2 mmol, 2.0 equiv.) and isobutyl chloroformate (3.81 mL, 29.2 mmol, 2.0 equiv.) were added successively, and the reaction was stirred at room temperature. After 24, 48, and 72 h, an additional equivalent isobutyl chloroformate (1.90 mL, 14.6 mmol) was added. After 96 h in total, the reaction mixture was concentrated under reduced pressure, and the crude residue was purified by silica gel column chromatography (ethyl acetate/*n*‐hexane 1 : 1) to obtain **25** as a colourless solid (1.17 g, 5.0 mmol, 34 %): *R*_f_=0.54 (ethyl acetate/*n*‐hexane 1 : 3); mp: 106 °C; ^1^H‐NMR (400 MHz, DMSO‐d_6_): δ(ppm)=9.44 (s, 1H), 8.16 (d, *J*=8.4 Hz, 1H), 7.85 (d, *J*=8.4 Hz, 1H), 3.93 (d, *J*=6.7 Hz, 2H), 2.50 (s, 3H), 2.05–1.86 (m, 1H), 0.94 (d, *J*=6.7 Hz, 6H); ^13^C‐NMR (100 MHz, DMSO‐d_6_): δ(ppm)=154.0, 152.1, 136.9, 129.1, 127.2, 125.8, 117.7, 70.9, 27.5, 21.1, 18.8; IR (ATR): $\tilde{\nu }$ =3336 (m, ν_N−H_), 3053 (w, ν_C−H_), 2235 (w, ν_C≡N_), 1729 (s, ν_C=O_).

**Isobutyl** (**6**‐**formyl**‐**2**‐**methylpyridin**‐**3**‐**yl**)**carbamate** (**26**): Compound **25** (300 mg, 1.29 mmol) was dissolved in dry DCM (20 mL). The resulting solution was set under an argon atmosphere and cooled to −85 °C. 3.86 mL of a 1 M solution of DIBAL in *n*‐hexane (3.9 mmol, 3.0 equiv.) were dissolved in dry DCM (20 mL) and added dropwise to the reaction mixture over a period of 1 h while maintaining the temperature at −85 °C. Afterwards, the reaction was continued to stir at −85 °C. After 2 h, an additional 1.29 mL of the 1 M DIBAL solution in *n*‐hexane (1.3 mmol, 1.0 equiv.) was added dropwise. The reaction was stirred for 1 h at −85 °C and then quenched by adding a 1 M aq. HCl solution (20 mL). Subsequently, the mixture was allowed to warm to room temperature and stirred vigorously for 1 h. Afterwards, the phases were separated, and the aq. phase was extracted with DCM (2×50 mL). The combined organic phases were dried over Na_2_SO_4_, filtrated, and concentrated under reduced pressure. The crude residue was purified by silica gel column chromatography (ethyl acetate/*n*‐hexane 1 : 1) to obtain **26** as a slightly yellow solid (70 mg, 0.30 mmol, 23 %): *R*_f_=0.73 (ethyl acetate/*n*‐hexane 1 : 1); mp: 102 °C; ^1^H‐NMR (400 MHz, DMSO‐d_6_): δ(ppm)=9.87 (d, *J*=0.8 Hz, 1H), 9.39 (s, 1H), 8.17 (d, *J*=8.3 Hz, 1H), 7.80 (d, *J*=8.4 Hz, 1H), 3.93 (d, *J*=6.7 Hz, 2H), 2.56 (s, 3H), 2.03–1.89 (m, 1H), 0.95 (d, *J*=6.7 Hz, 6H); ^13^C‐NMR (100 MHz, DMSO‐d_6_): δ(ppm)=192.6, 154.1, 150.5, 147.0, 137.3, 129.3, 120.2, 70.8, 27.5, 21.1, 18.9; IR (ATR): $\tilde{\nu }$ =3298 (w, ν_N−H_), 2958 (w, ν_C−H_), 1733 (s, ν_C=O_), 1682 (m, δ_N−H_).

**Isobutyl** (**6**‐**{**[(**4**‐**fluorophenyl**)**amino**]**methyl}**‐**2**‐**methylpyridin**‐**3**‐**yl**)**carbamate** (**27**): Compound **26** (65 mg, 0.28 mmol) and 4‐fluoroaniline (29 μL, 0.30 mmol, 1.1 equiv.) were dissolved in dry DCM (15 mL). After stirring at room temperature for 5 h, the reaction mixture was concentrated under reduced pressure. The residue was dissolved in methanol (20 mL), and sodium borohydride (47 mg, 1.38 mmol, 5.0 equiv.) was added in portions over 30 min at room temperature. The reaction kept stirring at room temperature for another 30 min. Thereafter, water (20 mL) was added, and subsequently, methanol was removed under reduced pressure. A brown precipitate formed, which was filtered off and washed with water. The crude product was recrystallized from methanol/water to obtain **27** as an off‐white solid (75 mg, 0.23 mmol, 82 %): *R*_f_=0.41 (ethyl acetate/*n*‐hexane 1 : 1); mp: 123 °C; ^1^H‐NMR (400 MHz, DMSO‐d_6_): δ(ppm)=8.99 (s, 1H), 7.63 (d, *J*=8.2 Hz, 1H), 7.14 (d, *J*=8.2 Hz, 1H), 6.93–6.82 (m, 2H), 6.57–6.48 (m, 2H), 6.22 (t, *J*=6.1 Hz, 1H), 4.24 (d, *J*=6.1 Hz, 2H), 3.85 (d, *J*=6.7 Hz, 2H), 2.41 (s, 3H), 1.98–1.83 (m, 1H), 0.91 (d, *J*=6.7 Hz, 6H); ^13^C‐NMR (100 MHz, DMSO‐d_6_): δ(ppm)=155.0, 154.6, 154.3 (d, *J*=229 Hz), 151.4, 145.1, 132.5, 130.9, 118.5, 115.2 (d, *J*=21.9 Hz), 112.9 (d, *J*=7.3 Hz), 70.3, 48.6, 27.6, 20.9, 18.9; IR (ATR): $\tilde{\nu }$ =3380, 3280 (w, ν_N−H_), 2928 (w, ν_C−H_), 1688 (s, ν_C=O_). ESI‐HRMS (*m/z*): calcd. for [C_18_H_22_N_3_O_2_F+H]^+^ 332.1769, found 332.1754; cpd purity (220 nm): 100 %.

**4**‐**Nitro**‐**3**‐(**trifluoromethyl**)**benzonitrile** (**29**): Pd(OAc)_2_ (45 mg, 0.20 mmol, 0.2 equiv.) and triphenylphoshine (polymer bound, 3 mmol/g, 150 mg, 0.45 mmol, 0.5 equiv) were suspended in DMF (3 mL) and set under an argon atmosphere. The mixture was stirred at room temperature. After 1.5 h, Zn(CN)_2_ (235 mg, 2.00 mmol, 2.0 equiv.) and a solution of 4‐bromo‐1‐nitro‐2‐(trifluormethyl)benzene (270 mg, 1.00 mmol) in DMF (2 mL) were added. Thereafter, the reaction mixture was stirred at 100 °C under an atmosphere of argon. After 1.5 h, the suspension was cooled to room temperature, poured into ethyl acetate (100 mL), and filtrated. The filtrate was extracted with water (3×100 mL), washed with brine, dried over Na_2_SO_4_, filtrated again, and concentrated under reduced pressure. The crude residue was purified by flash chromatography (mobile phase: ethyl acetate/*n*‐hexane with 10–20 % ethyl acetate) to obtain **29** as a colourless solid (180 mg, 0.83 mmol, 83 %): *R*_f_=0.66 (ethyl acetate/*n*‐hexane 1 : 3); mp: 89 °C; ^1^H‐NMR (400 MHz, DMSO‐d_6_): δ(ppm)=8.69 (d, *J*=1.8 Hz, 1H), 8,52 (dd, *J*=8.4, 1.8 Hz, 1H), 8.38 (d, *J*=8.4 Hz, 1H); ^13^C‐NMR (100 MHz, DMSO‐d_6_): δ(ppm)=149.3, 139.1, 132.6 (q, *J*=5.1 Hz), 126.2, 122.2 (q, *J*=34.3 Hz), 121.3 (q, *J*=273.3 Hz), 116.2, 116.2; IR (ATR): $\tilde{\nu }$ =3057, 2921 (w, ν_C−H_), 2239 (w, ν_C≡N_), 1539 (s, ν_N−O_).

**4**‐**Bromo**‐**2,6**‐**dimethylaniline** (**31 a**): 2,6‐Dimethylaniline (7.87 g, 65.0 mmol) was dissolved in DCM (200 mL), and the resulting solution was cooled to −78 °C. A solution of bromine (3.49 mL, 69.2 mmol, 1.1 equiv.) in DCM (20 mL) was slowly added to the mixture over a period of 30 min while maintaining the temperature at −78 °C. Subsequently, the cooling was removed. After the solution was warmed to room temperature, the reaction was quenched by adding a 10 % aq. solution of NaHSO_3_ (8 mL) to the mixture. Afterwards, a saturated solution of Na_2_CO_3_ (100 mL) was added. The organic layer was separated, dried over Na_2_SO_4_, filtrated, and concentrated under reduced pressure. The crude residue was purified by silica gel column chromatography (ethyl acetate/*n*‐hexane 1 : 4) to obtain **31 a** as a dark red oil (12.46 g, 62.3 mmol, 96 %): *R*_f_=0.79 (ethyl acetate/*n*‐hexane 1 : 1); ^1^H‐NMR (400 MHz, DMSO‐d_6_): δ(ppm)=6.96 (s, 2H), 4.69 (s, 2H), 2.06 (s, 6H); ^13^C‐NMR (100 MHz, DMSO‐d_6_): δ(ppm)=143.7, 129.6, 123.0, 106.1, 17.5; IR (ATR): $\tilde{\nu }$ =3425 (w, ν_N−H_), 2974 (w, ν_C−H_), 1627 (m, δ_N−H_).

**4**‐**Amino**‐**3,5**‐**dimethylbenzonitrile** (**32 a**): The synthesis was conducted from **31 a** (12.40 g, 62.0 mmol) according to the general procedure (Rosenmund‐von Braun reaction). The purification was carried out by silica gel column chromatography (ethyl acetate/*n*‐hexane 2 : 3) to obtain **32 a** as a dark red solid (3.81 g, 26.1 mmol, 42 %): *R*_f_=0.62 (ethyl acetate/*n*‐hexane 1 : 1); mp: 116 °C; ^1^H‐NMR (400 MHz, DMSO‐d_6_): δ(ppm)=7.21 (s, 2H), 5.57 (s, 2H), 2.09 (s, 6H); ^13^C‐NMR (100 MHz, DMSO‐d_6_): δ(ppm)=149.2, 131.4, 120.8, 120.8, 95.6, 17.4; IR (ATR): $\tilde{\nu }$ =3477 (m, ν_N−H_), 2965 (w, ν_C−H_), 2206 (m, ν_C≡N_), 1631 (s, δ_N−H_).

**4**‐**Amino**‐**3**‐**methylbenzonitrile** (**32 b**): The synthesis was carried out from 4‐bromo‐2‐methylaniline (10.15 g, 54.6 mmol) in accordance with the general procedure (Rosenmund‐von Braun reaction). The crude residue was further purified by silica gel column chromatography (ethyl acetate/*n*‐hexane 2 : 3) and subsequent recrystallization (methanol/water) to obtain **32 b** as an off‐white solid (3.22 g, 24.3 mmol, 45 %): *R*_f_=0.60 (ethyl acetate/*n*‐hexane 2 : 3); mp: 94 °C; ^1^H‐NMR (400 MHz, DMSO‐d_6_): δ(ppm)=7.33–7.24 (m, 2H), 6.64 (d, *J*=8.2 Hz, 1H), 5.89 (s, 2H), 2.05 (s, 3H); ^13^C‐NMR (100 MHz, DMSO‐d_6_): δ(ppm)=151.2, 133.5, 131.1, 121.2, 120.7, 113.4, 95.8, 17.0; IR (ATR): $\tilde{\nu }$ =3400 (w, ν_N−H_), 2944 (w, ν_C−H_), 2216 (m, ν_C≡N_), 1646 (s, δ_N−H_).

**4**‐**Amino**‐**3**‐**fluorobenzonitrile** (**32 c**): The synthesis was conducted from 4‐bromo‐2‐fluoroaniline (6.00 g, 31.6 mmol) according to the general procedure (Rosenmund‐von Braun reaction). The purification was carried out by silica gel column chromatography (ethyl acetate/*n*‐hexane 3 : 7) and subsequent recrystallization (toluene/*n*‐hexane) to obtain **32 c** as an orange solid (2.02 g, 14.8 mmol, 47 %): *R*_f_=0.81 (ethyl acetate/*n*‐hexane 1 : 1); mp: 88 °C; ^1^H‐NMR (400 MHz, DMSO‐d_6_): δ(ppm)=7.48 (dd, *J*=11.7, 1.9 Hz, 1H), 7.29 (dd, *J*=8.3, 1.8 Hz, 1H), 6.78 (dd, *J*=9.0, 8.4 Hz 1H), 6.21 (s, 2H); ^13^C‐NMR (100 MHz, DMSO‐d_6_): δ(ppm)=148.9 (d, *J*=239.4 Hz), 141.8 (d, *J*=12.7 Hz), 129.9 (d, *J*=2.5 Hz), 119.4 (d, *J*=2.6 Hz), 118.5 (d, *J*=21.3 Hz), 115.6 (d, *J*=5.5 Hz), 95.4 (d, *J*=8.5 Hz); IR (ATR): $\tilde{\nu }$ =3443 (w, ν_N−H_), 2221 (m, ν_C≡N_), 1633 (s, δ_N−H_).

**4**‐**Amino**‐**3**‐(**trifluoromethyl**)**benzonitrile** (**32 d**): Compound **29** (1.20 g, 5.6 mmol) was dissolved in ethyl acetate (50 mL). SnCl_2_ (6.26 g, 27.8 mmol, 5.0 equiv.) was added, and the reaction mixture was stirred at 70 °C. After 30 min, it was cooled to room temperature and a saturated aq. NaHCO_3_ solution (100 mL) was added. The phases were separated, and the aq. phase was extracted with ethyl acetate (2×100 mL). The combined organic phases were washed with brine, dried over Na_2_SO_4_, filtrated, and concentrated under reduced pressure. The crude residue was purified by silica gel column chromatography (ethyl acetate/*n*‐hexane 3 : 2) to obtained **32 d** as a slightly yellow solid (1.02 g, 5.5 mmol, 99 %): *R*_f_=0.79 (ethyl acetate/*n*‐hexane 1 : 1); mp: 105 °C; ^1^H‐NMR (400 MHz, DMSO‐d_6_): δ(ppm)=9.50 (s, 1H), 9.25 (s, 1H), 7.90 (d, *J*=1.9 Hz, 1H), 7.85 (dd, *J*=8.7, 1.9 Hz, 1H), 7.33 (d, *J*=8.7 Hz, 1H); ^13^C‐NMR (100 MHz, DMSO‐d_6_): δ(ppm)=150.8, 137.1, 130.7 (q, *J*=5.7 Hz), 123,3 (q, *J*=270.3 Hz), 118.9, 112.8, 110.1 (q, *J*=31.7 Hz), 98.2; IR (ATR): $\tilde{\nu }$ =3318 (m, ν_N−H_), 2237 (m, ν_C≡N_), 1618 (s, δ_N−H_).

***N***‐(**4**‐**Cyano**‐**2,6**‐**dimethylphenyl**)**butyramide** (**33 a**): Compound **32 a** (1.50 g, 10.3 mmol) was dissolved in THF (25 mL). *N,N*‐Dimethylpyridin‐4‐amine (125 mg, 1.03 mmol, 0.1 equiv.) and triethylamine (2.86 mL, 20.5 mmol, 2.0 equiv.) were added successively. The reaction mixture was cooled to 0 °C, and a solution of butanoyl chloride (1.27 mL, 12.3 mmol, 1.2 equiv.) in THF (10 mL) was added dropwise under stirring over a period of 30 min. Afterwards, the reaction mixture was allowed to warm to room temperature, and stirring was continued for 16 h. After completion of the reaction, ethyl acetate (200 mL) was added, and the mixture was successively extracted with a 2 M aq. HCl solution (200 mL) and a saturated aq. NaHCO_3_ solution (200 mL). Afterwards, the organic phase was washed with brine, dried over Na_2_SO_4_, filtrated, and concentrated under reduced pressure. The crude residue was purified by silica gel column chromatography (ethyl acetate/*n*‐hexane 1 : 1) to obtain **33 a** as a colourless solid (1.63 g, 7.6 mmol, 74 %): *R*_f_=0.45 (ethyl acetate/*n*‐hexane 1 : 1); mp: 193 °C; ^1^H‐NMR (400 MHz, DMSO‐d_6_): δ(ppm)=9.47 (s, 1H), 7.59–7.54 (m, 2H), 2.33 (t, *J*=7.2 Hz, 2H), 2.17 (s, 6H), 1.71–1.57 (m, 2H), 0.95 (t, *J*=7.4 Hz, 3H); ^13^C‐NMR (100 MHz, DMSO‐d_6_): δ(ppm)=170.7, 140.3, 136.8, 131.2, 118.8, 108.7, 37.3, 18.7, 17.9, 13.7; IR (ATR): $\tilde{\nu }$ =3250 (m, ν_N−H_), 2963 (w, ν_C−H_), 2223 (m, ν_C≡N_), 1651 (s, ν_C=O_).

***N***‐(**4**‐**Cyano**‐**2**‐**methylphenyl**)**butyramide** (**33 b**): Compound **32 b** (1.50 g, 11.4 mmol) was dissolved in DCM (20 mL), and triethylamine (3.16 mL, 22.7 mmol, 2.0 equiv.) was added in one portion. A solution of butanoyl chloride (1.76 mL, 17.0 mmol, 1.5 equiv.) in DCM (10 mL) was added dropwise under stirring over a period of 1 h. Afterwards, the reaction mixture was stirred for 3 h at 40 °C. After completion of the reaction, DCM (100 mL) was added, and the mixture was successively extracted with a 2 M aq. HCl solution (100 mL) and a saturated aq. NaHCO_3_ solution (100 mL). Subsequently, the organic phase was washed with brine, dried over Na_2_SO_4_, filtrated, and concentrated under reduced pressure. The crude residue was purified by flash chromatography (ethyl acetate/*n*‐hexane with 20–50 % ethyl acetate) to obtain **33 b** as a slightly yellow solid (2.02 g, 10.0 mmol, 88 %): *R*_f_=0.58 (ethyl acetate/*n*‐hexane 2 : 3); mp: 106 °C; ^1^H‐NMR (400 MHz, DMSO‐d_6_): δ(ppm)=9.39 (s, 1H), 7.81 (d, *J*=8.3 Hz, 1H), 7.68 (dd, *J*=1.8, 0.9 Hz, 1H), 7.61 (dd, *J*=8.4, 2.0 Hz, 1H), 2.39 (t, *J*=7.3 Hz, 2H), 2.26 (s, 3H), 1.70–1.56 (m, 2H), 0.93 (t, *J*=7.4 Hz, 3H); ^13^C‐NMR (100 MHz, DMSO‐d_6_): δ(ppm)=171.6, 141.2, 134.0, 131.4, 130.1, 124.0, 119.0, 106.3, 37.9, 18.6, 17.6, 13.6; IR (ATR): 3399 (m, ν_N−H_), 2979 (w, ν_C−H_), 2216 (m, ν_C≡N_), 1646 (s, ν_C=O_).

***N***‐(**4**‐**Cyano**‐**2**‐**fluorophenyl**)**butyramide** (**33 c**): Compound **32 c** (700 mg, 5.14 mmol) was dissolved in DCM (20 mL), and triethylamine (1070 μL, 7.71 mmol, 1.5 equiv.) was added in one portion. A solution of butanoyl chloride (798 μL, 7.71 mmol, 1.5 equiv.) in DCM (20 mL) was added dropwise under stirring over a period of 1 h. The reaction mixture was stirred at room temperature. After 24 h, additional amounts of triethylamine (1070 μL, 7.71 mmol, 1.5 equiv.) and butanoyl chloride (798 μL, 7.71 mmol, 1.5 equiv.) were added, and the reaction was continued for 24 h. Afterwards, DCM (100 mL) was added, and the mixture was extracted successively with a 2 M aq. HCl solution (100 mL) and a saturated aq. NaHCO_3_ solution (100 mL). The organic phase was washed with brine, dried over Na_2_SO_4_, filtrated, and concentrated under reduced pressure. The crude residue was purified by silica gel column chromatography (ethyl acetate/*n*‐hexane 3 : 7) to obtain **33 c** as an orange solid (715 mg, 3.47 mmol, 67 %): *R*_f_=0.81 (ethyl acetate/*n*‐hexane 1 : 1); mp: 89 °C; ^1^H‐NMR (400 MHz, DMSO‐d_6_): δ(ppm)=10.03 (s, 1H), 8.26 (pseudo‐t, *J*=8.2 Hz, 1H), 7.86 (dd, *J*=11.1, 1.9 Hz, 1H), 7.66–7.60 (m, 1H), 2.41 (t, *J*=7.3 Hz, 2H), 1.64–1.52 (m, 2H), 0.89 (t, *J*=7.4 Hz, 3H); ^13^C‐NMR (100 MHz, DMSO‐d_6_): δ(ppm)=172.3, 151.7 (d, *J*=247.3 Hz), 131.6 (d, *J*=11.1 Hz), 129.3 (d, *J*=3.5 Hz), 123.0 (d, *J*=2.7 Hz), 119.3 (d, *J*=23.4 Hz), 117.9 (d, *J*=2.6 Hz), 105.7 (d, *J*=9.4 Hz), 37.8, 18.4, 13.5; IR (ATR): $\tilde{\nu }$ =3320 (w, ν_N−H_), 2232 (m, ν_C≡N_), 1698 (s, ν_C=O_).

***N***‐[**4**‐**Cyano**‐**2**‐(**trifluoromethyl**)**phenyl**]**butyramide** (**33 d**): Compound **32 d** (300 mg, 1.61 mmol) was dissolved in THF (20 mL). Triethylamine (453 μL, 3.22 mmol, 2.0 equiv.) and *N,N*‐dimethylpyridin‐4‐amine (20 mg, 0.16 mmol, 0.1 equiv.) were added. The reaction mixture was cooled to 0 °C, and a solution of butanoyl chloride (333 μL, 3.33 mmol, 2.0 equiv.) in THF (20 mL) was added dropwise under stirring over a period of 30 min. Thereafter, the reaction mixture was stirred at 70 °C. After 7 d, the reaction was terminated. Ethyl acetate (100 mL) was added, and the mixture was extracted successively with a 2 M aq. HCl solution (100 mL) and a saturated aq. NaHCO_3_ solution (100 mL). Subsequently, the organic phase was washed with brine, dried over Na_2_SO_4_, filtrated, and concentrated under reduced pressure. The crude residue was purified by silica gel column chromatography (ethyl acetate/*n*‐hexane 1 : 3) to obtain **33 d** as a colourless solid (230 mg, 0.90 mmol, 56 %): *R*_f_=0.79 (ethyl acetate/*n*‐hexane 1 : 1); mp: 107 °C; ^1^H‐NMR (400 MHz, DMSO‐d_6_): δ(ppm)=9.71 (s, 1H), 8.28 (d, *J*=1.9 Hz, 1H), 8.13 (dd, *J*=8.4, 1.9 Hz, 1H), 7.81 (d, *J*=8.2 Hz, 1H), 2.38 (t, *J*=7.3 Hz, 2H), 1.68–1.54 (m, 2H), 0.92 (t, *J*=7.4 Hz, 3H); ^13^C‐NMR (100 MHz, DMSO‐d_6_): δ(ppm)=172.2, 139.9, 136.6, 131.0 (q, *J*=5.0 Hz), 130.0, 124.2 (q, *J*=30.0 Hz), 122.6 (q, *J*=272.0 Hz), 117.5, 108.7, 37.5, 18.5, 13.4; IR (ATR): $\tilde{\nu }$ =3273 (m, ν_N−H_), 3072, 2964 (w, 2237 ν_C−H_), 2235 (m, ν_C≡N_), 1671 (s, ν_C=O_) 1618 (m, δ_N−H_).

***N***‐(**4**‐**Cyano**‐**2**‐**methylphenyl**)**nicotinamide** (**33 e**): Compound **32 b** (700 mg, 5.30 mmol) was dissolved in DCM (40 mL), and triethylamine (1111 μL, 7.95 mmol, 1.5 equiv.) was added in one portion. Subsequently, pyridine‐3‐carbonyl chloride (1.50 g, 10.6 mmol, 2.0 equiv.) was added in small portions under stirring over a period of 1 h. Afterwards, the reaction mixture was stirred at room temperature. After 65 h, another portion of pyridine‐3‐carbonyl chloride (750 mg, 5.3 mmol, 1.0 equiv.) was added, and the reaction was stirred at 40 °C. After 24 h, DCM (100 mL) was added, and the mixture was extracted with a saturated aq. NaHCO_3_ solution (3×100 mL). Afterwards, the organic phase was washed with brine, dried over Na_2_SO_4_, filtrated, and freed from solvent under reduced pressure. The crude residue was purified by flash chromatography (100 % ethyl acetate) to obtain **33 e** as a colourless solid (857 mg, 3.61 mmol, 68 %): *R*_f_=0.54 (ethyl acetate/*n*‐hexane 2 : 3); mp: 191 °C; ^1^H‐NMR (400 MHz, DMSO‐d_6_): δ(ppm)=10.25 (s, 1H), 9.14 (dd, *J*=2.4, 0.9 Hz, 1H), 8.80 (dd, *J*=4.8, 1.7 Hz, 1H), 8.32 (ddd, *J*=7.9, 2.3, 1.7 Hz, 1H), 7.83–7.77 (m, 1H), 7.76–7.67 (m, 2H), 7.60 (ddd, *J*=7.9, 4.8, 0.9 Hz, 1H), 2.34 (d, *J*=0.8 Hz, 3H); ^13^C‐NMR (100 MHz, DMSO‐d_6_): δ(ppm)=164.7, 152.9, 149.3, 141.2, 136.1, 134.7, 134.6, 130.6, 130.3, 126.7, 124.0, 119.3, 108.5, 18.1; IR (ATR): 3300 (m, ν_N−H_), 3028, 2997 (w, ν_C−H_), 2228 (m, ν_C≡N_), 1655 (s, ν_C=O_).

***N***‐(**4**‐**Formyl**‐**2,6**‐**dimethylphenyl**)**butyramide** (**34 a**): The synthesis was conducted from **33 a** (1.40 g, 6.5 mmol) according to the general procedure (nitrile reduction). The purification was carried out by silica gel column chromatography (ethyl acetate/*n*‐hexane 1 : 1) to obtain **34 a** as an off‐white solid (1.26 g, 5.7 mmol, 88 %): *R*_f_=0.38 (ethyl acetate/*n*‐hexane 1 : 1); mp: 136 °C; ^1^H‐NMR (400 MHz, DMSO‐d_6_): δ(ppm)=9.92 (s, 1H), 9.45 (s, 1H), 7.61 (s, 2H), 2.34 (t, *J*=7.3 Hz, 2H), 2.22 (s, 6H), 1.72–1.58 (m, 2H), 0.96 (t, *J*=7.4 Hz, 3H); ^13^C‐NMR (100 MHz, DMSO‐d_6_): δ(ppm)=192.5, 170.6, 141.4, 136.1, 134.0, 128.8, 37.4, 18.8, 18.2, 13.7; IR (ATR): $\tilde{\nu }$ =3245 (m, ν_N−H_), 2958 (w, ν_C−H_), 1693, 1647 (s, ν_C=O_).

***N***‐(**4**‐**Formyl**‐**2**‐**methylphenyl**)**butyramide** (**34 b**): The synthesis was carried out from **33 b** (2.02 g, 10.0 mmol) in accordance with the general procedure (nitrile reduction). The crude residue was further purified by silica gel column chromatography (ethyl acetate/*n*‐hexane 1 : 1) to obtain **34 b** as an off‐white solid (1.52 g, 7.4 mmol, 74 %): *R*_f_=0.71 (ethyl acetate); mp: 79 °C; ^1^H‐NMR (400 MHz, DMSO‐d_6_): δ(ppm)=9.89 (s, 1H), 9.37 (s, 1H), 7.85 (d, *J*=8.2 Hz, 1H), 7.74 (d, *J*=1.6 Hz, 1H), 7.71 (dd, *J*=8.2, 2.0 Hz, 1H), 2.40 (t, *J*=7.3 Hz, 2H), 2.31 (s, 3H), 1.71–1.57 (m, 2H), 0.94 (t, *J*=7.4 Hz, 3H); ^13^C‐NMR (100 MHz, DMSO‐d_6_): δ(ppm)=192.4, 172.0, 142.9, 132.7, 132.1, 131.1, 128.2, 124.1, 38.4, 19.1, 18.3, 14.1; IR (ATR): $\tilde{\nu }$ =3288 (m, ν_N−H_), 2965 (w, ν_C−H_), 1698, 1653 (s, ν_C=O_).

***N***‐(**2**‐**Fluoro**‐**4**‐**formylphenyl**)**butyramide** (**34 c**): The synthesis was conducted from **33 c** (650 mg, 3.15 mmol) according to the general procedure (nitrile reduction). The purification was carried out by silica gel column chromatography (ethyl acetate/*n*‐hexane 3 : 7) to obtain **34 c** as an off‐white solid (483 mg, 2.31 mmol, 73 %): *R*_f_=0.56 (ethyl acetate/*n*‐hexane 3 : 7); mp: 87 °C; ^1^H‐NMR (400 MHz, DMSO‐d_6_): δ(ppm)=9.99 (s, 1H), 9.88 (d, *J*=1.9 Hz, 1H), 8.32 (dd, *J*=8.6, 7.5 Hz, 1H), 7.77–7.68 (m, 2H), 2.43 (t, *J*=7.3 Hz, 2H), 1.67–1.55 (m, 2H), 0.90 (t, *J*=7.4 Hz, 3H); ^13^C‐NMR (100 MHz, DMSO‐d_6_): δ(ppm)=191.0 (d, *J*=2.1 Hz), 172.2, 152.5 (d, *J*=247.5 Hz), 132.4 (d, *J*=11.5 Hz), 132.1 (d, *J*=5.5 Hz), 126.8 (d, *J*=2.9 Hz), 122.5 (d, *J*=2.1 Hz), 115.2 (d, *J*=19.8 Hz), 37.9, 18.4, 13.5; IR (ATR): $\tilde{\nu }$ =3313 (m, ν_N−H_), 3012, 2962 (w, ν_C−H_), 1706, 1672 (s, ν_C=O_), 1609 (s, δ_N−H_).

***N***‐[**4**‐**Formyl**‐**2**‐(**trifluoromethyl**)**phenyl**]**butyramide** (**34 d**): The synthesis was carried out from **33 d** (673 mg, 2.63 mmol) in accordance with the general procedure (nitrile reduction). The crude residue was further purified by silica gel column chromatography (ethyl acetate/*n*‐hexane 1 : 3) to obtain **34 d** as a colourless solid (372 mg, 1.44 mmol, 55 %): *R*_f_=0.76 (ethyl acetate/*n*‐hexane 1 : 1); mp: 70 °C; ^1^H‐NMR (400 MHz, DMSO‐d_6_): δ(ppm)=10.04 (s, 1H), 9.68 (s, 1H), 8.25 (d, *J*=1.9 Hz, 1H), 8.16 (dd, *J*=8.1, 1.7 Hz, 1H), 7.84 (d, *J*=8.2 Hz, 1H), 2.39 (t, *J*=7.3 Hz, 2H), 1.69–1.55 (m, 2H), 0.93 (t, *J*=7.4 Hz, 3H); ^13^C‐NMR (100 MHz, DMSO‐d_6_): δ(ppm)=191.5, 172.2, 140.7, 133.3, 132.8, 129.8, 128.2 (q, *J*=5.1 Hz), 123.9 (q, *J*=30.0 Hz), 123.1 (q, *J*=272.0 Hz), 37.6, 18.5, 13.4; IR (ATR): $\tilde{\nu }$ =3289 (m, ν_N−H_), 2969 (w, ν_C−H_), 1701, 1668 (s, ν_C=O_).

***N***‐(**4**‐**Formyl**‐**2**‐**methylphenyl**)**nicotinamide** (**34 e**): The synthesis was conducted from **33 e** (781 mg, 3.29 mmol) according to the general procedure (nitrile reduction). The purification was carried out by silica gel column chromatography (100 % ethyl acetate) to obtain **34 e** as a colourless solid (290 mg, 1.21 mmol, 37 %): *R*_f_=0.56 (ethyl acetate); mp: 156 °C; ^1^H‐NMR (400 MHz, DMSO‐d_6_): δ(ppm)=10.23 (s, 1H), 9.97 (s, 1H), 9.15 (dd, *J*=2.4, 0.9 Hz, 1H), 8.79 (dd, *J*=4.8, 1.7 Hz, 1H), 8.33 (ddd, *J*=7.9, 2.4, 1.7 Hz, 1H), 7.84 (d, *J*=1.9 Hz, 1H), 7.79 (dd, *J*=8.2, 1.9 Hz, 1H), 7.75 (d, *J*=8.2 Hz, 1H), 7.60 (ddd, *J*=7.9, 4.8, 0.9 Hz, 1H), 2.38 (s, 3H); ^13^C‐NMR (100 MHz, DMSO‐d_6_): δ(ppm)=192.3, 164.2, 152.4, 148.8, 141.8, 135.6, 133.5, 133.4, 131.7, 129.9, 127.6, 125.9, 123.6, 17.9; IR (ATR): $\tilde{\nu }$ =3298 (m, ν_N−H_), 3039, 2813 (w, ν_C−H_), 1693, 1652 (s, ν_C=O_).

***N***‐(**4**‐**{**[(**4**‐**Fluorophenyl**)**amino**]**methyl}**‐**2,6**‐**dimethylphenyl**)**butyramide** (**35 a**): The synthesis was carried out from **34 a** (250 mg, 1.14 mmol) and 4‐fluoroaniline (130 μL, 1.37 mmol, 1.2 equiv.) in accordance with the general procedure (reductive amination). The crude residue was further purified by silica gel column chromatography (ethyl acetate/*n*‐hexane 3 : 2) and subsequent recrystallization (methanol/water) to obtain **35 a** as a colourless solid (226 mg, 0.72 mmol, 63 %): *R*_f_=0.72 (ethyl acetate/*n*‐hexane 6 : 4); mp: 125 °C; ^1^H‐NMR (400 MHz, DMSO‐d_6_): δ(ppm)=9.09 (s, 1H), 7.03 (s, 2H), 6.93–6.82 (m, 2H), 6.58–6.48 (m, 2H), 6.08 (t, *J*=5.9 Hz, 1H), 4.12 (d, *J*=5.9 Hz, 2H), 2.27 (t, *J*=7.3 Hz, 2H), 2.10 (s, 6H), 1.70–1.56 (m, 2H), 0.94 (t, *J*=7.4 Hz, 3H); ^13^C‐NMR (100 MHz, DMSO‐d_6_): δ(ppm)=170.7, 154.2 (d, *J*=230.7 Hz), 145.4, 137.8, 134.9, 134.0, 126.4, 115.1 (d, *J*=21.8 Hz), 112.9 (d, *J*=7.3 Hz), 46.7, 37.4, 18.9, 18.2, 13.7; IR (ATR): $\tilde{\nu }$ =3270 (w, ν_N−H_), 3013, 2963 (w, ν_C−H_), 1654 (s, ν_C=O_); ESI‐HRMS (*m/z*): calcd. for [C_19_H_24_N_2_OF+H]^+^ 315.1867, found 315.1866; cpd purity (220 nm): 100 %.

***N***‐(**4**‐**{**[(**4**‐**Fluorophenyl**)**amino**]**methyl}**‐**2**‐**methylphenyl**)**butyramide** (**35 b**): The synthesis was conducted from **34 b** (500 mg, 2.44 mmol) and 4‐fluoroaniline (257 μL, 2.68 mmol, 1.1 equiv.) according to the general procedure (reductive amination). The purification was carried out by flash chromatography (ethyl acetate/*n*‐hexane, 30–40 % ethyl acetate) and subsequent recrystallization (methanol/water) to obtain **35 b** as a colourless solid (480 mg, 1.60 mmol, 66 %): *R*_f_=0.54 (ethyl acetate/*n*‐hexane 2 : 3); mp: 92 °C; ^1^H‐NMR (400 MHz, DMSO‐d_6_): δ(ppm)=9.17 (s, 1H), 7.27 (d, *J*=8.1 Hz, 1H), 7.17 (d, *J*=2.1 Hz, 1H), 7.11 (dd, *J*=8.2, 2.1 Hz, 1H), 6.92–6.81 (m, 2H), 6.58–6.48 (m, 2H), 6.09 (t, *J*=6.0 Hz, 1H), 4.15 (d, *J*=5.9 Hz, 2H), 2.28 (t, *J*=7.3 Hz, 2H), 2.15 (s, 3H), 1.68–1.54 (m, 2H), 0.93 (t, *J*=7.4 Hz, 3H); ^13^C‐NMR (100 MHz, DMSO‐d_6_): δ(ppm)=171.0, 154.2 (d, *J*=230.7 Hz), 145.4 (d, *J*=1.6 Hz), 136.7, 135.0, 131.8, 129.0, 125.3, 124.7, 115.1 (d, *J*=22.0 Hz), 112.9 (d, *J*=7.3 Hz), 46.6, 37.7, 18.8, 18.0, 13.6; IR (ATR): $\tilde{\nu }$ =3270 (w, ν_N−H_), 3013, 2963 (w, ν_C−H_), 1654 (s, ν_C=O_); ESI‐HRMS (*m/z*): calcd. for [C_18_H_22_N_2_OF+H]^+^ 301.1711, found 301.1714; cpd purity (220 nm): 100 %.

***N***‐(**2**‐**Fluoro**‐**4**‐**{**[(**4**‐**fluorophenyl**)**amino**]**methyl}phenyl**)**butyramide** (**35 c**): The synthesis was carried out from **34 c** (480 mg, 2.29 mmol) 4‐fluoroaniline (242 μL, 2.52 mmol, 1.1 equiv.) in accordance with the general procedure (reductive amination). The crude residue was further purified by silica gel column chromatography (ethyl acetate/*n*‐hexane 3 : 7) and subsequent recrystallization (methanol/water) to obtain **35 c** as a colourless solid (508 mg, 1.67 mmol, 73 %): *R*_f_=0.76 (ethyl acetate/*n*‐hexane 1 : 1); mp: 123 °C; ^1^H‐NMR (400 MHz, DMSO‐d_6_): δ(ppm)=9.55 (s, 1H), 7.71 (pseudo‐t, *J*=8.2 Hz, 1H), 7.16 (dd, *J*=11.8, 1.9 Hz, 1H), 7.10 (dd, *J*=8.3, 1.9 Hz, 1H), 6.91–6.81 (m, 2H), 6.57–6.47 (m, 2H), 6.16 (t, *J*=6.1 Hz, 1H), 4.19 (d, *J*=6.1 Hz, 2H), 2.30 (d, *J*=14.6 Hz, 1H), 1.62–1.50 (m, 2H), 0.89 (t, *J*=7.4 Hz, 3H); ^13^C‐NMR (100 MHz, DMSO‐d_6_): δ(ppm)=171.4, 154.4 (d, *J*=230 Hz), 153.9 (d, *J*=244 Hz), 145.1 (d, *J*=1.5 Hz), 138.0 (*J*=6.4 Hz), 124.6, 124.4, 122.7 (d, *J*=3.1 Hz), 115.2 (d, *J*=22.0 Hz), 113.9 (d, *J*=20.2 Hz), 113.1 (d, *J*=7.3 Hz), 46.1, 37.6, 18.6, 13.5; IR (ATR): $\tilde{\nu }$ =3430, 3300 (m, ν_N−H_), 3034, 2963 (w, ν_C−H_), 1672 (s, ν_C=O_), 1599 (s, δ_N−H_). ESI‐HRMS (*m/z*): calcd. for [C_17_H_18_F_2_N_2_O+H]^+^ 305.1460, found 305.1461; cpd purity (220 nm): 100 %.

***N***‐(**4**‐**{**[(**4**‐**Fluorophenyl**)**amino**]**methyl}**‐**2**‐(**trifluoromethyl**)**phenyl**)**butyramide hydrochloride** (**35 d**): The synthesis was conducted from **34 d** (320 mg, 1.23 mmol) and 4‐fluoroaniline (140 μL, 1.48 mmol, 1.2 equiv.) according to the general procedure (reductive amination). The purification was carried out by silica gel column chromatography (ethyl acetate/*n*‐hexane 1 : 3), which yielded the product as a colourless oil. To obtain a solid, the residue was dissolved in ethyl acetate. The resulting solution was cooled to 0 °C, and HCl gas was passed through the solution for 30 min to precipitate a hydrochloride salt. The precipitate was filtered off to obtain **35 d** as an off‐white solid (284 mg, 0.72 mmol, 59 %): *R*_f_=0.74 (ethyl acetate/*n*‐hexane 2 : 3); mp: 193 °C (decomp.); ^1^H‐NMR (400 MHz, MeOH‐d_4_): δ(ppm)=7.83 (d, *J*=2.0 Hz, 1H), 7.74 (dd, *J*=8.3, 2.1 Hz, 1H), 7.66 (d, *J*=8.3 Hz, 1H), 7.53–7.43 (m, 2H), 7.37–7.27 (m, 2H), 4.71 (s, 2H), 2.44 (t, *J*=7.4 Hz, 2H), 1.83–1.69 (m, 2H), 1.04 (t, *J*=7.4 Hz, 3H); ^13^C‐NMR (100 MHz, MeOH‐d_4_): δ(ppm)=175.9, 164.2 (d, *J*=250.2 Hz), 137.8, 136.0, 132.8, 131.8, 131.2, 129.9, 127.4 (q, *J*=30.3 Hz), 126.4, 124.8 (q, *J*=271.3 Hz), 118.4 (d, *J*=23.7 Hz), 55.7, 39.2, 20.3, 14.1; IR (ATR): $\tilde{\nu }$ =3286 (w, ν_N−H_), 2973 (w, ν_C−H_), 1666 (s, ν_C=O_); ESI‐HRMS (*m/z*): calcd. for [C_18_H_19_N_2_OF_4_+H]^+^ 355.1428, found 355.1427; cpd purity (220 nm): 99 %.

***N***‐(**4**‐**{**[(**2,4**‐**Difluorophenyl**)**amino**]**methyl}**‐**2**‐**methylphenyl**)**nicotinamide** (**35 e**): The synthesis was carried out from **34 e** (288 mg, 1.20 mmol) and 2,4‐difluoroaniline (179 μL, 1.80 mmol, 1.5 equiv.) in accordance with the general procedure (reductive amination). The crude residue was further purified by silica gel column chromatography (ethyl acetate/*n*‐hexane 4 : 1) and subsequent recrystallization (methanol/water) to obtain **35 e** as a colourless solid (180 mg, 0.51 mmol, 42 %): *R*_f_=0.42 (ethyl acetate); mp: 147 °C; ^1^H‐NMR (400 MHz, DMSO‐d_6_): δ(ppm)=10.01 (s, 1H), 9.15–9.09 (m, 1H), 8.76 (dd, *J*=4.8, 1.7 Hz, 1H), 8.29 (dt, *J*=8.1, 2.0 Hz, 1H), 7.56 (ddd, *J*=7.9, 4.8, 0.9 Hz, 1H), 7.32–7.24 (m, 2H), 7.20 (dd, *J*=8.1, 2.0 Hz, 1H), 7.07 (ddd, *J*=11.9, 8.9, 2.9 Hz, 1H), 6.78 (tdd, *J*=8.8, 2.9, 1.4 Hz, 1H), 6.54 (ddd, *J*=10.1, 9.0, 5.6 Hz, 1H), 6.08 (td, *J*=6.2, 2.0 Hz, 1H), 4.30 (d, *J*=6.1 Hz, 2H), 2.22 (s, 3H); ^13^C‐NMR (100 MHz, DMSO‐d_6_): δ(ppm)=163.9, 152.1, 152.8 (dd, *J*=233.7, 11.0 Hz), 150.0 (dd, *J*=241.2, 11.9 Hz), 148.6, 137.7, 135.4, 134.5, 133.6, 133.4 (dd, *J*=11.7, 2.6 Hz), 130.1, 128.9, 126.5, 124.6, 123.5, 112.1 (dd, *J*=8.8, 5.2 Hz), 110.6 (dd, *J*=21.2, 3.5 Hz), 103.4 (dd, *J*=26.8, 22.7 Hz), 45.9, 18.0; IR (ATR): $\tilde{\nu }$ =3293 (m, ν_N−H_), 1644 (s, ν_C=O_), 1590 (s, δ_N−H_); ESI‐HRMS (*m/z*): calcd. for [C_20_H_18_N_3_OF_2_+H]^+^ 354.1408, found 354.1410; cpd purity (220 nm): 100 %.

***N***‐(**2**‐**Methyl**‐**4**‐**{**[(**5**‐**methylpyridin**‐**2**‐**yl**)**amino**]**methyl}phenyl**)**butyramide** (**35 f**): The synthesis was conducted from **34 b** (500 mg, 2.44 mmol) and 5‐methylpyridin‐2‐amine (290 mg, 2.68 mmol, 1.1 equiv.) according to the general procedure (reductive amination). The purification was carried out by flash chromatography (ethyl acetate/*n*‐hexane, 30–40 % ethyl acetate) and subsequent recrystallization (methanol/water) to obtain **35 f** as a colourless solid (480 mg, 1.51 mmol, 62 %): *R*_f_=0.48 (ethyl acetate/*n*‐hexane 2 : 3); mp: 134 °C; ^1^H‐NMR (400 MHz, DMSO‐d_6_): δ(ppm)=9.17 (s, 1H), 7.80–7.74 (m, 1H), 7.23 (d, *J*=8.1 Hz, 1H), 7.19 (dd, *J*=8.5, 2.4 Hz, 1H), 7.13 (d, *J*=2.0 Hz, 1H), 7.07 (dd, *J*=8.1, 2.1 Hz, 1H), 6.72 (t, *J*=6.0 Hz, 1H), 6.41 (d, *J*=8.4 Hz, 1H), 4.36 (d, *J*=6.0 Hz, 2H), 2.27 (t, *J*=7.3 Hz, 2H), 2.14 (s, 3H), 2.07 (s, 3H), 1.67–1.54 (m, 2H), 0.92 (t, *J*=7.4 Hz, 3H); ^13^C‐NMR (100 MHz, DMSO‐d_6_): δ(ppm)=170.9, 156.9, 146.9, 137.6, 137.5, 134.8, 131.7, 129.0, 125.2, 124.7, 119.7, 107.7, 44.0, 37.7, 18.8, 18.0, 17.0, 13.6; IR (ATR): $\tilde{\nu }$ =3251 (m, ν_N−H_), 3086, 2921 (w, ν_C−H_), 1650 (s, ν_C=O_), 1612 (s, δ_N−H_); ESI‐HRMS (*m/z*): calcd. for [C_18_H_23_N_3_O+H]^+^ 298.1914, found 298.1913; cpd purity (220 nm): 100 %.

**Ethyl 6**‐**aminonicotinate** (**37**): 6‐Amino‐3‐carboxylic acid (12.50 g, 90.5 mmol) was suspended in dry ethanol (250 mL). The suspension was set under an argon atmosphere. Thionyl chloride (19.7 mL, 272 mmol, 3.0 equiv.) was added dropwise, and the suspension was stirred at 90 °C. After 18 h, the reaction mixture was concentrated under reduced pressure. The residue was dissolved in ethyl acetate (250 mL) and extracted with an aq. saturated NaHCO_3_ solution (250 mL). The organic phase was washed with brine, dried over Na_2_SO_4_, filtrated, and concentrated to dryness under reduced pressure to obtain **37** as an off‐white solid (13.20 g, 79.4 mmol, 88 %): *R*_f_=0.38 (ethyl acetate/*n*‐hexane 1 : 1); mp: 153 °C; ^1^H‐NMR (400 MHz, DMSO‐d_6_): δ(ppm)=8.48 (dd, *J*=2.4, 0.8 Hz, 1H), 7.80 (dd, *J*=8.7, 2.4 Hz, 1H), 6.79 (s, 2H), 6.43 (dd, *J*=8.8, 0.8 Hz, 1H), 4.21 (q, *J*=7.1 Hz, 2H), 1.26 (t, *J*=7.1 Hz, 3H); ^13^C‐NMR (100 MHz, DMSO‐d_6_): δ(ppm)=165.2, 162.5, 151.0, 137.5, 113.5, 107.0, 59.8, 14.3; IR (ATR): $\tilde{\nu }$ =3409 (m, ν_N−H_), 2977 (w, ν_C−H_), 1687 (s, ν_C=O_), 1597 (s, δ_N−H_).

**Ethyl 6**‐**amino**‐**5**‐**bromonicotinate** (**38**): Compound **37** (7.80 g, 46.9 mmol) was dissolved in dry THF (70 mL). The solution was set under an argon atmosphere and cooled to 0 °C. NBS (8.77 g, 49.3 mmol, 1.1 equiv.) was added in portions under stirring. Afterwards, the cooling was removed, and the mixture was stirred at room temperature with the exclusion of light. After 17 h, the mixture was poured into an ice‐cold saturated aq. NaHCO_3_ solution (200 mL), which was subsequently extracted with ethyl acetate (3×200 mL). The combined organic phases were washed with brine, dried over Na_2_SO_4_, filtrated, and concentrated under reduced pressure. The crude residue was purified by silica gel column chromatography (ethyl acetate/*n*‐hexane 3 : 7) to obtain **38** as an off‐white solid (8.80 g, 35.9 mmol, 77 %): *R*_f_=0.71 (ethyl acetate/*n*‐hexane 1 : 1); mp: 129 °C; ^1^H‐NMR (400 MHz, DMSO‐d_6_): δ(ppm)=8.48 (d, *J*=2.0 Hz, 1H), 8.06 (d, *J*=2.0 Hz, 1H), 7.13 (s, 2H), 4.23 (q, *J*=7.1 Hz, 2H), 1.27 (t, *J*=7.1 Hz, 3H); ^13^C‐NMR (100 MHz, DMSO‐d_6_): δ(ppm)=164.0, 159.1, 149.4, 140.1, 115.3, 101.9, 60.3, 14.2; IR (ATR): $\tilde{\nu }$ =3431 (m, ν_N−H_), 2981 (w, ν_C−H_), 1707 (s, ν_C=O_), 1635 (s, δ_N−H_).

(**6**‐**Amino**‐**5**‐**bromopyridin**‐**3**‐**yl**)**methanol** (**39**): Compound **38** (3.00 g, 12.2 mmol) was set under an argon atmosphere and dissolved in dry THF (30 mL). A 2.96 mM solution of LiAlH_4_ in THF (4.96 mL, 14.7 mmol, 1.2 equiv.) was added dropwise. The reaction mixture was stirred at room temperature. After 2 h, additional LiAlH_4_ solution (4.96 mL, 14.7 mmol, 1.2 equiv.) was added, and stirring was continued for 3 h. Afterwards, the reaction was quenched by the addition of water (0.5 mL). The resulting precipitate was filtered off and rinsed with ethyl acetate (200 mL). The filtrate was washed with brine, dried over Na_2_SO_4_, filtrated, and concentrated under reduced pressure. The crude residue was purified by silica gel column chromatography (DCM/methanol 9 : 1) to obtain **39** as a yellow solid (1.35 g, 6.7 mmol, 54 %): *R*_f_=0.30 (ethyl acetate/*n*‐hexane 3 : 1); mp: 113 °C; ^1^H‐NMR (400 MHz, DMSO‐d_6_): δ(ppm)=7.86 (d, *J*=2.0 Hz, 1H), 7.64 (d, *J*=2.0 Hz, 1H), 6.07 (s, 2H), 5.02 (t, *J*=5.7 Hz, 1H), 4.29 (d, *J*=5.7 Hz, 2H); ^13^C‐NMR (100 MHz, DMSO‐d_6_): δ(ppm)=155.4, 145.7, 139.4, 127.7, 102.8, 59.9; IR (ATR): 3400–3000 (b, ν_O‐H_), 3373 (m, ν_N−H_), 2952 (w, ν_C−H_), 1603 (s, δ_N−H_).

**6**‐**Amino**‐**5**‐**bromonicotinaldehyde** (**40**): Compound **39** (2.56 g, 12.6 mmol) was suspended in toluene. Activated MnO_2_ (3.01 g, 34.6 mmol, 2.8 equiv.) was added in one portion, and the reaction mixture was stirred at 80 °C. After 2 h, it was filtered through a pad of celite, which was subsequently rinsed with ethyl acetate. The combined filtrates were concentrated under reduced pressure to obtain **40** as a yellow solid (1.03 g, 5.1 mmol, 41 %): *R*_f_=0.49 (ethyl acetate/*n*‐hexane 1 : 1); mp: 177 °C; ^1^H‐NMR (400 MHz, DMSO‐d_6_): δ(ppm)=9.67 (s, 1H), 8.47 (d, *J*=1.9 Hz, 1H), 8.06 (d, *J*=1.9 Hz, 1H), 7.40 (s, 2H); ^13^C‐NMR (100 MHz, DMSO‐d_6_): δ(ppm)=188.5, 159.7, 152.6, 138.8, 123.5, 103.2; IR (ATR): $\tilde{\nu }$ =3474 (m, ν_N−H_), 2988 (w, ν_C−H_), 1672 (s, ν_C=O_), 1634 (s, δ_N−H_).

***N***‐(**3**‐**Bromo**‐**5**‐**formylpyridin**‐**2**‐**yl**)**butyramide** (**41 a**): Compound **40** (2.00 g, 10.0 mmol) was dissolved in DCM (100 mL). DIPEA (3.47 mL, 19.9 mmol, 2.0 equiv.) was added in one portion. The reaction mixture was cooled to 0 °C, and a solution of 3,3‐dimethylbutanoyl chloride (2.06 mL, 19.9 mmol, 2.0 equiv.) in DCM (20 mL) was added dropwise over a period of 30 min. The cooling was removed, and the reaction mixture was stirred at room temperature. After 16 h, additional DCM (200 mL) was added, and the solution was extracted successively with a saturated aq. NaHCO_3_ solution (200 mL), and a 2 M aq. HCl solution (200 mL). The organic phase was washed with brine, dried over Na_2_SO_4_, filtrated, and concentrated under reduced pressure. The crude residue was purified by flash chromatography (ethyl acetate/*n*‐hexane, 30–70 % ethyl acetate) to obtain **41 a** as a colourless solid (1.35 g, 5.0 mmol, 50 %): *R*_f_=0.52 (ethyl acetate/*n*‐hexane 1 : 1); mp: 101 °C; ^1^H‐NMR (400 MHz, DMSO‐d_6_): δ(ppm)=10.37 (s, 1H), 10.00 (s, 1H), 8.88 (d, *J*=1.9 Hz, 1H), 8.48 (d, *J*=1.9 Hz, 1H), 2.37 (t, *J*=7.3 Hz, 2H), 1.76–1.42 (m, 2H), 0.93 (t, *J*=7.4 Hz, 3H); ^13^C‐NMR (100 MHz, DMSO‐d_6_): δ(ppm)=190.4, 171.3, 153.6, 149.1, 141.7, 130.1, 115.8, 37.5, 18.3, 13.6; IR (ATR): $\tilde{\nu }$ =3248 (m, ν_N−H_), 3052, 2963 (w, ν_C−H_), 1687, 1670 (s, ν_C=O_).

***N***‐(**3**‐**Bromo**‐**5**‐**formylpyridin**‐**2**‐**yl**)‐**3,3**‐**dimethylbutanamide** (**41 b**): Compound **40** (500 mg, 2.49 mmol) was dissolved in DCM (25 mL). DIPEA (866 μL, 4.98 mmol, 2.0 equiv.) was added in one portion. The reaction mixture was cooled to 0 °C, and a solution of 3,3‐dimethylbutanoyl chloride (415 μL, 2.99 mmol, 1.2 equiv.) in DCM (5 mL) was added dropwise over a period of 30 min. The cooling was removed, and the reaction mixture was stirred at room temperature. After 16 h, additional 3,3‐dimethylbutanoyl chloride (415 μL, 2.99 mmol, 1.2 equiv.) dissolved in DCM (5 mL) was added dropwise at 0 °C. Afterwards, stirring was continued at room temperature. After another 8 h, DCM (100 mL) was added, and the solution was extracted successively with saturated aq. NaHCO_3_ solution (100 mL) and 2 M aq. HCl solution (100 mL). The organic phase was washed with brine, dried over Na_2_SO_4_, filtrated, and concentrated under reduced pressure. The crude residue was purified by flash chromatography (ethyl acetate/*n*‐hexane, 30–70 % ethyl acetate) to obtain **41 b** as slightly yellow oil (360 mg, 1.20 mmol, 48 %): *R*_f_=0.59 (ethyl acetate/*n*‐hexane 1 : 1); ^1^H‐NMR (400 MHz, DMSO‐d_6_): δ(ppm)=10.34 (s, 1H), 10.01 (s, 1H), 8.89 (d, *J*=2.0 Hz, 1H), 8.48 (d, *J*=1.9 Hz, 1H), 2.27 (s, 2H), 1.04 (s, 9H); ^13^C‐NMR (100 MHz, DMSO‐d_6_): δ(ppm)=190.4, 169.9, 153.6, 149.0, 141.8, 130.2, 116.0, 48.6, 31.0, 29.7; IR (ATR): $\tilde{\nu }$ =3241 (m, ν_N−H_), 3050, 2905 (w, ν_C−H_), 1689, (s, ν_C=O_).

***N***‐(**3**‐**Bromo**‐**5**‐**{**[(**4**‐**fluorophenyl**)**amino**]**methyl}pyridin**‐**2**‐**yl**)**butyramide** (**42 a**): The synthesis was conducted from **41 a** (1.29 g, 4.77 mmol) and 4‐fluoroaniline (542 μL, 5.719 mmol, 1.2 equiv.) according to the general procedure (reductive amination). The purification was carried out by silica gel column chromatography (ethyl acetate/*n*‐hexane 1 : 1), yielding **42 a** as a colourless oil. To obtain a solid, the residue was dissolved in methanol, followed by the addition of ice‐cold water to form a precipitate, which was filtered off, yielding the title compound a colourless solid (998 mg, 2.73 mmol, 57 %): *R*_f_=0.31 (ethyl acetate/*n*‐hexane 1 : 1); mp: 151 °C; ^1^H‐NMR (400 MHz, DMSO‐d_6_): δ(ppm)=10.03 (s, 1H), 8.40 (d, *J*=2.0 Hz, 1H), 8.05 (d, *J*=2.0 Hz, 1H), 6.96–6.85 (m, 2H), 6.63–6.54 (m, 2H), 6.22 (t, *J*=6.3 Hz, 1H), 4.28 (d, *J*=6.2 Hz, 2H), 2.28 (t, *J*=7.3 Hz, 2H), 1.65–1.54 (m, 2H), 0.93 (t, *J*=7.4 Hz, 3H); ^13^C‐NMR (100 MHz, DMSO‐d_6_): δ(ppm)=171.2, 154.5 (d, *J*=231.7 Hz), 148.1, 146.5, 144.7 (d, *J*=1.5 Hz), 140.6, 135.6, 117.3, 115.3 (d, *J*=22.0 Hz), 113.2 (d, *J*=7.4 Hz), 43.4, 37.2, 18.4, 13.6; IR (ATR): $\tilde{\nu }$ =3239 (m, ν_N−H_), 2966 (w, ν_C−H_), 1665 (s, ν_C=O_). ESI‐HRMS (*m/z*): calcd. for [C_16_H_17_BrFN_3_O+H]^+^ 366.0612, found 366.0608; cpd purity (220 nm): 100 %.

***N***‐(**3**‐**Bromo**‐**5**‐**{**[(**4**‐**fluorophenyl**)**amino**]**methyl}pyridin**‐**2**‐**yl**)‐**3,3**‐**dimethylbutanamide** (**42 b**): The synthesis was carried out from **41 b** (320 mg, 1.07 mmol) and 4‐fluoroaniline (123 μL, 1.28 mmol, 1.2 equiv.) in accordance with the general procedure (reductive amination). The crude residue was further purified by silica gel column chromatography (ethyl acetate/*n*‐hexane 1 : 1), yielding the product as a colourless oil. To obtain a solid, the residue was dissolved in methanol, followed by the addition of ice‐cold water to form a precipitate, which was filtered off, yielding **42 b** as a colourless solid (310 mg, 0.79 mmol, 74 %): *R*_f_=0.39 (ethyl acetate/*n*‐hexane 1 : 1); mp: 141 °C; ^1^H‐NMR (400 MHz, DMSO‐d_6_): δ(ppm)=9.96 (s, 1H), 8.38 (d, *J*=2.1 Hz, 1H), 8.03 (d, *J*=2.0 Hz, 1H), 6.94–6.83 (m, 2H), 6.61–6.52 (m, 2H), 6.20 (t, *J*=6.3 Hz, 1H), 4.26 (d, *J*=6.2 Hz, 2H), 2.17 (s, 2H), 1.03 (s, 9H); ^13^C‐NMR (100 MHz, DMSO‐d_6_): δ(ppm)=169.8, 154.5 (d, *J*=231.4 Hz), 148.2, 146.5, 144.7, 140.6, 135.6, 117.2, 115.3 (d, *J*=22.0 Hz), 113.2 (d, *J*=7.3 Hz), 48.5, 43.3, 30.8, 29.7; IR (ATR): $\tilde{\nu }$ =3383, 3311 (m, ν_N−H_), 3059, 2958 (w, ν_C−H_), 1667 (s, ν_C=O_), 1576 (s, δ_N−H_).

***N***‐(**5**‐**{**[(**4**‐**Fluorophenyl**)**amino**]**methyl}**‐**3**‐**methylpyridin**‐**2**‐**yl**)**butyramide** (**43 a**): The synthesis was conducted from **42 a** (450 mg, 1.23 mmol) and trimethylboroxine (702 μL of a 3.5 M solution in THF, 2.46 mmol, 2.0 equiv.) according to the general procedure (Suzuki reaction). The purification was carried out by flash chromatography (ethyl acetate/*n*‐hexane, 60–90 % ethyl acetate), yielding the product as a colourless oil. To obtain a solid, the residue was dissolved in methanol, followed by the addition of ice‐cold water to form a precipitate, which was filtered off, yielding **43 a** as a colourless solid (70 mg, 0.23 mmol, 19 %): *R*_f_=0.39 (ethyl acetate/*n*‐hexane 3 : 1); mp: 104 °C; ^1^H‐NMR (400 MHz, DMSO‐d_6_): δ(ppm)=9.87 (s, 1H), 8.21 (d, *J*=2.2 Hz, 1H), 7.60 (d, *J*=2.3 Hz, 1H), 6.97–6.80 (m, 2H), 6.69–6.49 (m, 2H), 6.13 (t, *J*=6.0 Hz, 1H), 4.21 (d, *J*=6.0 Hz, 2H), 2.29 (t, *J*=7.3 Hz, 2H), 2.12 (s, 3H), 1.74–1.49 (m, 2H), 0.92 (t, *J*=7.4 Hz, 3H); ^13^C‐NMR (100 MHz, DMSO‐d_6_): δ(ppm)=171.2, 154.4 (d, *J*=231.1 Hz), 149.1, 145.1 (d, *J*=1.6 Hz), 144.7, 138.3, 133.2, 128.3, 115.2 (d, *J*=22.0 Hz), 113.1 (d, *J*=7.3 Hz), 44.1, 37.3, 18.5, 17.7, 13.6; IR (ATR): $\tilde{\nu }$ =3256 (m, ν_N−H_), 2964 (w, ν_C−H_), 1686 (s, ν_C=O_). ESI‐HRMS (*m/z*): calcd. for [C_17_H_20_FN_3_O+H]^+^ 302.1663, found 302.1665; cpd purity (220 nm): 99 %.

***N***‐(**5**‐**{**[(**4**‐**Fluorophenyl**)**amino**]**methyl}**‐**3**‐**methylpyridin**‐**2**‐**yl**)‐**3,3**‐**dimethylbutanamide** (**43 b**): The synthesis was carried out from **42 b** (500 mg, 1.27 mmol) and trimethylboroxine (725 μL of a 3.5 M solution in THF, 2.54 mmol, 2.0 equiv.) in accordance with the general procedure (Suzuki reaction). The crude residue was further purified by flash chromatography (ethyl acetate/*n*‐hexane, 50–80 % ethyl acetate), yielding the product as a colourless oil. To obtain a solid, the residue was dissolved in methanol, followed by the addition of ice‐cold water to form a precipitate, which was filtered off, yielding **43 b** as a colourless solid (180 mg, 0.55 mmol, 43 %): *R*_f_=0.35 (ethyl acetate/*n*‐hexane 1 : 1); mp: 93 °C; ^1^H‐NMR (400 MHz, DMSO‐d_6_): δ(ppm)=9.82 (s, 1H), 8.21 (d, *J*=2.2 Hz, 1H), 7.61 (d, *J*=2.3 Hz, 1H), 6.95–6.84 (m, 2H), 6.63–6.53 (m, 2H), 6.14 (t, *J*=6.0 Hz, 1H), 4.22 (d, *J*=6.0 Hz, 2H), 2.21 (s, 2H), 2.15 (s, 3H), 1.04 (s, 9H); ^13^C‐NMR (100 MHz, DMSO‐d_6_): δ(ppm)=169.9, 154.4 (d, *J*=231.3 Hz), 149.1, 145.1 (d, *J*=1.6 Hz), 144.8, 138.3, 133.2, 128.4, 115.2 (d, *J*=21.9 Hz), 113.1 (d, *J*=7.3 Hz), 48.6, 44.1, 30.7, 29.7, 18.0; IR (ATR): $\tilde{\nu }$ =3242 (m, ν_N−H_), 2956 (w, ν_C−H_), 1660 (s, ν_C=O_), 1586 (w, δ_N−H_). ESI‐HRMS (*m/z*): calcd. for [C_19_H_24_FN_3_O+H]^+^ 330.1976, found 330.1979; cpd purity (220 nm): 99 %.

***N***‐(**3**‐**Cyclopropyl**‐**5**‐**{**[(**4**‐**fluorophenyl**)**amino**]**methyl}pyridin**‐**2**‐**yl**)**butyramide** (**43 c**): The synthesis was conducted from **42 a** (450 mg, 1.23 mmol) and cyclopropylboronic acid (211 mg, 2.46 mmol, 2.0 equiv.) according to the general procedure (Suzuki reaction). The purification was carried out by flash chromatography (ethyl acetate/*n*‐hexane, 50–90 % ethyl acetate), yielding the product as a colourless oil. To obtain a solid, the residue was dissolved in methanol, followed by the addition of ice‐cold water to form a precipitate, which was filtered off, yielding **43 c** as a colourless solid (60 mg, 0.18 mmol, 15 %): *R*_f_=0.39 (ethyl acetate/*n*‐hexane 1 : 1); mp: 127 °C; ^1^H‐NMR (400 MHz, DMSO‐d_6_): δ(ppm)=9.83 (s, 1H), 8.17 (d, *J*=2.2 Hz, 1H), 7.34 (d, *J*=2.3 Hz, 1H), 6.95–6.84 (m, 2H), 6.62–6.52 (m, 2H), 6.09 (t, *J*=6.2 Hz, 1H), 4.19 (d, *J*=6.1 Hz, 2H), 2.30 (d, *J*=7.3 Hz, 2H), 1.95–1.84 (m, 1H), 1.65–1.53 (m, 2H), 0.92 (t, *J*=7.4 Hz, 3H), 0.89–0.84 (m, 2H), 0.58–0.49 (m, 2H); ^13^C‐NMR (100 MHz, DMSO‐d_6_): δ(ppm)=171.5, 154.4 (d, *J*=231.2 Hz), 149.3, 145.0 (d, *J*=1.6 Hz), 144.3, 133.8, 133.3, 133.3, 115.2 (d, *J*=22.0 Hz), 113.2 (d, *J*=7.3 Hz), 44.2, 37.3, 18.6, 13.7, 11.1, 7.8; IR (ATR): $\tilde{\nu }$ =3262 (m, ν_N−H_), 3002, 2962 (w, ν_C−H_), 1666 (s, ν_C=O_), 1584 (m, δ_N−H_). ESI‐HRMS (*m/z*): calcd. for [C_19_H_22_FN_3_O+H]^+^ 328.1820, found 328.1817; cpd purity (220 nm): 100 %.

***N***‐(**5**‐**{**[(**4**‐**Fluorophenyl**)**amino**]**methyl}pyridin**‐**2**‐**yl**)**butyramide** (**44**): Compound **44** was a side product in the synthesis of **43 a**. The separation from the main product by flash chromatography yielded **44** as a colourless solid (25 mg, 0.09 mmol, 7 %): *R*_f_=0.54 (ethyl acetate/*n*‐hexane 1 : 1); mp: 92 °C; ^1^H‐NMR (400 MHz, DMSO‐d_6_): δ(ppm)=10.37 (s, 1H), 8.28 (d, *J*=2.5 Hz, 1H), 8.04 (d, *J*=8.5 Hz, 1H), 7.72 (dd, *J*=8.5, 2.4 Hz, 1H), 6.95–6.84 (m, 2H), 6.62–6.52 (m, 2H), 6.10 (t, *J*=6.1 Hz, 1H), 4.19 (d, *J*=6.0 Hz, 2H), 2.34 (t, *J*=7.3 Hz, 2H), 1.66–1.54 (m, 2H), 0.89 (t, *J*=7.4 Hz, 3H); ^13^C‐NMR (100 MHz, DMSO‐d_6_): δ(ppm)=171.9, 154.4 (d, *J*=231.1 Hz), 151.0, 146.9, 145.1 (d, *J*=1.5 Hz), 137.3, 130.5, 115.2 (d, *J*=21.9 Hz), 113.2 (d, *J*=7.3 Hz), 113.0, 44.2, 37.9, 18.4, 13.5; IR (ATR): $\tilde{\nu }$ =3307 (m, ν_N−H_), 2965 (w, ν_C−H_), 1672 (s, ν_C=O_), 1578 (m, δ_N−H_).

**4**‐(**1,3**‐**Dioxoisoindolin**‐**2**‐**yl**)‐**3**‐**methylbenzonitrile** (**45**): Compound **32 b** (460 mg, 3.48 mmol) and phthalic anhydride (523 mg, 3.48 mmol, 1.0 equiv.) were suspended in acetic acid (20 mL). The mixture was stirred at 130 °C. After 5 h, the reaction was poured on ice water. The precipitate was filtered off and recrystallized from methanol to obtain **45** as an off‐white solid (756 mg, 2.88 mmol, 83 %): *R*_f_=0.76 (ethyl acetate/*n*‐hexane 1 : 1); mp: 211 °C; ^1^H‐NMR (400 MHz, DMSO‐d_6_): δ(ppm)=8.05–7.98 (m, 2H), 7.98–7.89 (m, 3H), 7.85 (ddd, *J*=8.1, 2.0, 0.7 Hz, 1H), 7.63 (d, *J*=8.1 Hz, 1H), 2.21 (s, 3H); ^13^C‐NMR (100 MHz, DMSO‐d_6_): δ(ppm)=166.4, 138.3, 135.5, 134.9, 134.5, 131.7, 130.5, 130.4, 123.7, 118.3, 111.8, 17.3; IR (ATR): $\tilde{\nu }$ =3074, 2926 (w, ν_C−H_), 2233 (m, ν_C≡N_), 1719 (s, ν_C=O_).

**4**‐(**1,3**‐**Dioxoisoindolin**‐**2**‐**yl**)‐**3**‐**methylbenzaldehyde** (**46**): The synthesis was conducted from **45** (3.30 g, 12.6 mmol) according to the general procedure (nitrile reduction). The purification was carried out by silica gel column chromatography (ethyl acetate/*n*‐hexane 2 : 3) to obtain **46** as a colourless solid (2.71 g, 10.2 mmol, 81 %): *R*_f_=0.72 (ethyl acetate/*n*‐hexane 1 : 1); mp: 188 °C; ^1^H‐NMR (400 MHz, DMSO‐d_6_): δ(ppm)=10.06 (s, 1H), 8.03–7.99 (m, 2H), 7.98–7.92 (m, 3H), 7.91–7.87 (m, 1H), 7.63 (d, *J*=8.0 Hz, 1H), 2.25 (s, 3H); ^13^C‐NMR (100 MHz, DMSO‐d_6_): δ(ppm)=192.6, 166.6, 137.6, 136.4, 136.3, 134.9, 131.7, 131.6, 130.1, 127.5, 123.7, 17.5; IR (ATR): $\tilde{\nu }$ =3027, 2976 (w, ν_C−H_), 1704, 1681 (s, ν_C=O_).

***N***‐(**4**‐**{**[(**4**‐**Fluorophenyl**)**amino**]**methyl}**‐**2**‐**methylphenyl**)‐**2**‐(**hydroxymethyl**)**benzamide** (**48**): The synthesis was carried out from **46** (1.20 g, 4.5 mmol) and 4‐fluoroaniline (520 μL, 5.43 mmol, 1.2 equiv.) in accordance with the general procedure (reductive amination). The crude residue was further purified by silica gel column chromatography (ethyl acetate/*n*‐hexane 2 : 3) and subsequent recrystallization (methanol/water), which yielded **48** as a colourless solid (1.12 g, 3.1 mmol, 68 %): *R*_f_=0.72 (ethyl acetate/*n*‐hexane 3 : 2); mp: 144 °C; ^1^H‐NMR (400 MHz, DMSO‐d_6_): δ(ppm)=9.82 (s, 1H), 7.63–7.55 (m, 2H), 7.53–7.45 (m, 1H), 7.42–7.33 (m, 2H), 7.24 (d, *J*=2.0 Hz, 1H), 7.18 (dd, *J*=8.3, 2.1 Hz, 1H), 6.94–6.83 (m, 2H), 6.60–6.50 (m, 2H), 6.14 (t, *J*=6.0 Hz, 1H), 5.36 (t, *J*=5.5 Hz, 1H), 4.69 (d, *J*=5.5 Hz, 2H), 4.19 (d, *J*=5.9 Hz, 2H), 2.24 (s, 3H); ^13^C‐NMR (100 MHz, DMSO‐d_6_): δ(ppm)=167.3, 154.3 (d, *J*=230.9 Hz), 145.3, 140.2, 137.5, 135.2, 134.8, 132.7, 129.9, 129.1, 127.6, 127.6, 126.7, 125.9, 124.8, 115.2 (d, *J*=22.0 Hz), 113.0 (d, *J*=7.3 Hz), 61.0, 46.7, 18.1; IR (ATR): $\tilde{\nu }$ =3000–3300 (b, ν_O‐H_), 3267 (m, ν_N−H_), 3036, 2907 (w, ν_C−H_), 1645 (s, ν_C=O_); ESI‐HRMS (*m/z*): calcd. for [C_22_H_22_N_2_O_2_F+H]^+^ 365.1660, found 365.1658; cpd purity (220 nm): 100 %.

**2**‐(**4**‐**{**[(**4**‐**Fluorophenyl**)**amino**]**methyl}**‐**2**‐**methylphenyl**)**isoindoline**‐**1,3**‐**dione** (**49**): Compound **46** (1.64 g, 6.2 mmol) was suspended in dry toluene (50 mL). 4 Å molecular sieves (5 g), and 4‐fluoroaniline (713 μL, 7.43 mmol, 1.2 equiv.) were added. The reaction mixture was stirred at 120 °C. After 6 h, the reaction was terminated, and the mixture was filtered hot to remove the molecular sieves. The filtrate was cooled to room temperature and concentrated under reduced pressure. The residue was dissolved in ethyl acetate (50 mL). Pd/C (10 % Pd, 50 % water wet, 300 mg) was added, and the mixture was carefully set under a hydrogen atmosphere (balloon pressure). After the reaction stirred for 6 h at room temperature, the catalyst was filtered off, and the filtrate was concentrated under reduced pressure. The crude residue was purified by silica gel column chromatography (ethyl acetate/*n*‐hexane 3 : 7) and subsequent recrystallization (toluene/*n*‐hexane) to obtain **49** as a slightly yellow coloured solid (1.49 g, 4.1 mmol, 67 %): *R*_f_=0.81 (ethyl acetate/*n*‐hexane 1 : 1); mp: 156 °C; ^1^H‐NMR (400 MHz, DMSO‐d_6_): δ(ppm)=8.01–7.94 (m, 2H), 7.94–7.87 (m, 2H), 7.40–7.34 (m, 1H), 7.33–7.23 (m, 2H), 6.94–6.85 (m, 2H), 6.61–6.53 (m, 2H), 6.21 (t, *J*=6.0 Hz, 1H), 4.25 (d, *J*=6.0 Hz, 2H), 2.09 (s, 3H); ^13^C‐NMR (100 MHz, DMSO‐d_6_): δ(ppm)=167.1, 154.3 (d, *J*=231.0 Hz), 145.3 (d, *J*=1.3 Hz), 141.3, 136.1, 134.7, 131.6, 129.4, 129.3, 129.1, 125.4, 123.5, 115.2 (d, *J*=22.0 Hz), 112.9 (d, *J*=7.3 Hz), 46.6, 17.5; IR (ATR): $\tilde{\nu }$ =3399 (m, ν_N−H_), 2976 (w, ν_C−H_), 1720 (s, ν_C=O_); 1610 (m, δ_N−H_).

**Benzyl** [**4**‐(**1,3**‐**dioxoisoindolin**‐**2**‐**yl**)‐**3**‐**methylbenzyl**](**4**‐**fluorophenyl**)**carbamate** (**50**): Compound **49** (3.00 g, 8.3 mmol) was dissolved in DCM (200 mL). DIPEA (2.90 mL, 16.7 mmol, 2.0 equiv.) and benzyl chloroformate (1.46 mL, 10.4 mmol, 1.3 equiv.) were added, and the resulting solution was stirred at room temperature. After 4 h, the reaction mixture was concentrated under reduced pressure. The residue was purified by flash chromatography (ethyl acetate/*n*‐hexane, 30–50 % ethyl acetate) to obtain **50** as an off‐white solid (3.46 g, 7.0 mmol, 84 %): *R*_f_=0.40 (ethyl acetate/*n*‐hexane 1 : 3); mp: 150 °C; ^1^H‐NMR (400 MHz, DMSO‐d_6_): δ(ppm)=8.01–7.95 (m, 2H), 7.95–7.89 (m, 2H), 7.42–7.14 (m, 12H), 5.16 (s, 2H), 4.92 (s, 2H), 2.07 (s, 3H); ^13^C‐NMR (100 MHz, DMSO‐d_6_): δ(ppm)=167.0, 160.1 (d, *J*=243.7 Hz), 154.9, 138.7, 138.0, 136.5, 136.3, 134.7, 131.6, 129.8, 129.3, 128.7, 128.6 (d, *J*=8.9 Hz), 128.4, 127.8, 127.4, 125.3, 123.5, 115.6 (d, *J*=22.5 Hz), 66.9, 53.1, 17.47; IR (ATR): $\tilde{\nu }$ =3031, 2973 (w, ν_C−H_), 1695 (s, ν_C=O_).

**Benzyl** (**4**‐**amino**‐**3**‐**methylbenzyl**)(**4**‐**fluorophenyl**)**carbamate** (**51**): Compound **50** (3.40 g, 6.9 mmol) was dissolved in THF (100 mL). An 80 % hydrazine hydrate solution (8.37 mL, 137.5 mmol, 20.0 equiv.) was added, and the reaction mixture was stirred for 16 h at room temperature. After complete conversion, the volatiles were removed under reduced pressure. The residue was dissolved in DCM (200 mL), and insoluble solids were filtered off. The filtrate was concentrated under reduced pressure, and the crude residue was purified by silica gel column chromatography (ethyl acetate/*n*‐hexane 1 : 1), which yielded **51** as a slightly yellow oil (2.45 g, 6.7 mmol, 98 %): *R*_f_=0.75 (ethyl acetate/*n*‐hexane 1 : 1); ^1^H‐NMR (400 MHz, DMSO‐d_6_): δ(ppm)=7.39–7.24 (m, 5H), 7.19–7.07 (m, 4H), 6.73 (d, *J*=2.4 Hz, 1H), 6.66 (dd, *J*=8.1, 2.1 Hz, 1H), 6.48 (d, *J*=8.0 Hz, 1H), 5.12 (s, 2H), 4.75 (s, 2H), 4.64 (s, 2H), 1.96 (s, 3H); ^13^C‐NMR (100 MHz, DMSO‐d_6_): δ(ppm)=160.1 (*J*=243.2 Hz), 154.8, 145.8, 137.9, 136.7, 129.6, 129.2 (d, *J*=8.4 Hz), 128.3, 127.8, 127.4, 126.1, 124.6, 120.9, 115.4 (d, *J*=22.5 Hz), 113.8, 66.6, 53.2, 17.4; IR (ATR): $\tilde{\nu }$ =3367 (m, ν_N−H_), 3032, 2930 (w, ν_C−H_), 1692 (s, ν_C=O_); 1626 (m, δ_N−H_).

**Benzyl {4**‐[**3**‐(**1*H***‐**pyrazol**‐**1**‐**yl**)**propanamido**]‐**3**‐**methylbenzyl}**(**4**‐**fluorophenyl**)**carbamate** (**52**): Compound **58** (423 mg, 3.02 mmol, 2.0 equiv.) was dissolved in DMF (10 mL). HOBt (510 mg, 3.77 mmol, 2.5 equiv.), DIC (591 μL, 3.77 mmol, 2.5 equiv.) and **51** (550 mg, 1.51 mmol) were added successively. The reaction mixture was stirred at room temperature. After 16 h, the solution was partitioned between ethyl acetate (100 mL) and water (100 mL). The organic phase was extracted with water (2×100 mL), washed with brine, dried over Na_2_SO_4_, filtrated, and concentrated under reduced pressure. The crude residue was purified by silica gel column chromatography (ethyl acetate/*n*‐hexane 9 : 1). NMR analysis of the obtained product revealed that it contained a significant amount of diisopropyl urea as a side product of the coupling reaction. To remove the diisopropyl urea, the crude product was suspended in 30 mL of diethyl ether. Solids were filtered off, and the filtrate was concentrated under reduced pressure to obtain the product as a colourless solid. NMR analysis showed a reduction of the diisopropyl urea by a factor of 10, but the product still contained about 25 mol% (8 wt%) of diisopropyl urea based on NMR analysis. Nevertheless, the obtained product (550 mg in total containing 506 mg of the desired product, 1.04 mmol, 69 %) was used for the following reaction without further purification. ^1^H‐NMR (400 MHz, DMSO‐d_6_): δ(ppm)=9.29 (s, 1H), 7.67 (d, *J*=2.3 Hz, 1H), 7.44 (d, *J*=2.0 Hz, 1H), 7.38–7.20 (m, 8H), 7.19–7.09 (m, 2H), 6.99 (d, *J*=2.1 Hz, 1H), 6.95 (dd, *J*=8.1, 2.1 Hz, 1H), 6.21 (t, *J*=2.0 Hz, 1H), 5.13 (s, 2H), 4.79 (s, 2H), 4.40 (t, *J*=6.8 Hz, 2H), 2.88 (t, *J*=6.8 Hz, 2H), 2.04 (s, 3H); ^13^C‐NMR (100 MHz, DMSO‐d_6_): δ(ppm)=168.5, 160.1 (d, *J*=242.8 Hz), 154.9, 138.6, 137.8, 136.6, 135.2, 134.3, 131.7, 129.9, 129.3, 128.9, 128.3, 127.8, 127.4, 125.0, 124.9, 115.5 (d, *J*=22.6 Hz), 104.9, 66.8, 53.0, 47.4, 36.4, 17.8.

***N***‐(**4**‐**{**[(**4**‐**Fluorophenyl**)**amino**]**methyl}**‐**2**‐**methylphenyl**)‐**3**‐(**1*H***‐**pyrazol**‐**1**‐**yl**)**propanamide** (**53**): Compound **52** (417 mg, 0.86 mmol) was suspended in a 33 wt% solution of HBr in acetic acid (10 mL) and stirred at room temperature in a sealed vessel. After 2 h, the reaction mixture was poured into diethyl ether (100 mL). The resulting precipitate was filtered off. The colourless solid was washed with diethyl ether, and afterwards, partitioned between ethyl acetate (100 mL) and a saturated aq. solution of NaHCO_3_ (100 mL). The organic phase was washed with brine, dried over Na_2_SO_4_, filtrated, and concentrated under reduced pressure. The crude residue was purified by silica gel column chromatography (ethyl acetate/*n*‐hexane 9 : 1) and subsequent recrystallization (methanol/water) to obtain **53** as a colourless solid (270 mg, 0.77 mmol, 89 %): *R*_f_=0.39 (ethyl acetate/*n*‐hexane 3 : 1); mp: 183 °C; ^1^H‐NMR (400 MHz, DMSO‐d_6_): δ(ppm)=9.30 (s, 1H), 7.67 (d, *J*=2.2 Hz, 1H), 7.44 (d, *J*=1.8 Hz, 1H), 7.24 (d, *J*=8.1 Hz, 1H), 7.16 (d, *J*=2.1 Hz, 1H), 7.11 (dd, *J*=8.1, 2.1 Hz, 1H), 6.92–6.81 (m, 2H), 6.58–6.48 (m, 2H), 6.21 (t, *J*=2.0 Hz, 1H), 6.09 (t, *J*=6.0 Hz, 1H), 4.40 (t, *J*=6.8 Hz, 2H), 4.14 (d, *J*=5.9 Hz, 2H), 2.87 (t, *J*=6.8 Hz, 2H), 2.07 (s, 3H); ^13^C‐NMR (100 MHz, DMSO‐d_6_): δ(ppm)=168.5, 154.2 (d, *J*=230.7 Hz), 145.4, 138.6, 136.9, 134.7, 131.8, 129.9, 129.0, 125.1, 124.7, 115.2 (d, *J*=22.0 Hz), 112.9 (d, *J*=7.3 Hz), 104.9, 47.5, 46.6, 36.4, 17.8; IR (ATR): $\tilde{\nu }$ =3371, 3171 (m, ν_N−H_), 3108, 2991 (w, ν_C−H_), 1670 (s, ν_C=O_); 1612 (m, δ_N−H_); ESI‐HRMS (*m/z*): calcd. for [C_20_H_21_FN_4_O+H]^+^ 353.1771, found 353.1775; cpd purity (220 nm): 100 %.

***N***‐(**2,4**‐**Dimethylphenyl**)‐**3**‐(**1*H***‐**pyrazol**‐**1**‐**yl**)**propanamide** (**54**): Compound **52** (280 mg, 0.58 mmol) was dissolved in ethyl acetate (25 mL). Pd/C (10 % Pd, 50 % water wet, 50 mg) was added, the suspension was carefully set under a hydrogen atmosphere (balloon pressure) and stirred at room temperature. After 20 min, the reaction mixture was filtered through a pad of celite, and the filtrate was concentrated under reduced pressure. The crude residue was purified by silica gel column chromatography (ethyl acetate/*n*‐hexane 9 : 1) and subsequent recrystallization (water), which yielded **54** as a colourless solid (86 mg, 0.35 mmol, 61 %): *R*_f_=0.38 (ethyl acetate/*n*‐hexane 3 : 1); mp: 118 °C; ^1^H‐NMR (400 MHz, DMSO‐d_6_): δ(ppm)=9.27 (s, 1H), 7.67 (d, *J*=1.6 Hz, 1H), 7.44 (d, *J*=1.1 Hz, 1H), 7.17 (d, *J*=8.0 Hz, 1H), 6.98 (d, *J*=2.0 Hz, 1H), 6.93 (dd, *J*=8.0, 2.1 Hz, 1H), 6.21 (t, *J*=2.0 Hz, 1H), 4.40 (t, *J*=6.8 Hz, 2H), 2.87 (t, *J*=6.8 Hz, 2H), 2.23 (s, 3H), 2.05 (s, 3H); ^13^C‐NMR (100 MHz, DMSO‐d_6_): δ(ppm)=168.5, 138.6, 134.2, 133.5, 131.8, 130.7, 129.9, 126.3, 125.2, 104.9, 47.5, 36.4, 20.4, 17.7; IR (ATR): $\tilde{\nu }$ =3293 (m, ν_N−H_), 3021, 2957 (w, ν_C−H_), 1651 (s, ν_C=O_); 1590 (m, δ_N−H_).

**Methyl 3**‐(**1*H***‐**pyrazol**‐**1**‐**yl**)**propanoate** (**57**): Methyl prop‐2‐enate (2.27 mL, 25.0 mmol), pyrazole (1.70 g, 25.0 mmol, 1.0 equiv.) and DBU (3.73 mL, 25.0 mmol, 1.0 equiv.) were dissolved in acetonitrile (25 mL). The reaction mixture was stirred at room temperature. After 16 h, the volatiles were removed under reduced pressure, and the residue was purified by silica gel column chromatography (ethyl acetate/*n*‐hexane 3 : 7) to obtain **57** as a colourless oil (2.93 g, 19.0 mmol, 76 %): *R*_f_=0.46 (ethyl acetate/*n*‐hexane 3 : 7); ^1^H‐NMR (400 MHz, DMSO‐d_6_): δ(ppm)=7.69 (dd, *J*=2.3, 0.8 Hz, 1H), 7.42 (dd, *J*=1.9, 0.7 Hz, 1H), 6.20 (t, *J*=2.1 Hz, 1H), 4.35 (t, *J*=6.7 Hz, 2H), 3.59 (s, 3H), 2.86 (t, *J*=6.7 Hz, 2H); ^13^C‐NMR (100 MHz, DMSO‐d_6_): δ(ppm)=171.1, 138.7, 130.0, 105.0, 51.5, 46.7, 34.2; IR (ATR): $\tilde{\nu }$ =2954 (w, ν_C−H_), 1732 (s, ν_C=O_).

**3**‐(**1*H***‐**Pyrazol**‐**1**‐**yl**)**propanoic acid** (**58**): Compound **57** (2.72 g, 17.7 mmol) was dissolved in methanol (30 mL). A solution of potassium hydroxide (3.89 g, 70.6 mmol, 4.0 equiv.) in water (10 mL) was added, and the solution was stirred at room temperature. After 16 h, the reaction mixture was acidified to pH 1–2 with conc. aq. HCl and subsequently concentrated under reduced pressure. The residue was treated with diethyl ether several times and remaining solids were filtered off. The resulting filtrate was concentrated under reduced pressure again to obtain **58** as a colourless solid (1.83 g, 13.1 mmol, 74 %): *R*_f_=0.49 (ethyl acetate/toluene/AcOH 5 : 5 : 1); mp: 67 °C; ^1^H‐NMR (400 MHz, DMSO‐d_6_): δ(ppm)=11.14 (s, 1H), 7.69 (dd, *J*=2.3, 0.7 Hz, 1H), 7.43 (dd, *J*=1.8, 0.7 Hz, 1H), 6.21 (t, *J*=2.1 Hz, 1H), 4.31 (t, *J*=6.8 Hz, 2H), 2.77 (t, *J*=6.8 Hz, 2H); ^13^C‐NMR (100 MHz, DMSO‐d_6_): δ(ppm)=172.2, 138.6, 130.1, 105.0, 46.9, 34.5; IR (ATR): $\tilde{\nu }$ =3300–2500 (b, ν_O‐H_), 3120, 2971 (w, ν_C−H_), 1712 (s, ν_C=O_).

**Benzyl** (**4**‐**acrylamido**‐**3**‐**methylbenzyl**)(**4**‐**fluorophenyl**)**carbamate** (**59**): Compound **51** (270 mg, 0.74 mmol) was dissolved in DCM (25 mL). DIPEA (258 μL, 1.48 mmol, 2.0 equiv.) was added in one portion. The reaction mixture was cooled to 0 °C, and a solution of prop‐2‐enoyl chloride (91 μL, 1.11 mmol, 1.5 equiv.) in DCM (2 mL) was added dropwise over a period of 30 min. Subsequently, stirring at 0 °C was continued. After 2 h, the reaction mixture was concentrated under reduced pressure, and the crude residue was purified by flash chromatography (ethyl acetate/*n*‐hexane, 30–60 % ethyl acetate) to obtain **59** as a colourless oil (290 mg, 0.69 mmol, 94 %): *R*_f_=0.30 (ethyl acetate/*n*‐hexane 3 : 7); ^1^H‐NMR (400 MHz, DMSO‐d_6_): δ(ppm)=9.42 (s, 1H), 7.41 (d, *J*=8.1 Hz, 1H), 7.38–7.20 (m, 7H), 7.20–7.10 (m, 2H), 7.04 (d, *J*=2.1 Hz, 1H), 6.99 (dd, *J*=8.1, 2.1 Hz, 1H), 6.52 (dd, *J*=17.0, 10.2 Hz, 1H), 6.23 (dd, *J*=17.1, 2.0 Hz, 1H), 5.73 (dd, *J*=10.2, 2.1 Hz, 1H), 5.14 (s, 2H), 4.81 (s, 2H), 2.14 (s, 3H); ^13^C‐NMR (100 MHz, DMSO‐d_6_): δ(ppm)=163.2, 160.1 (d, *J*=243.5 Hz), 154.9, 137.9, 136.6, 135.1, 134.4, 131.7, 131.5, 129.4, 128.9, 128.4, 127.8, 127.4, 126.5, 125.1, 124.8, 115.5 (d, *J*=22.6 Hz), 66.8, 53.0, 17.9; IR (ATR): $\tilde{\nu }$ =3271 (m, ν_N−H_), 3031, 2953 (w, ν_C−H_), 1687, 1663 (s, ν_C=O_).

**Benzyl** (**4**‐**fluorophenyl**)**{3**‐**methyl**‐**4**‐[**3**‐(**pyrrolidin**‐**1**‐**yl**)**propanamido**]**benzyl}carbamate** (**60**): Compound **59** (280 mg, 0.67 mmol) was dissolved in ethanol (25 mL). Pyrrolidine (98 μL, 1.34 mmol, 2.0 equiv.) was added in one portion, and the reaction mixture was stirred at 60 °C. After 7 h, the solution was concentrated under reduced pressure, and the crude residue was purified by silica gel column chromatography (DCM/methanol 95 : 5) to obtain **60** as a colourless oil (285 mg, 0.58 mmol, 87 %): *R*_f_=0.73 (DCM/methanol 9 : 1); ^1^H‐NMR (400 MHz, DMSO‐d_6_): δ(ppm)=9.98 (s, 1H), 7.64 (d, *J*=8.2 Hz, 1H), 7.39–7.18 (m, 7H), 7.18–7.09 (m, 2H), 7.00 (d, *J*=2.1 Hz, 1H), 6.95 (dd, *J*=8.2, 2.1 Hz, 1H), 5.14 (s, 2H), 4.78 (s, 2H), 2.72 (t, *J*=6.5 Hz, 2H), 2.61–2.41 (m, 6H), 2.12 (s, 3H), 1.79–1.65 (m, 4H); ^13^C‐NMR (100 MHz, DMSO‐d_6_): δ(ppm)=170.2, 160.1 (d, *J*=243.4 Hz), 154.9, 137.8, 136.6, 135.9, 133.1, 129.3, 129.2, 129.0, 128.4, 127.8, 127.4, 125.2, 122.8, 115.5 (d, *J*=22.6 Hz), 66.8, 53.2, 52.9, 51.5, 35.2, 23.1, 17.6; IR (ATR): $\tilde{\nu }$ =1698 (s, ν_C=O_).

***N***‐(**4**‐**{**[(**4**‐**Fluorophenyl**)**amino**]**methyl}**‐**2**‐**methylphenyl**)‐**3**‐(**pyrrolidin**‐**1**‐**yl**)**propanamide 2,2,2**‐**trifluoroacetate** (**61**): Compound **60** (480 mg, 0.98 mmol) was dissolved in methanol (25 mL). Pd/C (10 % Pd, 50 % water wet, 200 mg) was added, the suspension was carefully set under a hydrogen atmosphere (balloon pressure) and stirred at room temperature. After 2 h, the reaction mixture was filtered through a pad of celite. The filtrate was concentrated under reduced pressure. The crude residue was purified by silica gel column chromatography (DCM/methanol 95 : 5) and successive preparative HPLC (Hibar RT 250–25 column, mobile phase methanol/water 1 : 1 with 0.1 % TFA). The title compound was obtained as a colourless solid, which turned into an orange oil after contact with air (230 mg, 0.49 mmol, 50 %): ^1^H‐NMR (400 MHz, MeOH‐d_4_): δ(ppm)=7.55 (d, *J*=8.2 Hz, 1H), 7.20–7.35 (m, 7H), 4.52 (s, 2H), 3.78–3.68 (m, 2H), 3.58 (t, *J*=6.8 Hz, 2H), 3.24–3.11 (m, 2H), 3.30 (t, *J*=6.8 Hz, 2H), 2.31 (s, 3H), 2.23–2.14 (m, 2H), 2.14–2.03 (m, 2H).

### LogD_7.4_ estimation

The log*D*_7.4_ estimation was carried out as previously reported by means of a standard HPLC‐based method, which was established in accordance with guideline OPPTS 830.7570 of the United States Environmental Protection Agency.^[43]^ Briefly, the HPLC analysis was performed with a Phenomenex Luna 5 μm Phenyl‐Hexyl 100 Å column (150×4.6 mm) as stationary phase and a mixture of methanol (75 %) and 10 mM Tris/HCl buffer (25 %) at pH 7.4 as mobile phase (flow rate 1.0 mL/min). In the first step, the capacity factors of seven reference substances were determined from their retention times (acetophenone, benzene, ethyl benzoate, benzophenone, phenyl benzoate, diphenyl ether, bibenzyl) with uracil used as a dead‐time marker. The reference substances were taken from a list of recommended standards included in the above‐mentioned guideline and cover the required log*D*_7.4_ range of 1.7–4.8. The logarithm of the capacity factors of the reference substances was then plotted against the corresponding log*P* values taken from the mentioned guideline to obtain a calibration function. For non‐ionizable compounds like the reference substances used for calibration, log*P* values can be considered equivalent to the log*D*_7.4_ values. To obtain a reference mixture, 2 mg of the reference substances were each dissolved in 1 mL of methanol, and 50 μL aliquots of each reference solution were subsequently combined. The reference mixture was measured before and after the substances to be tested, and the mean retention time from both measurements was used to calculate the calibration function. The calibration function was then used to determine the log*D*_7.4_ values from the corresponding retention times for each substance to be tested. The compounds of interest were injected as a solution in methanol (2 mg/mL), and the retention time was determined as the mean value from two measurements.

### Cyclic Voltammetry

The cyclic voltammetry measurements were carried out as described elsewhere.[^24^, ^33^] Briefly, a 797 VA Computrace device from Metrohm was used with a three‐electrode cell setup consisting of a glassy carbon working electrode, a platinum auxiliary electrode, and an Ag/AgCl reference electrode. To prepare the test solutions, 2 mg of each substance were suspended in 10 mL of a 0.1 M Tris‐HCl buffer (pH 7.4) and dissolved under ultrasonic treatment. If a substance did not dissolve completely, the insoluble residues were separated. Before each measurement, the cell containing the test solution was flushed with nitrogen for 150 s. The cyclic voltammograms were recorded in a potential range from −0.5 to 1.0 V with 5 mV voltage steps and a sweep rate of 0.1 V/s. After each measurement, the glassy carbon working electrode was cleaned by using an aluminum oxide suspension and a polishing cloth.

### Evaluation of K_V_7.2/3 channel opening activity

The FLIPR Potassium Assay Kit (Molecular Devices, CA, USA) was used to determine the K_V_7.2/3 channel opening activity of the test compounds in accordance with the manufacturer‘s instructions. The culturing of the HEK‐293 cells transfected with KCNQ2/3 (SB Drug Discovery, Glasgow, UK) and the data processing were carried out as described in earlier work.[^24^, ^33^] In short, 60,000 cells per well were seeded into black‐walled 96‐well plates with a clear bottom (4titude Vision Plates from Azenta Life Sciences). After incubation for 24 h, 100 μL of loading buffer containing 5 mM probenecid was added to obtain a total volume of 200 μL. Subsequently, the plates were incubated in the dark for 1 h at room temperature. A serial dilution of the test compounds in DMSO was prepared, added to the wells, and incubation was continued for 30 min. Control wells contained the loading buffer with a DMSO concentration corresponding to the substance samples (1 % (v/v)). Fluorimetric measurements were conducted at extinction/emission wavelengths of 485 nm and 535 nm, respectively, with an Infinite F200 Pro plate reader (Tecan). The background fluorescence (baseline) was measured for 20 s. Subsequently, a stimulus buffer (25 mM K^+^, 15 mM Tl^+^) was added to each well, and the fluorescence intensity was recorded for 2.5 min. The measured fluorescence intensity changes were normalized by dividing with the average baseline signal (F/F_0_) at each time point of signal acquisition. A correction was then performed by determining the difference between the normalized control signal and the normalized baseline signal (average value of 1) and subtracting the result from the F/F_0_ value at each time point at a given concentration (corr. ▵F/F_0_). To obtain a dose‐response curve, the maximal corr. ΔF/F_0_ values were plotted against the logarithmic compound concentration. The EC_50_ value for each compound was calculated as a relative value with GraphPad Prism 6 (La Jolla, CA, USA) by determining the inflexion point of the sigmoidal curve. The corresponding E_max_ value indicated the intrinsic activity of a compound and was determined relative to flupirtine by defining the maximum corr. ΔF/F_0_ value of flupirtine as 100 %. The EC_50_ and E_max_ values are the means of at least three independent experiments±standard deviation (SD).

### Hepatic Cell Viability Assay

The cell culture of the TAMH and HEP−G2 cells and the MTT assay used to evaluate the cell viability were performed as previously described in detail.[^24^, ^33^] Briefly, for the TAMH mouse liver cell line, 20,000 cells/well grown in serum‐free DMEM/F12 medium supplemented with 5 % PANEXIN NTA, 10 mM nicotinamide and 10 μg/mL gentamicin sulfate were seeded into 96 well plates. In the case of the HEP−G2 human hepatoma cell line, 15,000 cells/well grown in RPMI 1640 (PAN Biotech), supplemented with 10 % heat‐inactivated fetal bovine serum and 1 % penicillin/streptomycin, were seeded into 96‐well plates. For both cell lines, the following incubation was carried out at 37 °C in 5 % CO_2_ atmosphere for 24 h. Solutions of test compounds in DMSO were serially diluted into the corresponding culture medium to obtain 5–9 compound concentrations with 1 % DMSO (v/v). The resulting serial dilutions were used to replace the medium in the wells. Additional wells were used for control and contained the same number of cells and 1 % (v/v) DMSO without test compounds. To determine the background optical density (OD), wells without cells were used, which were treated analogously to the control wells. After 24 h of incubation, the respective medium was removed and fresh medium supplemented with 10 % (v/v) of a 2.5 mg/mL solution of 3‐(4,5‐dimethylthiazol‐2‐yl)‐2,5‐diphenyltetrazolium bromide (MTT) was added. After an additional 4 h of incubation, the culture medium in each well was carefully replaced with 50 μL of DMSO to dissolve the formazan crystals. Subsequently, ODs were measured at 570 nm using a SpectraMax 190 microplate reader. To determine the cell viability, the ODs of the test compound (T) and control (C) wells were corrected by subtracting the bank value. The calculated T/C_corr._‐ratios were then plotted against the logarithmic compound concentration. The data analysis was performed by using GraphPad Prism 6. After interpolation of a sigmoidal standard curve the LD_50_ and LD_25_ values represented the concentrations that reduced cell viability to 50 or 75 %, respectively. If no reduction of cell viability to 75 % was observed at the highest possible concentration, the LD_25_ was reported as higher than the highest tested concentration. The determined LD_50_ and LD_25_ values are the means of at least three independent experiments ± standard deviation (SD).

### Molecular docking

The heterotetrameric structure of the K_V_7.2/3 potassium channel was taken from an in‐house library and is published elsewhere.^[43]^ In brief, the prepared structure of the homotetrameric K_V_7.2 potassium channel with retigabine (PDB 7CR2) was used as a template for an energy‐based homology modelling within the Multiple Sequence Viewer in Maestro (Schrödinger, LLC, NY, USA).^[39]^ All compounds were prepared by the Ligand Preparation tool and docked with Glide (version 94137) by using an induced fit approach (standard sampling protocol, 20 protein conformations per ligand).^[56]^ The bounding box was placed onto the bound retigabine ligand structure, and an implicit membrane was applied to the transmembrane region. Finally, all ligand poses were visually compared and analyzed.

### T‐REMD simulations

The 3D structures of 1,2‐diphenylethane and *N*‐benzylaniline were generated from their corresponding 2D structures and solvated in a cubic box of octan‐1‐ol with an edge length of 3.5 nm. OPLS4 force field parameters were assigned,^[57]^ and all simulations were performed with Desmond (version 6.9.137 with GPU support).^[58]^ Both systems were minimized for 100 ps and equilibrated by using a standard relaxation protocol provided by Schrodinger, followed by 5 ns of NPT simulation at a temperature of 300 K, 1 atm pressure and a timestep of 2 ps. Temperature and pressure in all simulations were maintained by a Langevin thermostat and barostat, respectively. Short‐range van der Waals and electrostatic interaction cut‐offs were set to 1 nm, including a 0.1 nm switching function. The temperature replica exchange simulations (T‐REMD) were started from the last frame of the previous equilibration simulation and performed in an NVT ensemble for 50 ns.^[59]^ In total, eight replica were distributed on a temperature range (evenly spaced log scale) between 300 and 330 K. Exchange attempts were performed every 500 fs and snapshots were saved every 5 ps. The baseline trajectory (10.000 structures) was analyzed with custom scripts using the Schrodinger Python API to calculate ring angles and distances.

## Conflict of interest

The authors declare no conflict of interest.

1

## Supporting information

As a service to our authors and readers, this journal provides supporting information supplied by the authors. Such materials are peer reviewed and may be re‐organized for online delivery, but are not copy‐edited or typeset. Technical support issues arising from supporting information (other than missing files) should be addressed to the authors.

Supporting Information

## Data Availability

The data that support the findings of this study are available in the supplementary material of this article.
