# Supplementary material for: Carba Analogues of Flupirtine and Retigabine with Improved Oxidation Resistance and Reduced Risk of Quinoid Metabolite Formation
Source: ChemMedChem. 2022 Jul 7;17(16):e202200262. doi: 10.1002/cmdc.202200262 (PMC9541272; doi:10.1002/cmdc.202200262)
Supplement: Supplementary file 1 — Supporting Information [file CMDC-17-0-s001.pdf]

ChemMedChem

Supporting Information

Carba Analogues of Flupirtine and Retigabine with Improved Oxidation Resistance and Reduced Risk of Quinoid Metabolite Formation

Konrad W. Wurm, Frieda-Marie Bartz, Lukas Schulig, Anja Bodtke, Patrick J. Bednarski, and Andreas Link*

Table of contents

1 ^{1}H- and ^{13}C-NMR spectra 2

2 Assignement of NMR signals 64

3 Log$D_{7.4}$ estimation 86

1 ^{1}H- and ^{13}C-NMR spectra

Ethyl [2-amino-6-(4-fluorophenethyl)pyridin-3-yl]carbamate (**7**)

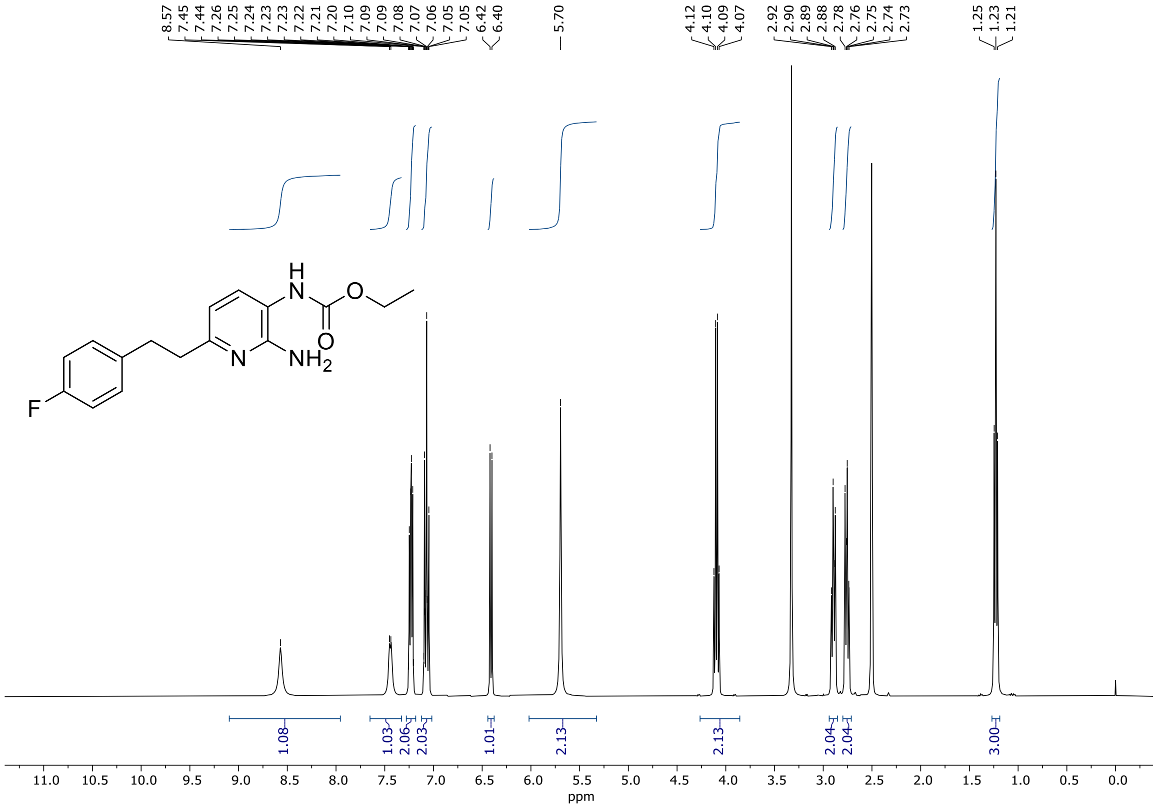

Figure S1: ^{1}H-NMR spectrum of compound **7**.

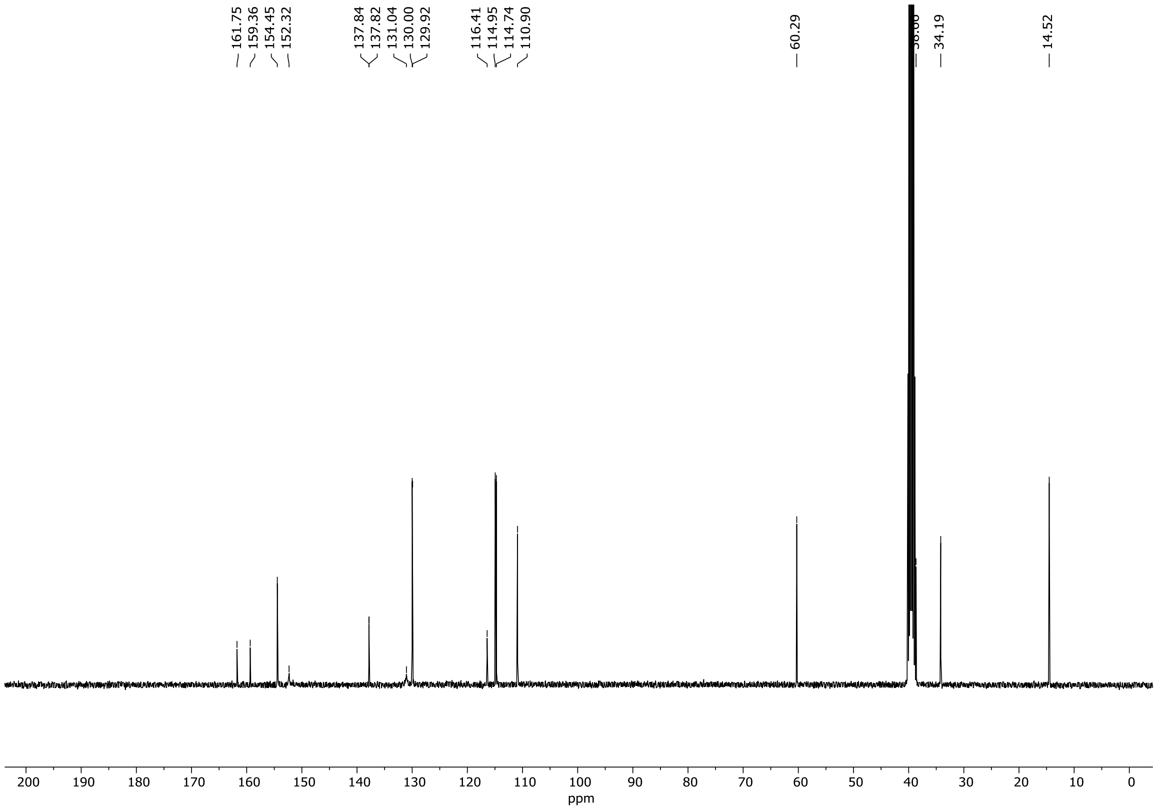

Figure S2: ^{13}C-NMR spectrum of compound **7**.

[(4-Fluorophenyl)ethynyl]trimethylsilane (**10**)

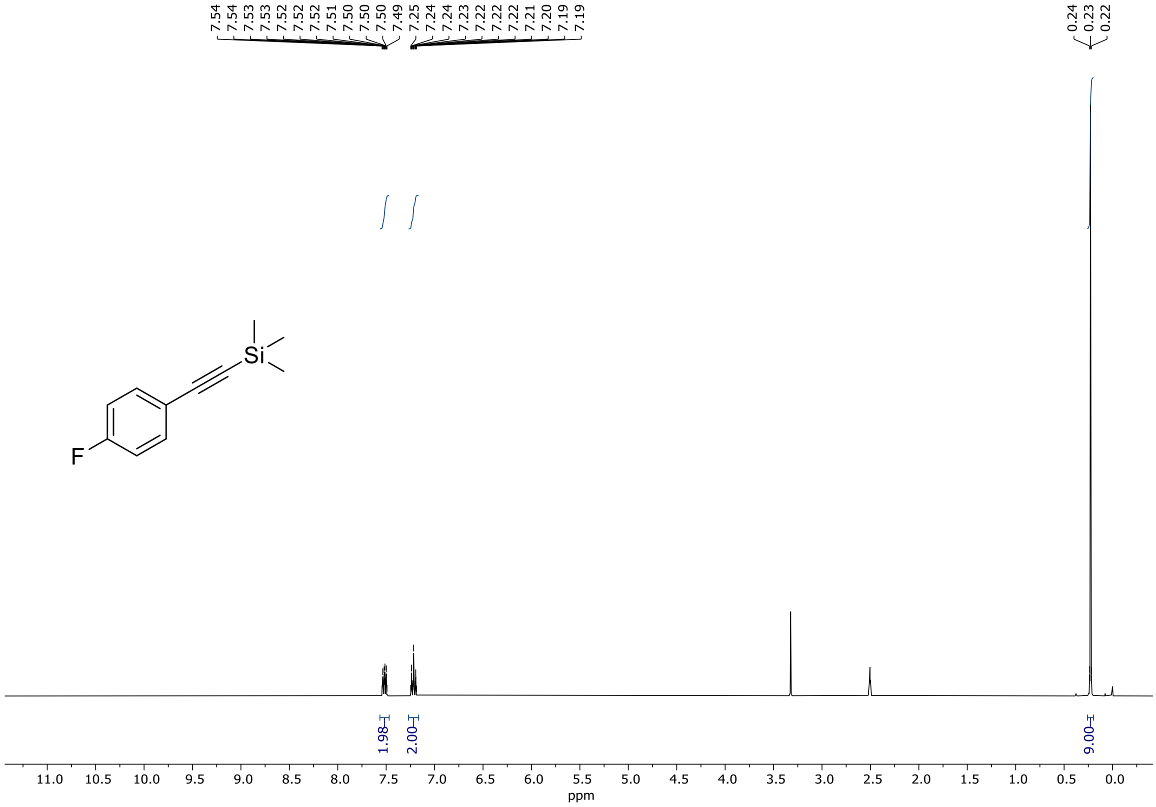

Figure S3: ^{1}H-NMR spectrum of compound **10**.

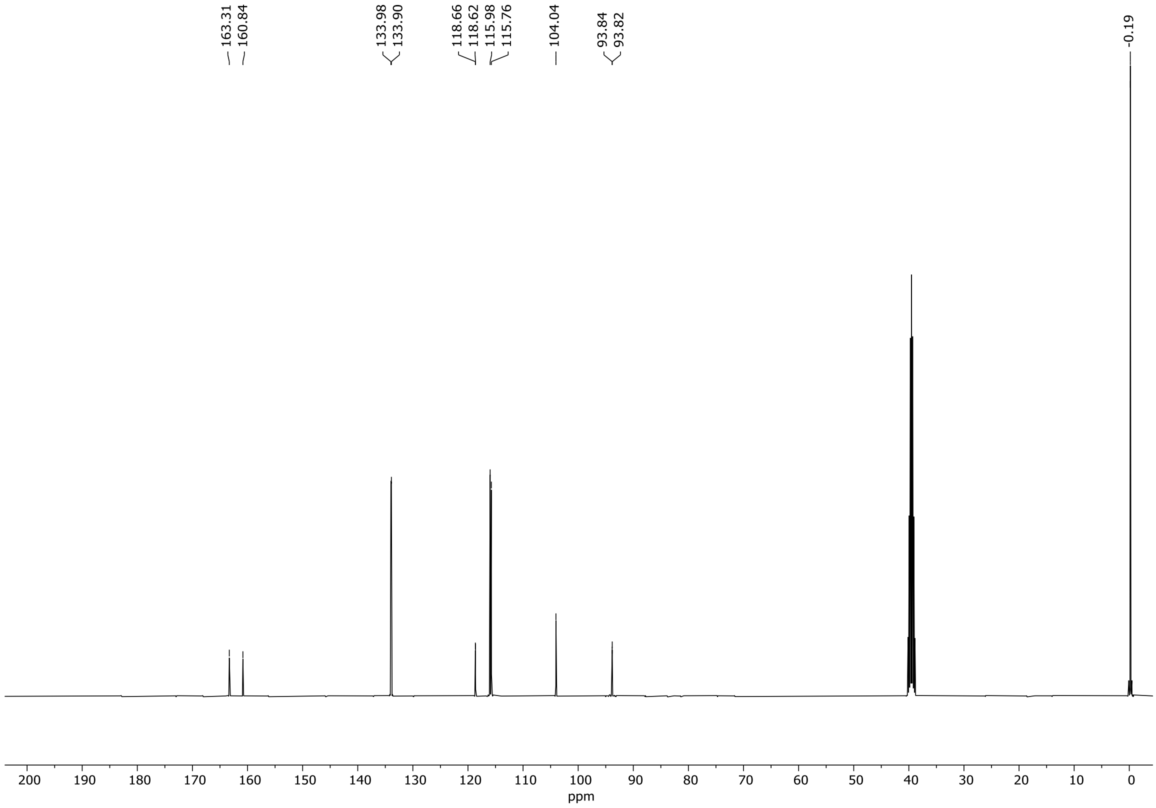

Figure S4: ^{13}C-NMR spectrum of compound **10**.

6-Chloro-3-nitropyridin-2-amine (**13**)

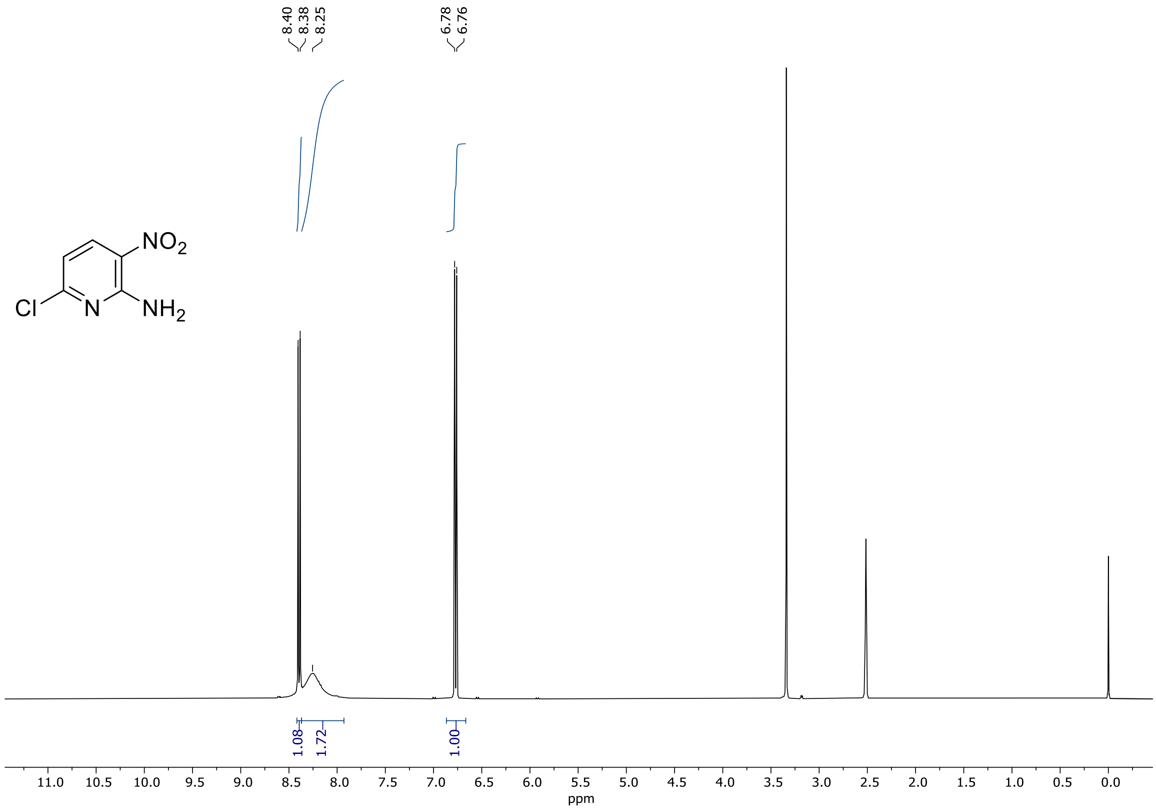

Figure S5: ^{1}H-NMR spectrum of compound **13**.

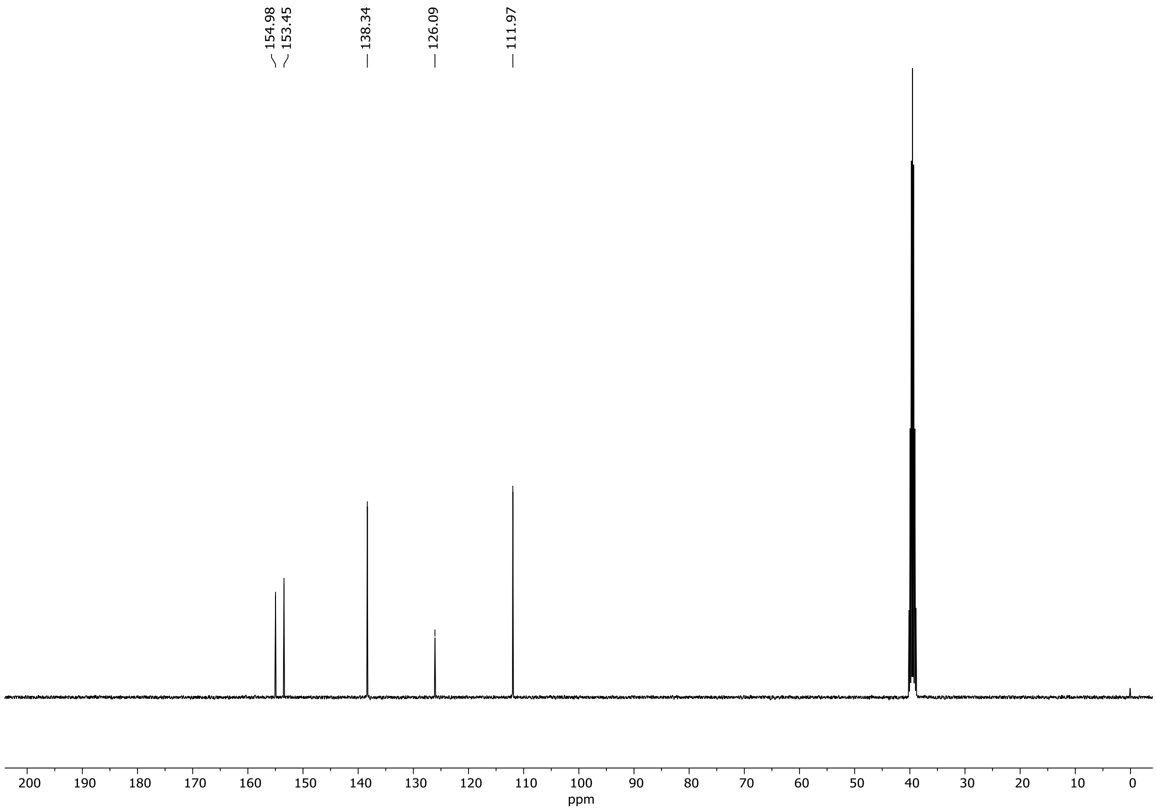

Figure S6: ^{13}C-NMR spectrum of compound **13**.

6-Bromo-3-nitropyridin-2-amine (**14**)

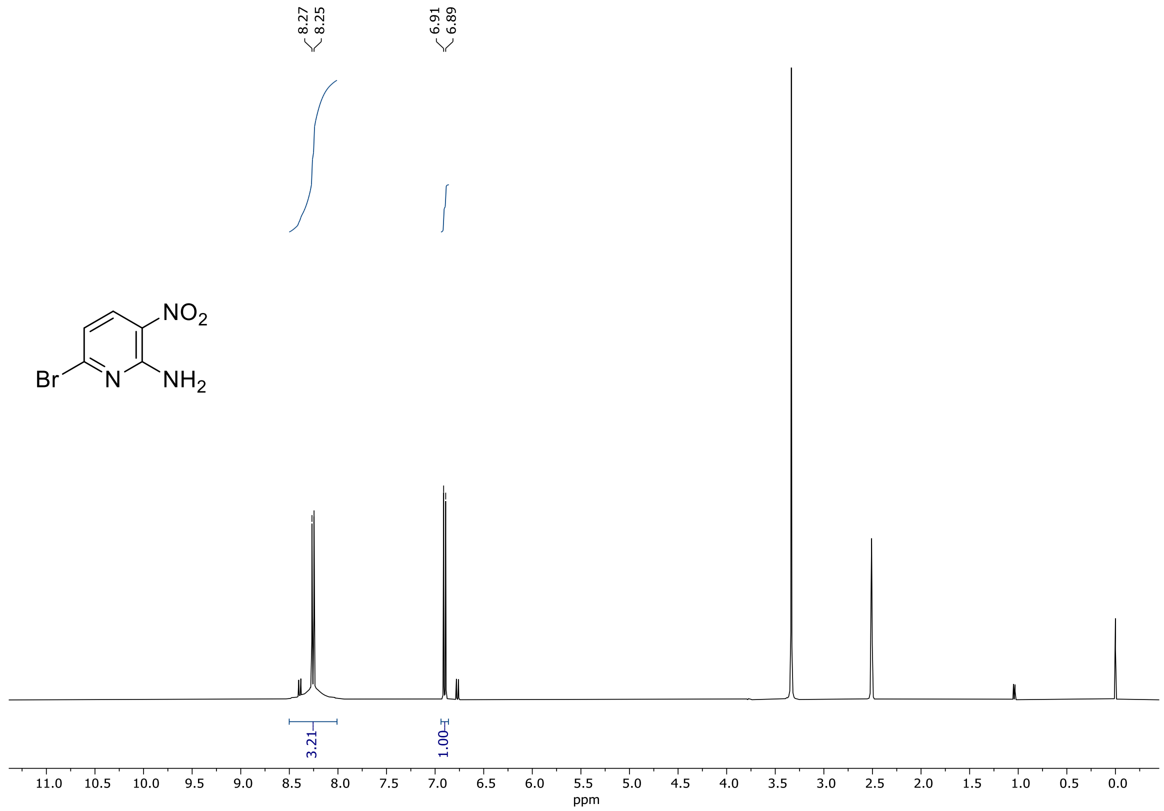

Figure S7: ^{1}H-NMR spectrum of compound **14**.

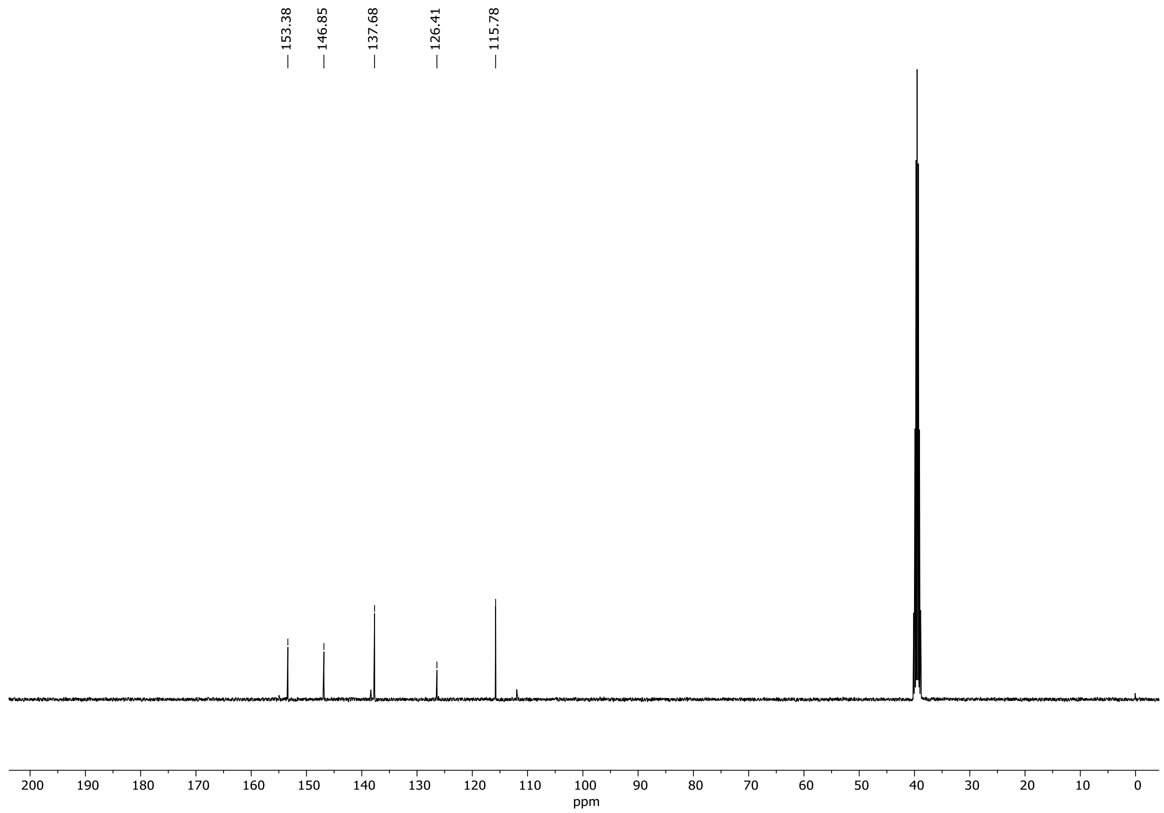

Figure S8: ^{13}C-NMR spectrum of compound **14**.

6-[(4-Fluorophenyl)ethynyl]-3-nitropyridin-2-amine (**15**)

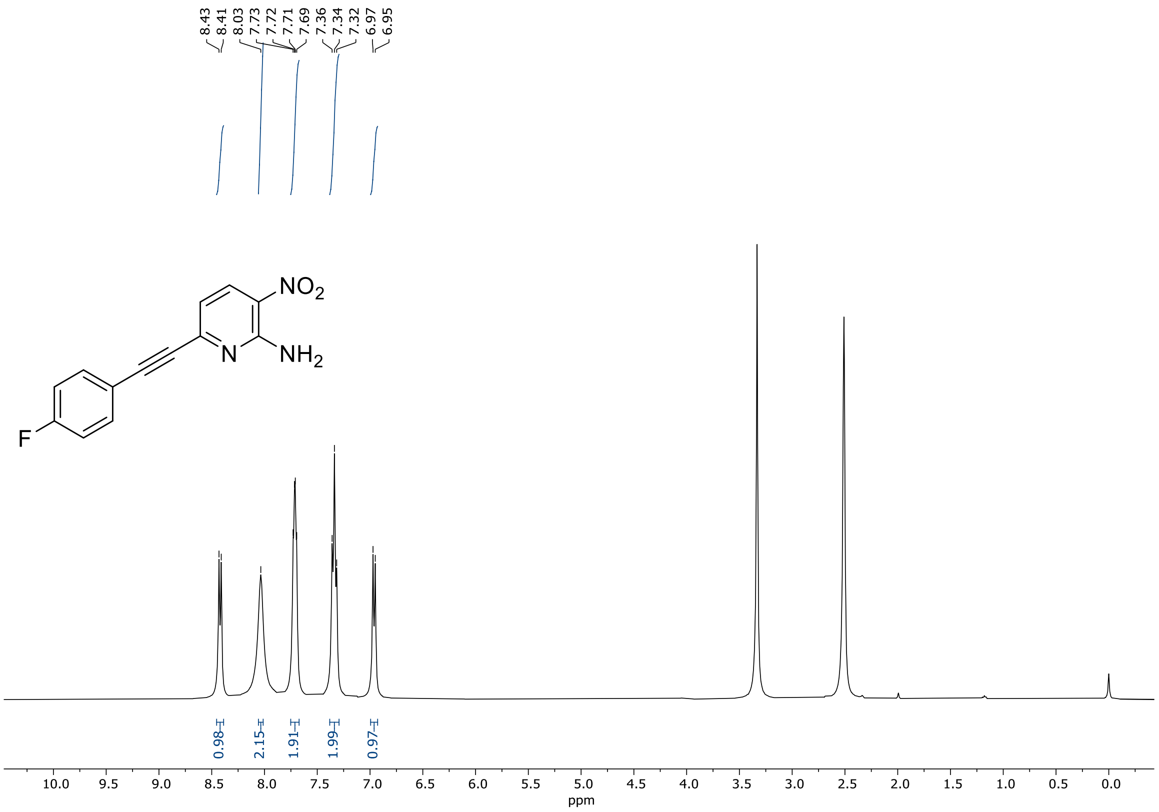

Figure S9: ^{1}H-NMR spectrum of compound **15**.

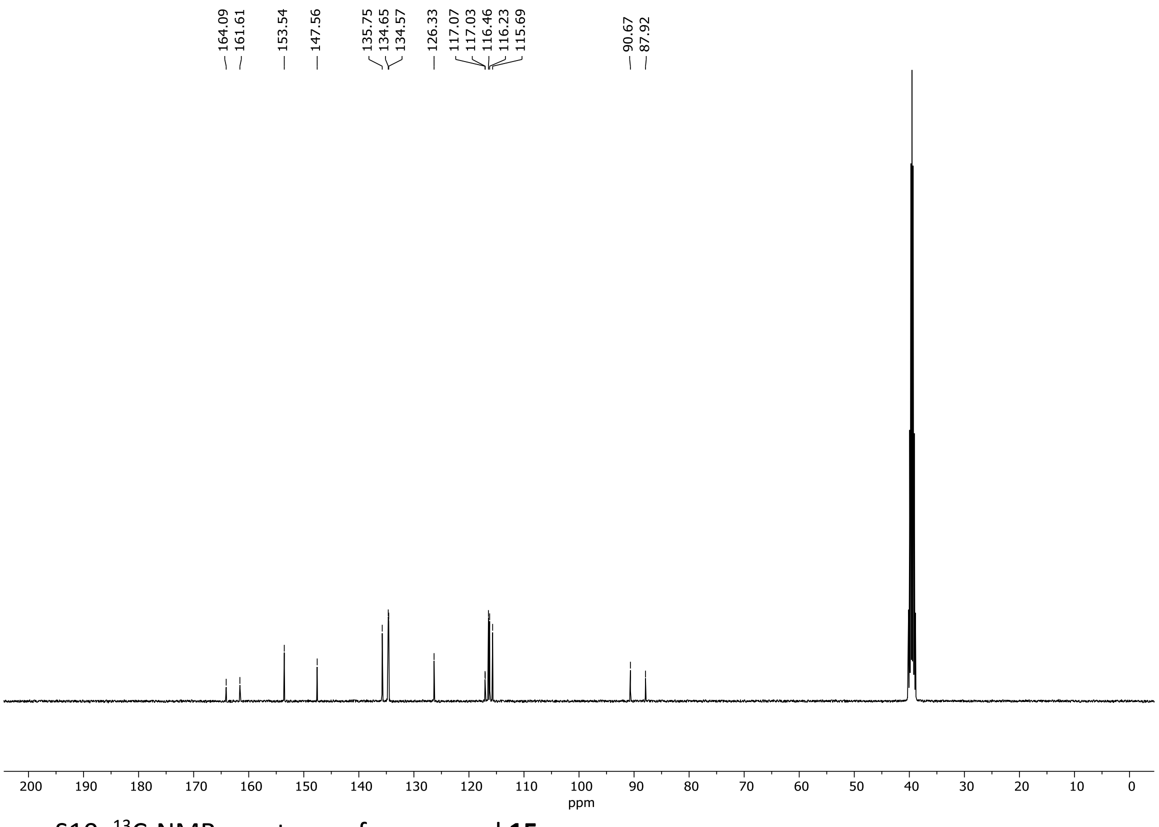

Figure S10: ^{13}C-NMR spectrum of compound **15**.

3-Nitro-6-(phenylethynyl)pyridin-2-amine (**16**)

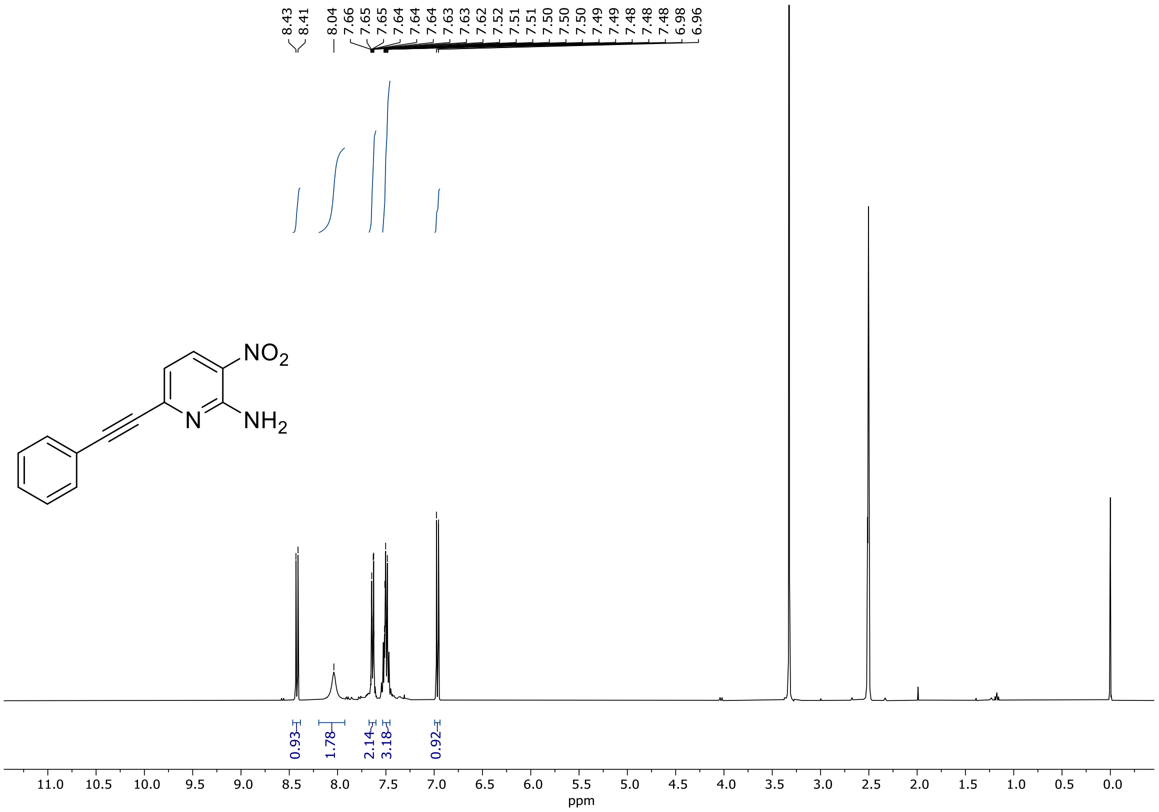

Figure S11: ^{1}H-NMR spectrum of compound **16**.

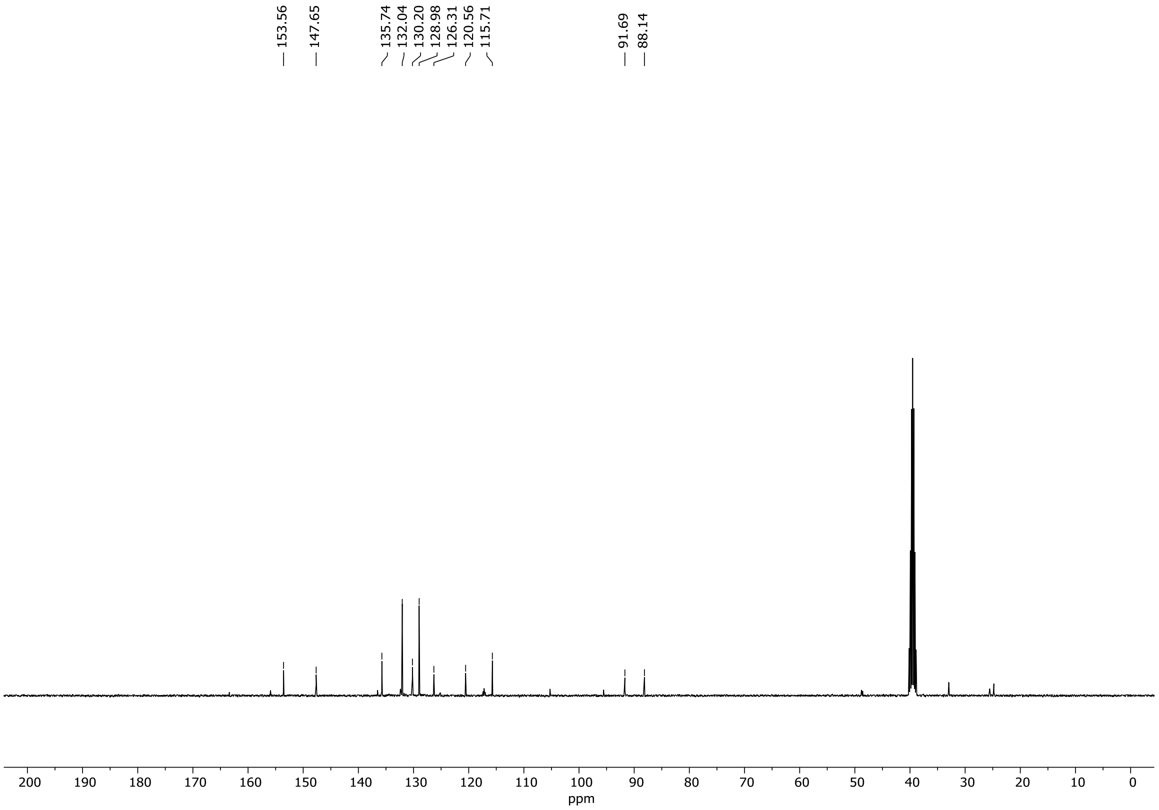

Figure S12: ^{13}C-NMR spectrum of compound **16**.

3-Nitro-6-[(trimethylsilyl)ethynyl]pyridin-2-amine (**17**)

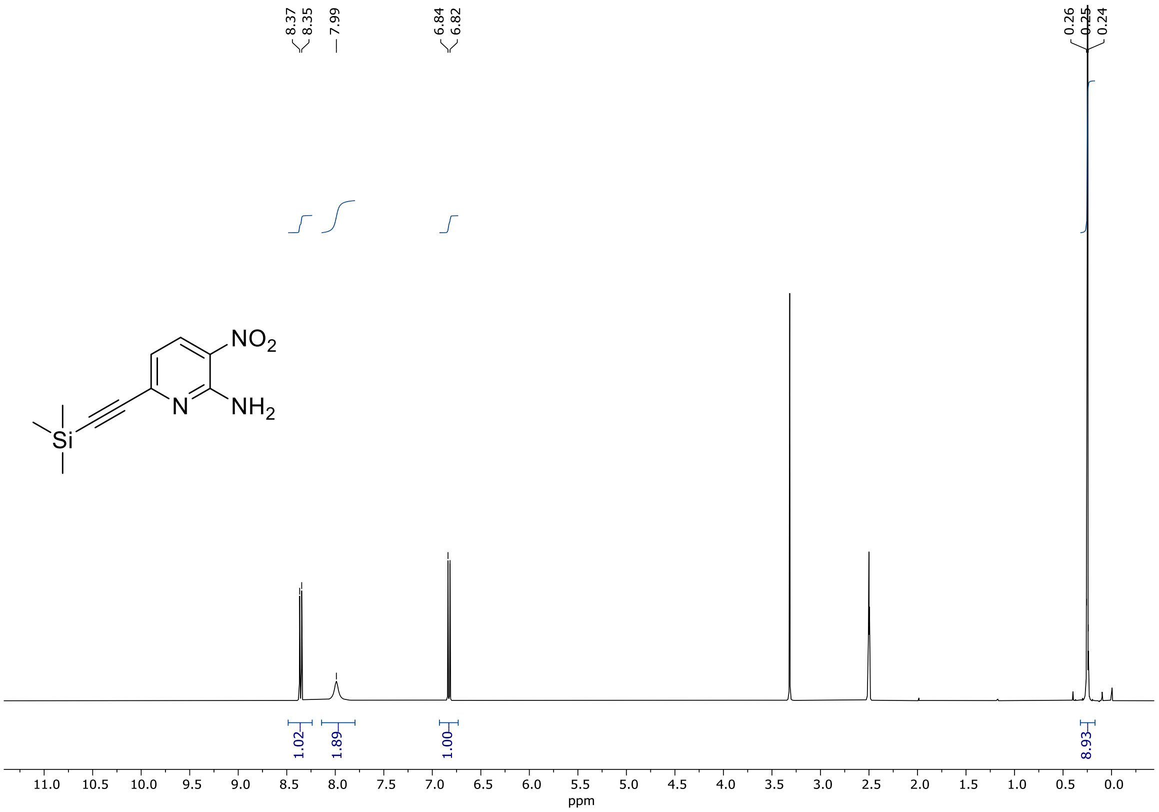

Figure S13: ^{1}H-NMR spectrum of compound **17**.

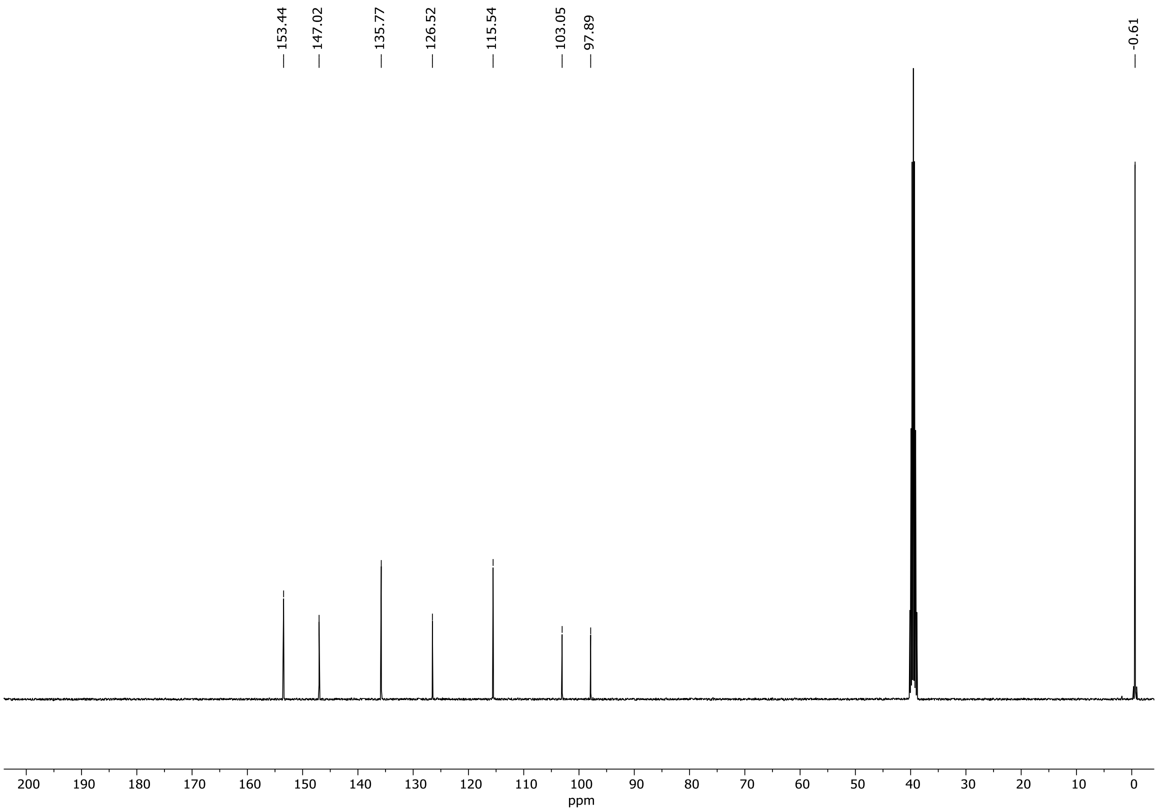

Figure S14: ^{13}C-NMR spectrum of compound **17**.

6-Ethynyl-3-nitropyridin-2-amine (**18**)

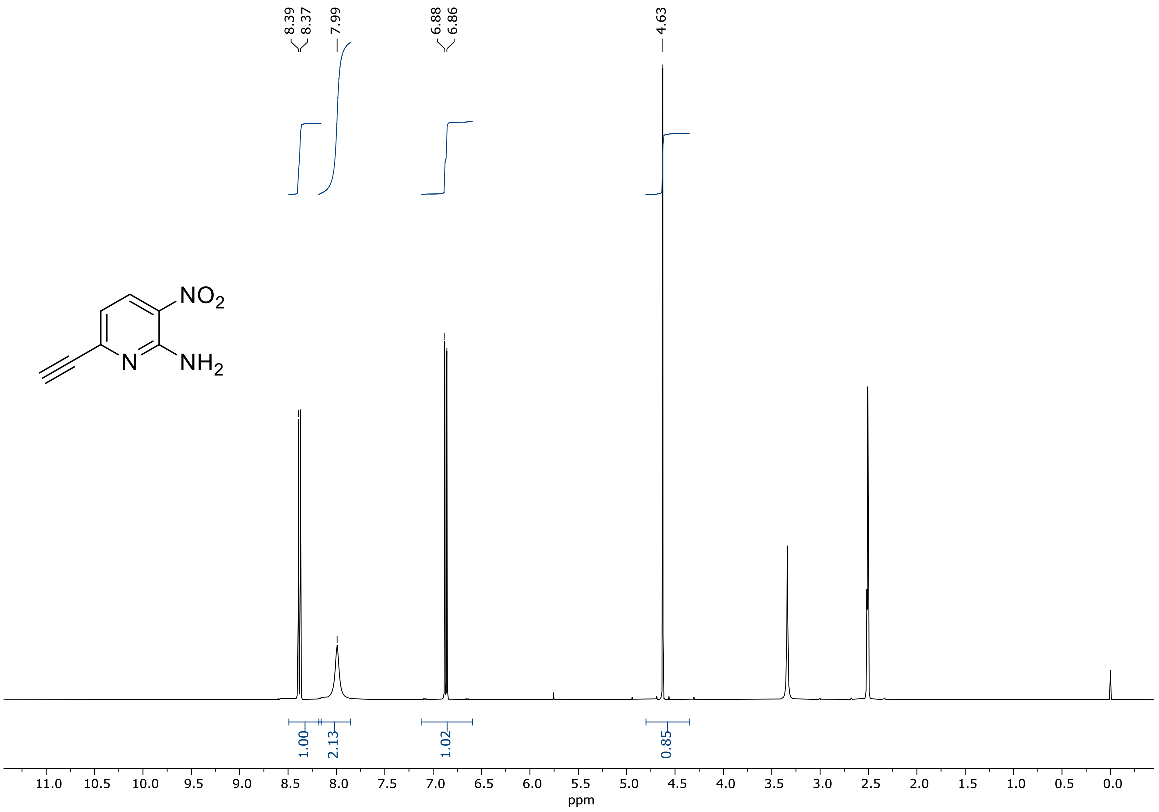

Figure S15: ^{1}H-NMR spectrum of compound **18**.

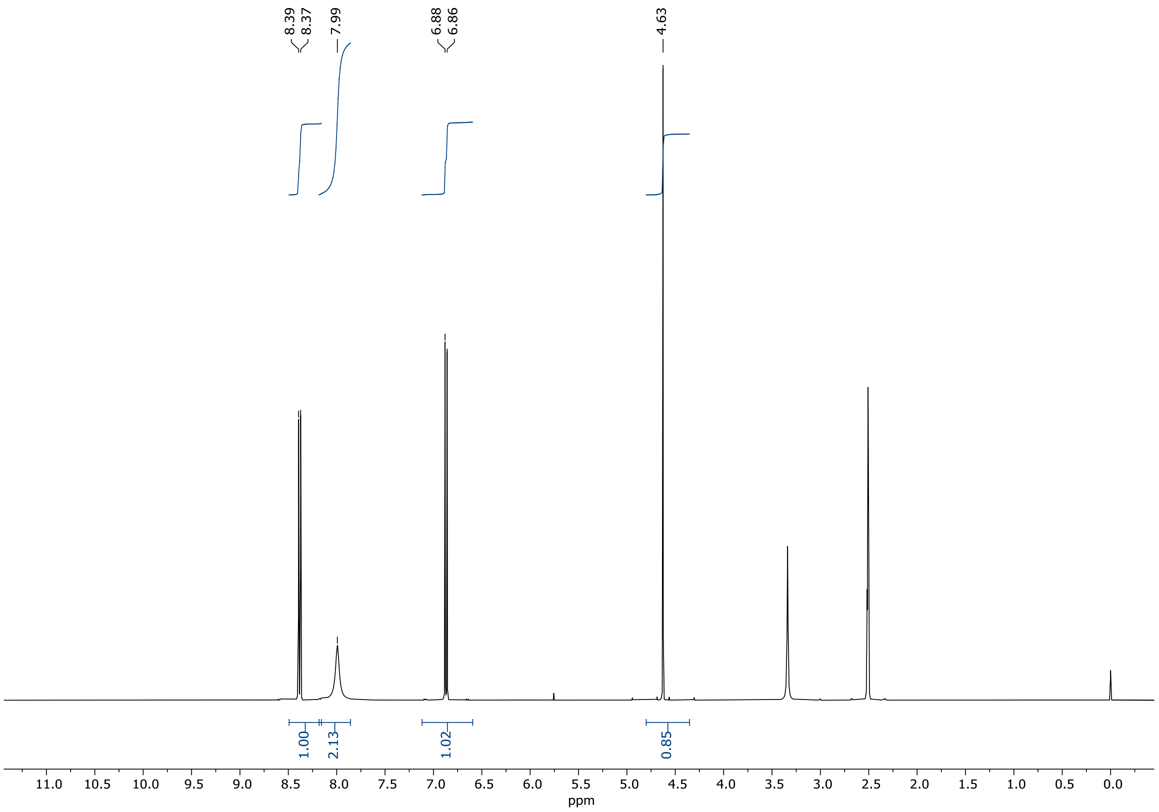

Figure S16: ^{13}C-NMR spectrum of compound **18**.

Ethyl [2-amino-6-(phenylethynyl)pyridin-3-yl]carbamate (**21**)

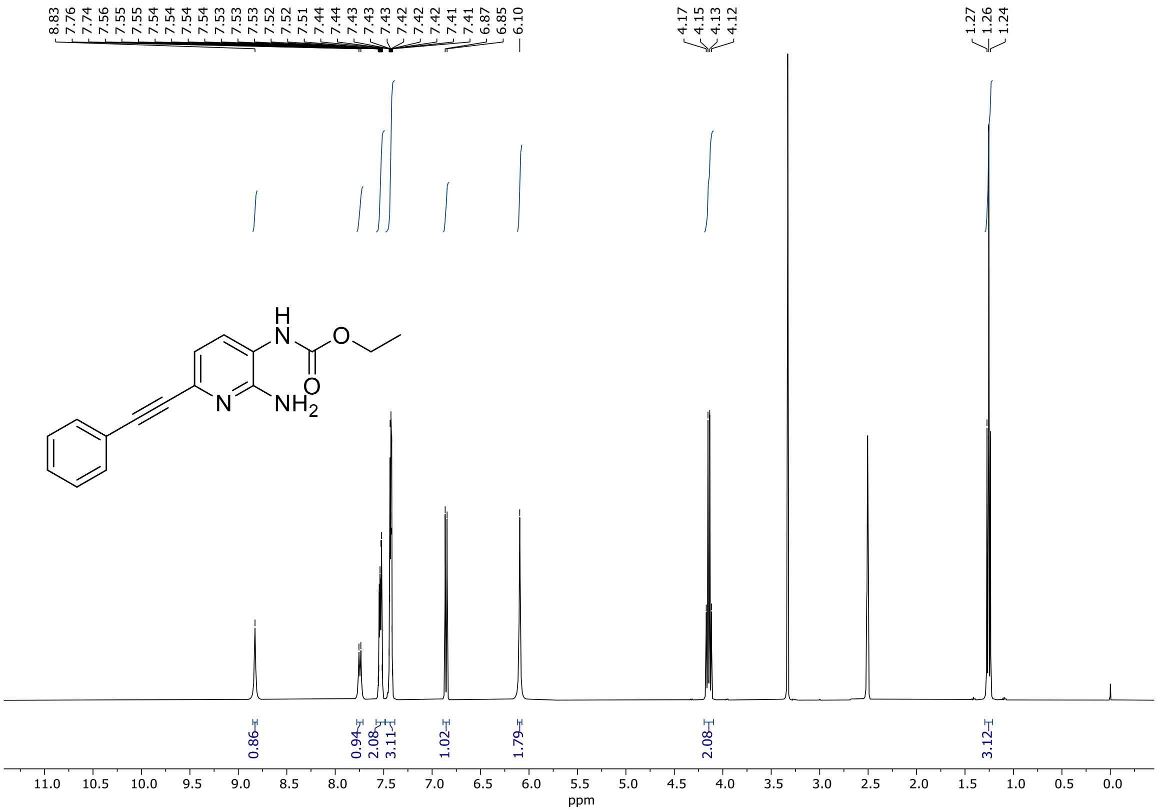

Figure S17: ^{1}H-NMR spectrum of compound **21**.

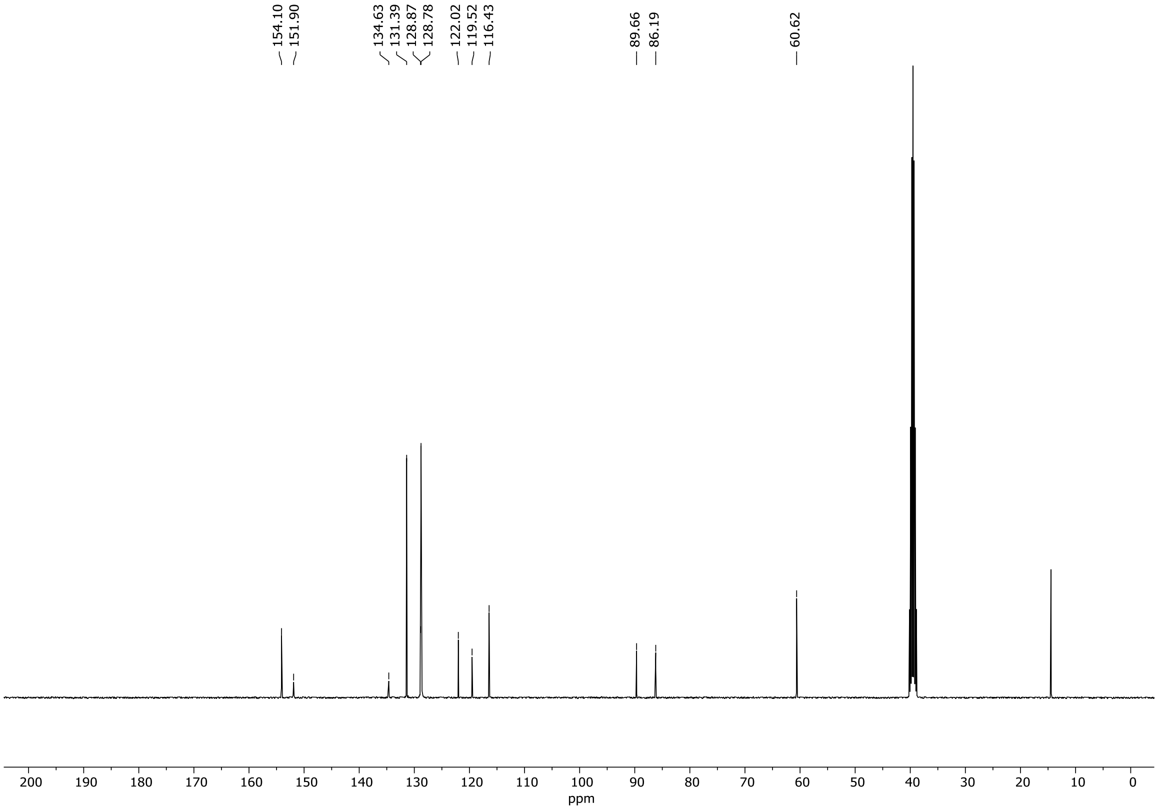

Figure S18: ^{13}C-NMR spectrum of compound **21**.

6-Methyl-5-nitropicolinonitrile (**23**)

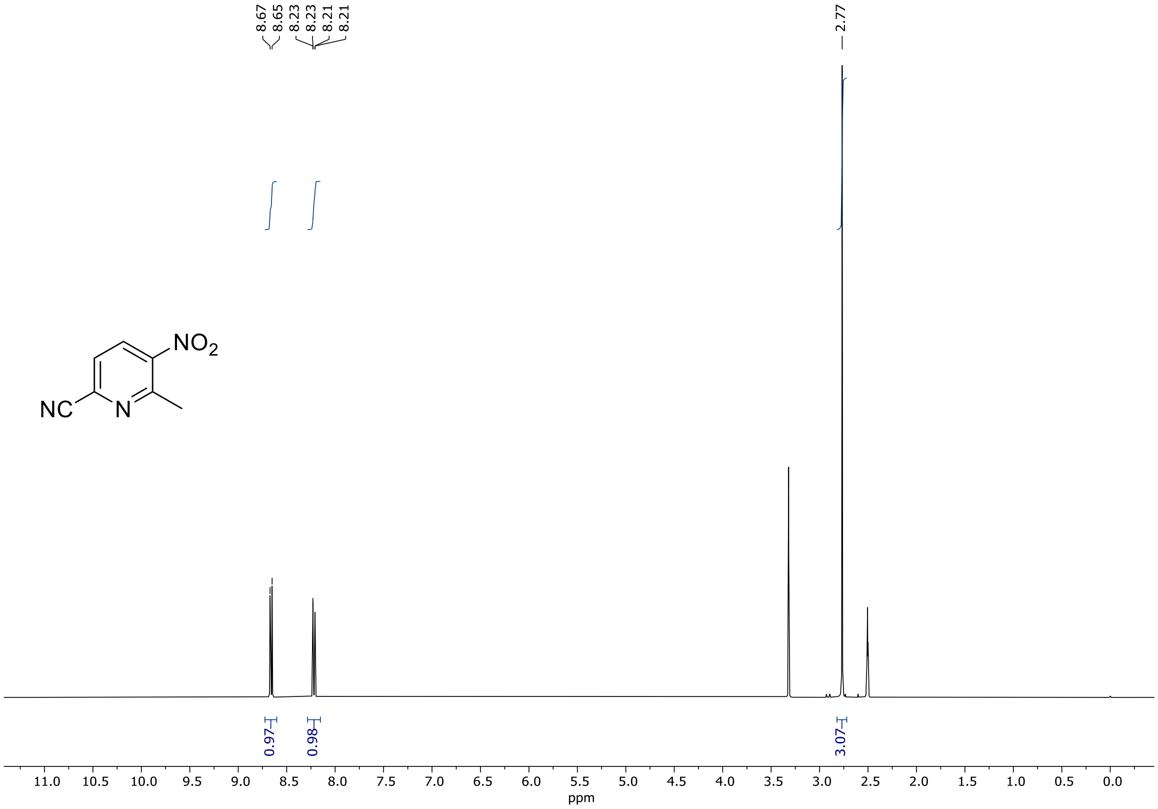

Figure S19: ^{1}H-NMR spectrum of compound **23**.

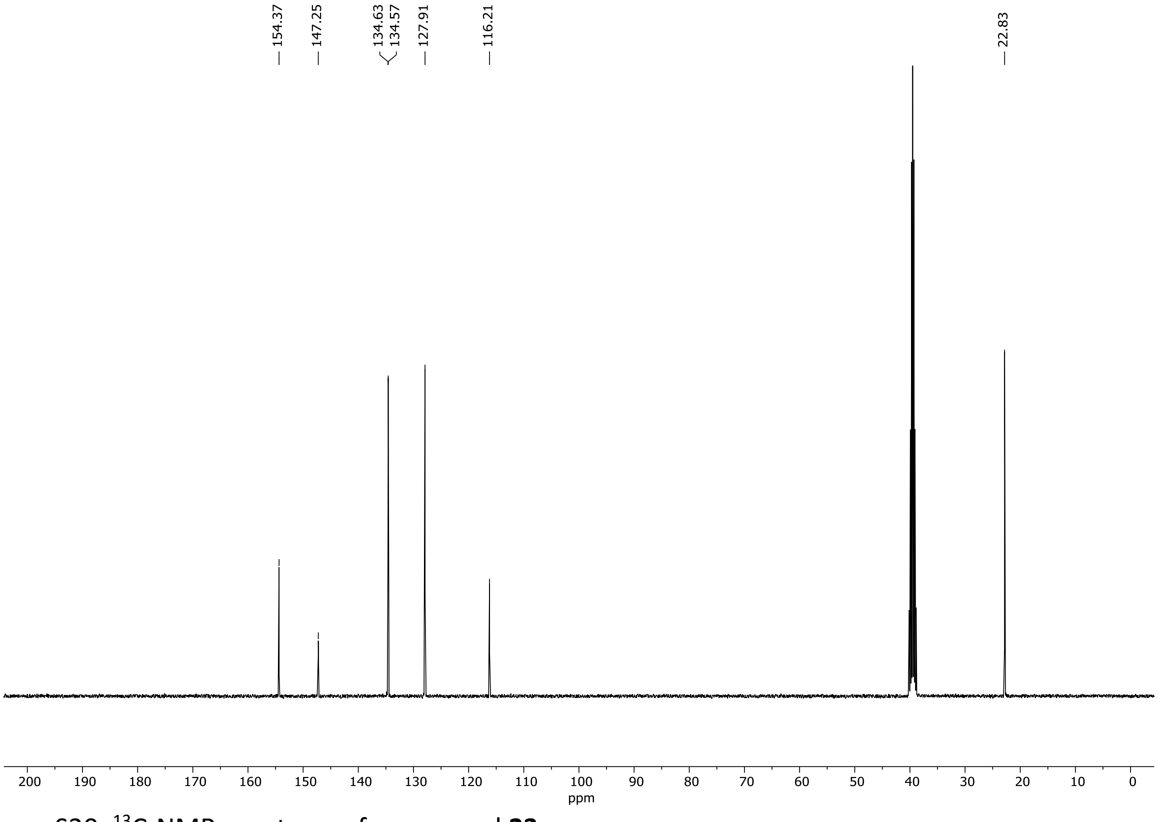

Figure S20: ^{13}C-NMR spectrum of compound **23**.

5-Amino-6-methylpicolinonitrile (**24**)

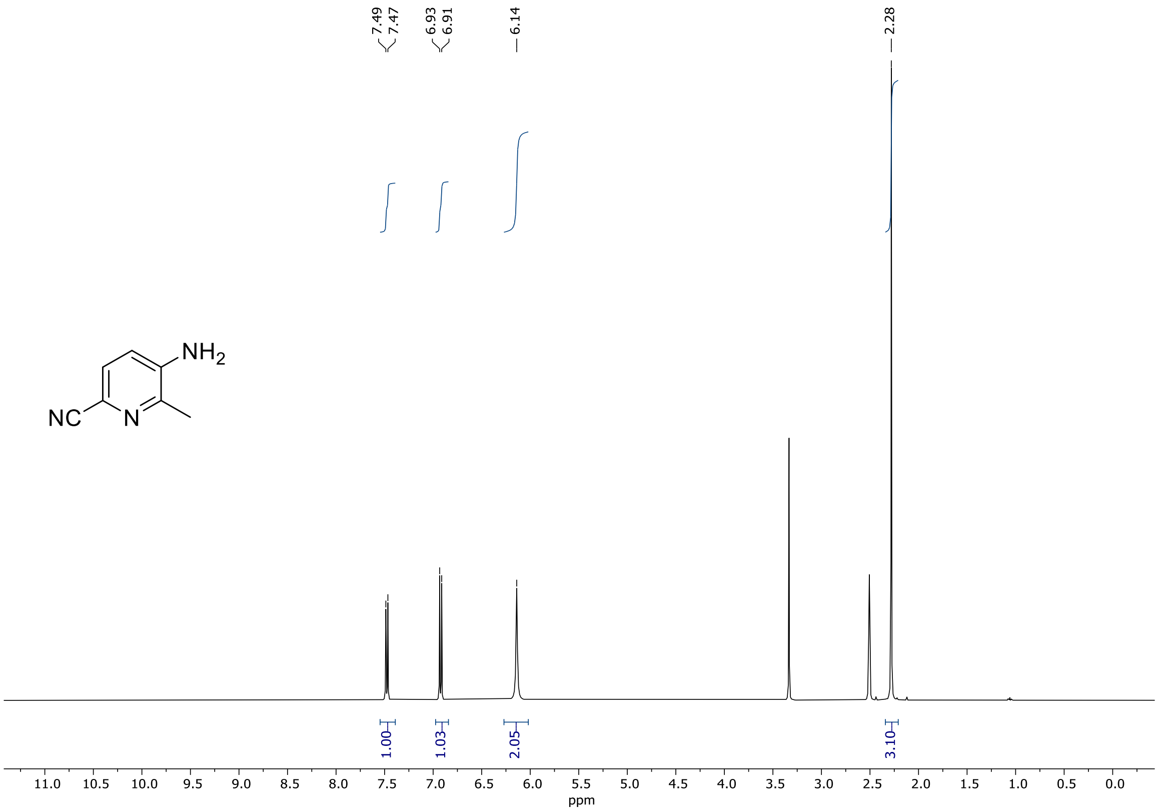

Figure S21: ^{1}H-NMR spectrum of compound **24**.

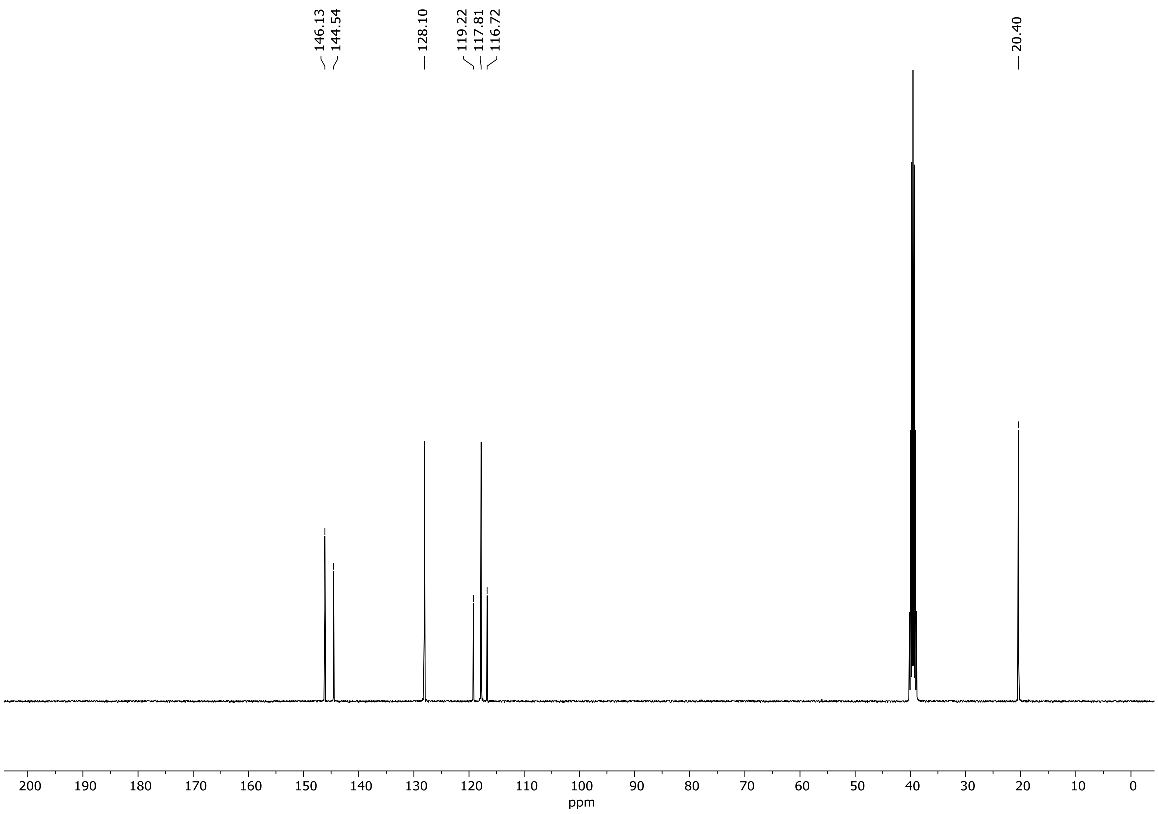

Figure S22: ^{13}C-NMR spectrum of compound **24**.

Isobutyl (6-cyano-2-methylpyridin-3-yl)carbamate (**25**)

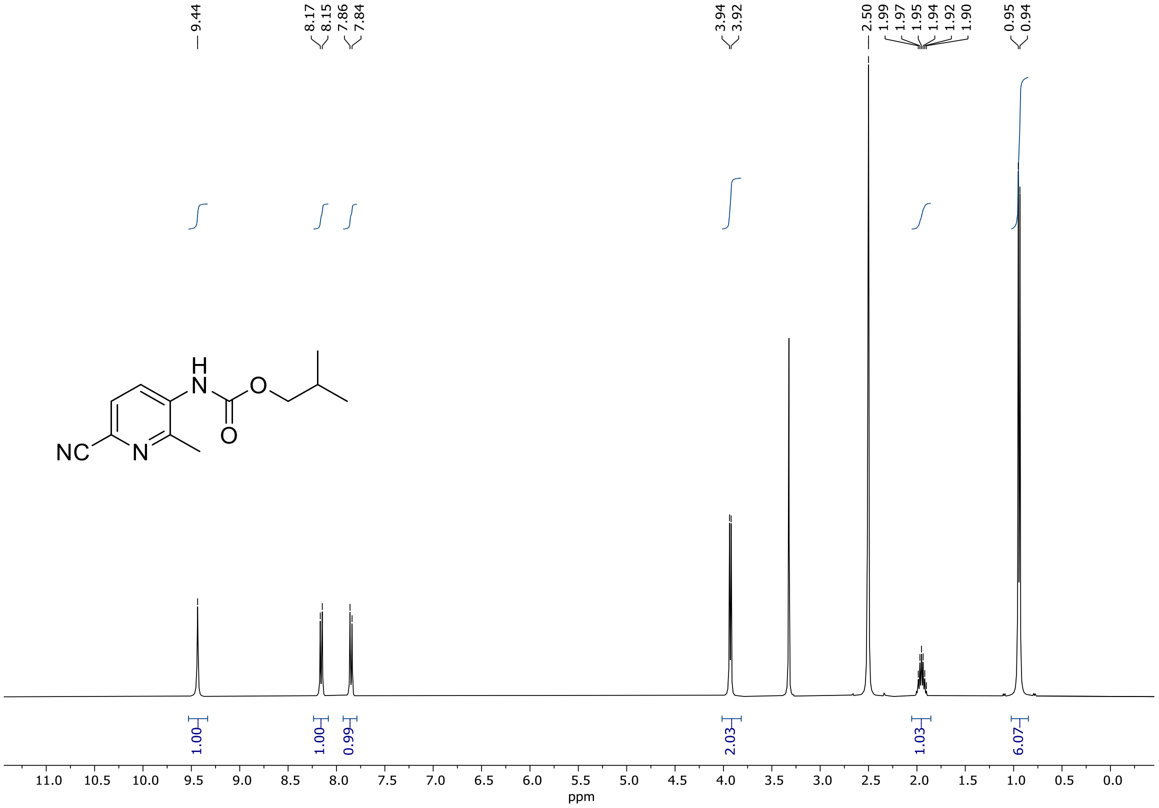

Figure S23: ^{1}H-NMR spectrum of compound **25** (2D-NMR spectroscopic data suggest the apparently missing methyl signal is overlayed by the DMSO signal at 2.5 ppm).

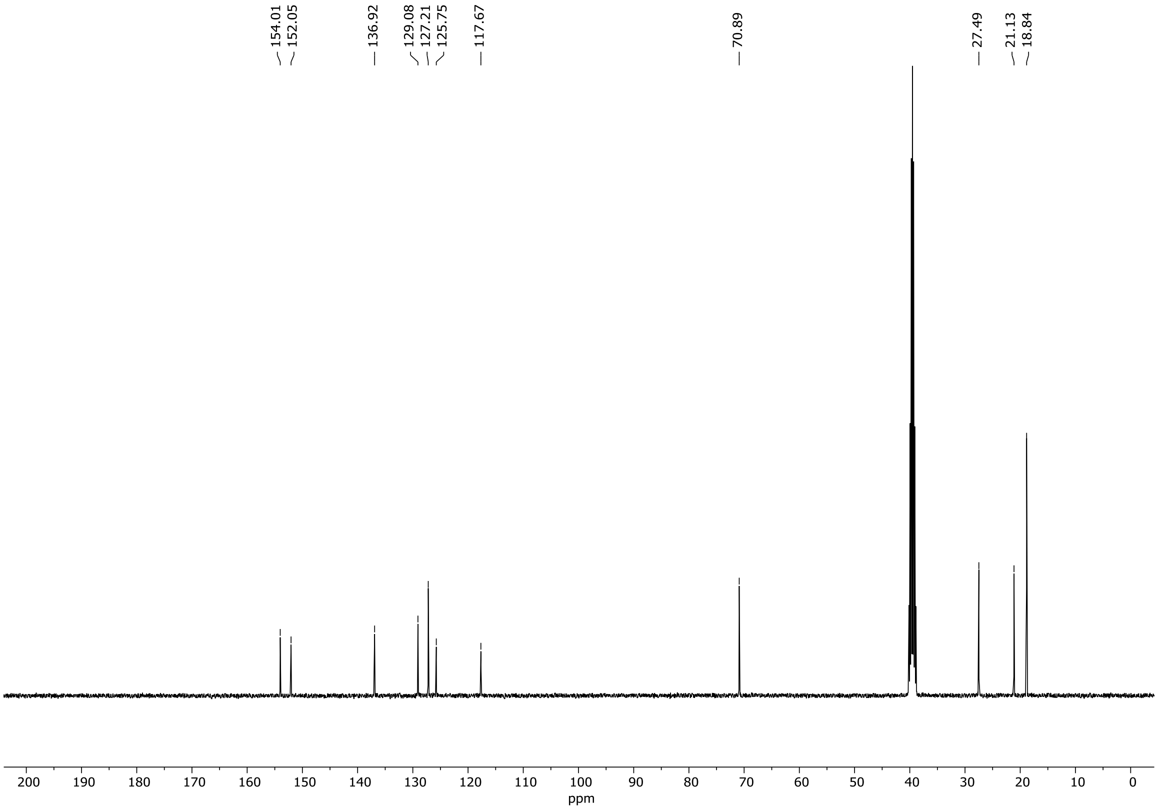

Figure S24: ^{13}C-NMR spectrum of compound **25**.

Isobutyl (6-formyl-2-methylpyridin-3-yl)carbamate (**26**)

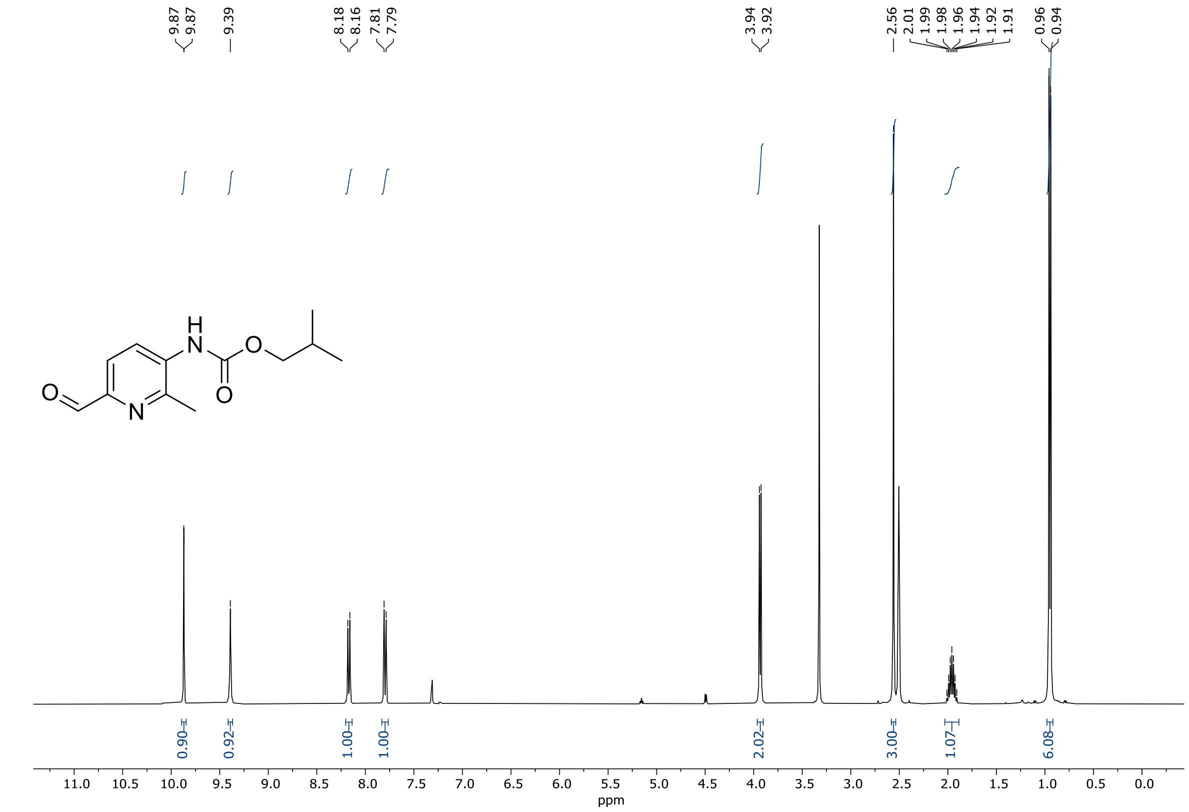

Figure S25: ^{1}H-NMR spectrum of compound **26**.

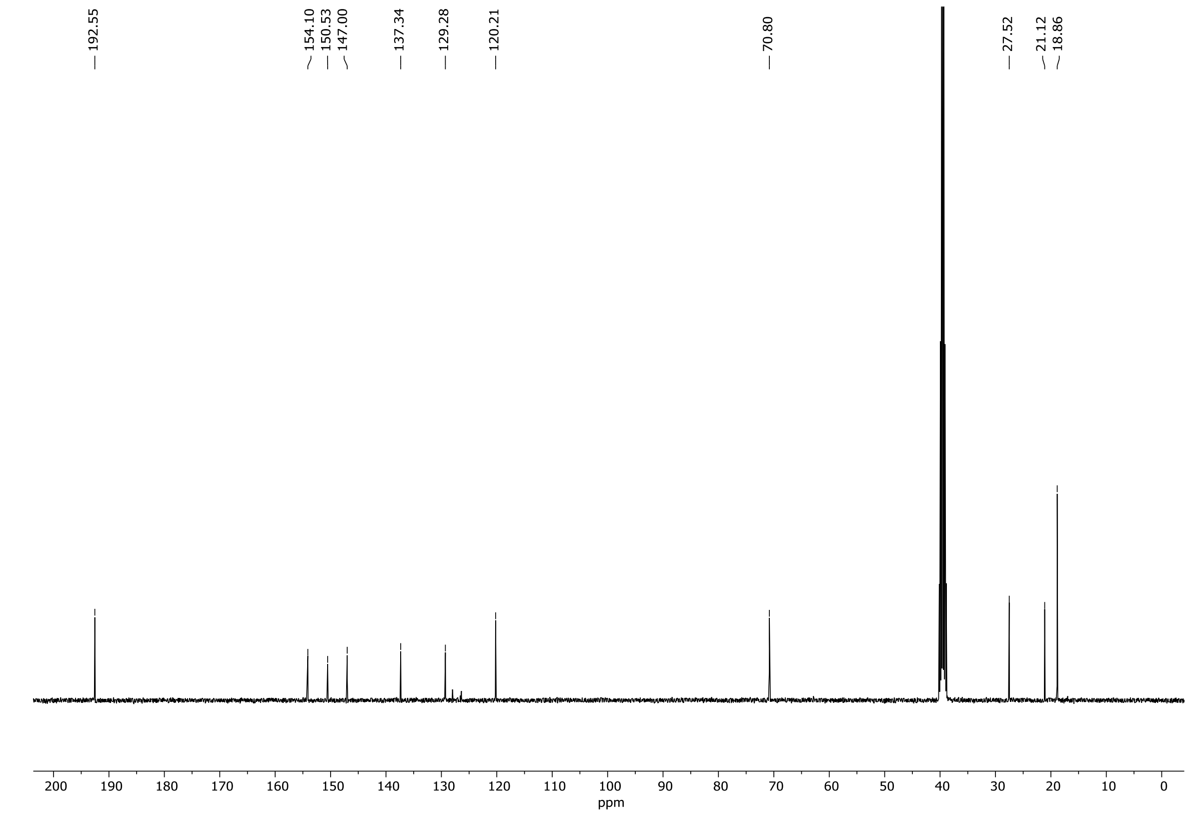

Figure S26: ^{13}C-NMR spectrum of compound **26**.

Isobutyl (6-{[(4-fluorophenyl)amino]methyl}-2-methylpyridin-3-yl)carbamate (**27**)

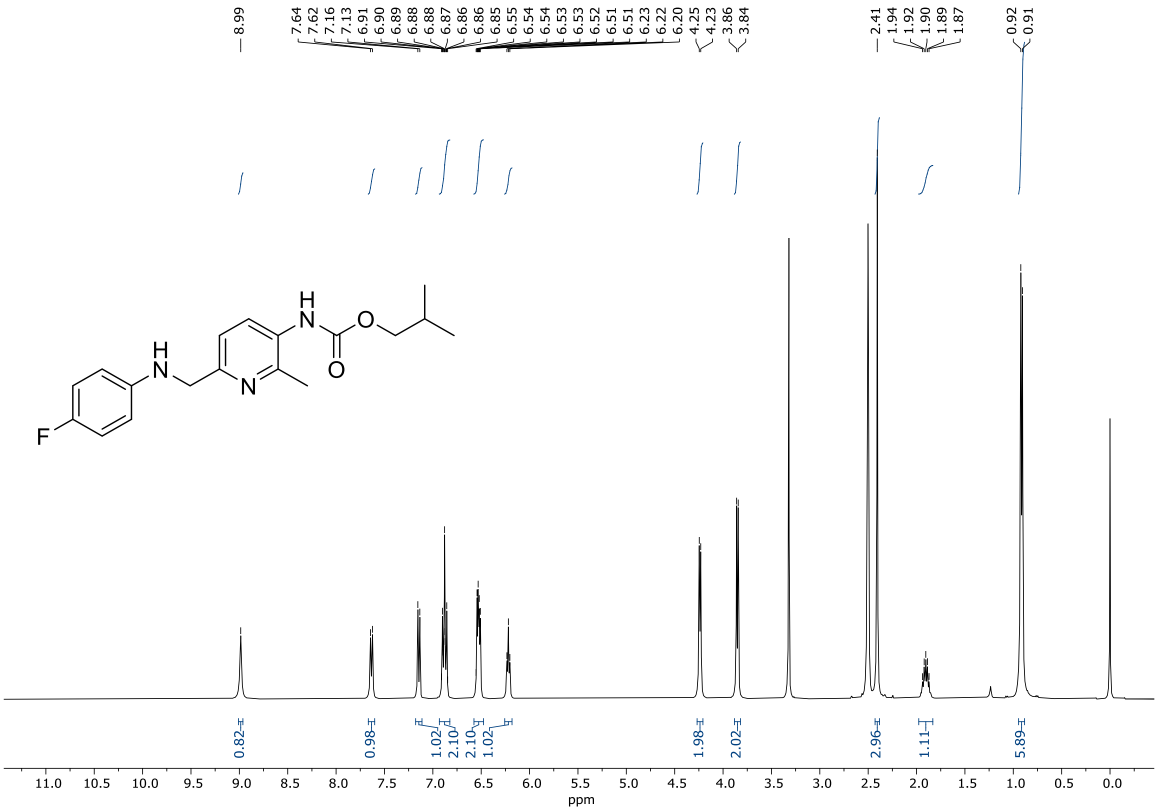

Figure S27: ^{1}H-NMR spectrum of compound **27**.

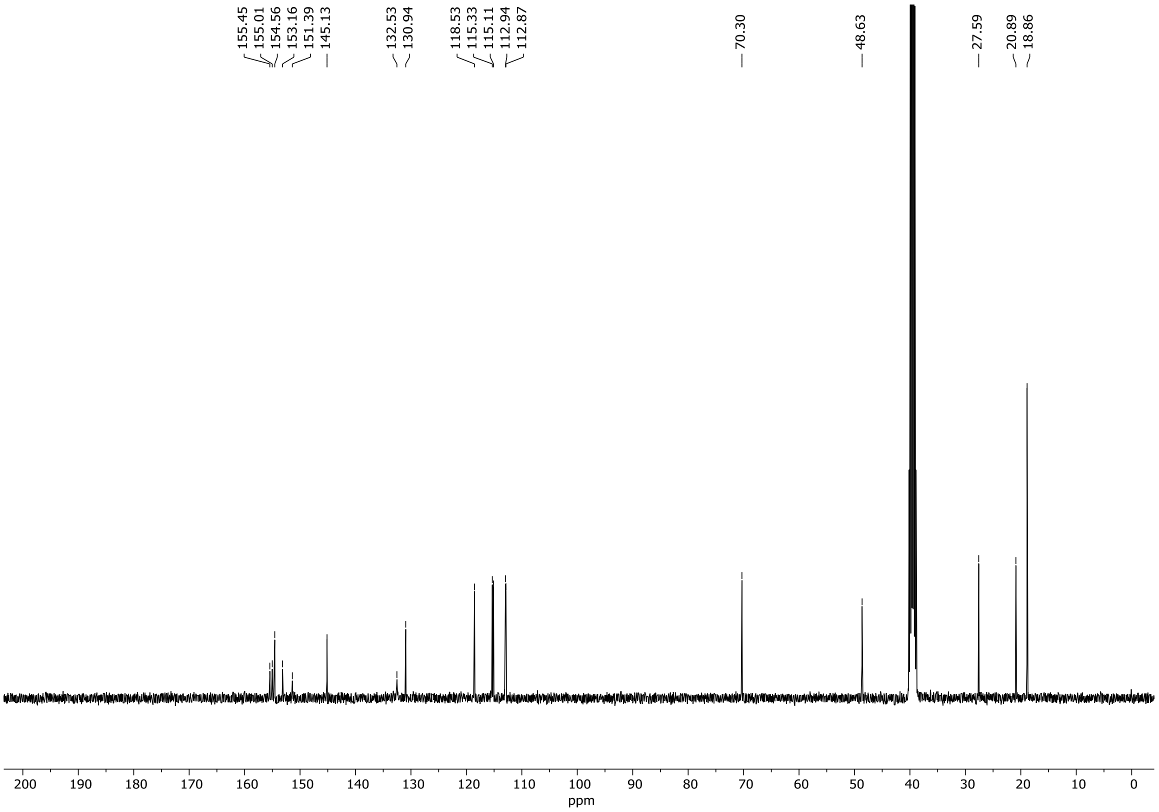

Figure S28: ^{13}C-NMR spectrum of compound **27**.

4-Nitro-3-(trifluoromethyl)benzonitrile (**29**)

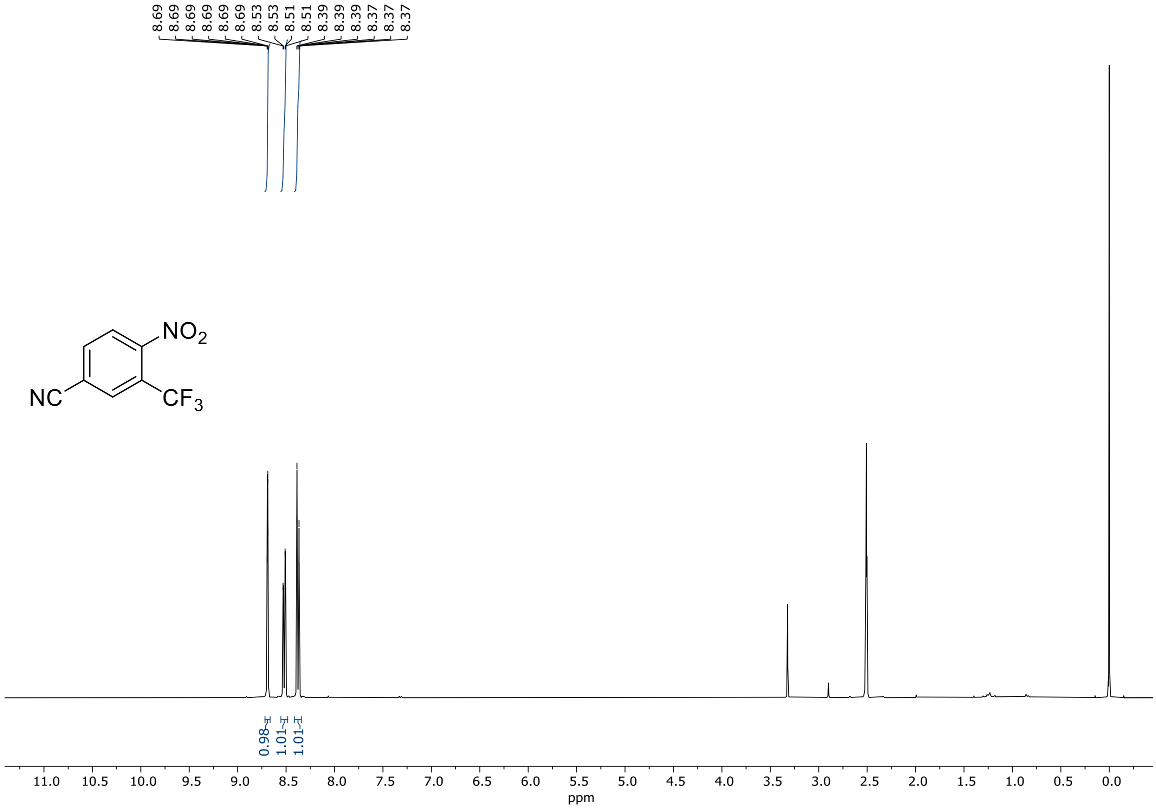

Figure S29: ^{1}H-NMR spectrum of compound **29**.

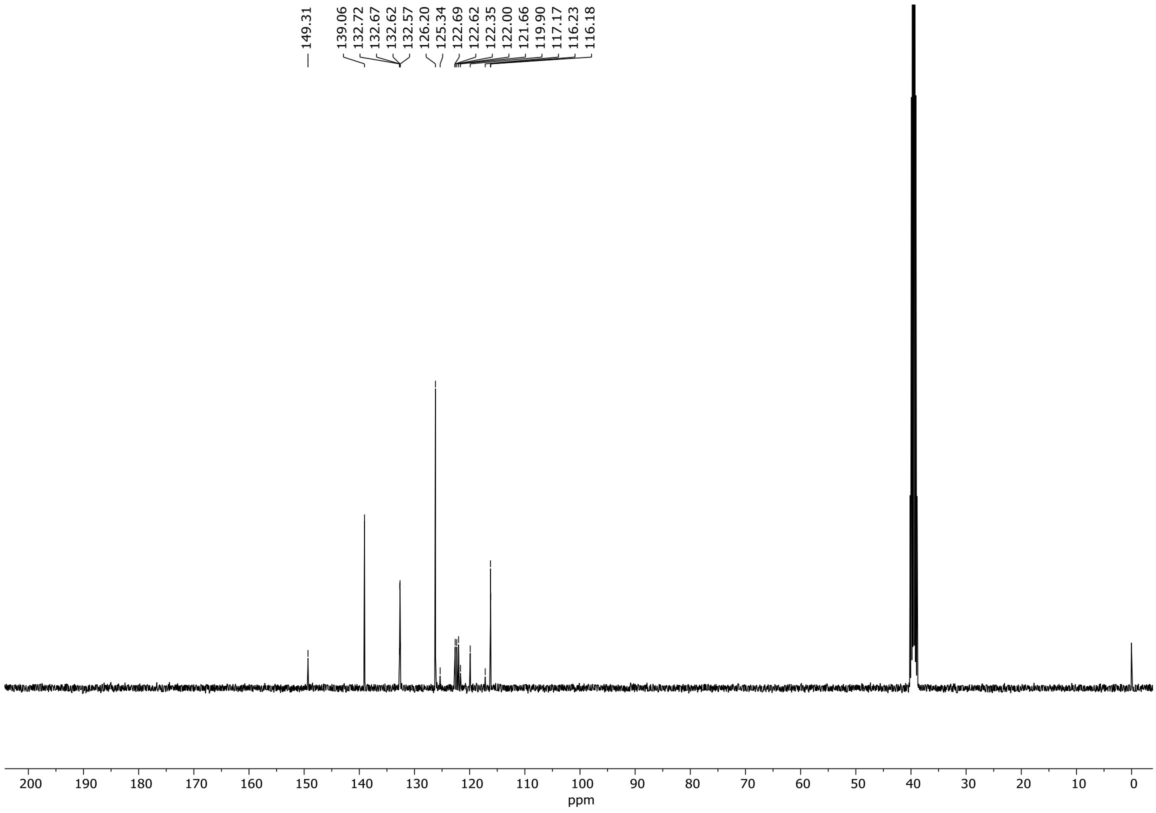

Figure S30: ^{13}C-NMR spectrum of compound **29**.

4-Bromo-2,6-dimethylaniline (**31a**)

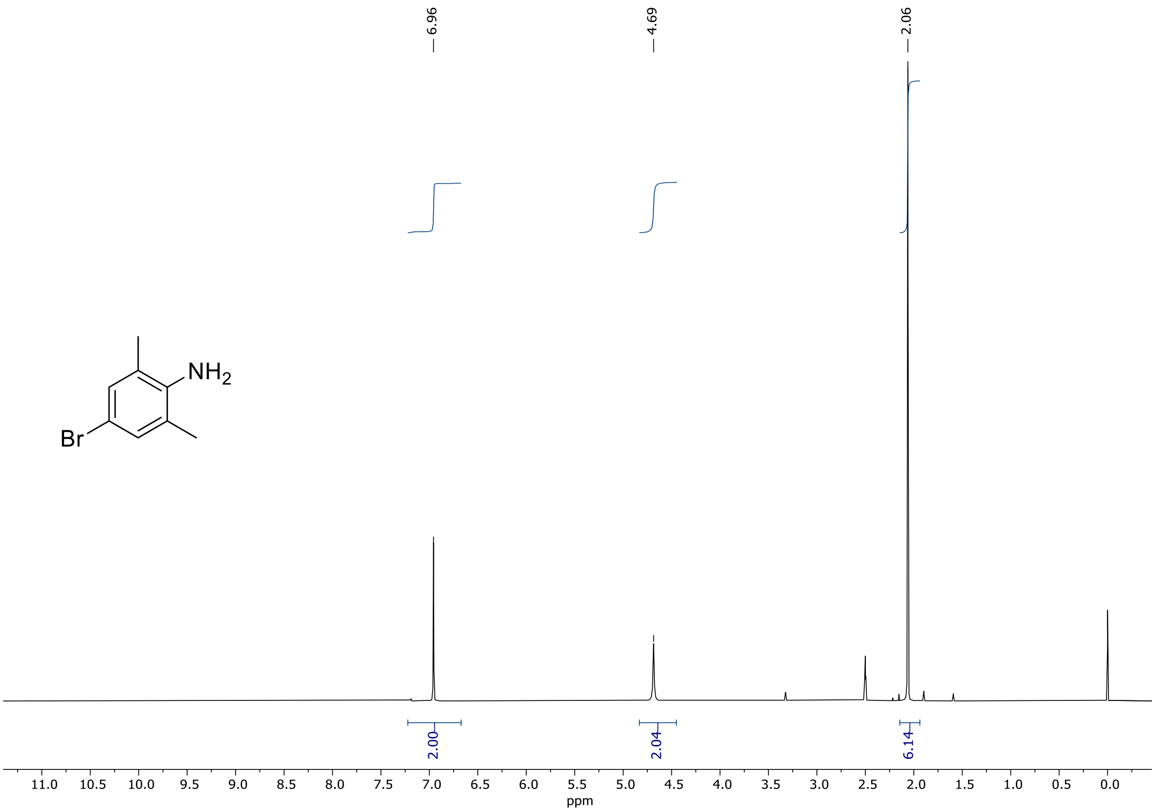

Figure S31: ^{1}H-NMR spectrum of compound **31a**.

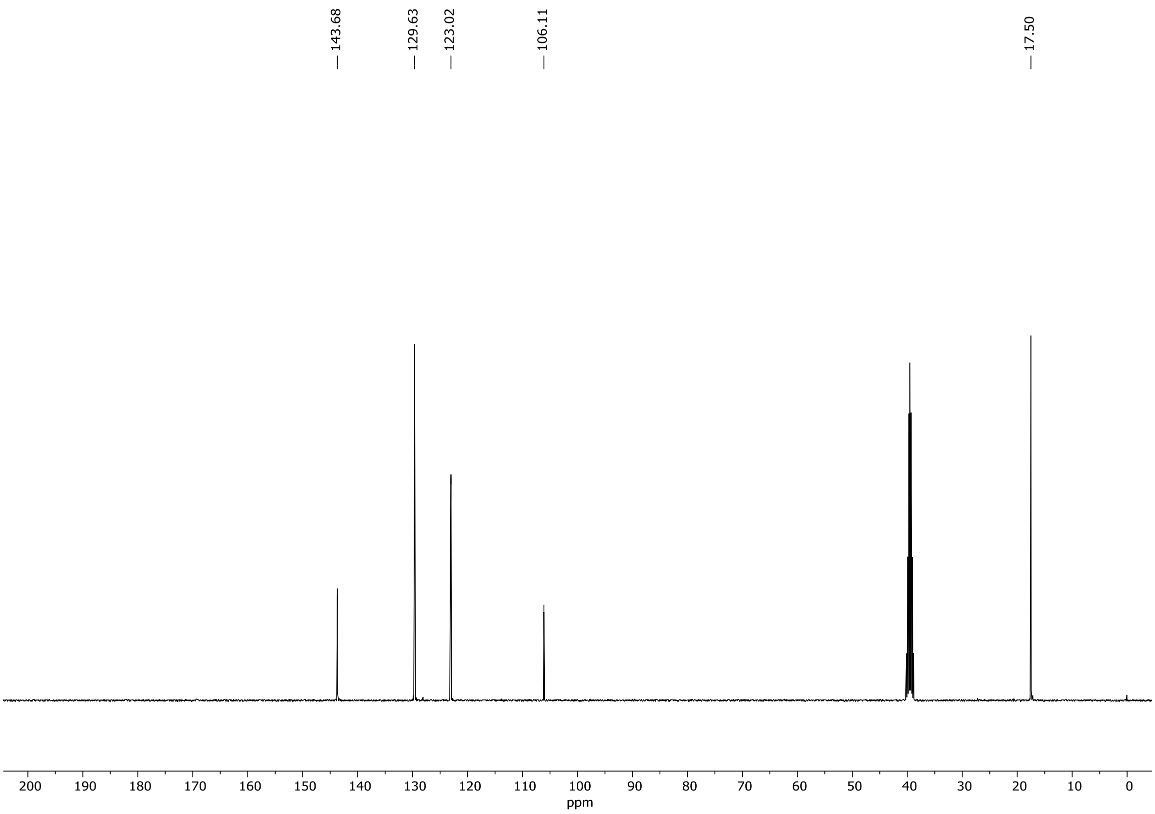

Figure S32: ^{13}C-NMR spectrum of compound **31a**.

4-Amino-3,5-dimethylbenzonitrile (**32a**)

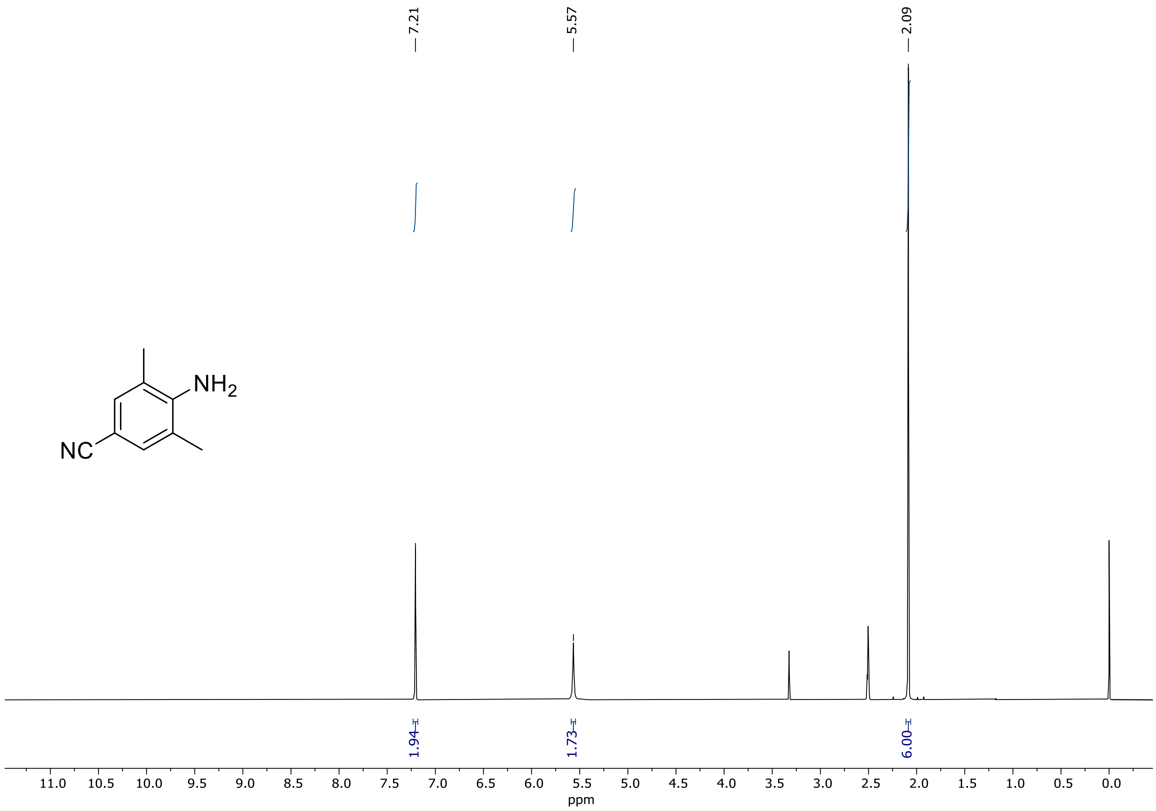

Figure S33: ^{1}H-NMR spectrum of compound **32a**.

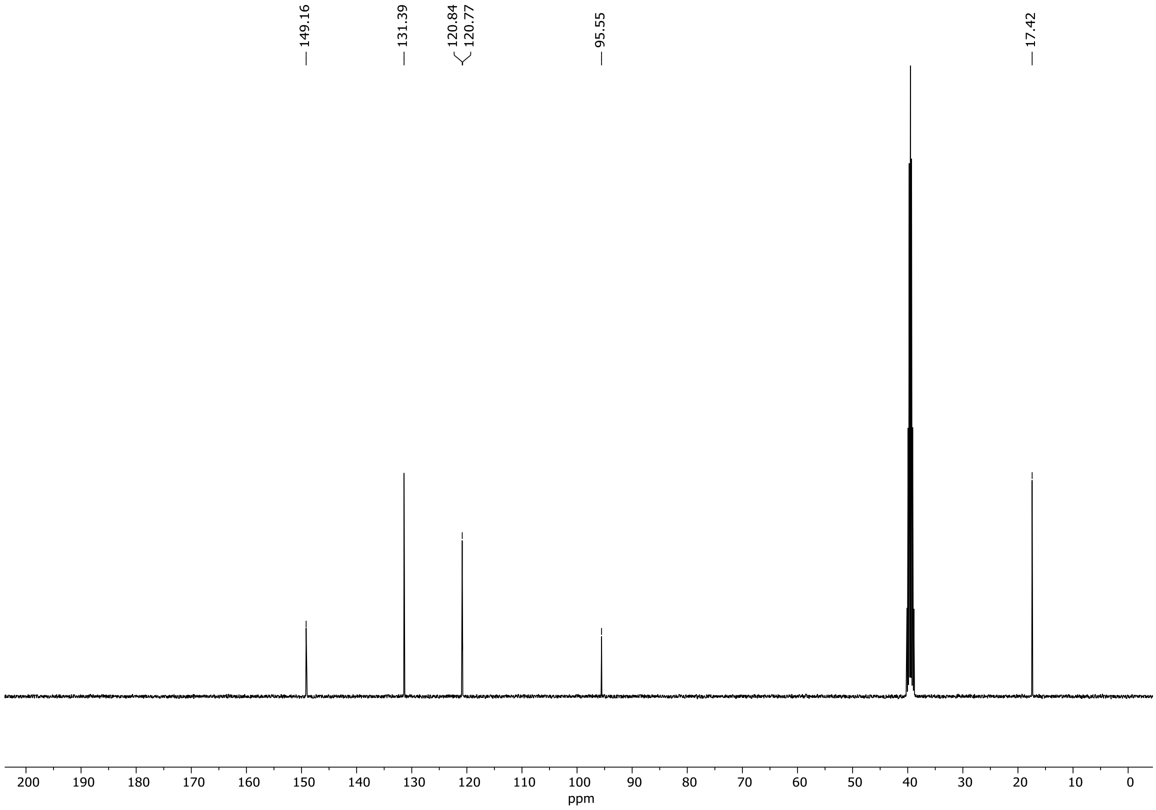

Figure S34: ^{13}C-NMR spectrum of compound **32a**.

4-Amino-3-methylbenzonitrile (**32b**)

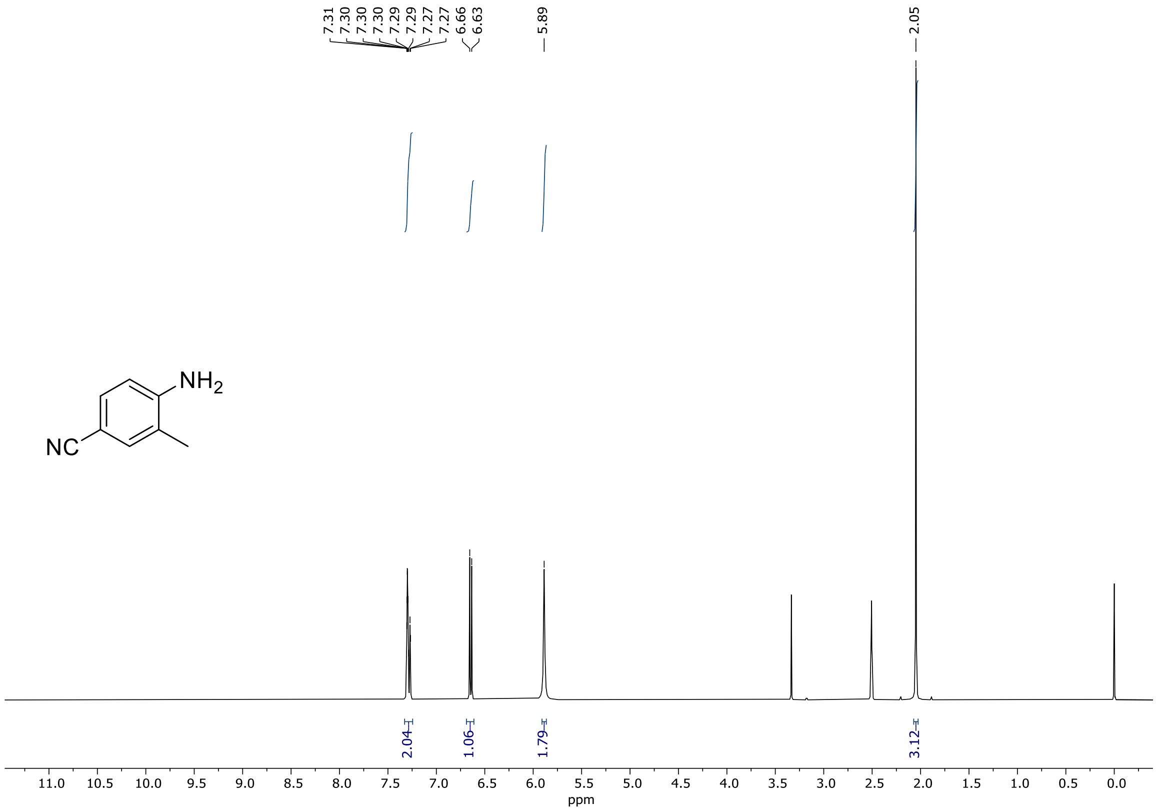

Figure S35: ^{1}H-NMR spectrum of compound **32b**.

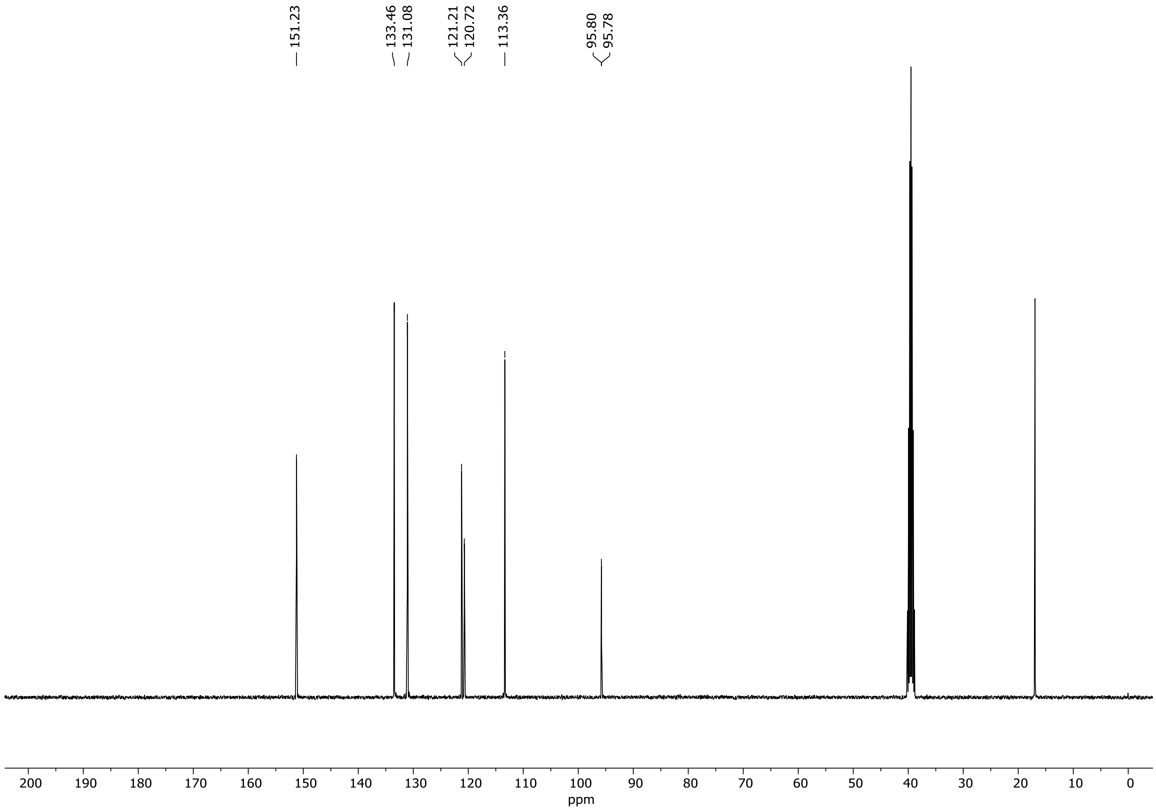

Figure S36: ^{13}C-NMR spectrum of compound **32b**.

4-Amino-3-fluorobenzonitrile (**32c**)

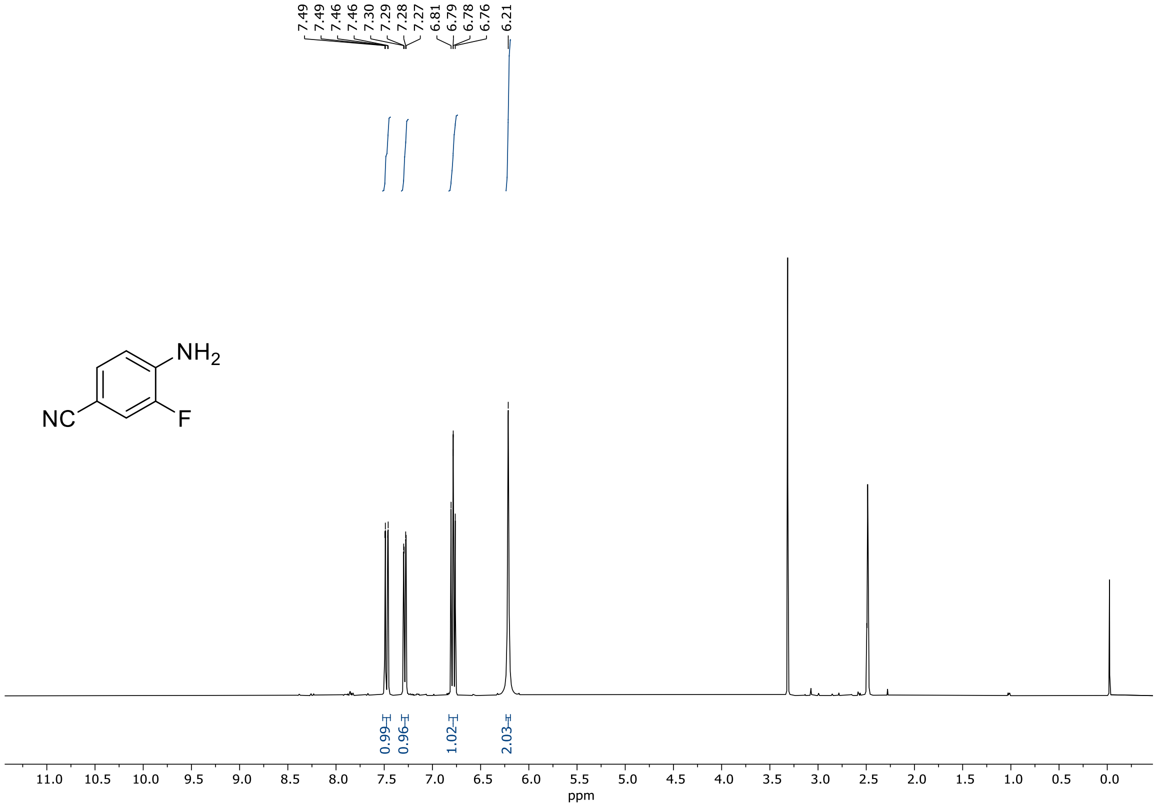

Figure S37: ^{1}H-NMR spectrum of compound **32c**.

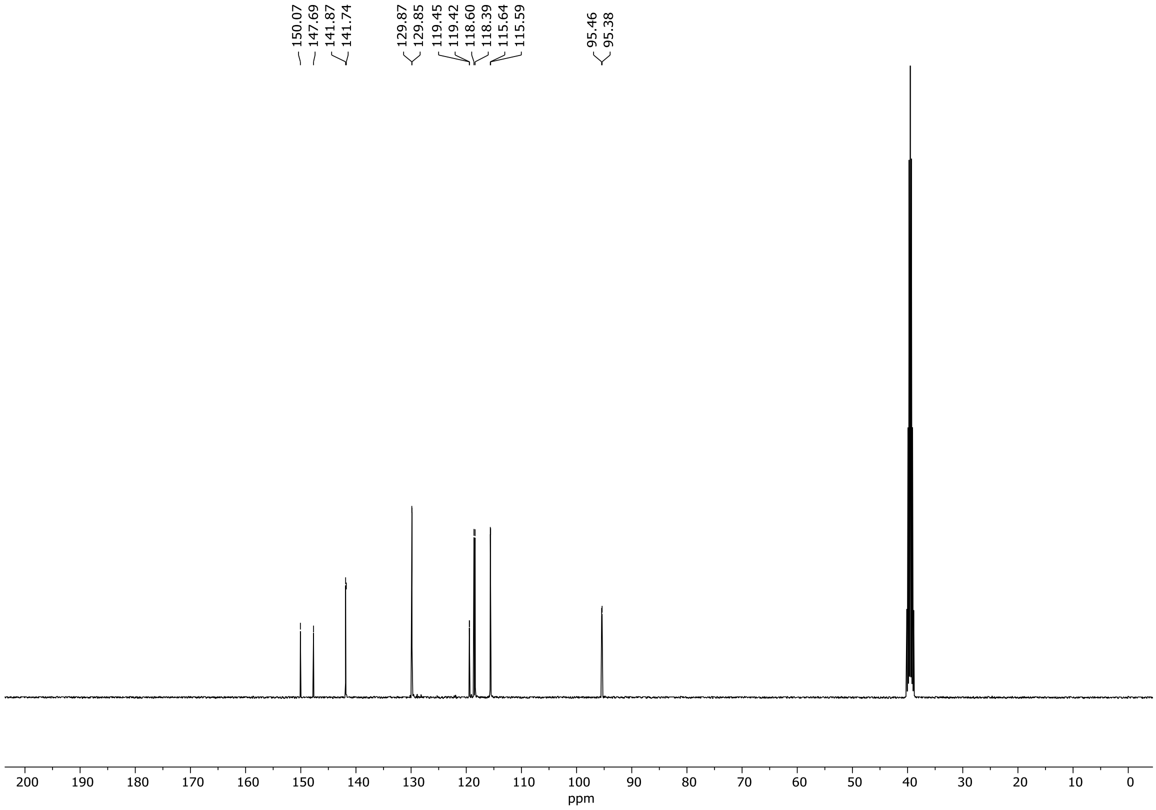

Figure S38: ^{13}C-NMR spectrum of compound **32c**.

4-Amino-3-(trifluoromethyl)benzonitrile (**32d**)

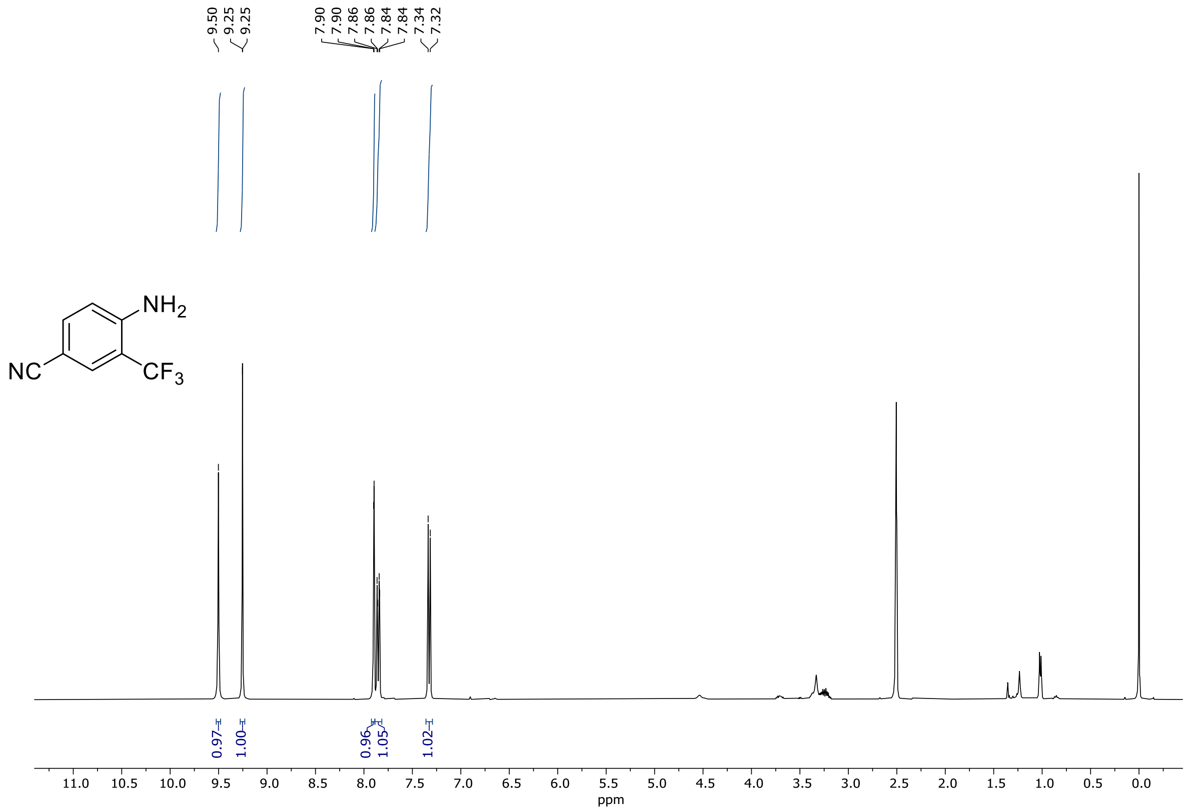

Figure S39: ^{1}H-NMR spectrum of compound **32d**.

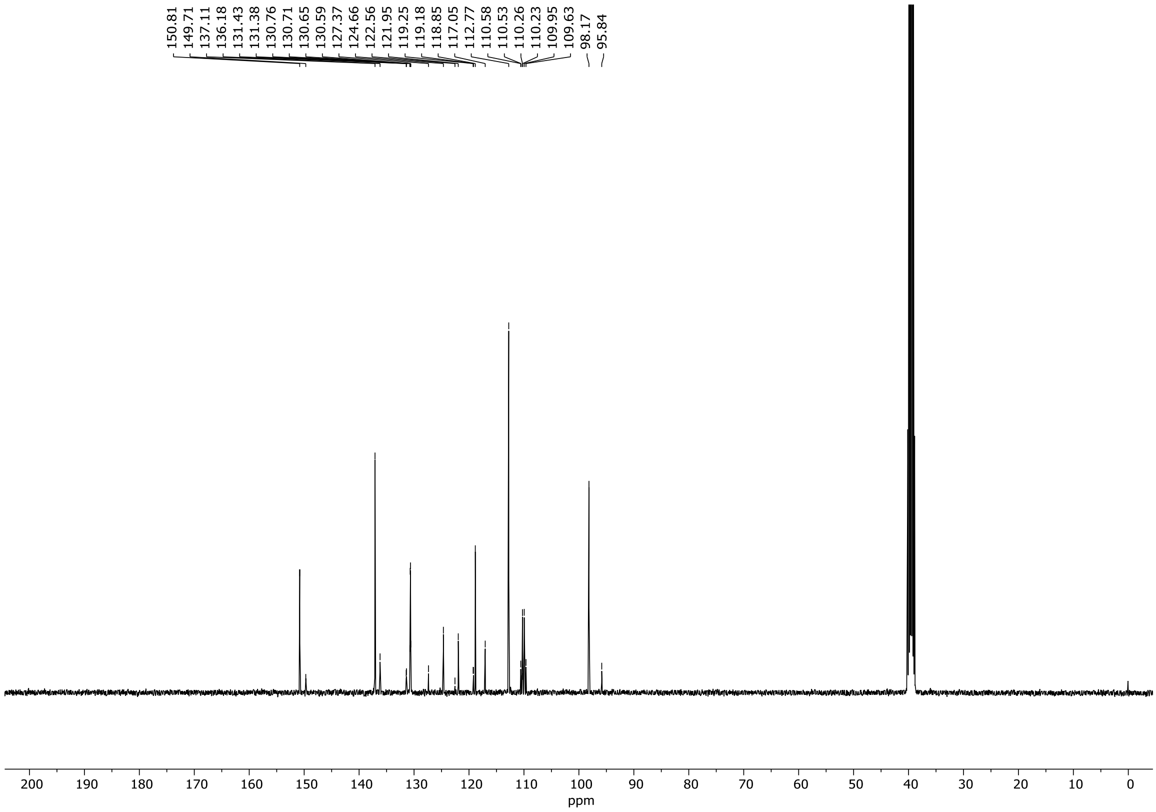

Figure S40: ^{13}C-NMR spectrum of compound **32d**.

N-(4-Cyano-2,6-dimethylphenyl)butyramide (**33a**)

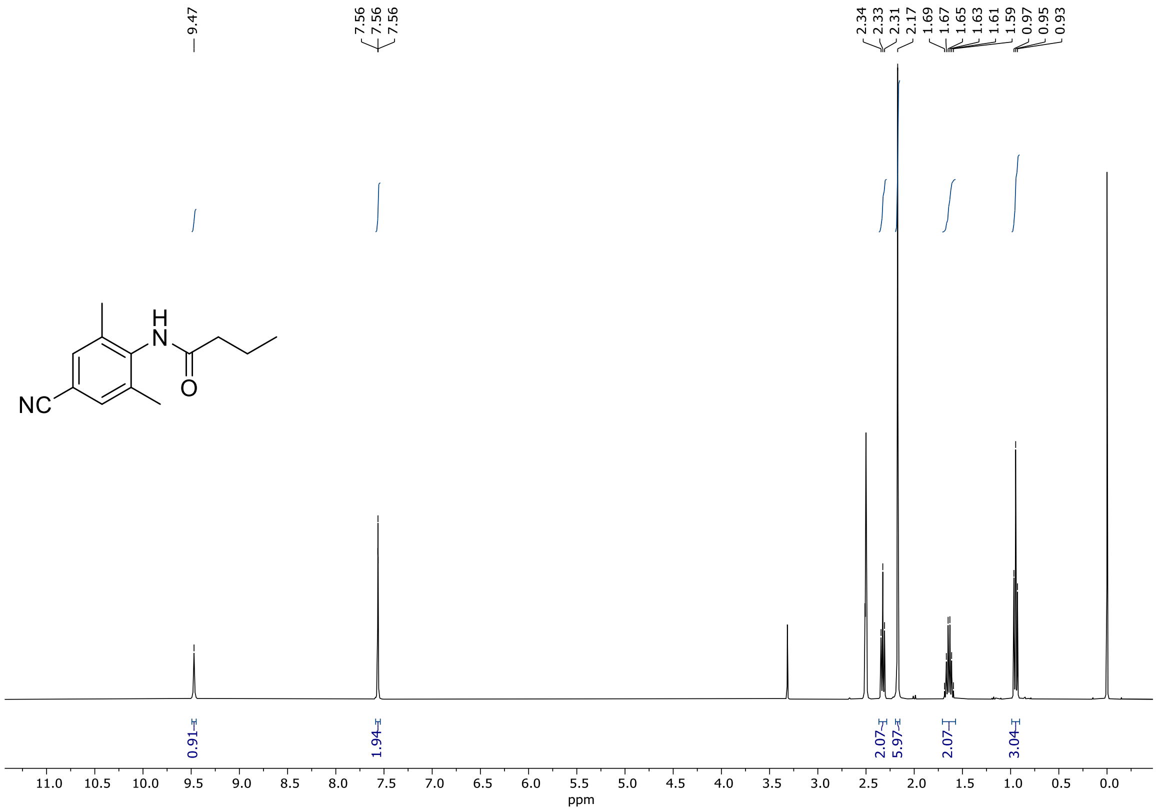

Figure S41: ^{1}H-NMR spectrum of compound **33a**.

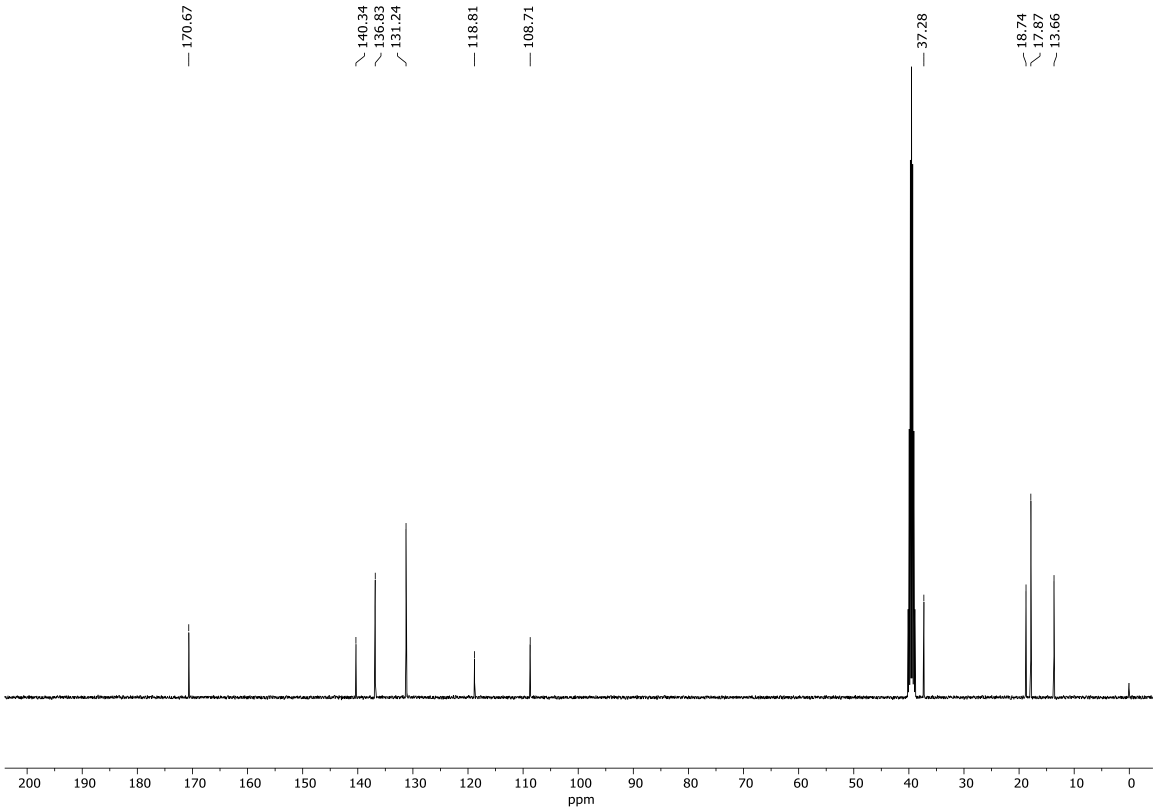

Figure S42: ^{13}C-NMR spectrum of compound **33a**.

N-(4-Cyano-2-methylphenyl)butyramide (**33b**)

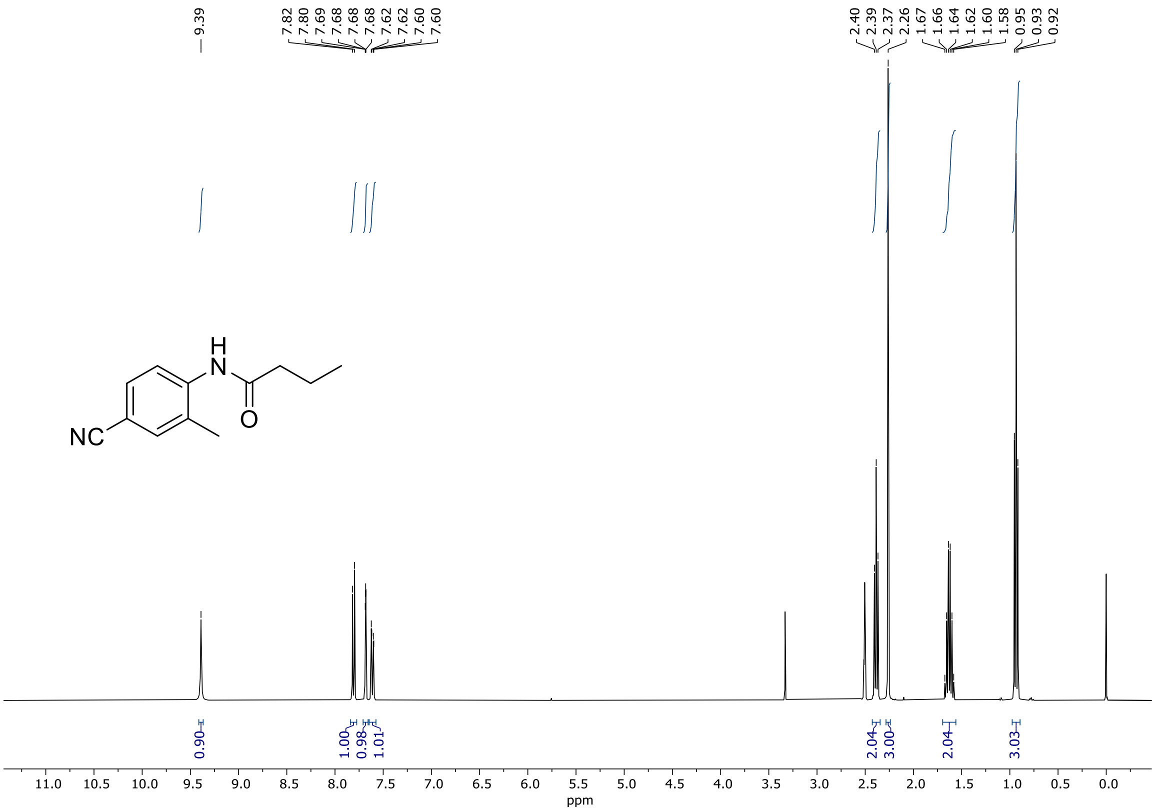

Figure S43: ^{1}H-NMR spectrum of compound **33b**.

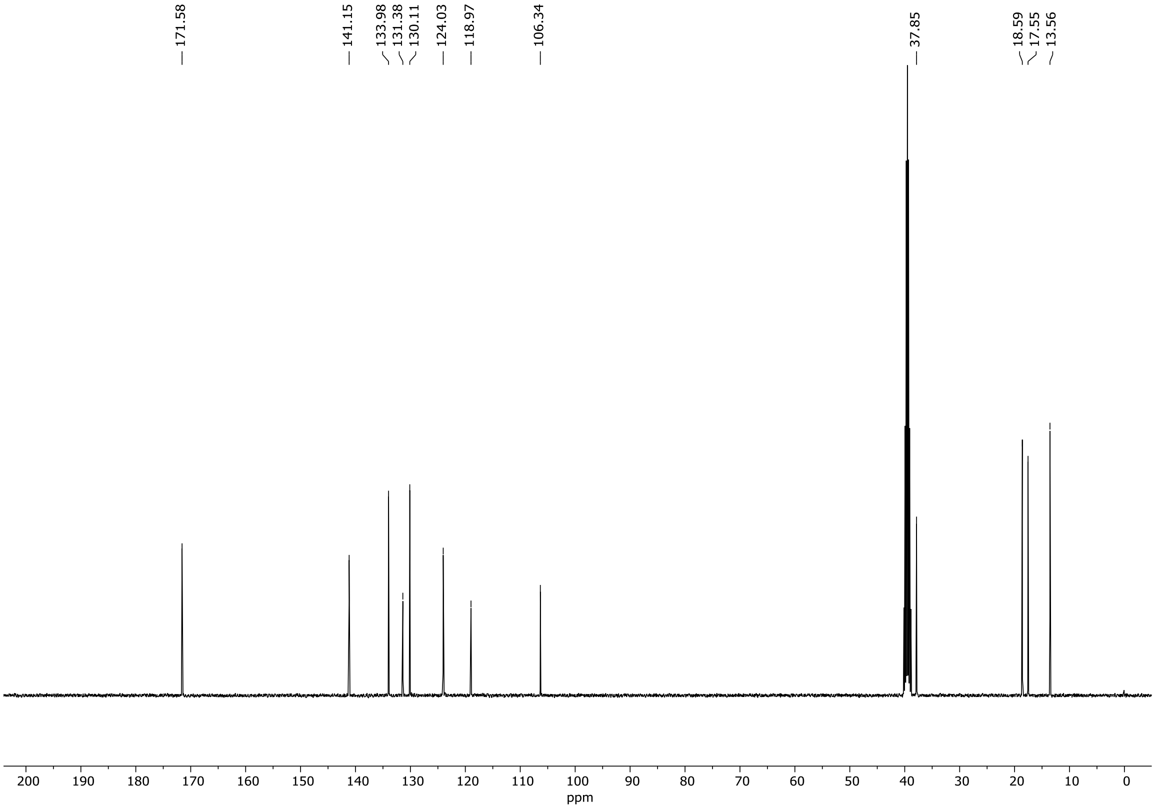

Figure S44: ^{13}C-NMR spectrum of compound **33b**.

N-(4-Cyano-2-fluorophenyl)butyramide (**33c**)

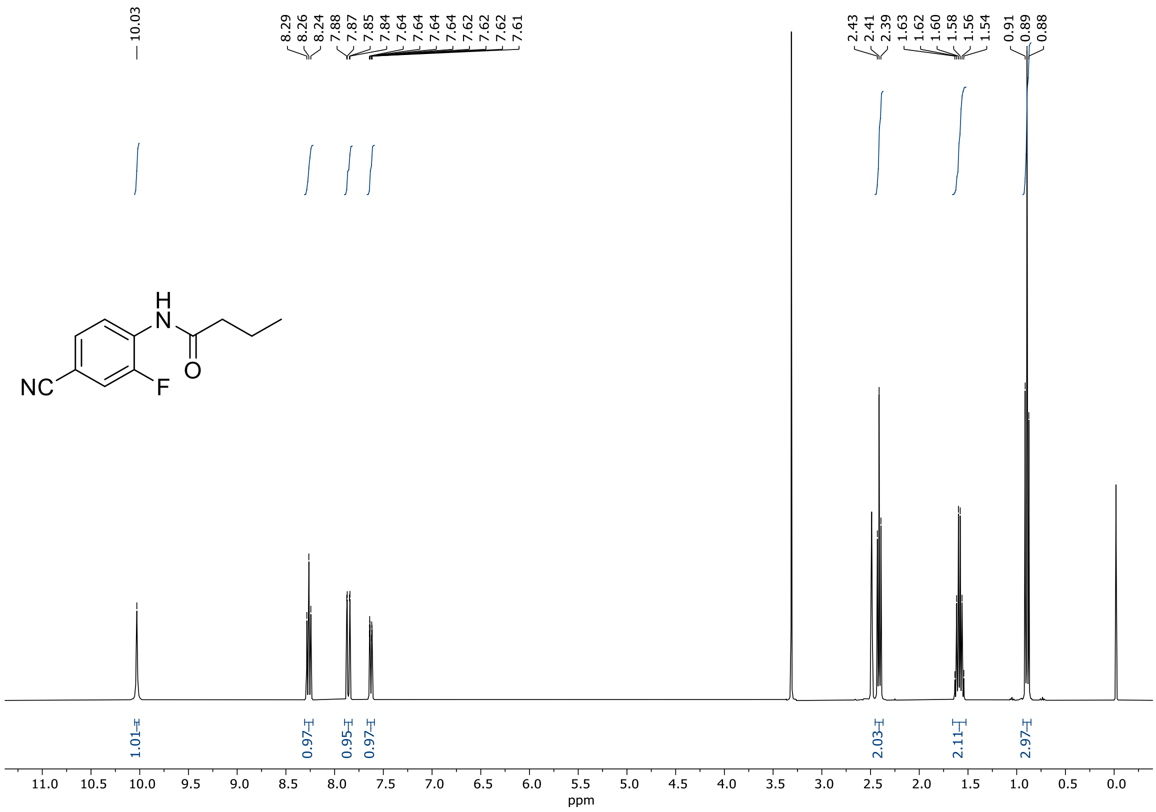

Figure S45: ^{1}H-NMR spectrum of compound **33c**.

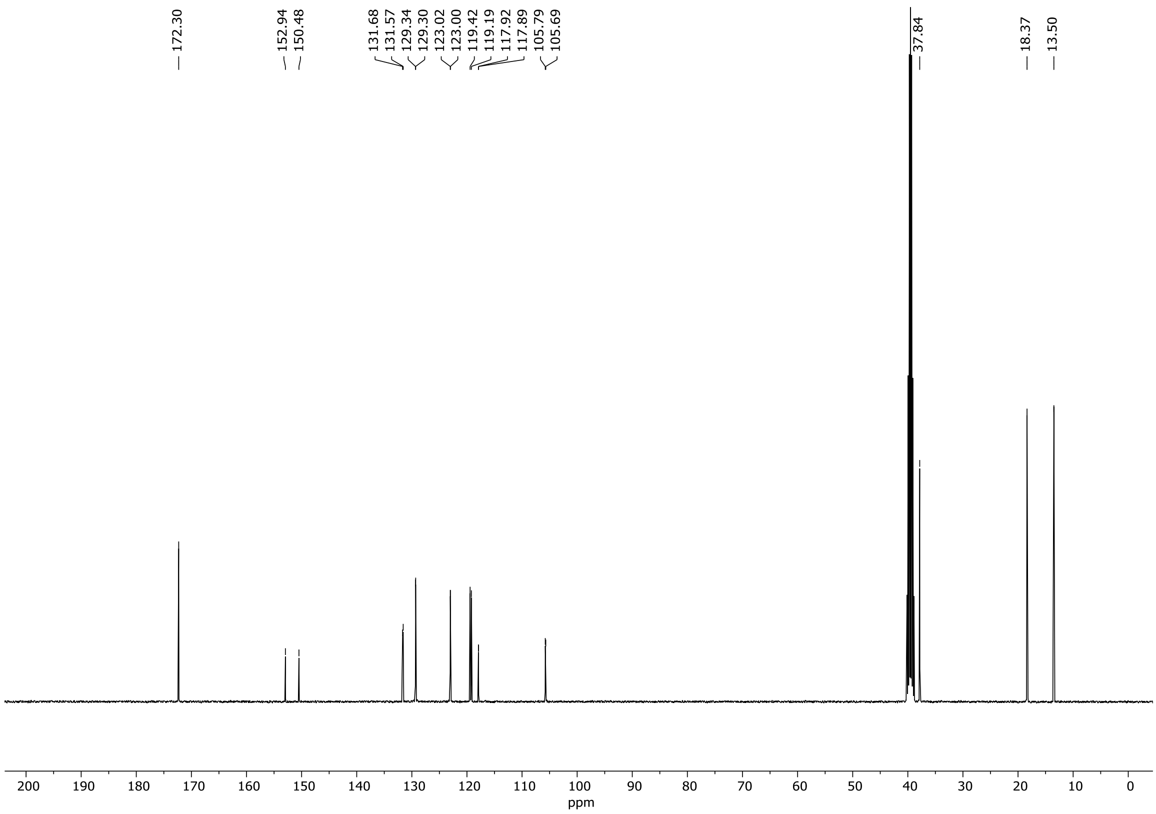

Figure S46: ^{13}C-NMR spectrum of compound **33c**.

N-[4-Cyano-2-(trifluoromethyl)phenyl]butyramide (**33d**)

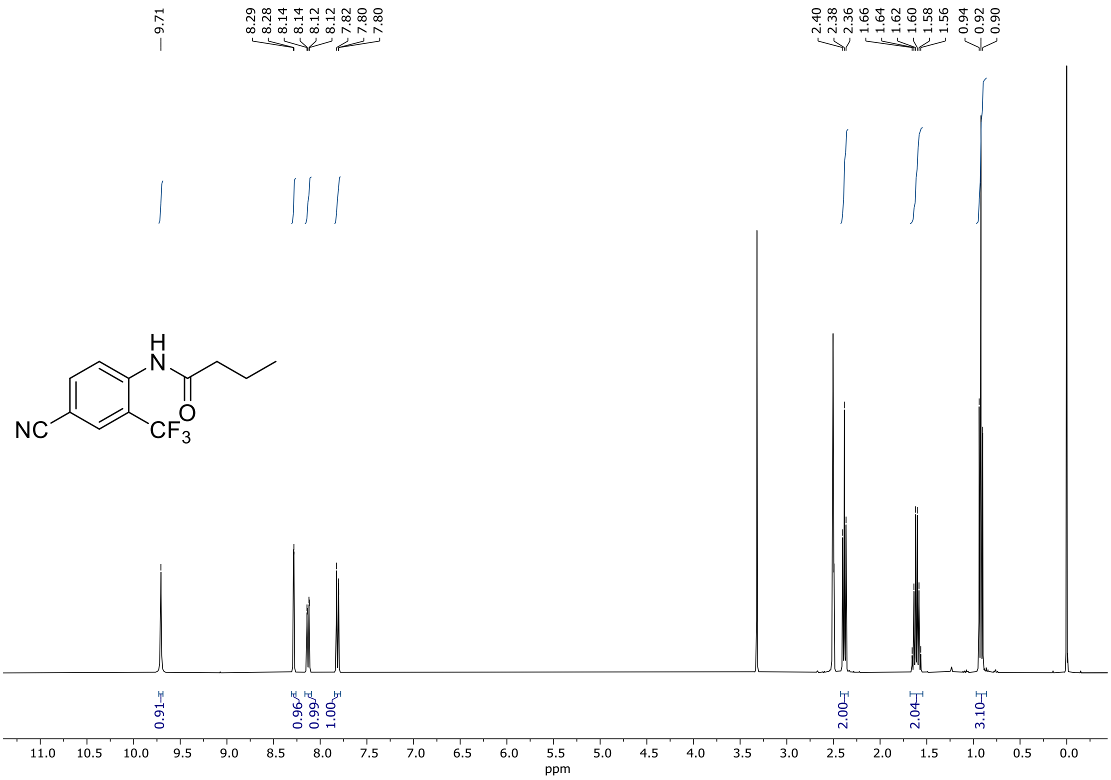

Figure S47: ^{1}H-NMR spectrum of compound **33d**.

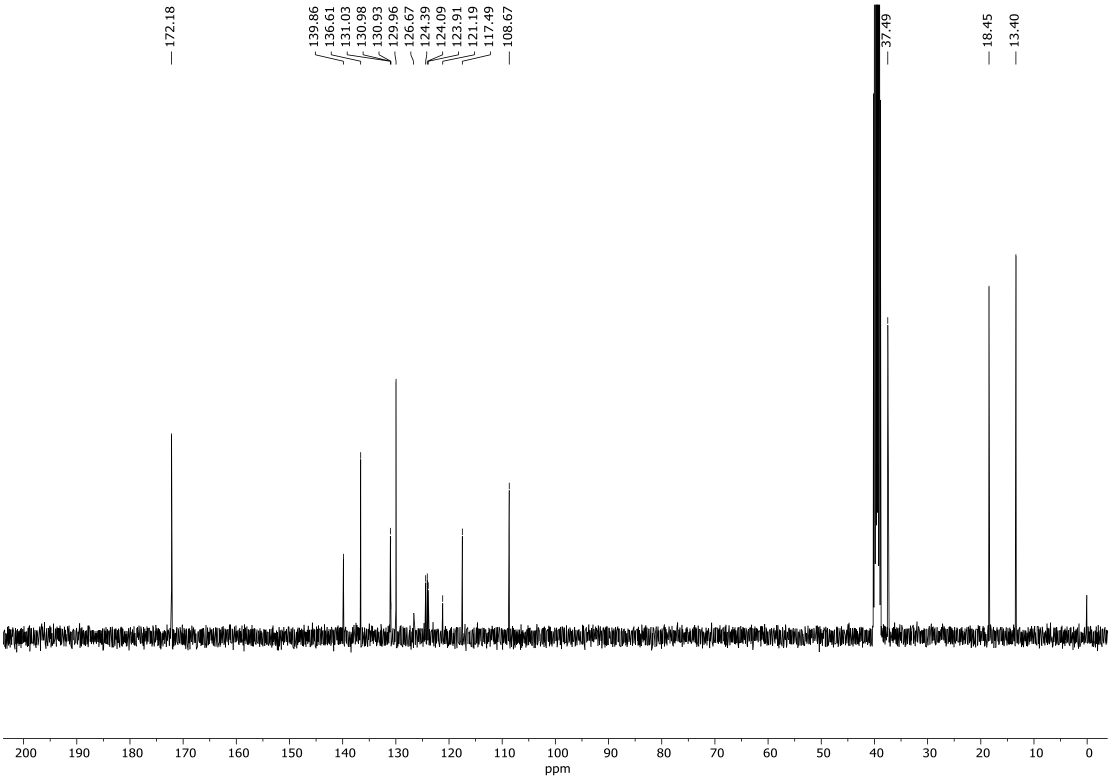

Figure S48: ^{13}C-NMR spectrum of compound **33d**.

N-(4-Cyano-2-methylphenyl)nicotinamide (**33e**)

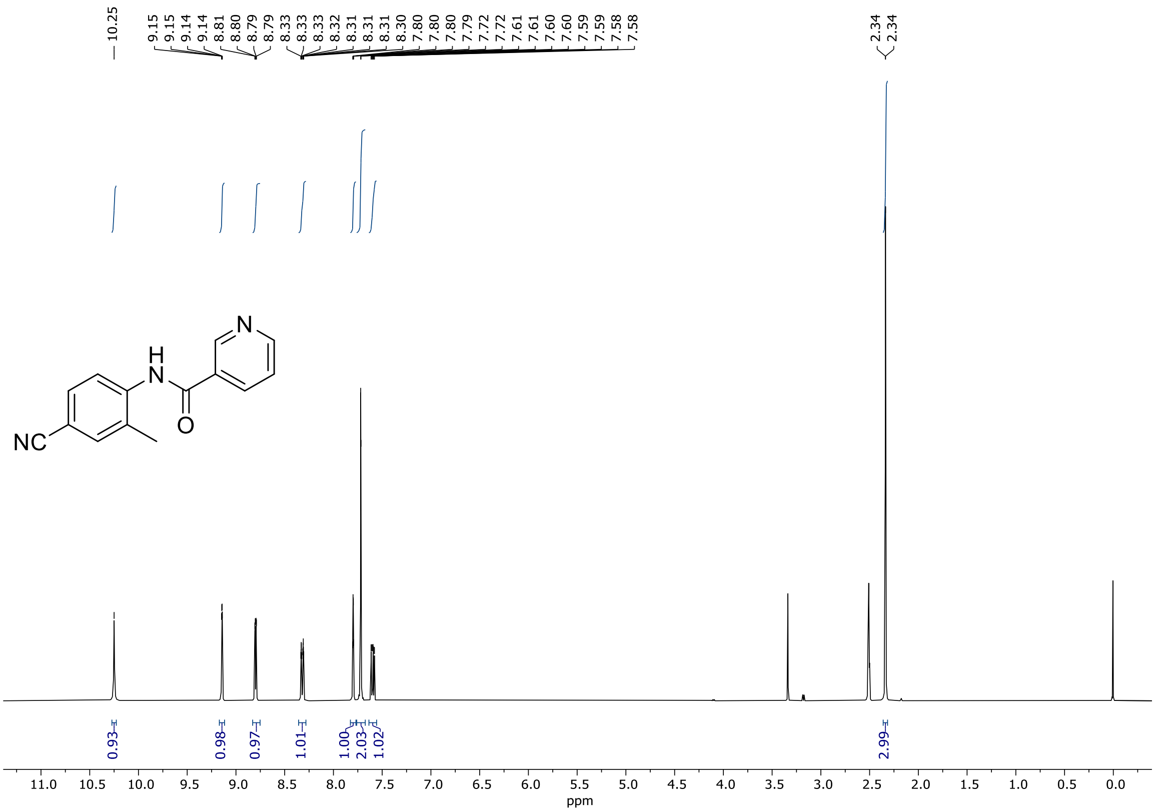

Figure S49: ^{1}H-NMR spectrum of compound **33e**.

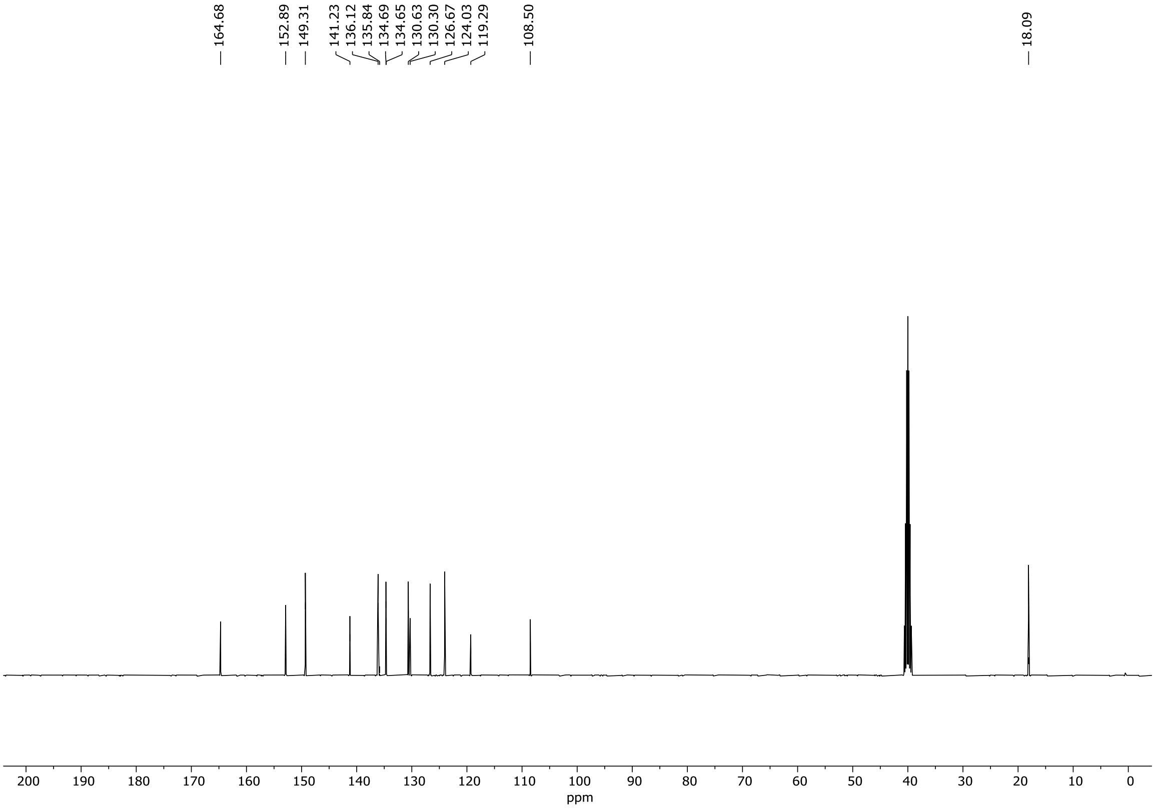

Figure S50: ^{13}C-NMR spectrum of compound **33e**.

N-(4-Formyl-2,6-dimethylphenyl)butyramide (**34a**)

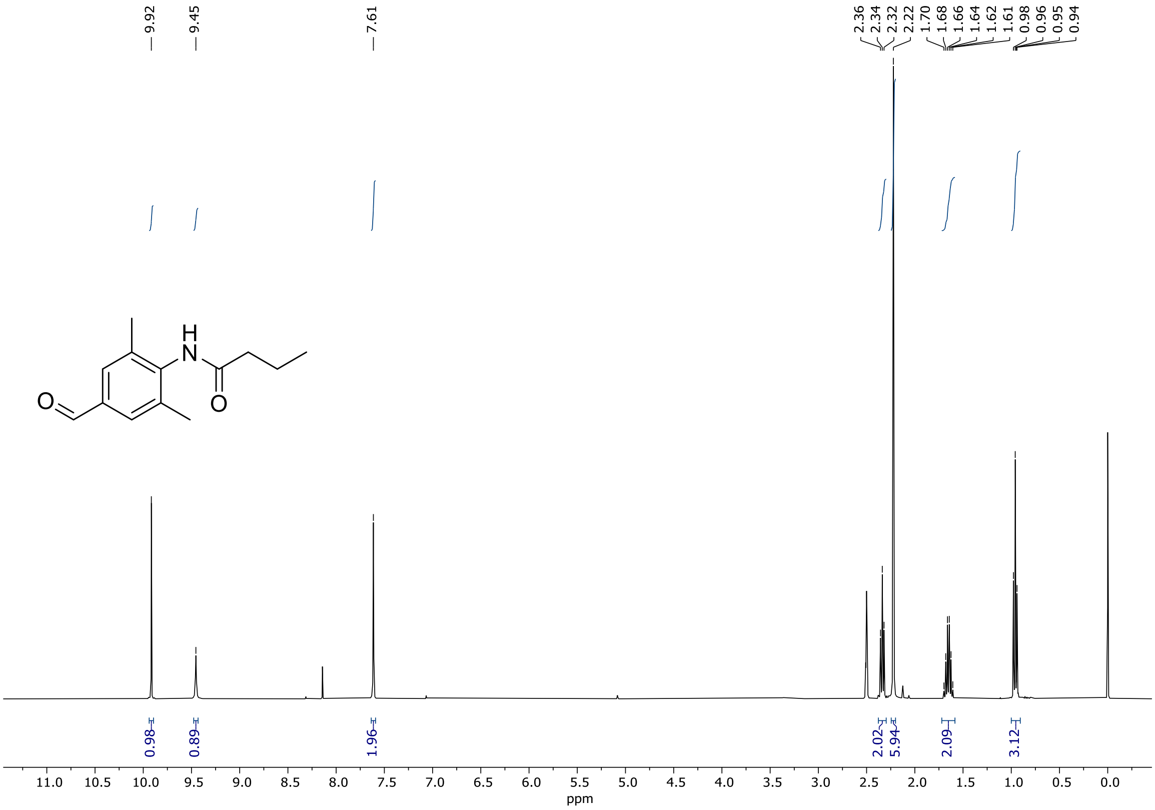

Figure S51: ^{1}H-NMR spectrum of compound **34a**.

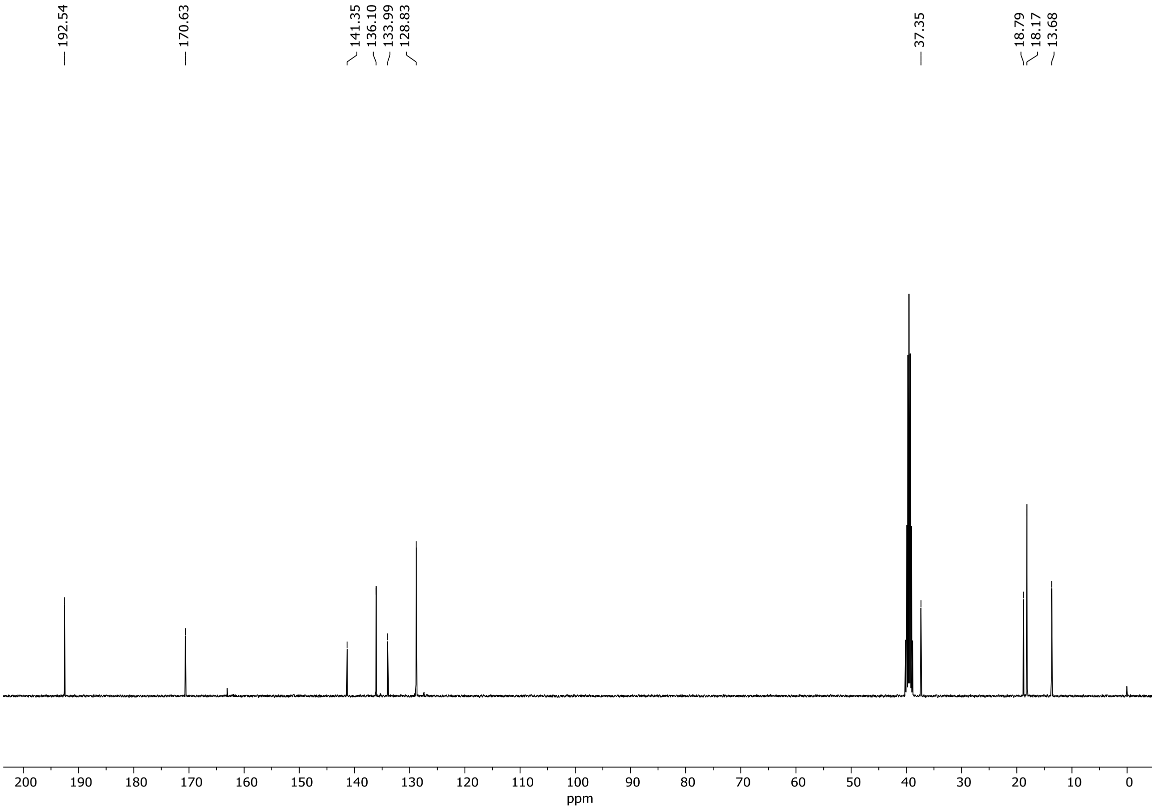

Figure S52: ^{13}C-NMR spectrum of compound **34a**.

N-(4-Formyl-2-methylphenyl)butyramide (**34b**)

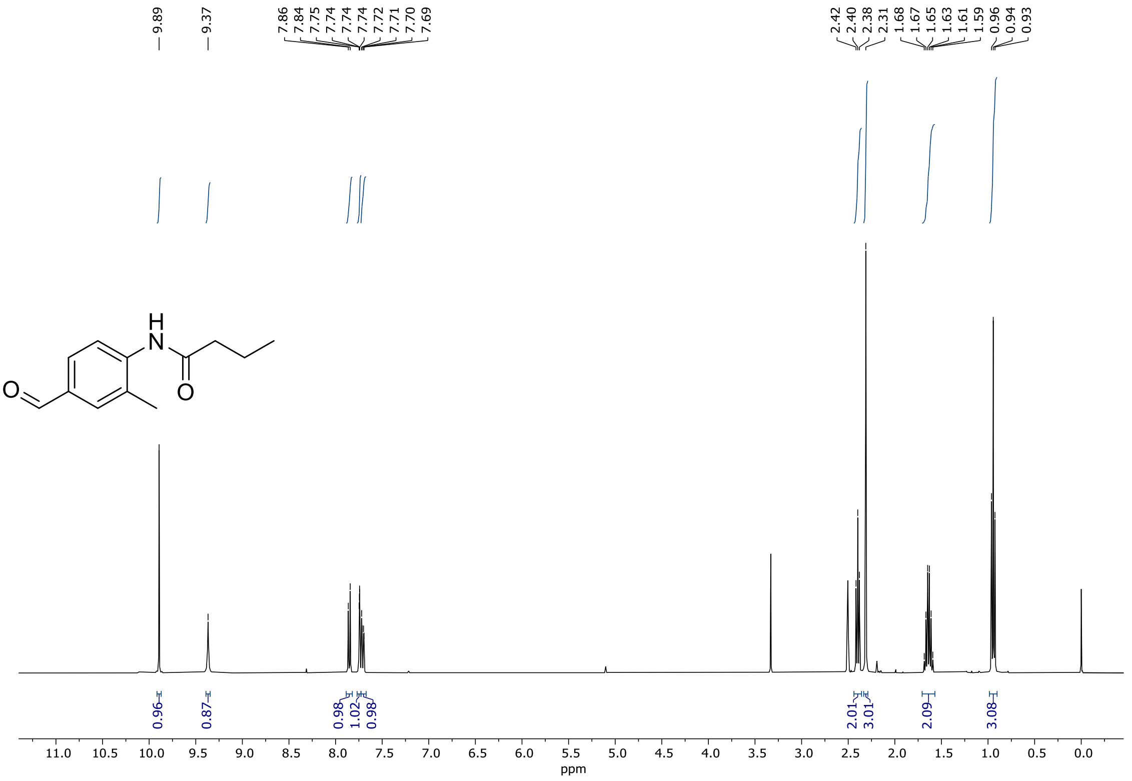

Figure S53: ^{1}H-NMR spectrum of compound **34b**.

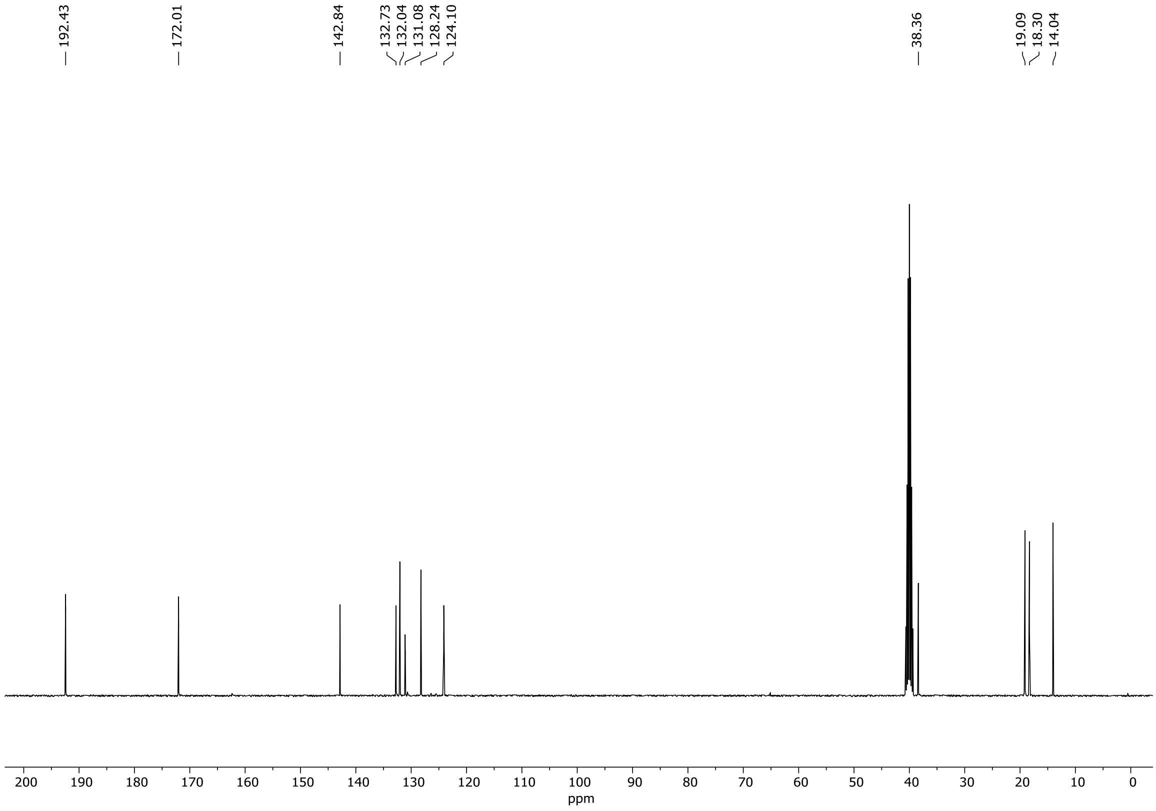

Figure S54: ^{13}C-NMR spectrum of compound **34b**.

N-(2-Fluoro-4-formylphenyl)butyramide (**34c**)

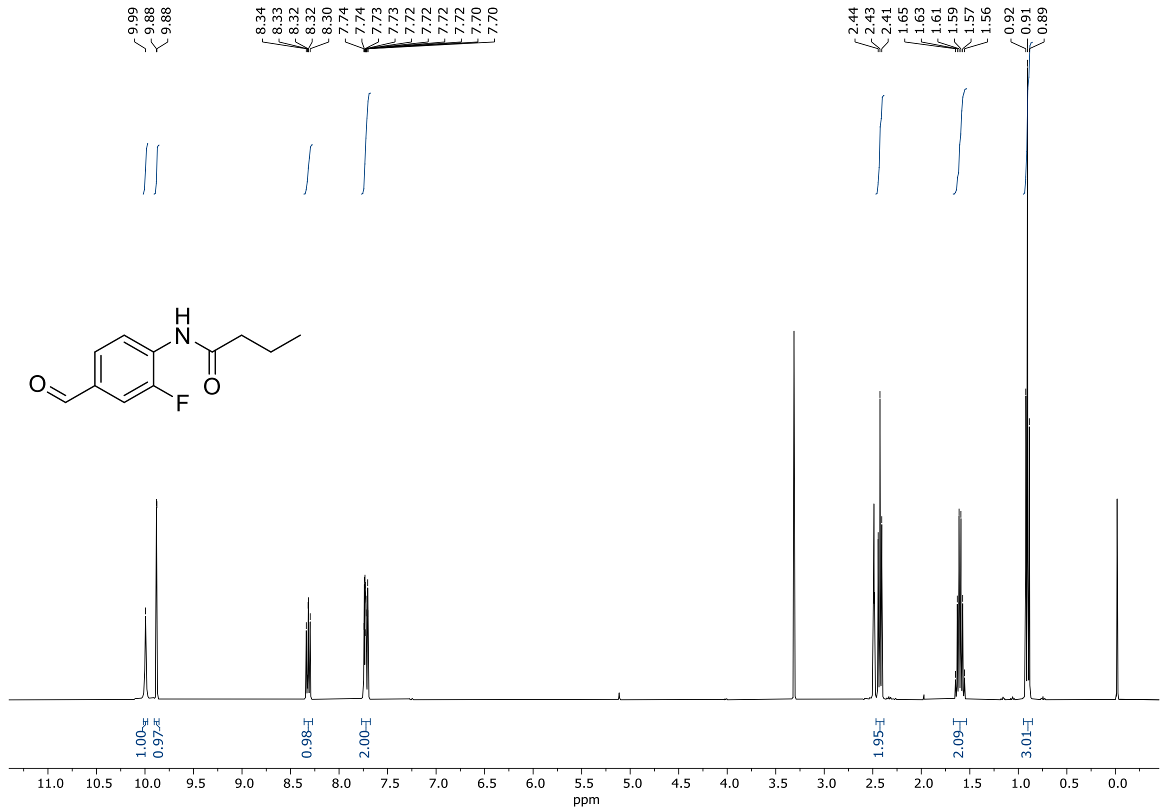

Figure S55: ^{1}H-NMR spectrum of compound **34c**.

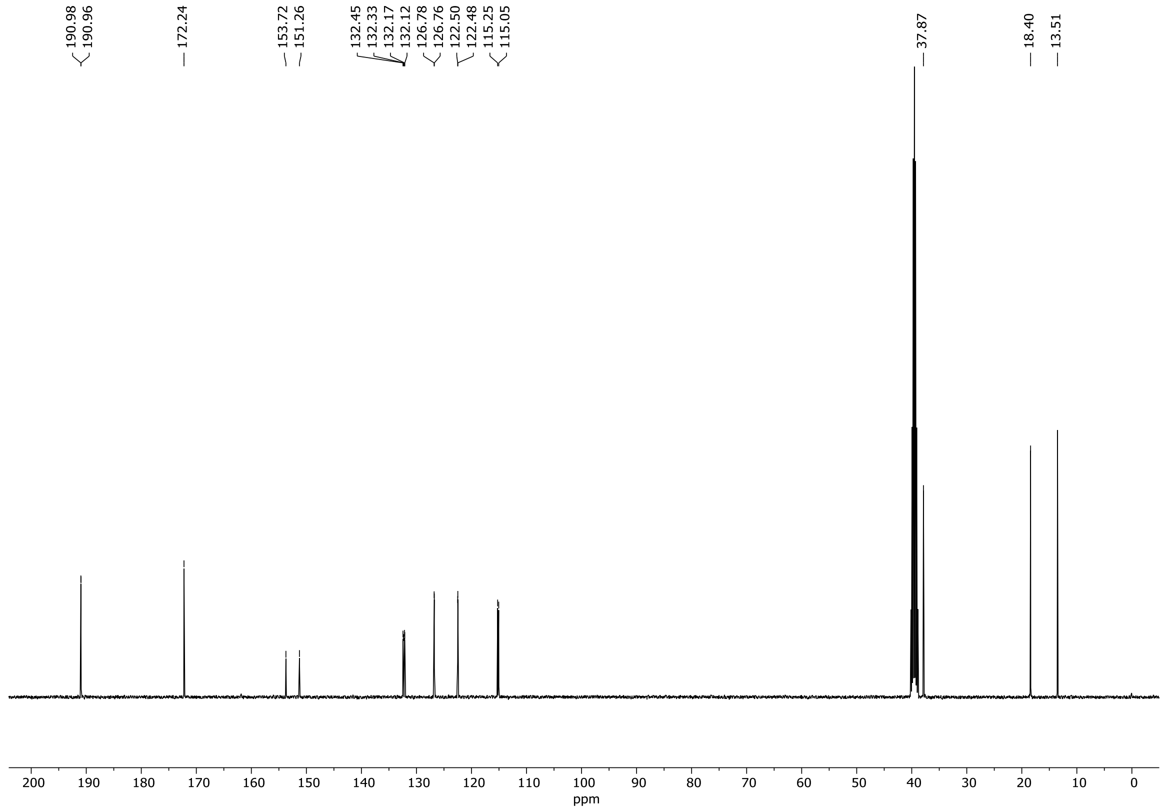

Figure S56: ^{13}C-NMR spectrum of compound **34c**.

N-[4-Formyl-2-(trifluoromethyl)phenyl]butyramide (**34d**)

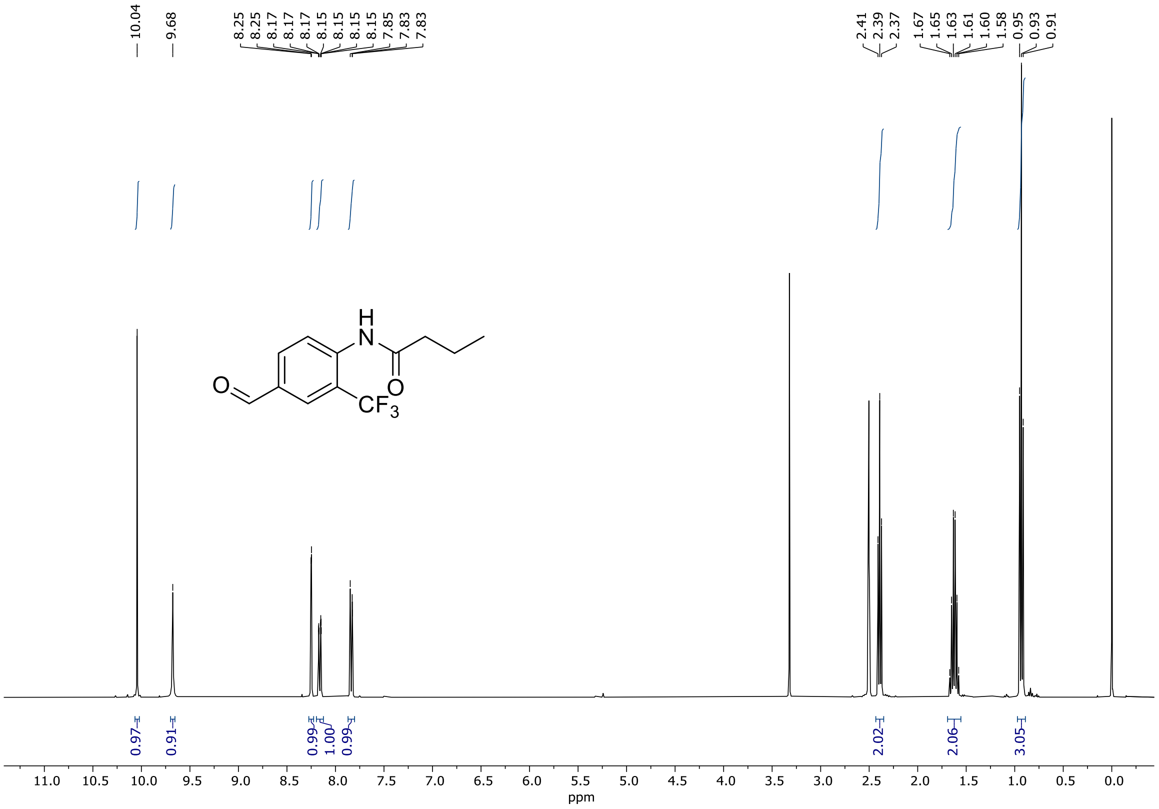

Figure S57: ^{1}H-NMR spectrum of compound **34d**.

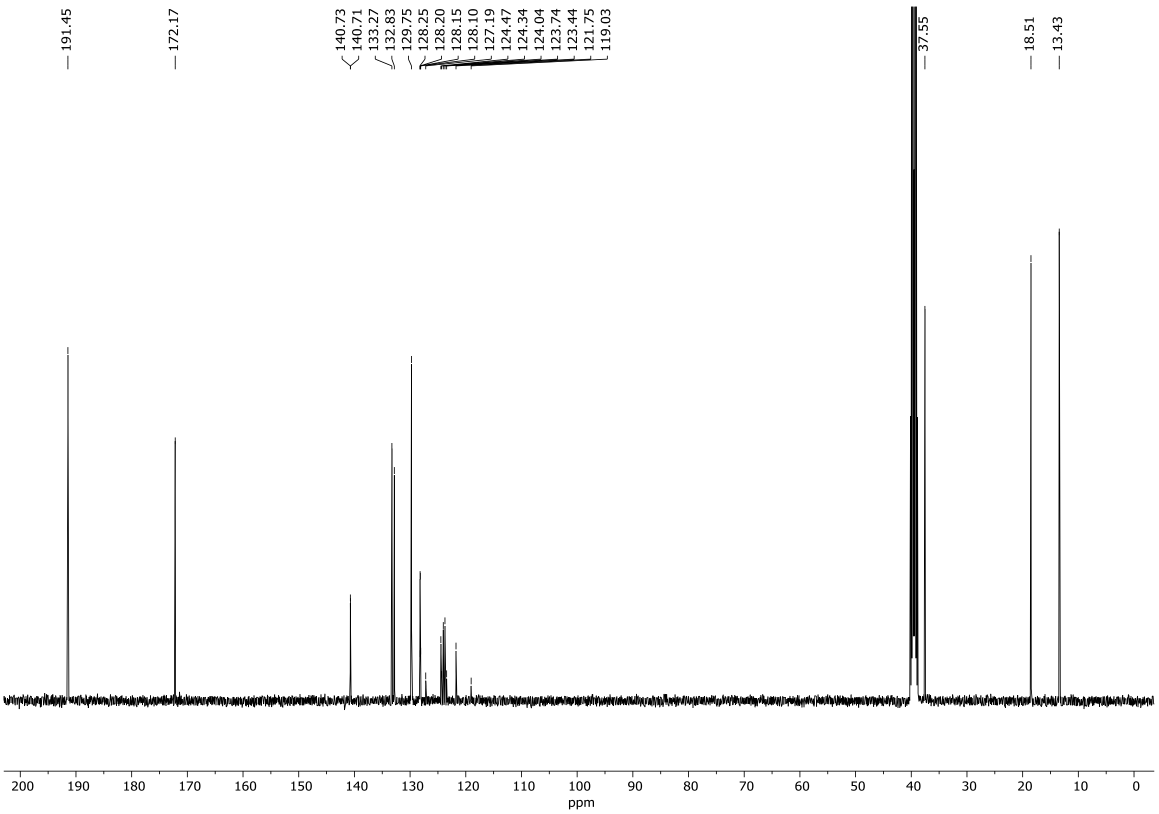

Figure S58: ^{13}C-NMR spectrum of compound **34d**.

N-(4-Formyl-2-methylphenyl)nicotinamide (**34e**)

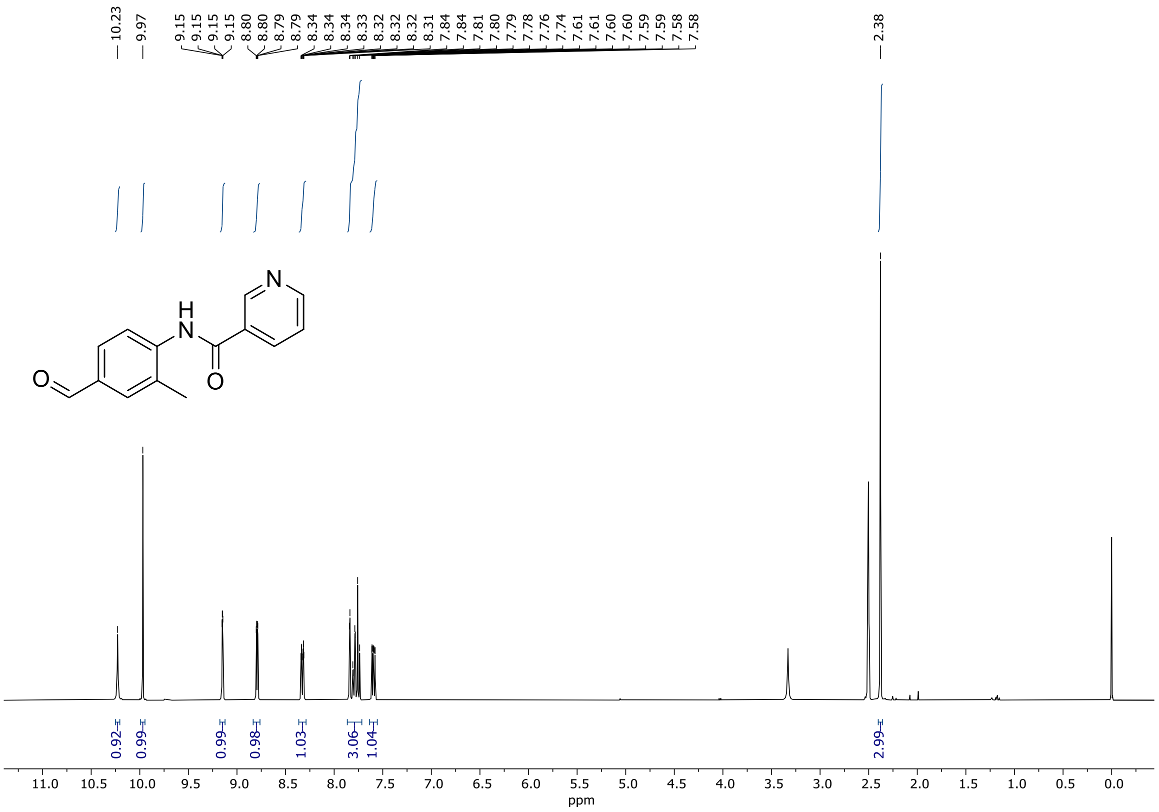

Figure S59: ^{1}H-NMR spectrum of compound **34e**.

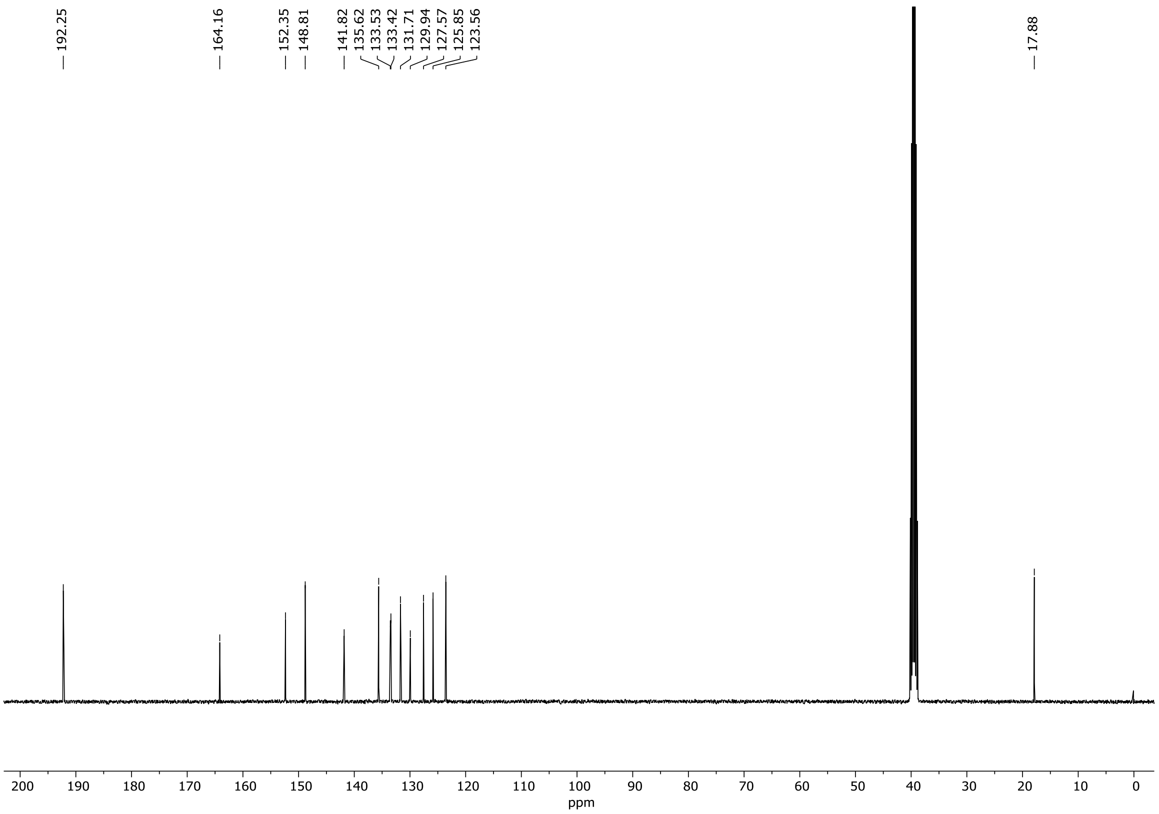

Figure S60: ^{13}C-NMR spectrum of compound **34e**.

N-(4-{[(4-Fluorophenyl)amino]methyl}-2,6-dimethylphenyl)butyramide (**35a**)

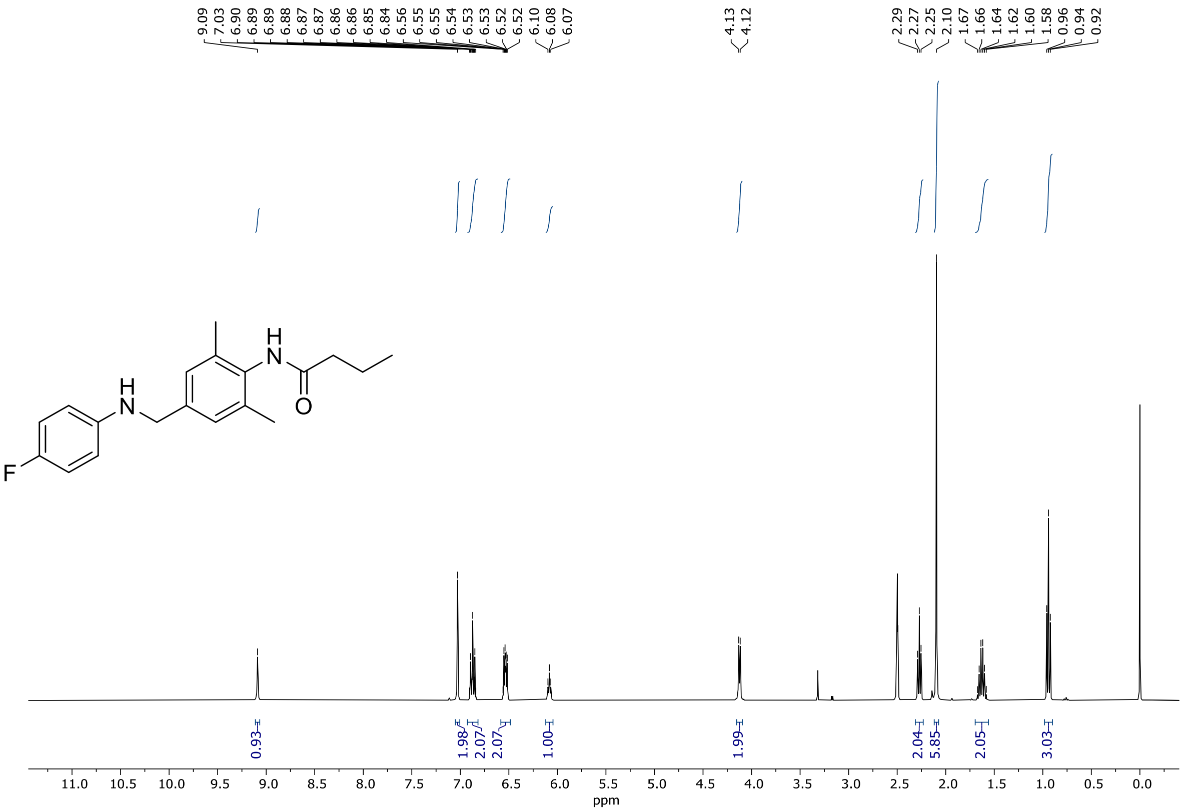

Figure S61: ^{1}H-NMR spectrum of compound **35a**.

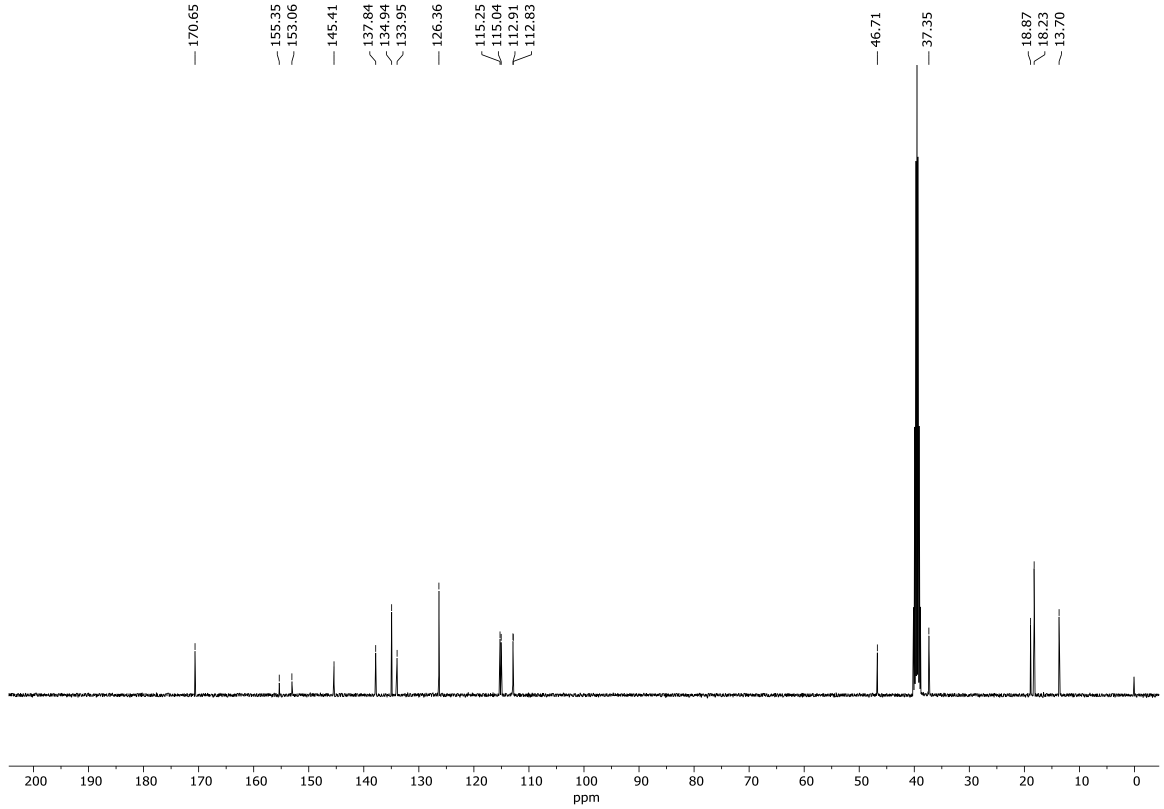

Figure S62: ^{13}C-NMR spectrum of compound **35a**.

N-(4-{[(4-Fluorophenyl)amino]methyl}-2-methylphenyl)butyramide (**35b**)

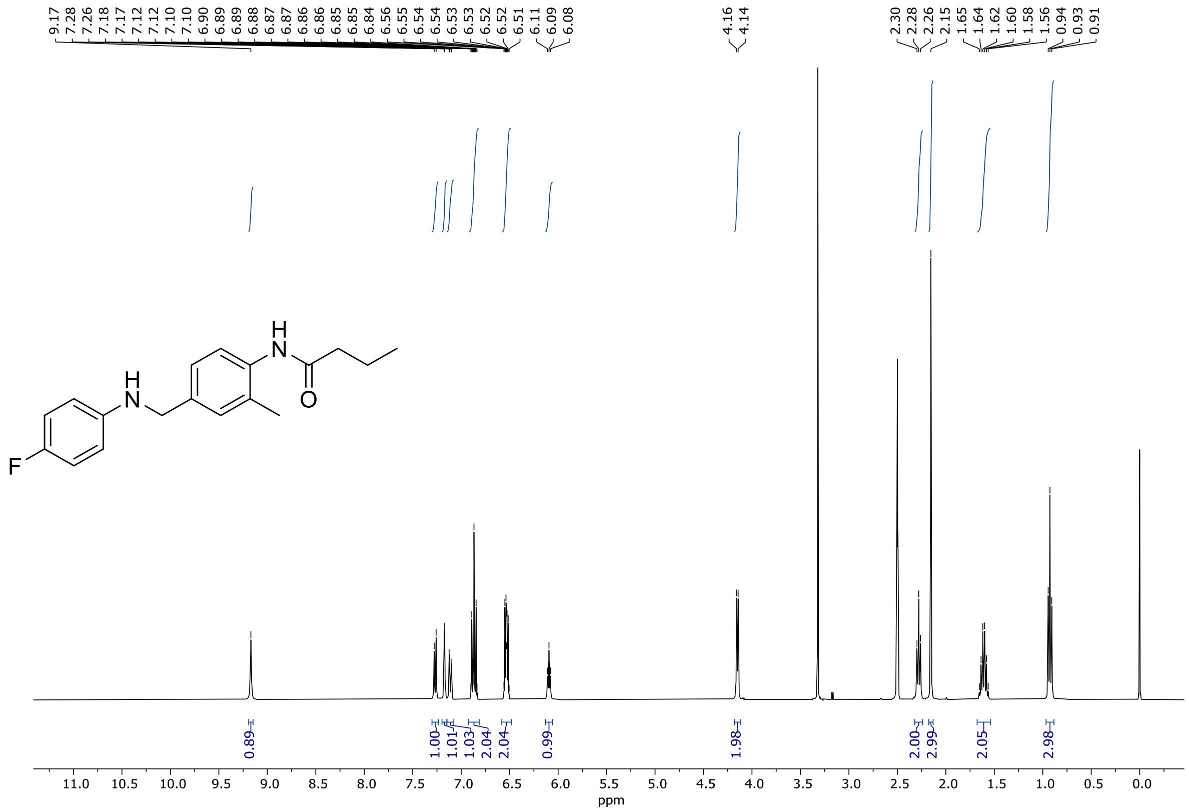

Figure S63: ^{1}H-NMR spectrum of compound **35b**.

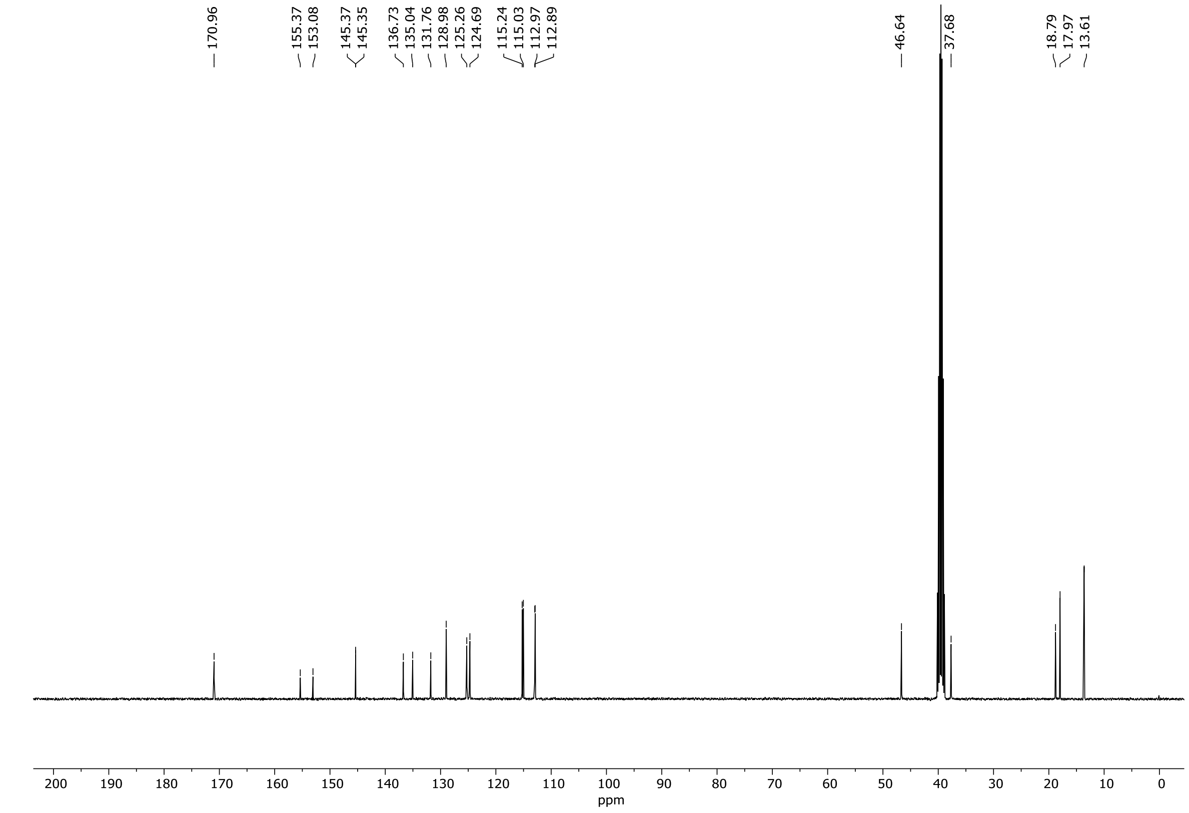

Figure S64: ^{13}C-NMR spectrum of compound **35b**.

N-(2-Fluoro-4-{[(4-fluorophenyl)amino]methyl}phenyl)butyramide (**35c**)

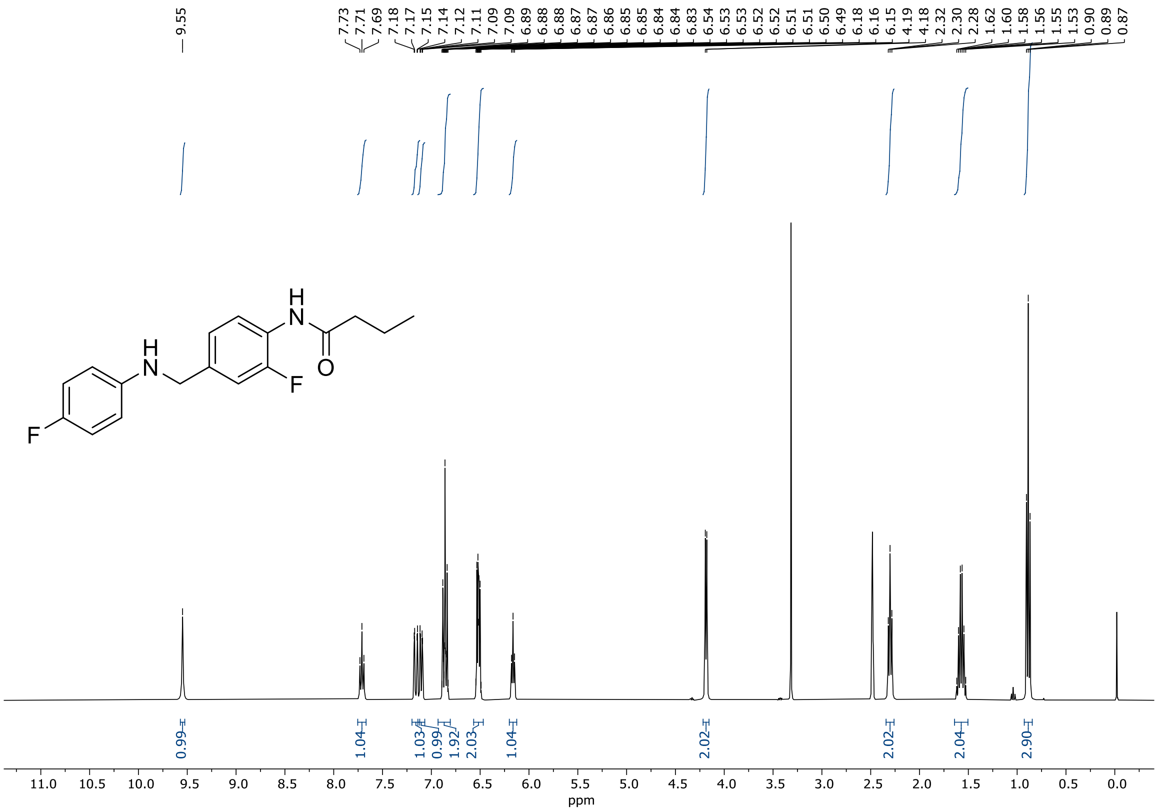

Figure S65: ^{1}H-NMR spectrum of compound **35c**.

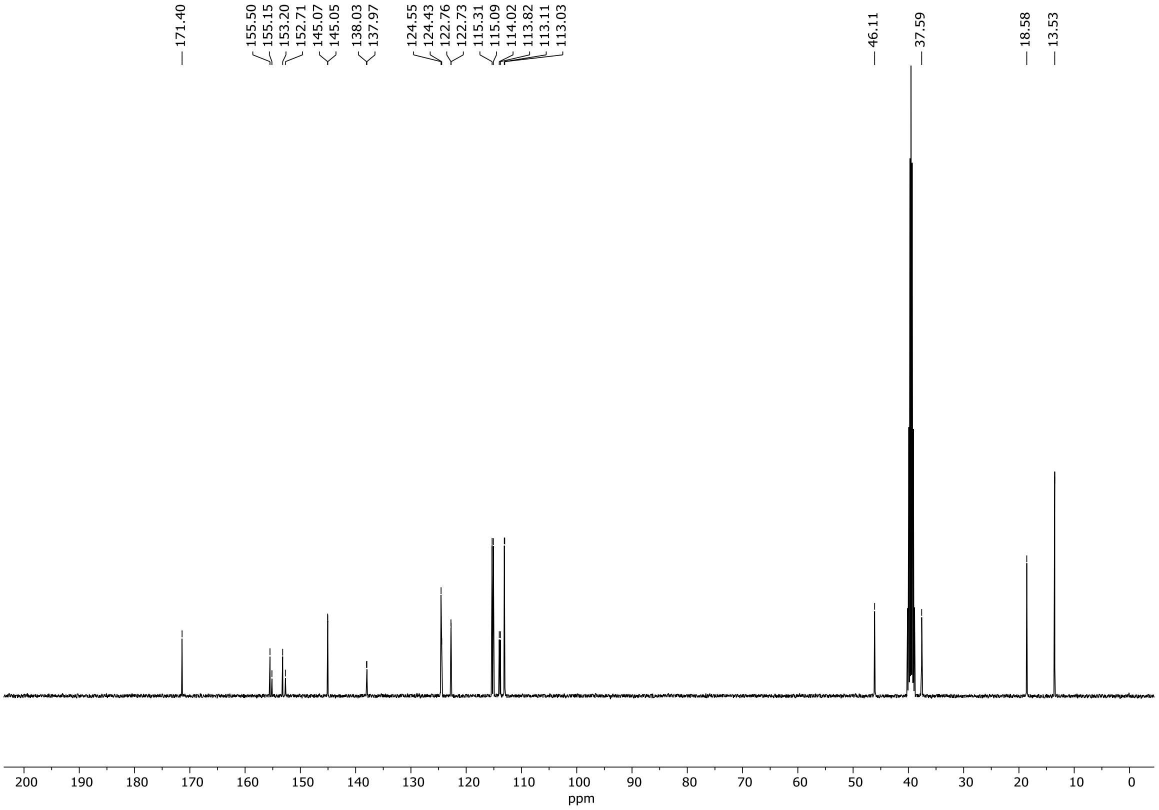

Figure S66: ^{13}C-NMR spectrum of compound **35c**.

N-(4-{[(4-Fluorophenyl)amino]methyl}-2-(trifluoromethyl)phenyl)butyramide hydrochloride (**35d**)

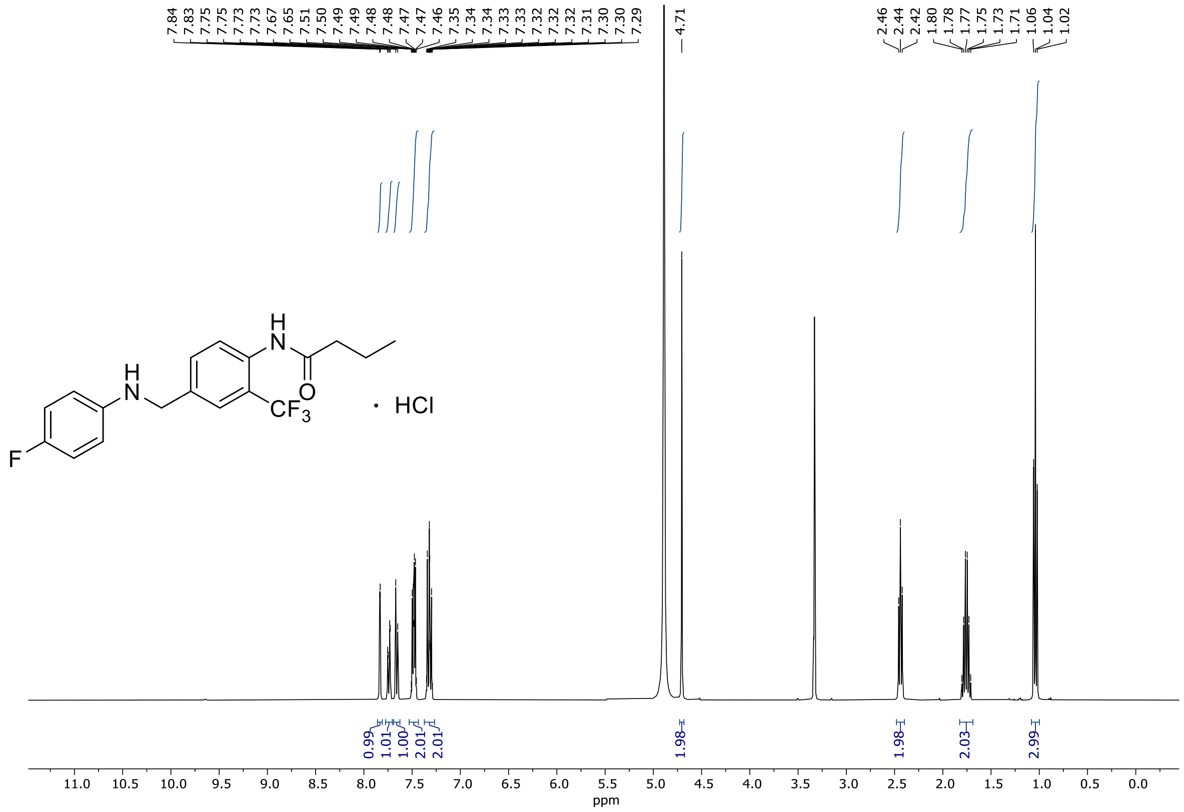

Figure S67: ^{1}H-NMR spectrum of compound **35d**.

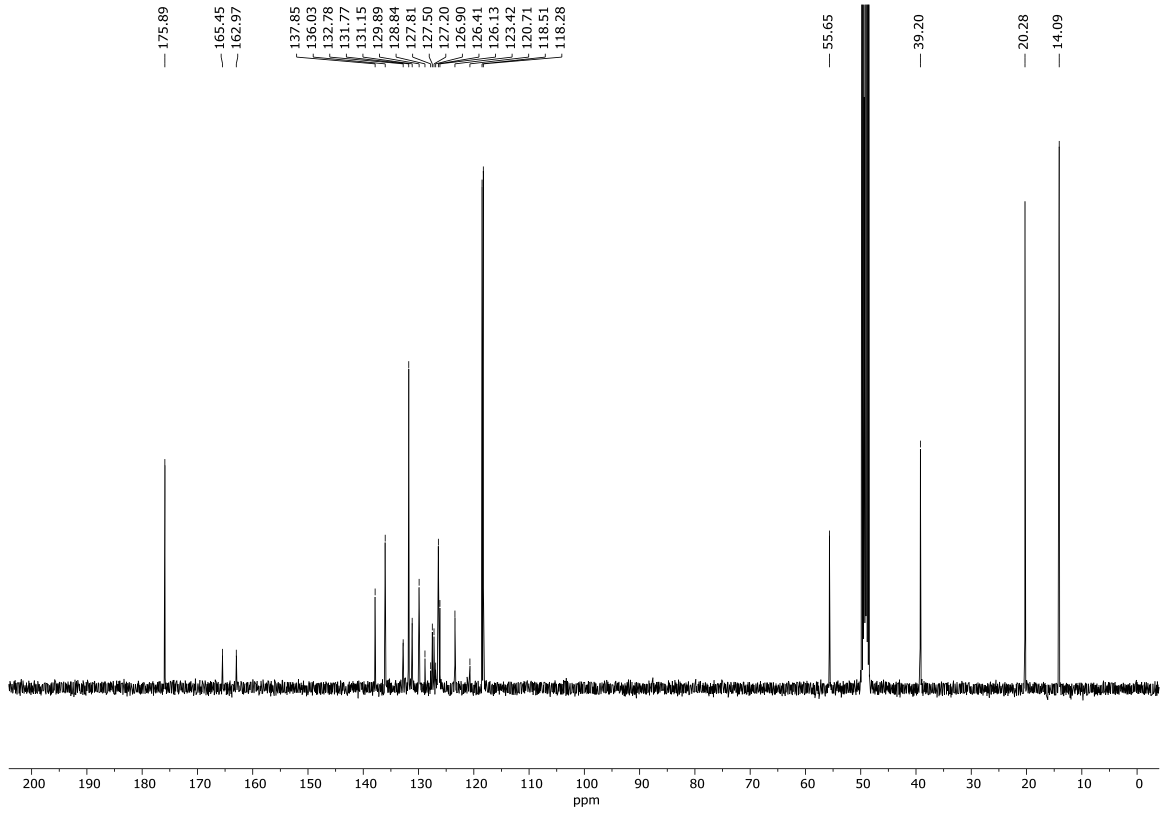

Figure S68: ^{13}C-NMR spectrum of compound **35d**.

N-(4-{[(2,4-Difluorophenyl)amino]methyl}-2-methylphenyl)nicotinamide (**35e**)

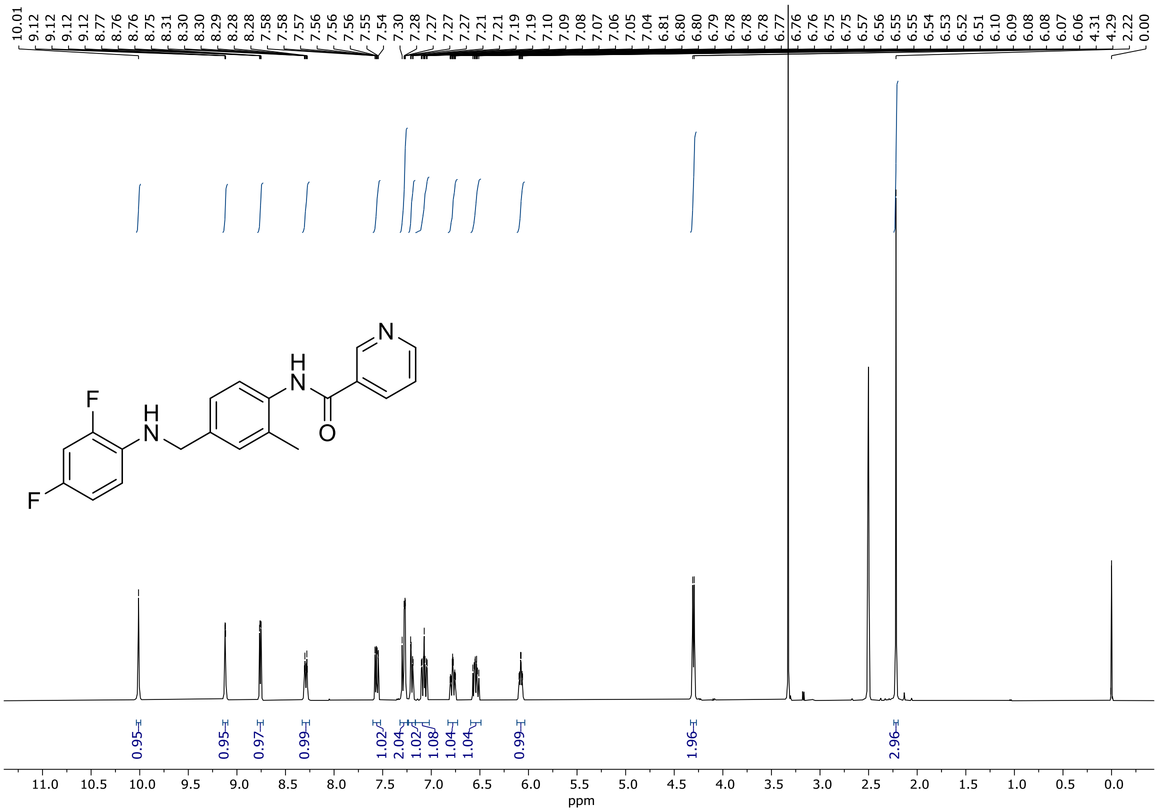

Figure S69: ^{1}H-NMR spectrum of compound **35e**.

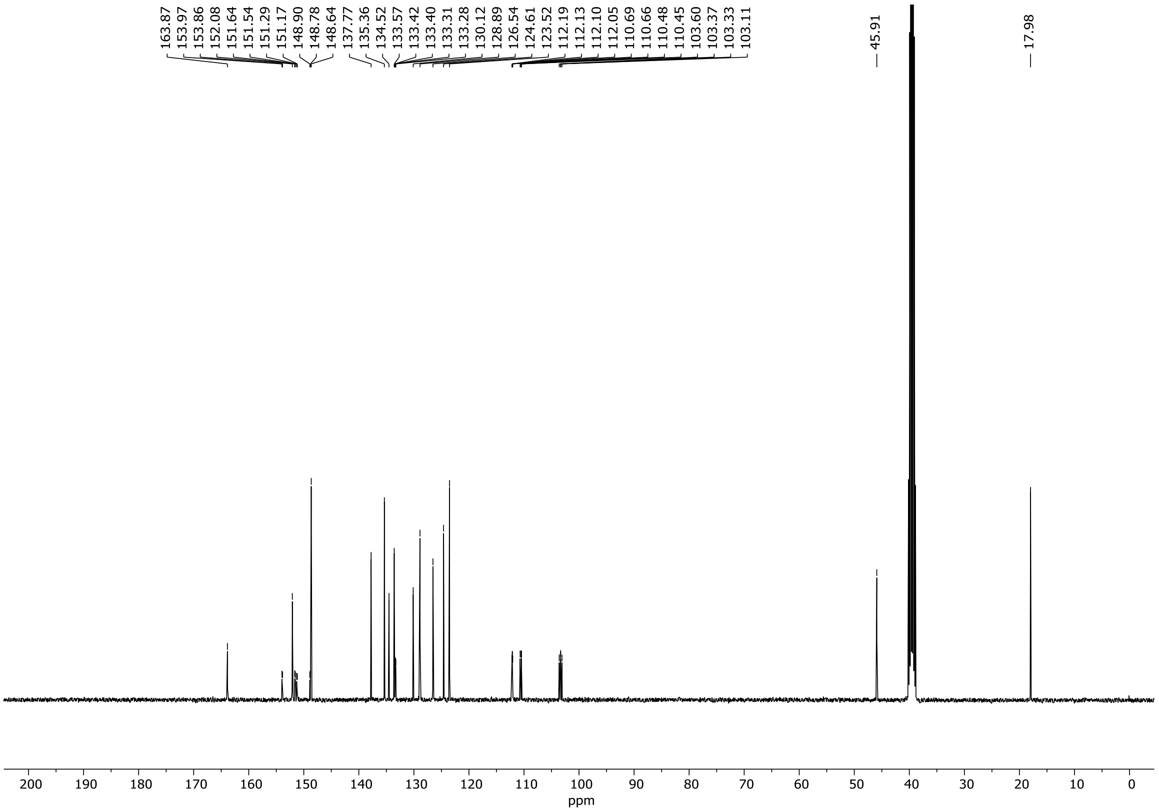

Figure S70: ^{13}C-NMR spectrum of compound **35e**.

N-(2-Methyl-4-{[(5-methylpyridin-2-yl)amino]methyl}phenyl)butyramide (**35f**)

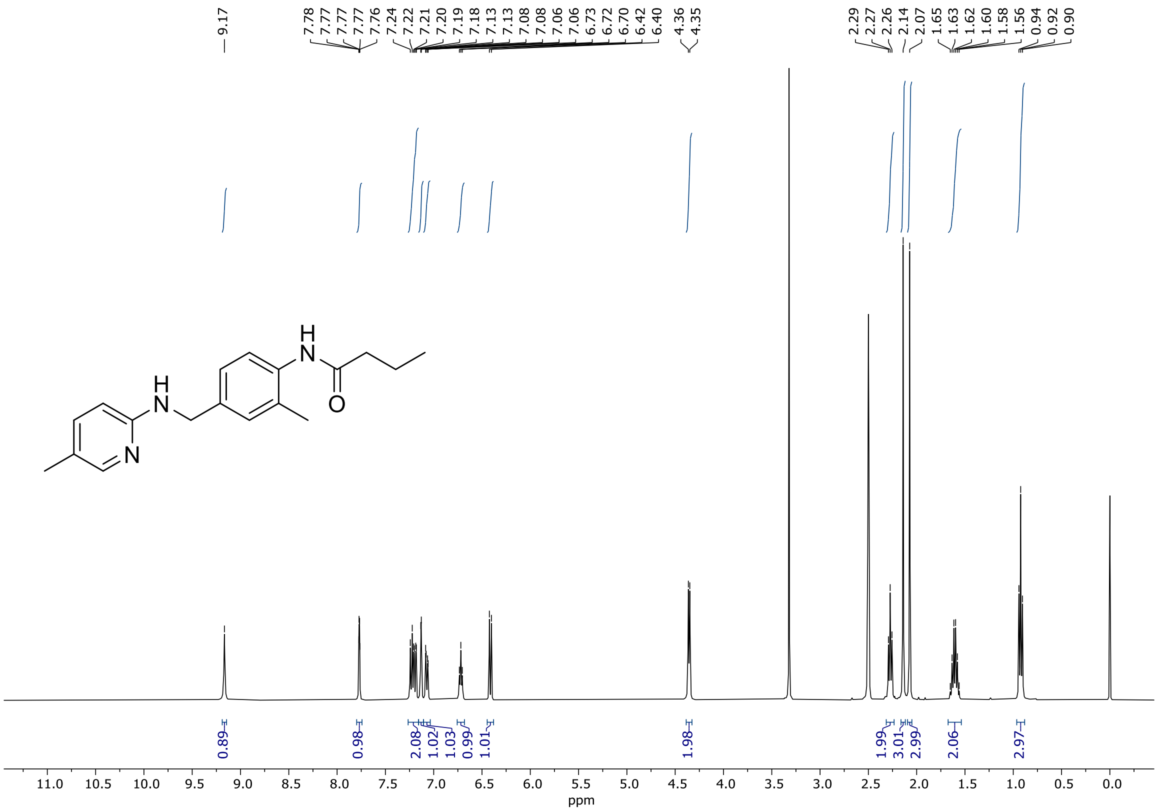

Figure S71: ^{1}H-NMR spectrum of compound **35f**.

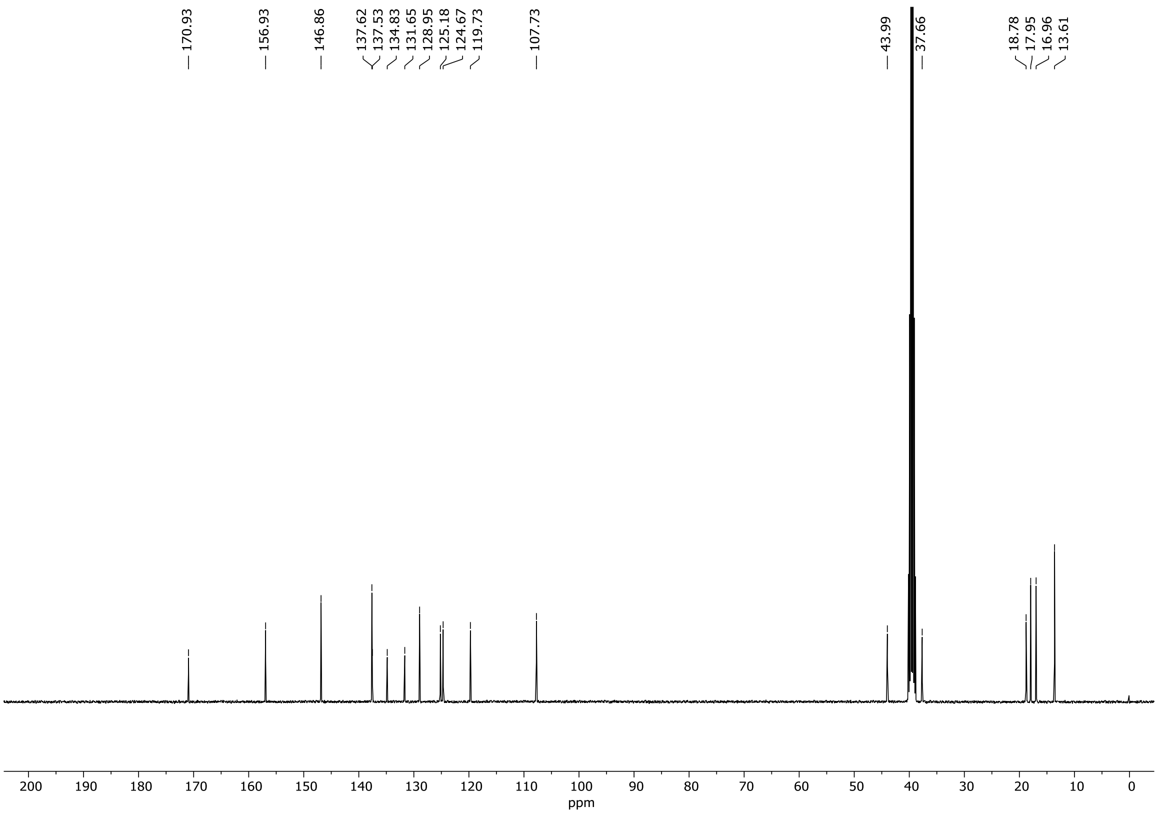

Figure S72: ^{13}C-NMR spectrum of compound **35f**.

Ethyl 6-aminonicotinate (**37**)

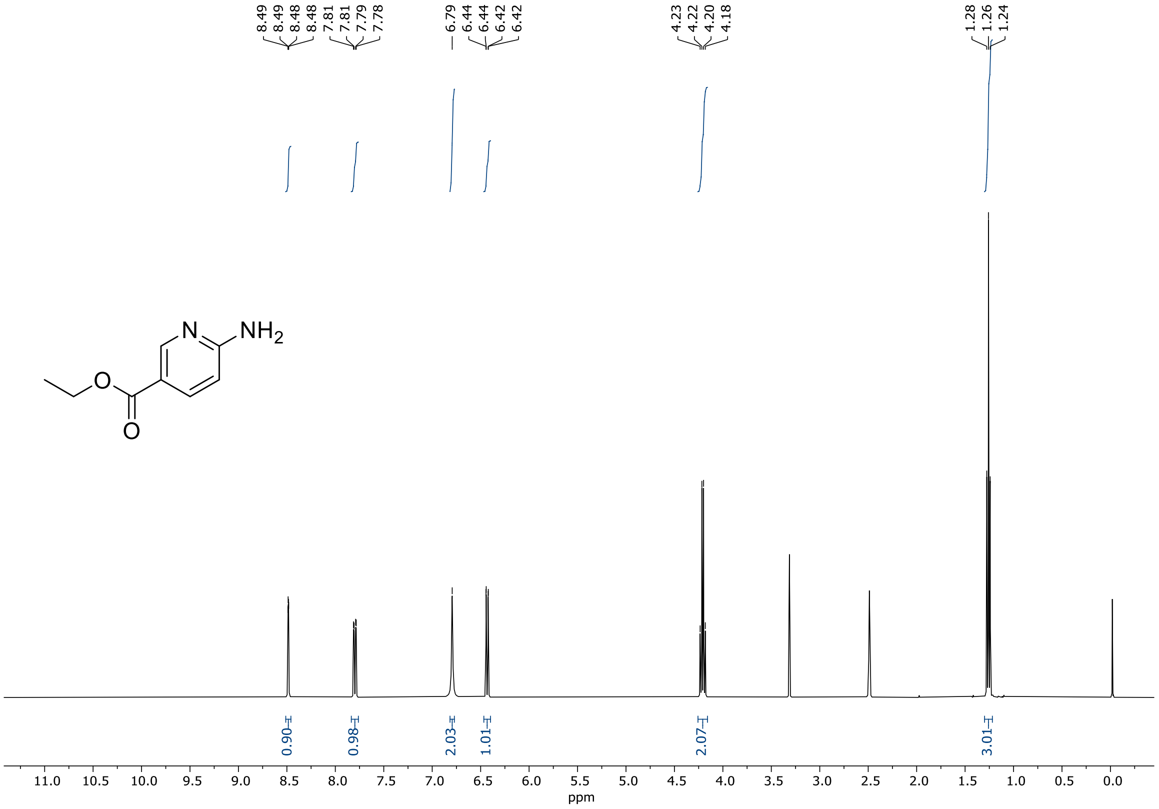

Figure S73: ^{1}H-NMR spectrum of compound **37**.

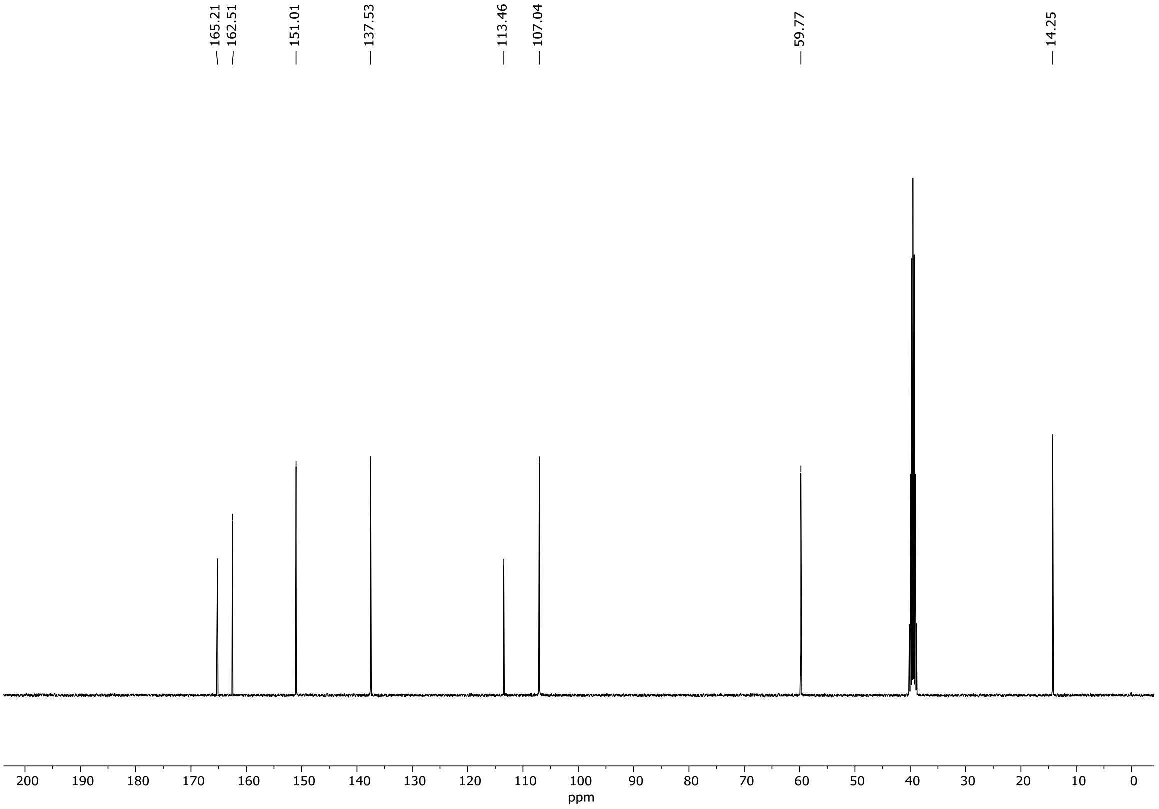

Figure S74: ^{13}C-NMR spectrum of compound **37**.

Ethyl 6-amino-5-bromonicotinate (**38**)

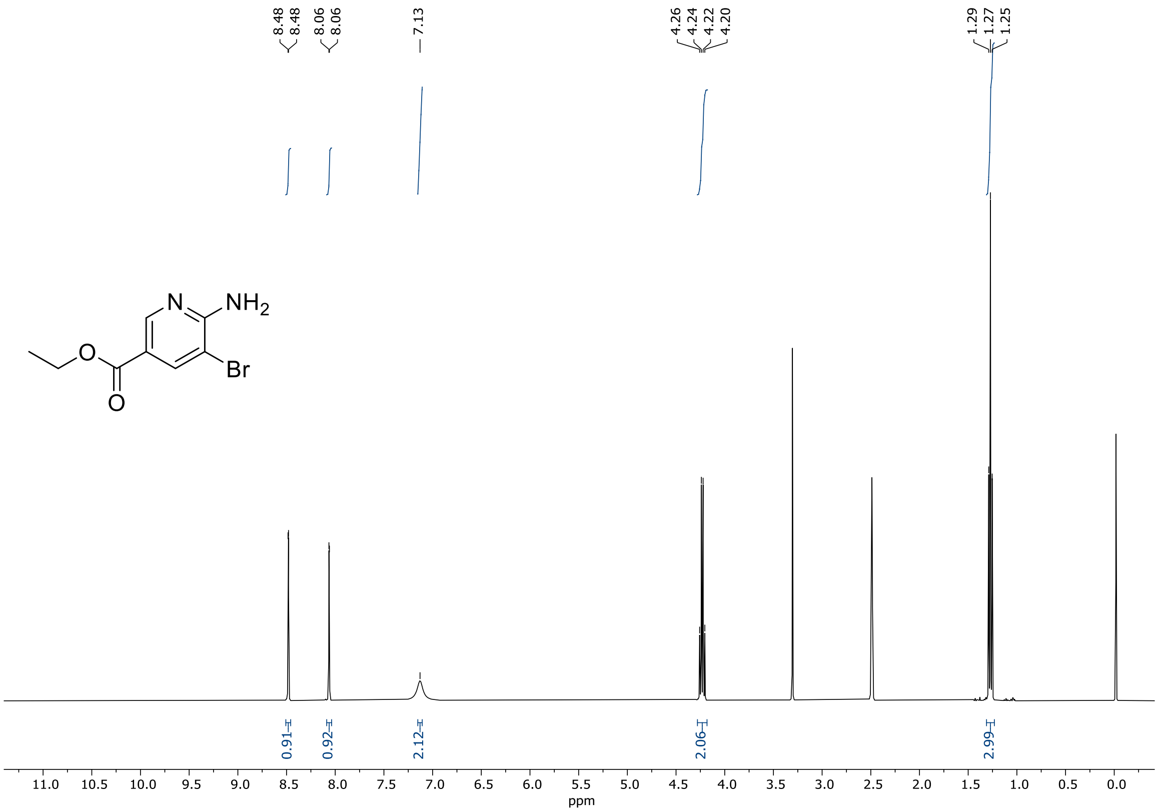

Figure S75: ^{1}H-NMR spectrum of compound **38**.

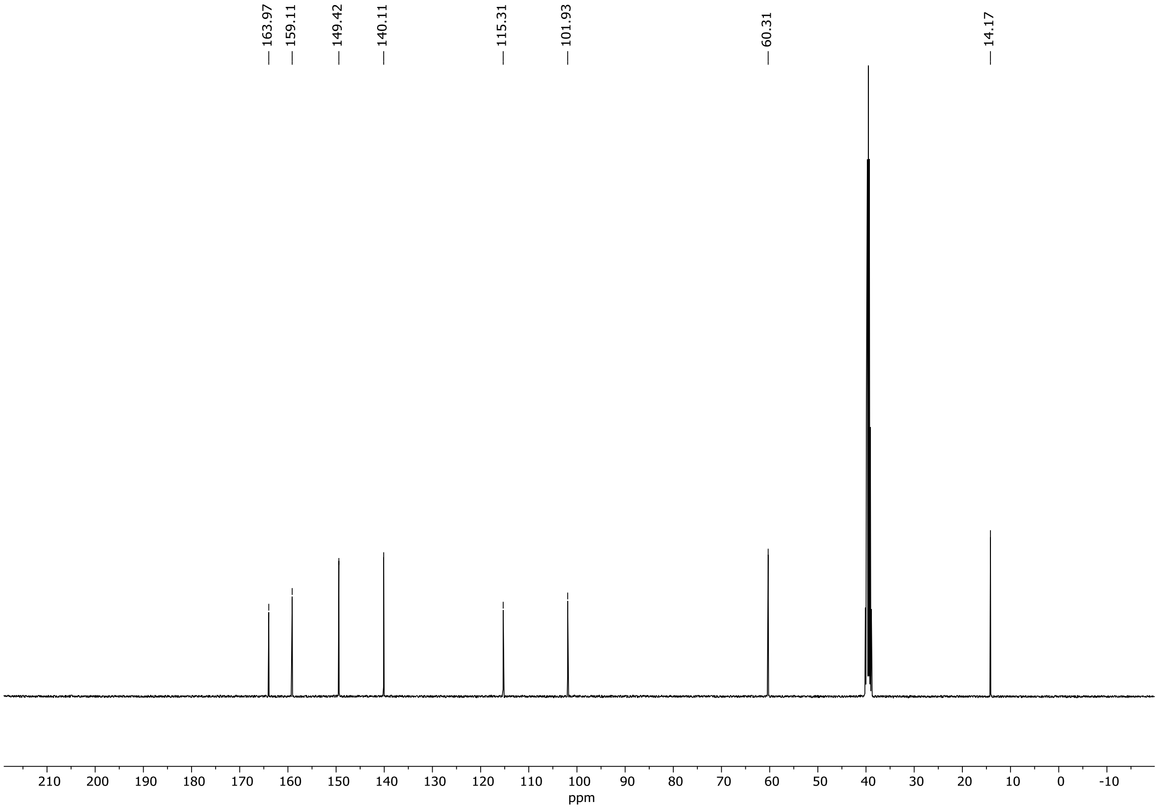

Figure S76: ^{13}C-NMR spectrum of compound **38**.

(6-Amino-5-bromopyridin-3-yl)methanol (**39**)

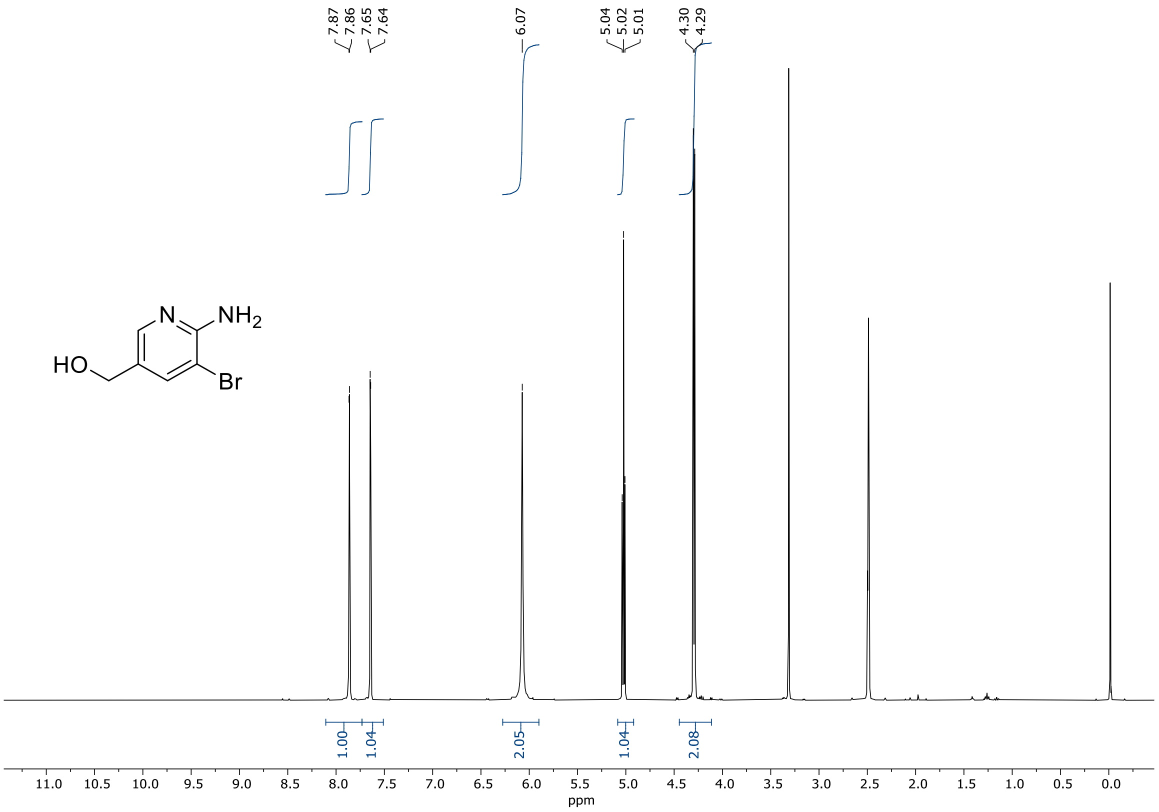

Figure S77: ^{1}H-NMR spectrum of compound **39**.

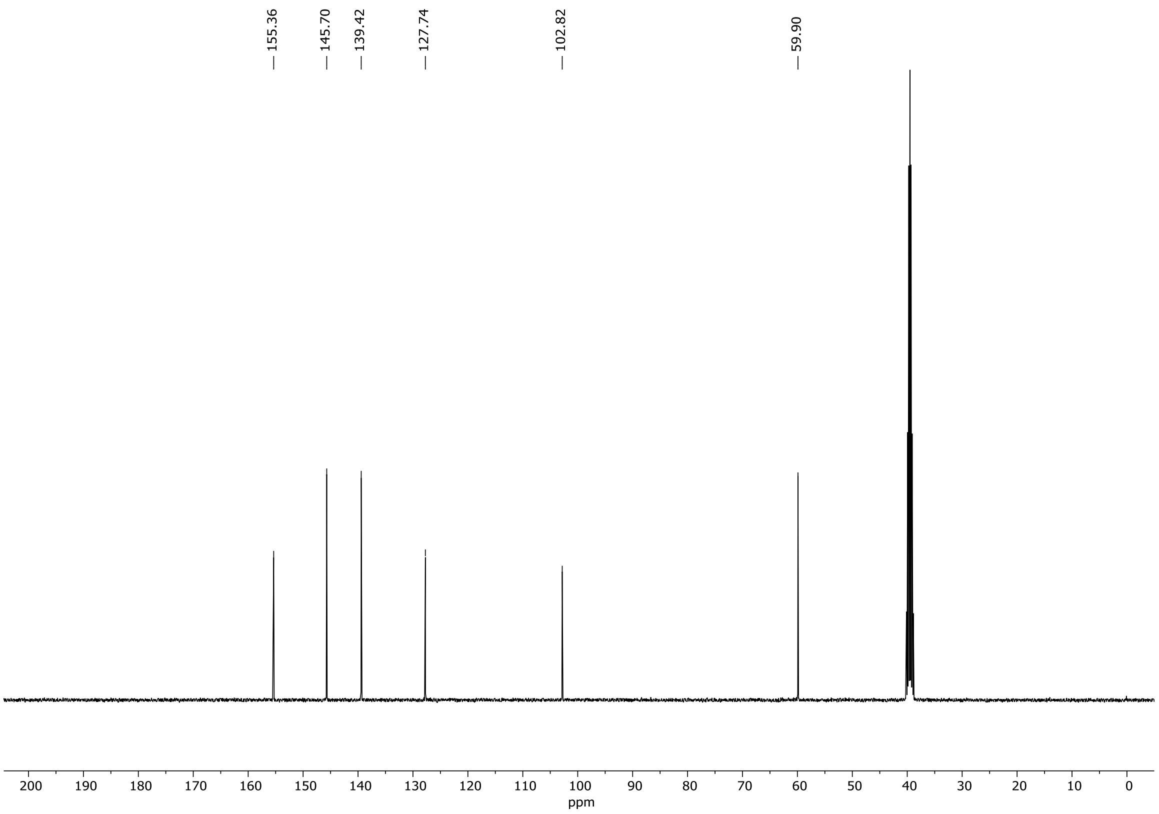

Figure S78: ^{13}C-NMR spectrum of compound **39**.

6-Amino-5-bromonicotinaldehyde (**40**)

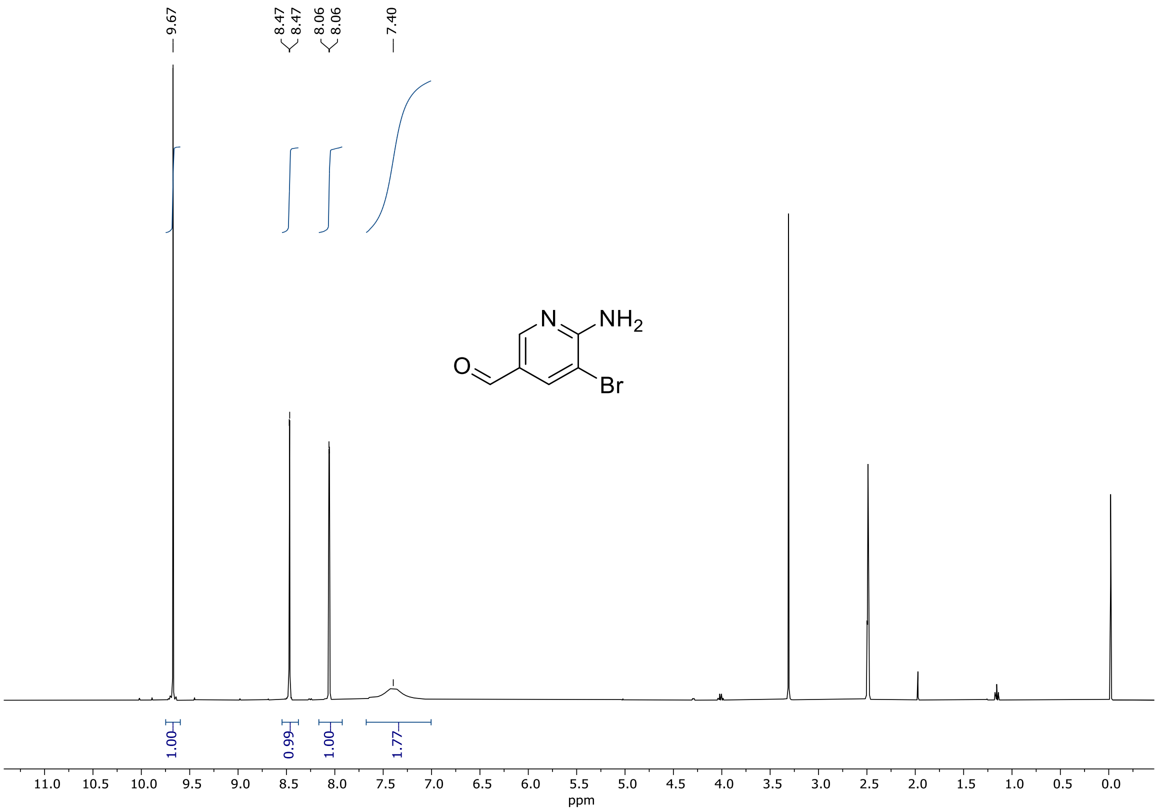

Figure S79: ^{1}H-NMR spectrum of compound **40**.

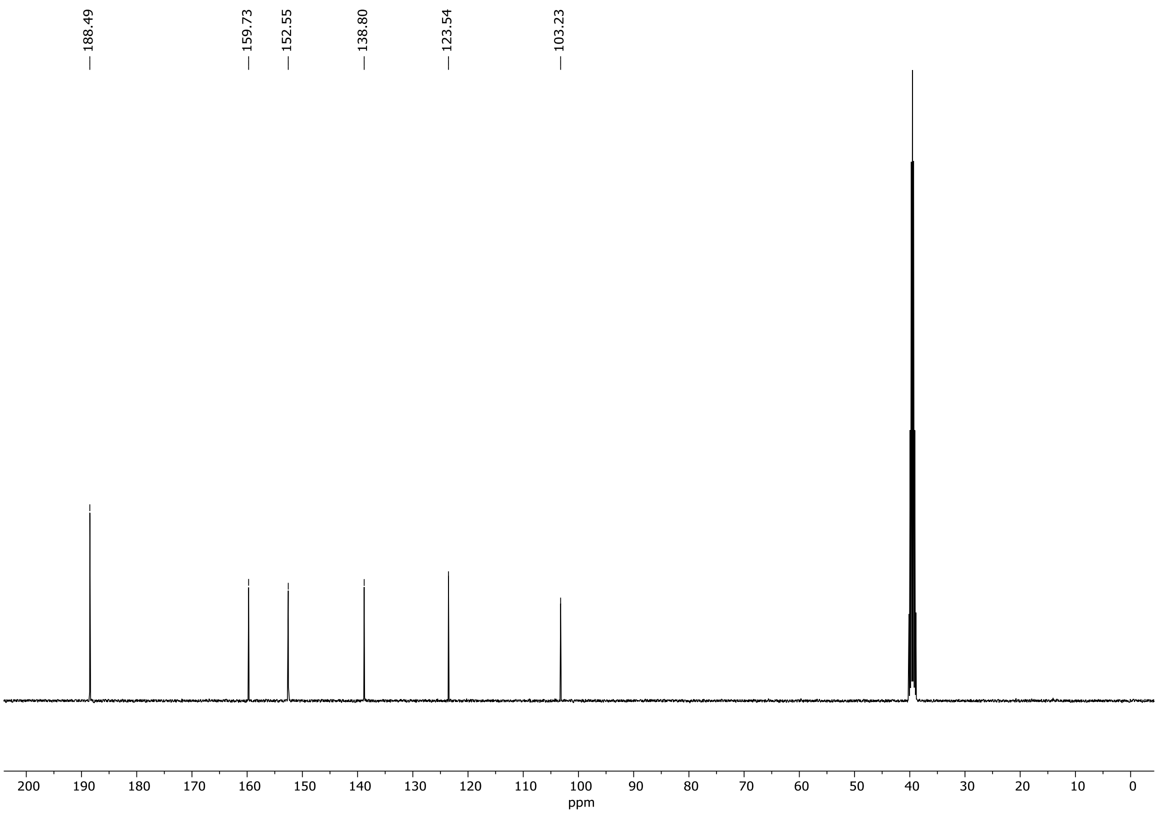

Figure S80: ^{13}C-NMR spectrum of compound **40**.

N-(3-Bromo-5-formylpyridin-2-yl)butyramide (**41a**)

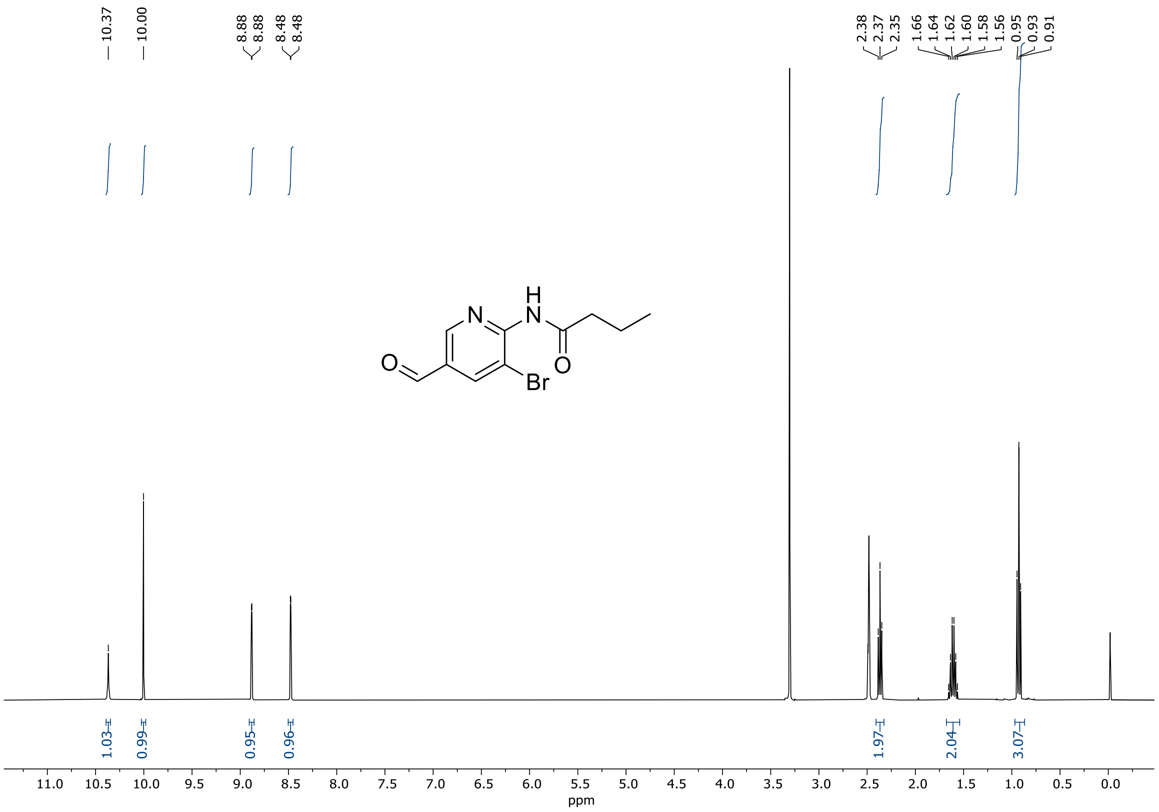

Figure S81: ^{1}H-NMR spectrum of compound **41a**.

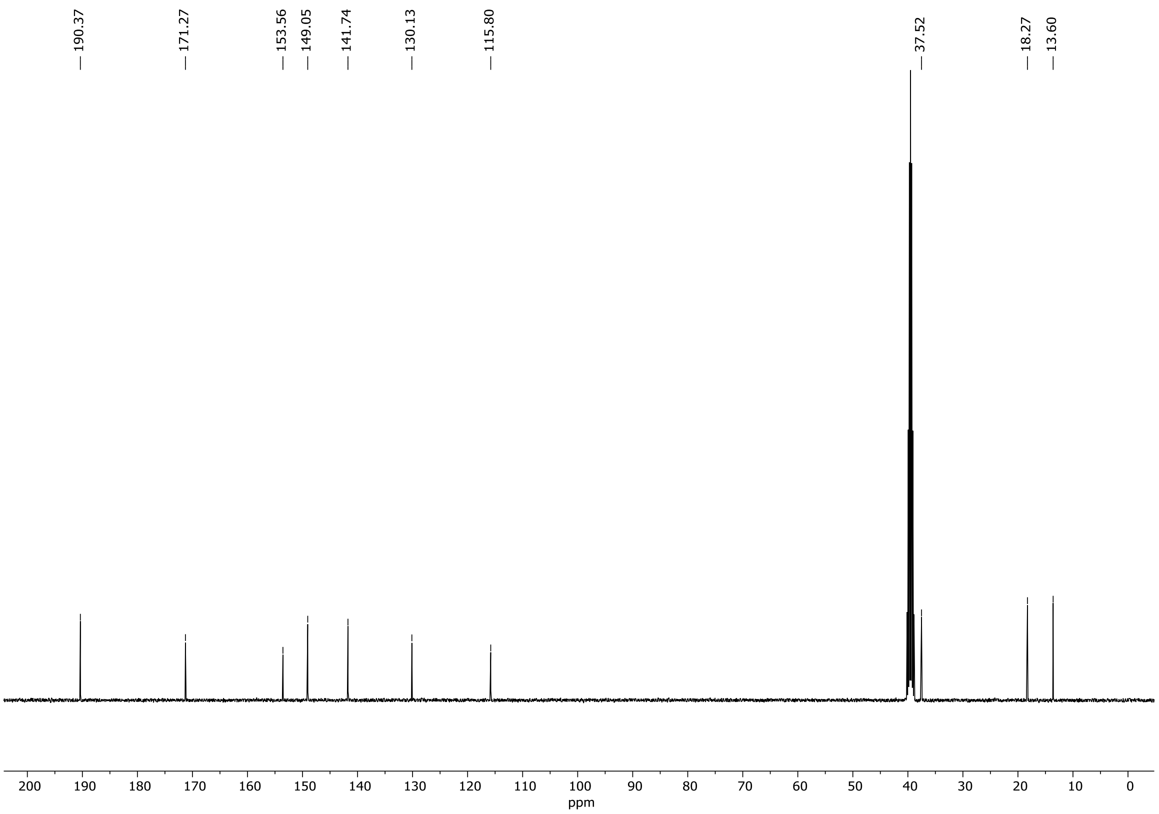

Figure S82: ^{13}C-NMR spectrum of compound **41a**.

N-(3-Bromo-5-formylpyridin-2-yl)-3,3-dimethylbutanamide (**41b**)

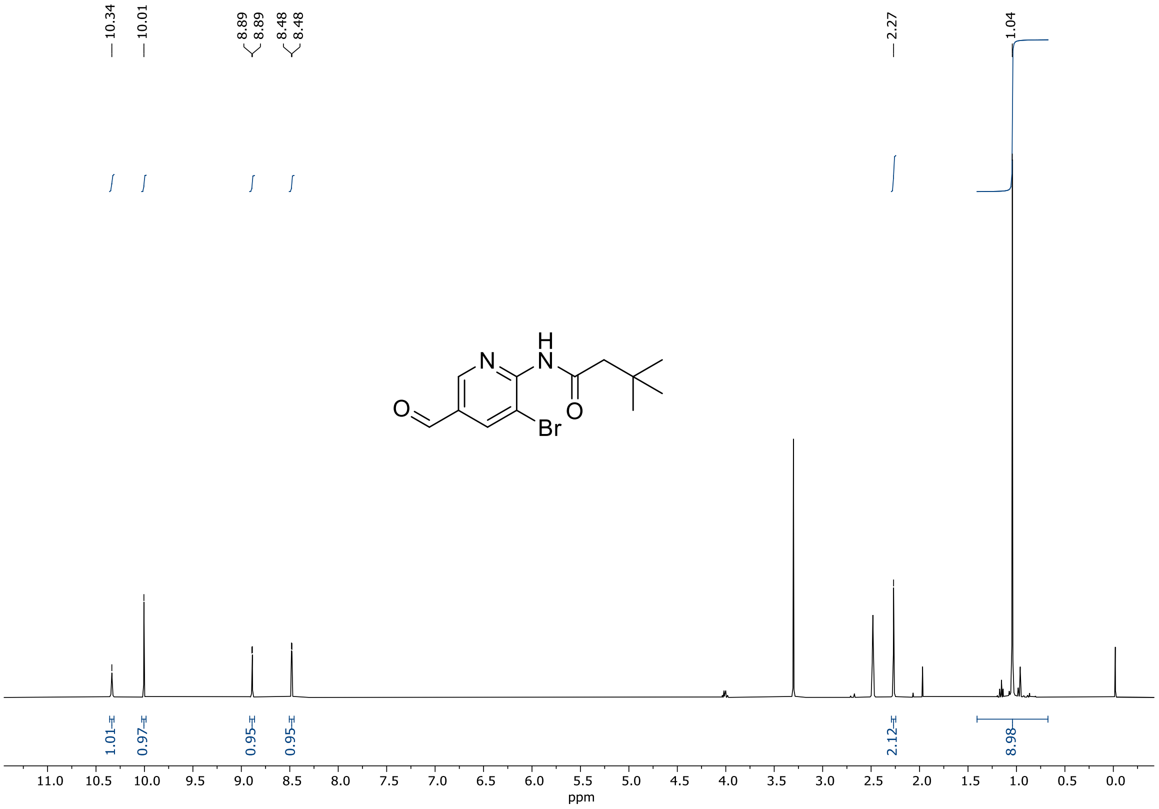

Figure S83: ^{1}H-NMR spectrum of compound **41b**.

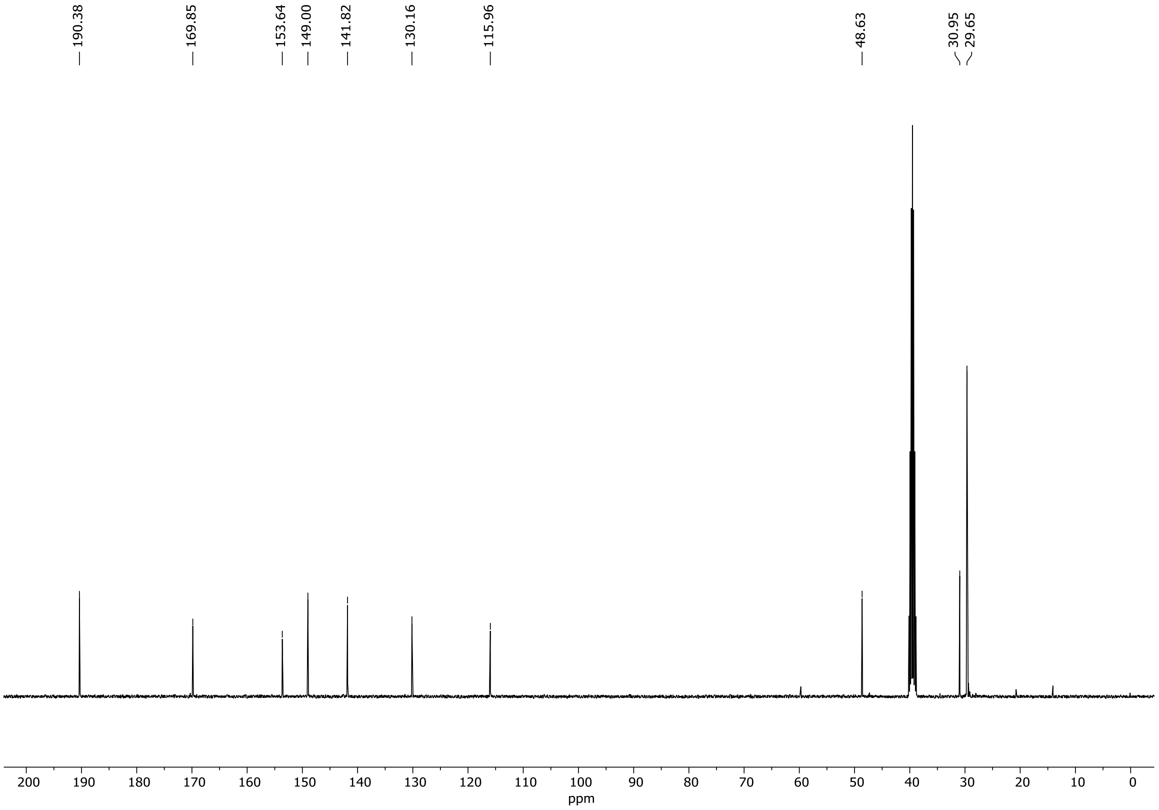

Figure S84: ^{13}C-NMR spectrum of compound **41b**.

N-(3-Bromo-5-{[(4-fluorophenyl)amino]methyl}pyridin-2-yl)butyramide (**42a**)

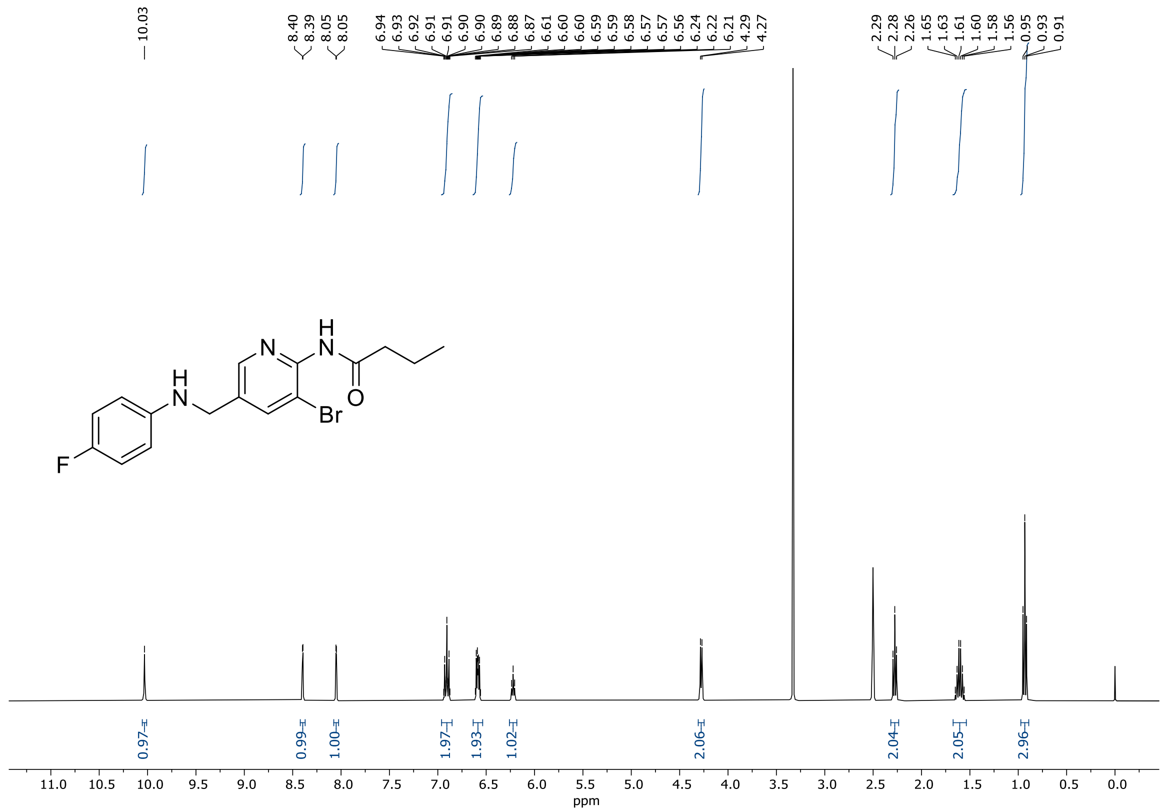

Figure S85: ^{1}H-NMR spectrum of compound **42a**.

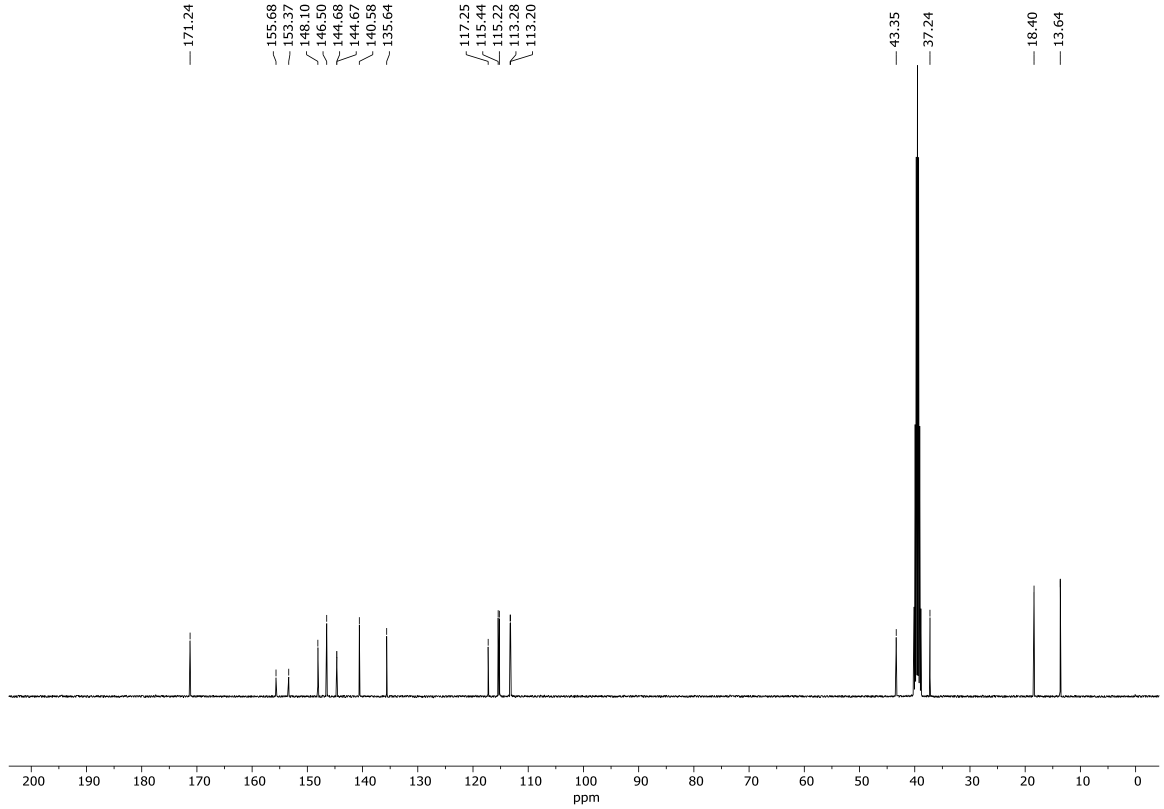

Figure S86: ^{13}C-NMR spectrum of compound **42a**.

N-(3-Bromo-5-{[(4-fluorophenyl)amino]methyl}pyridin-2-yl)-3,3-dimethylbutanamide (**42b**)

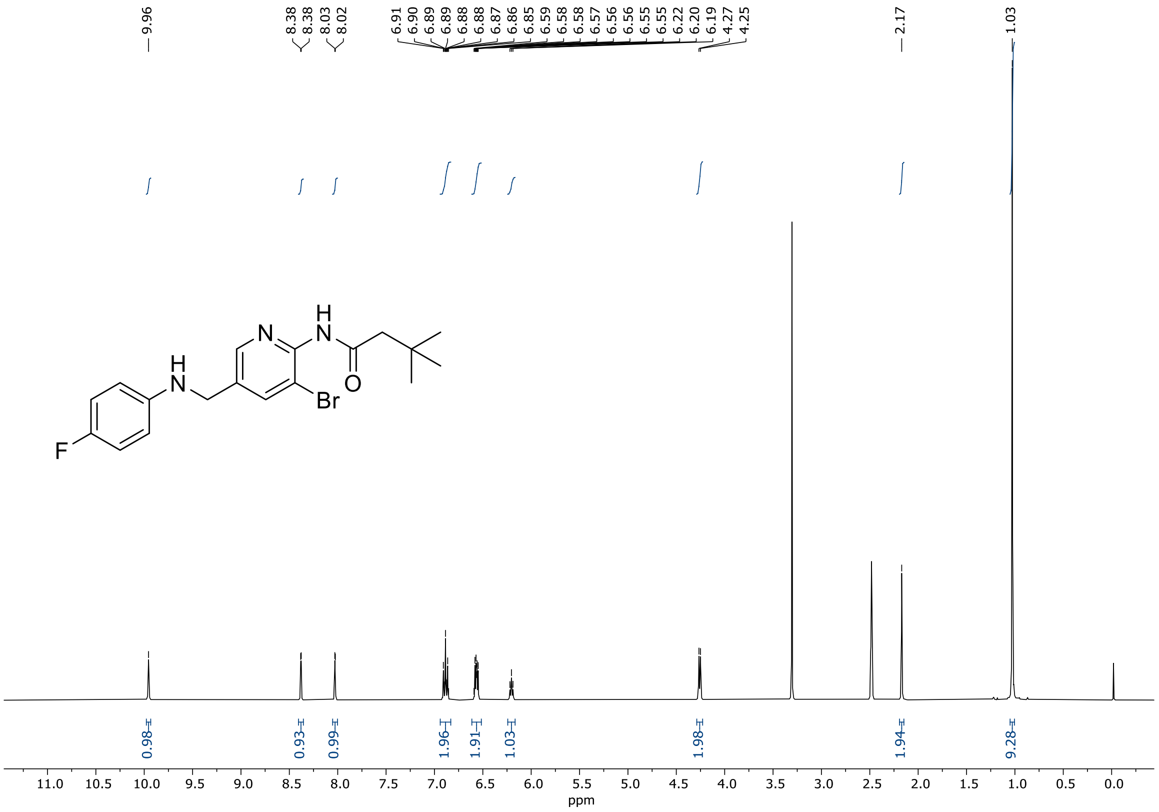

Figure S87: ^{1}H-NMR spectrum of compound **42b**.

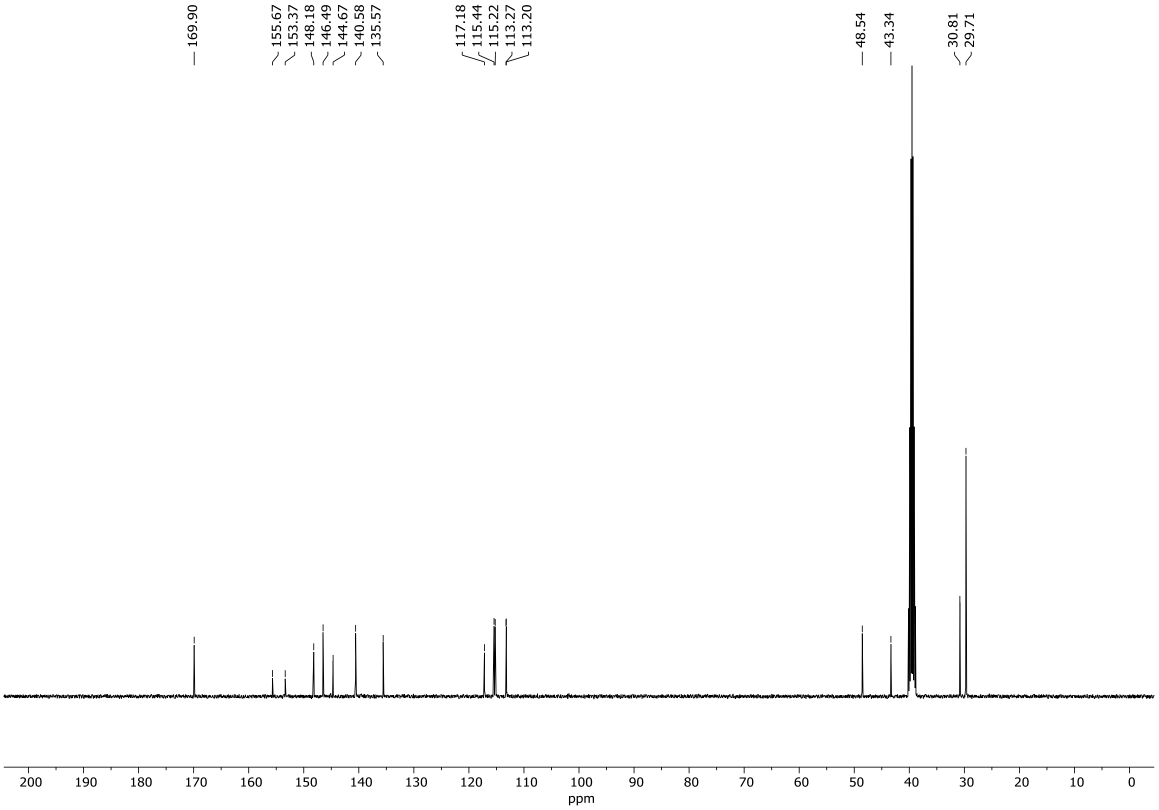

Figure S88: ^{13}C-NMR spectrum of compound **42b**.

N-(5-{[(4-Fluorophenyl)amino]methyl}-3-methylpyridin-2-yl)butyramide (**43a**)

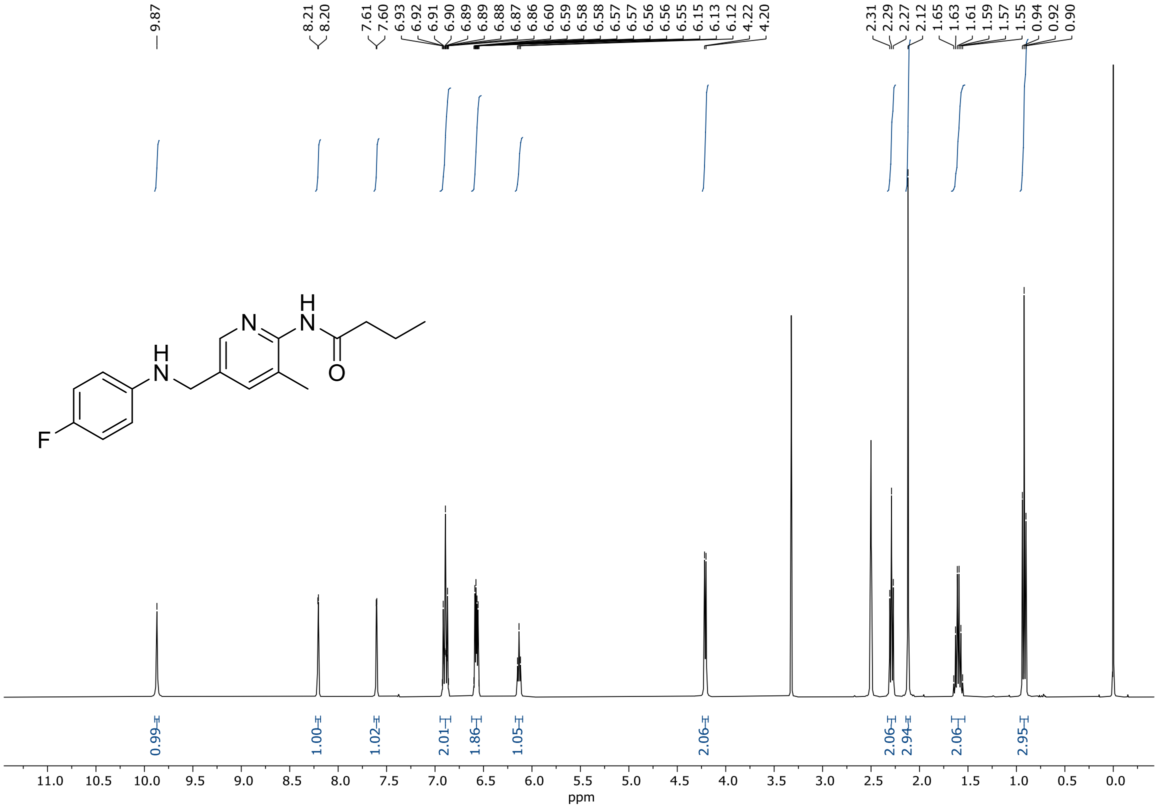

Figure S89: ^{1}H-NMR spectrum of compound **43a**.

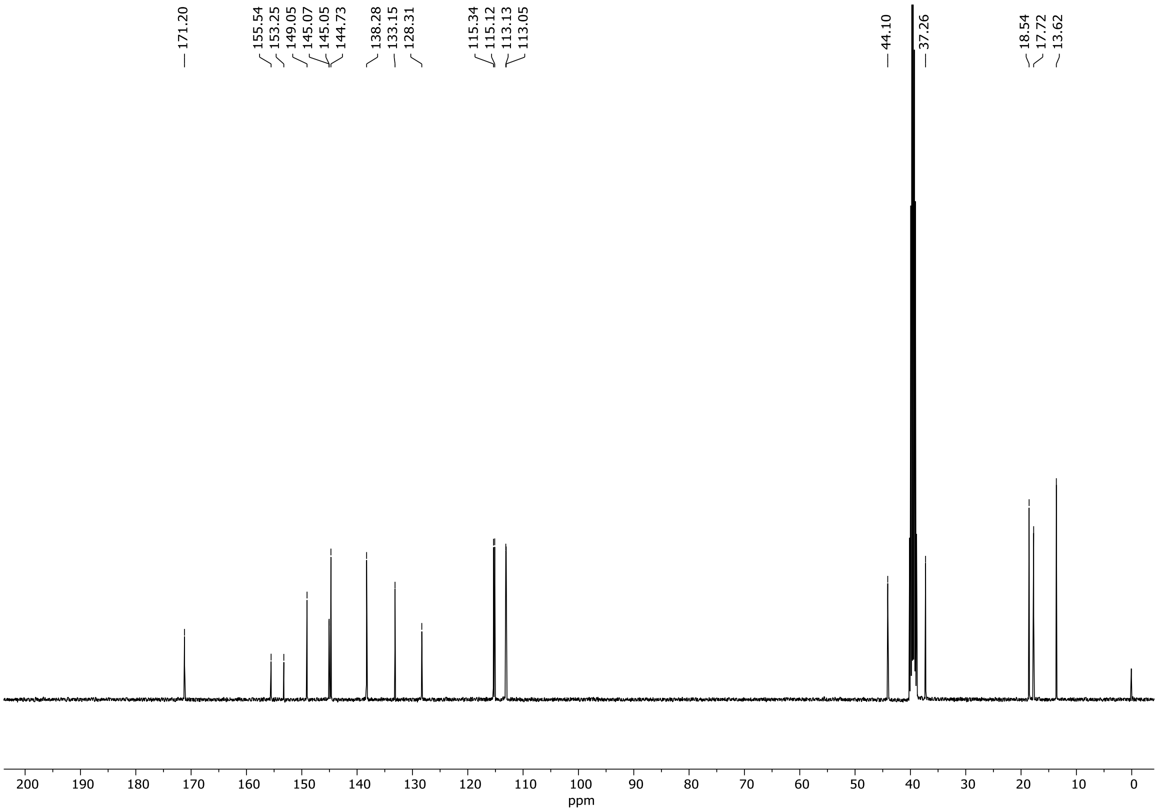

Figure S90: ^{13}C-NMR spectrum of compound **43a**.

N-(5-{[(4-Fluorophenyl)amino]methyl}-3-methylpyridin-2-yl)-3,3-dimethylbutanamide (**43b**)

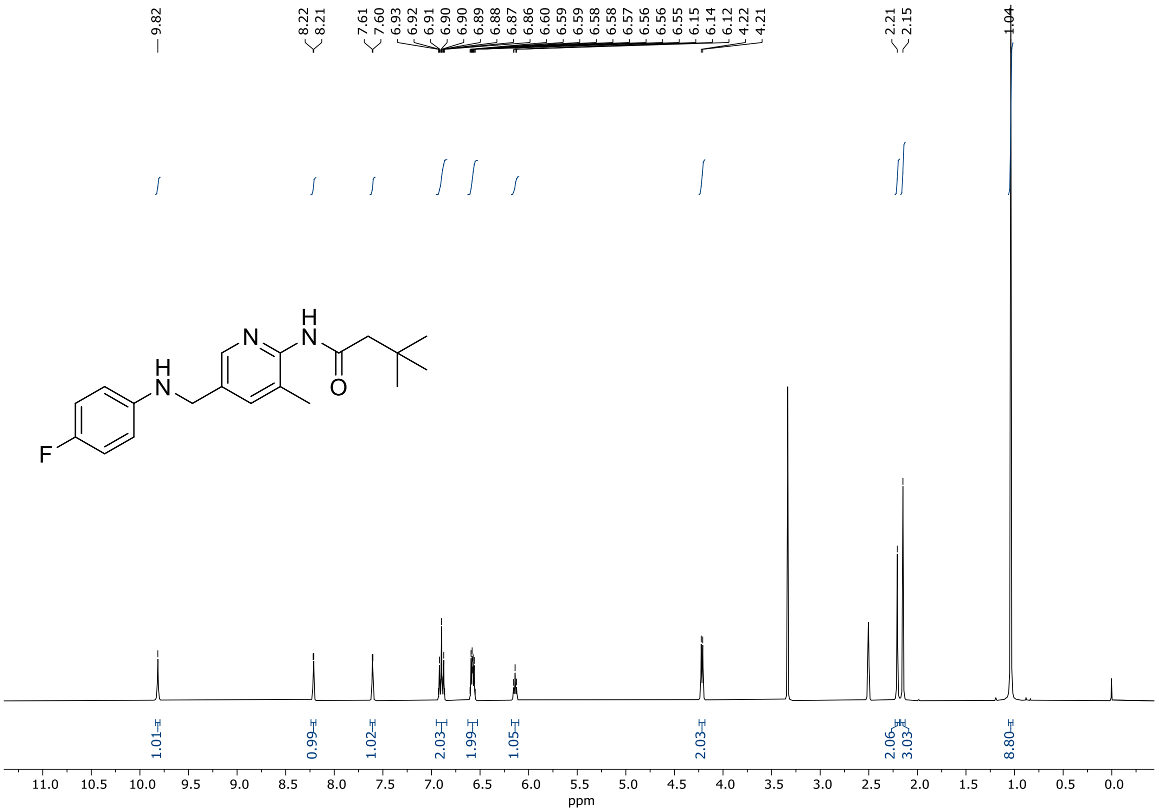

Figure S91: ^{1}H-NMR spectrum of compound **43b**.

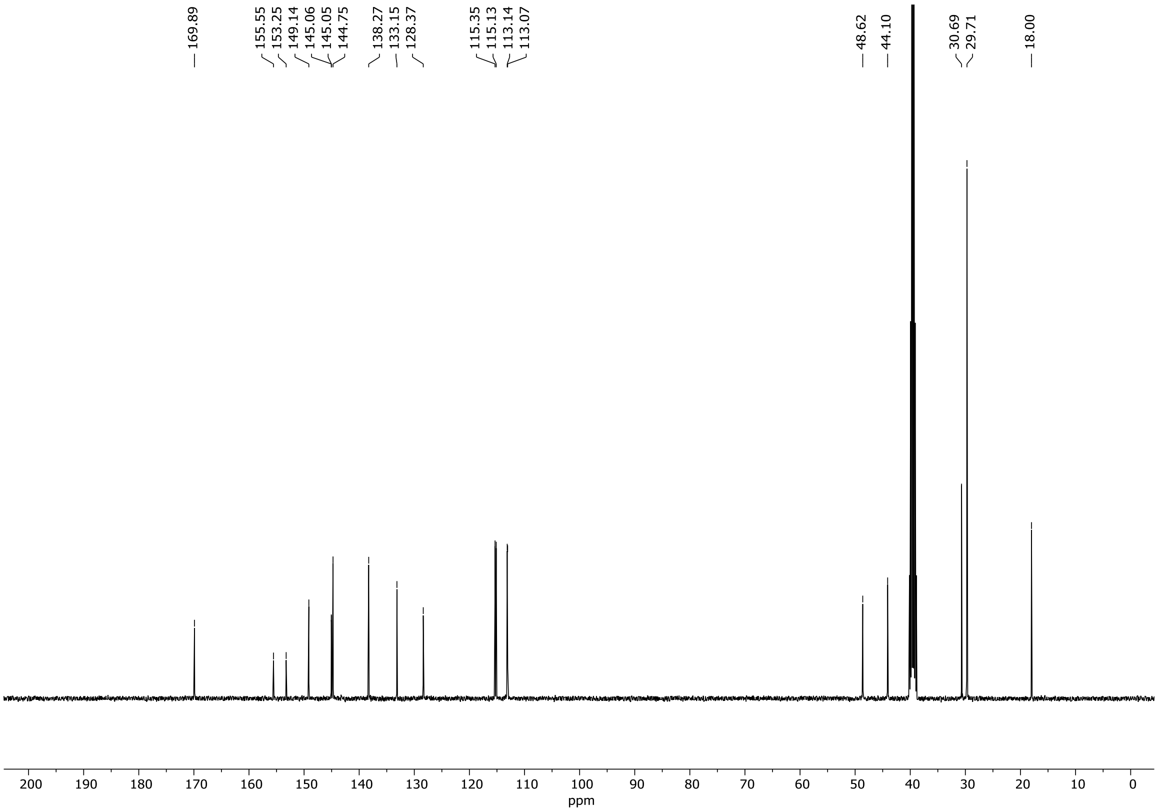

Figure S92: ^{13}C-NMR spectrum of compound **43b**.

N-(3-Cyclopropyl-5-{[(4-fluorophenyl)amino]methyl}pyridin-2-yl)butyramide (**43c**)

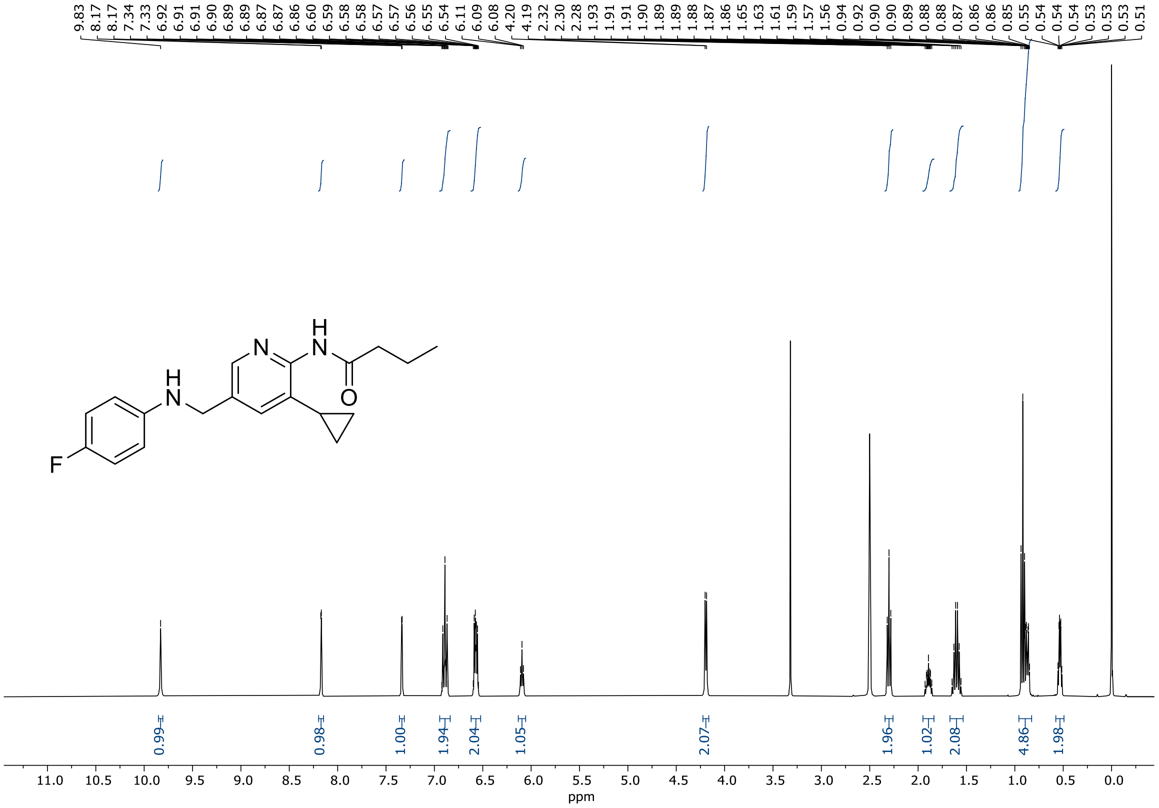

Figure S93: ^{1}H-NMR spectrum of compound **43c**.

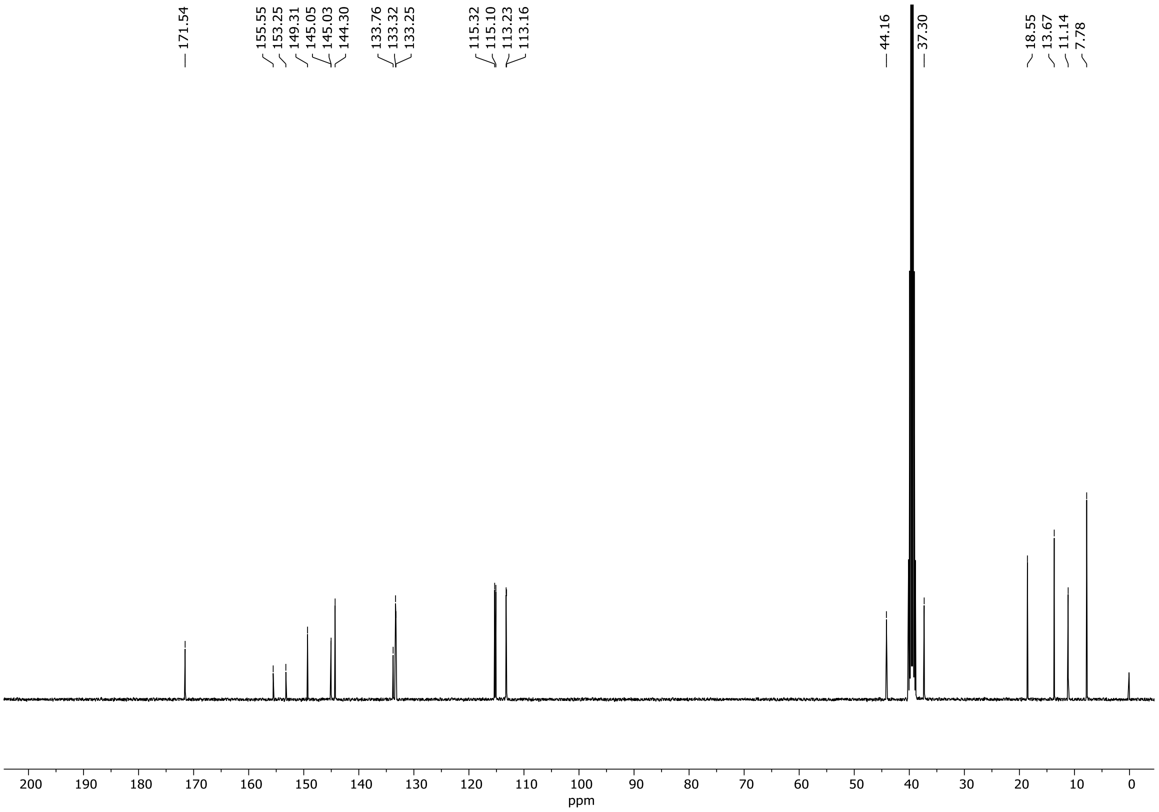

Figure S94: ^{13}C-NMR spectrum of compound **43c**.

N-(5-{[(4-Fluorophenyl)amino]methyl}pyridin-2-yl)butyramide (**44**)

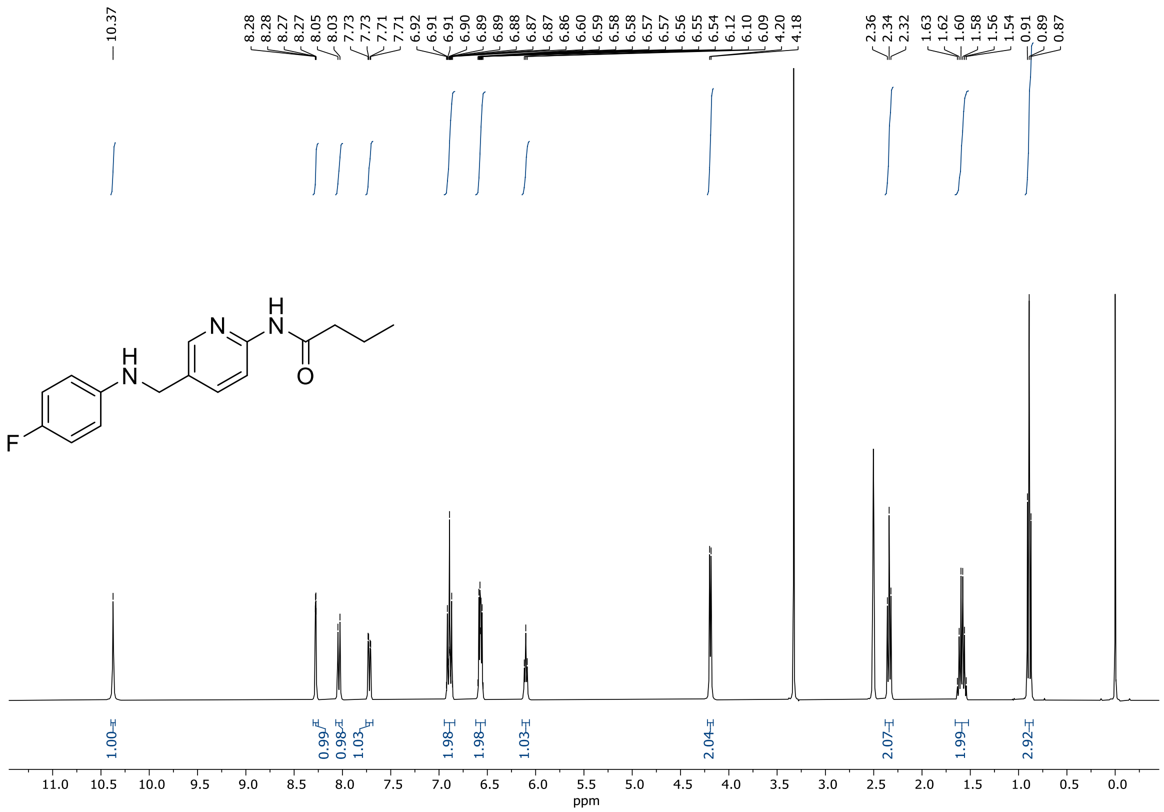

Figure S95: ^{1}H-NMR spectrum of compound **44**.

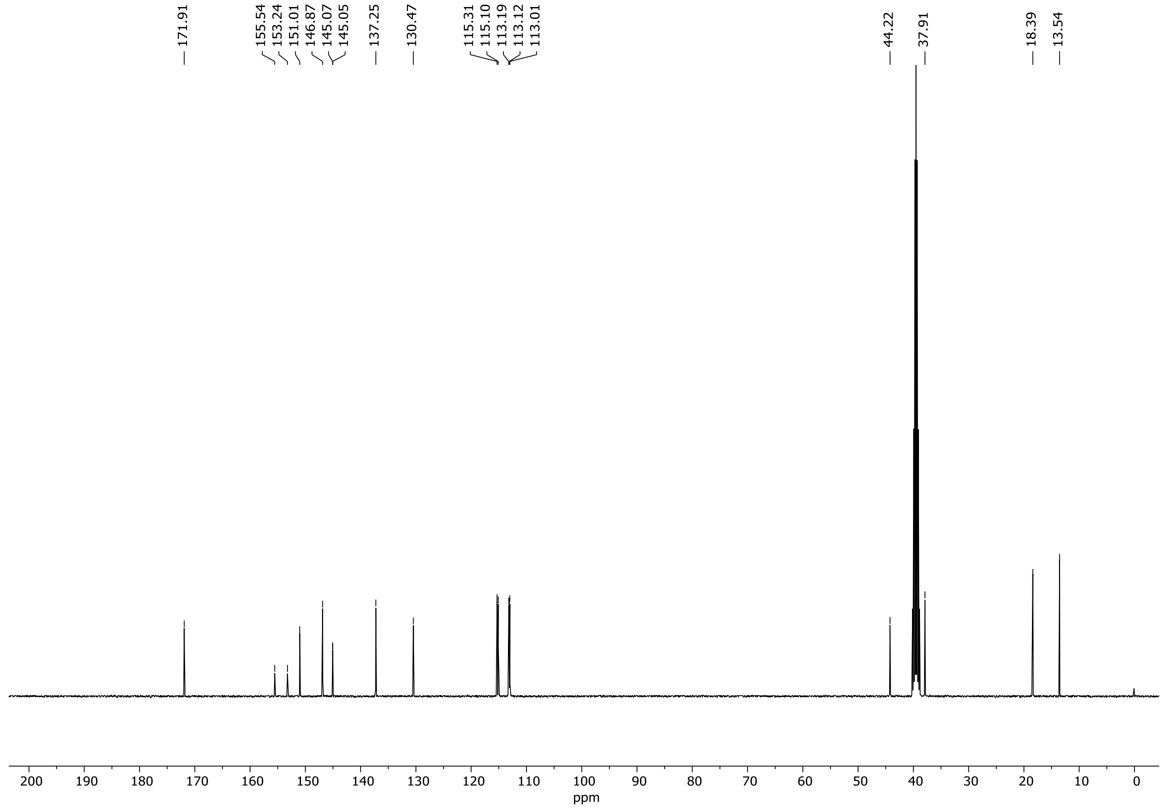

Figure S96: ^{13}C-NMR spectrum of compound **44**.

4-(1,3-Dioxoisoindolin-2-yl)-3-methylbenzonitrile (**45**)

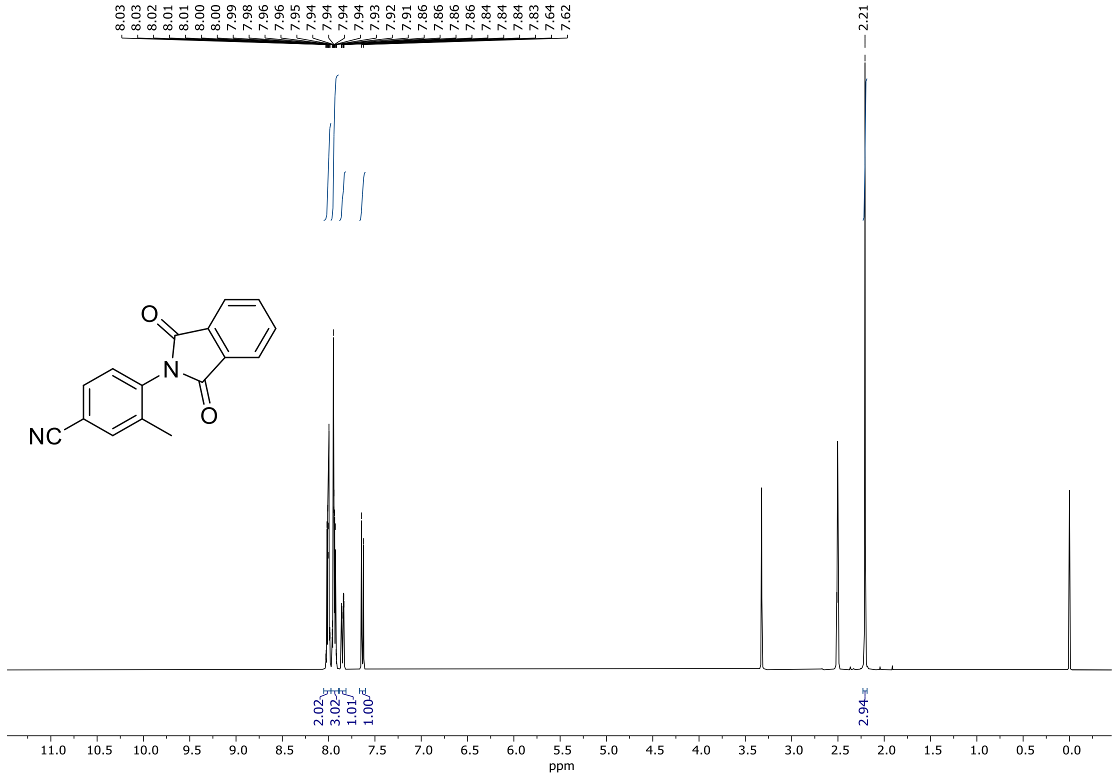

Figure S97: ^{1}H-NMR spectrum of compound **45**.

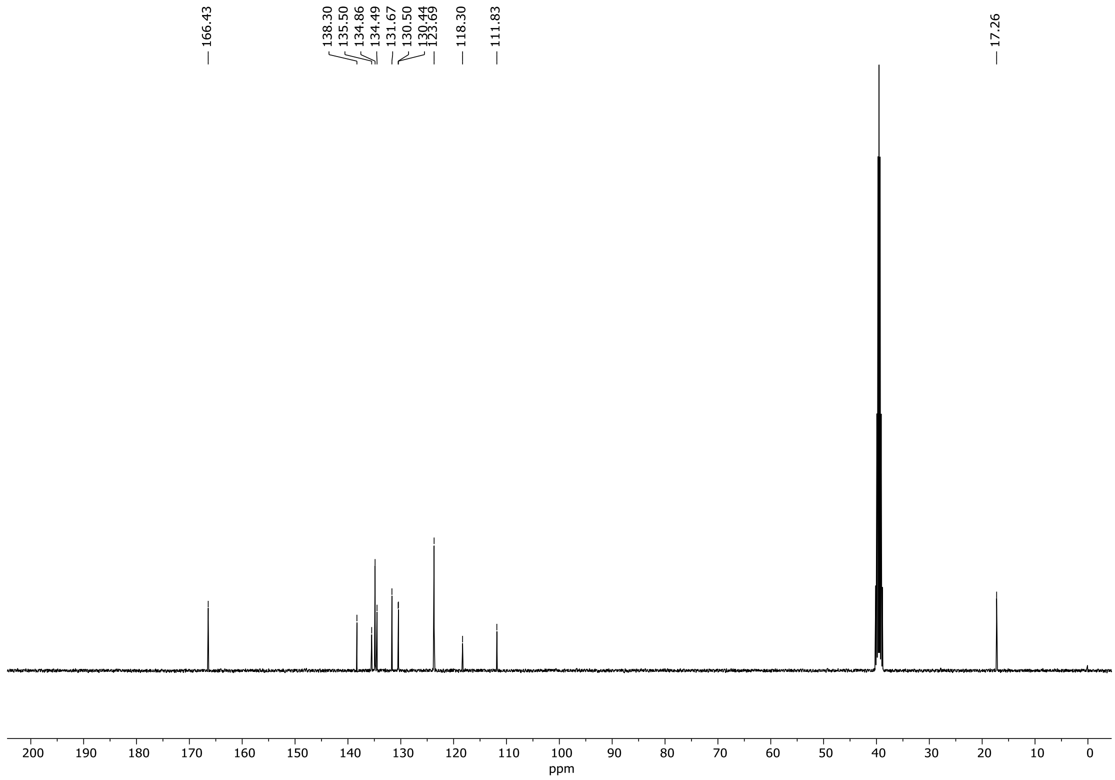

Figure S98: ^{13}C-NMR spectrum of compound **45**.

4-(1,3-Dioxoisoindolin-2-yl)-3-methylbenzaldehyde (**46**)

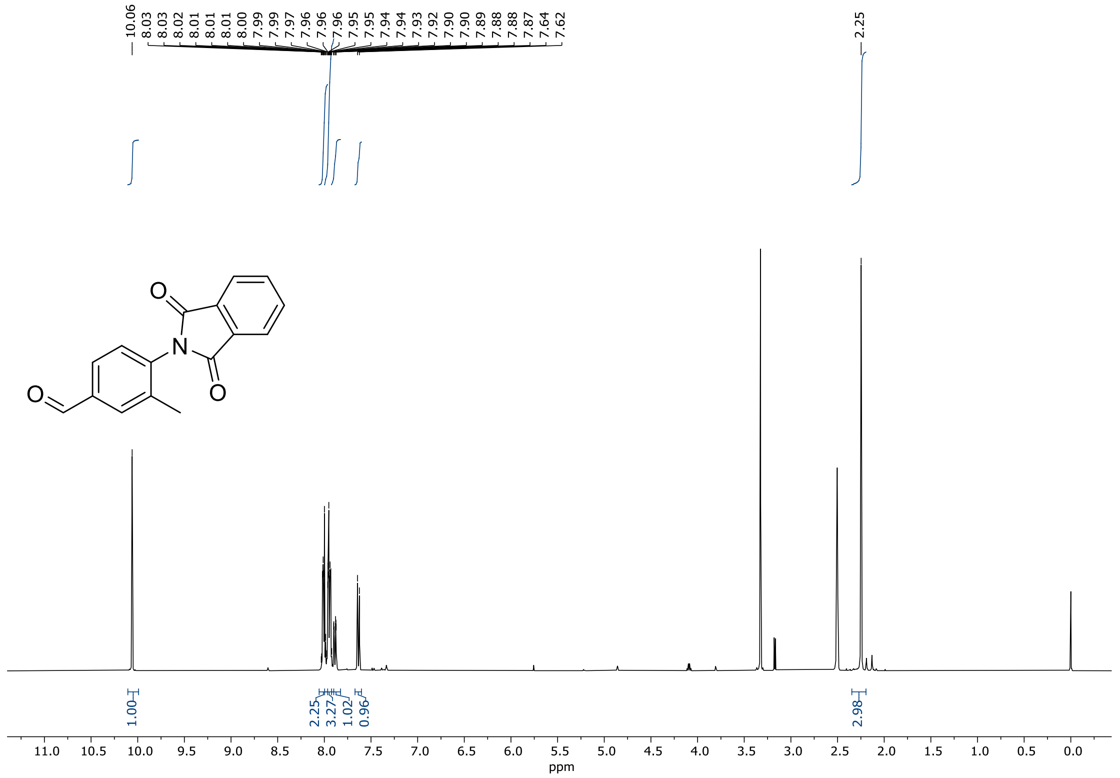

Figure S99: ^{1}H-NMR spectrum of compound **46**.

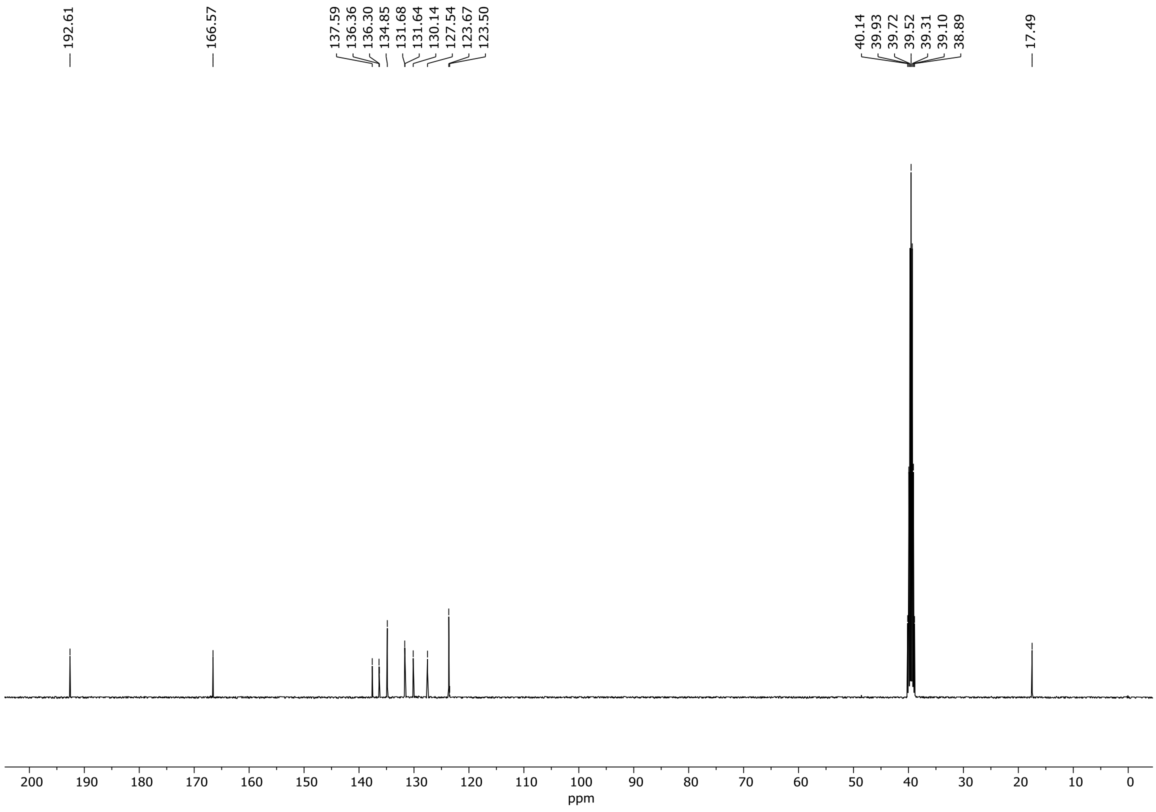

Figure S100: ^{13}C-NMR spectrum of compound **46**.

N-(4-{[(4-Fluorophenyl)amino]methyl}-2-methylphenyl)-2-(hydroxymethyl)benzamide (**48**)

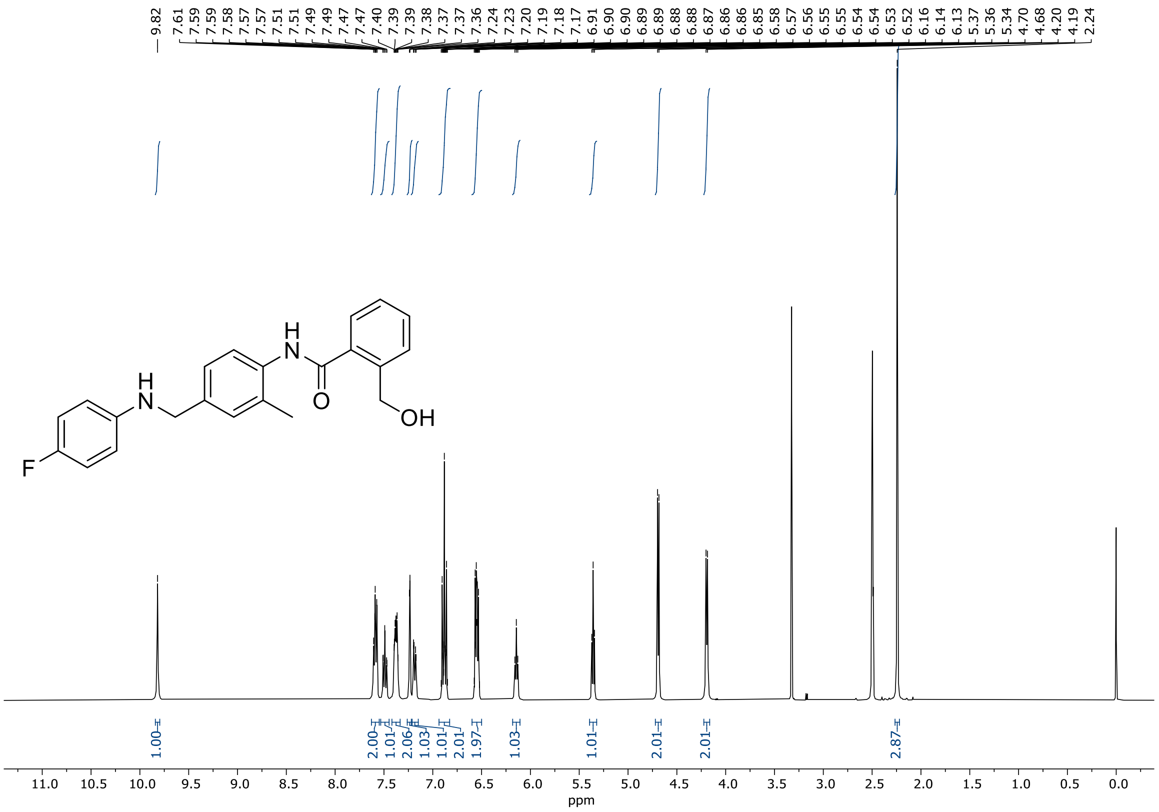

Figure S101: ^{1}H-NMR spectrum of compound **48**.

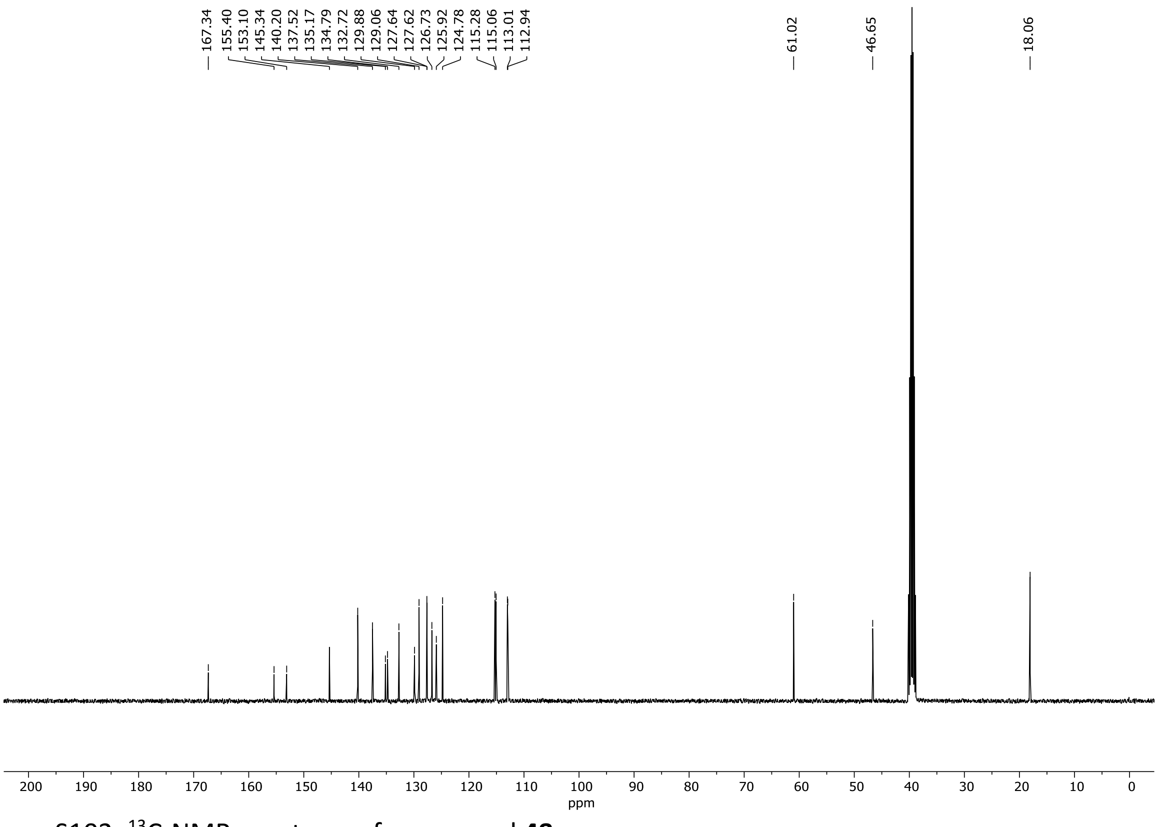

Figure S102: ^{13}C-NMR spectrum of compound **48**.

2-(4-{[(4-Fluorophenyl)amino]methyl}-2-methylphenyl)isoindoline-1,3-dione (**49**)

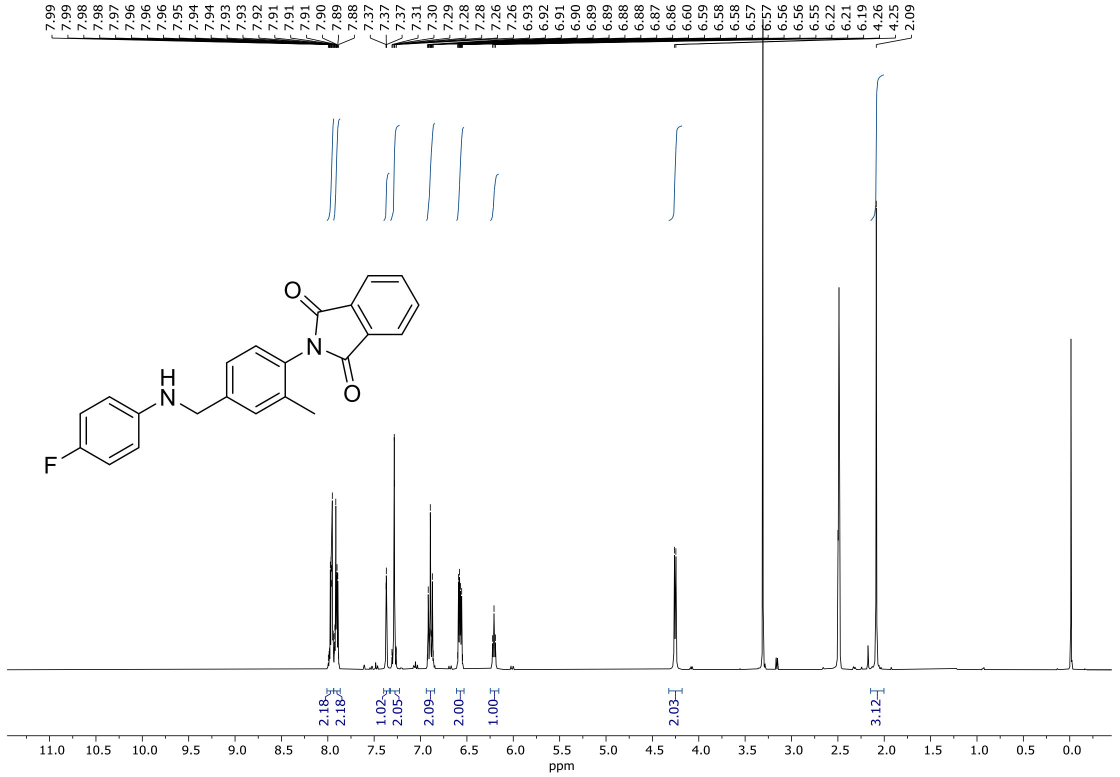

Figure S103: ^{1}H-NMR spectrum of compound **49**.

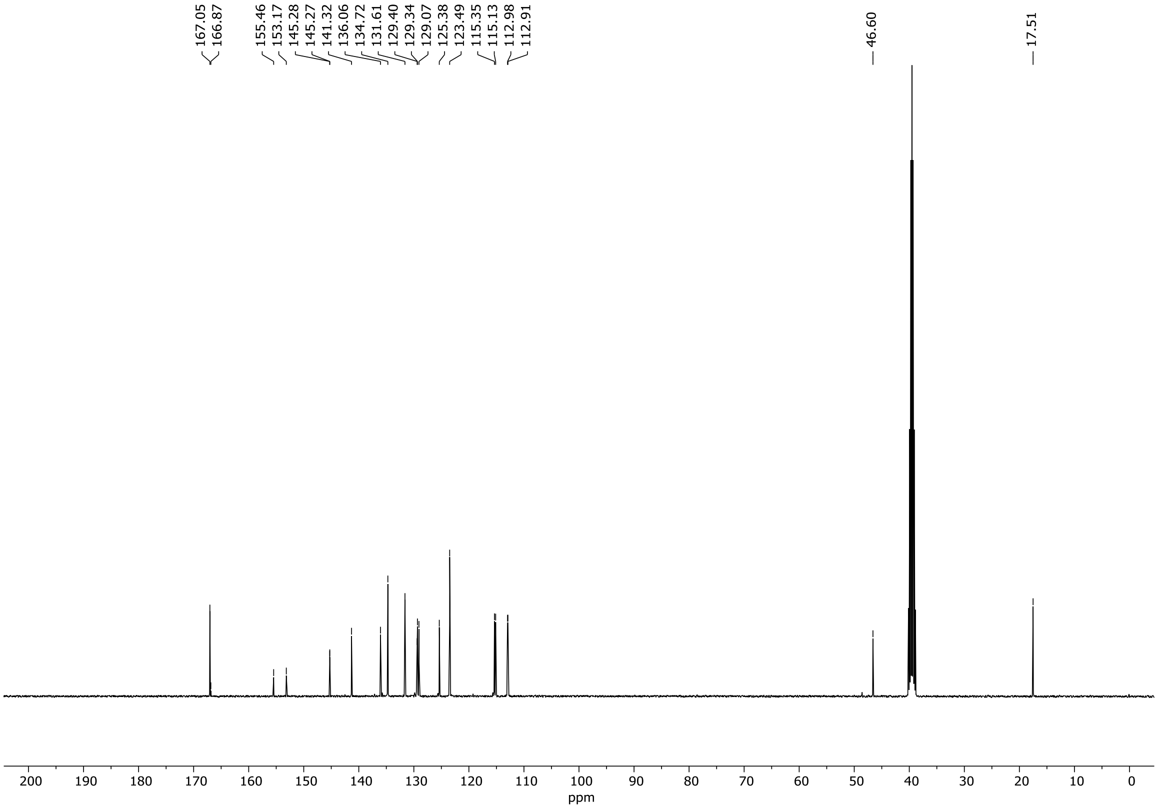

Figure S104: ^{13}C-NMR spectrum of compound **49**.

Benzyl [4-(1,3-dioxoisoindolin-2-yl)-3-methylbenzyl](4-fluorophenyl)carbamate (**50**)

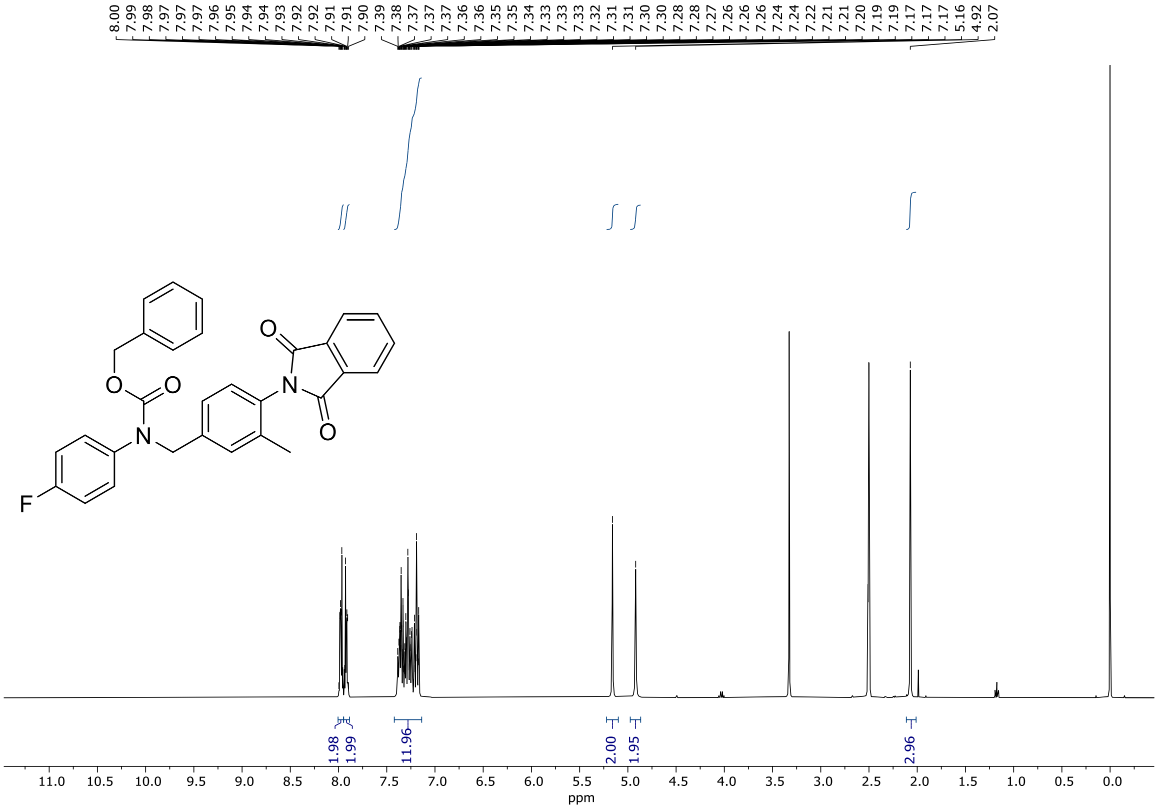

Figure S105: ^{1}H-NMR spectrum of compound **50**.

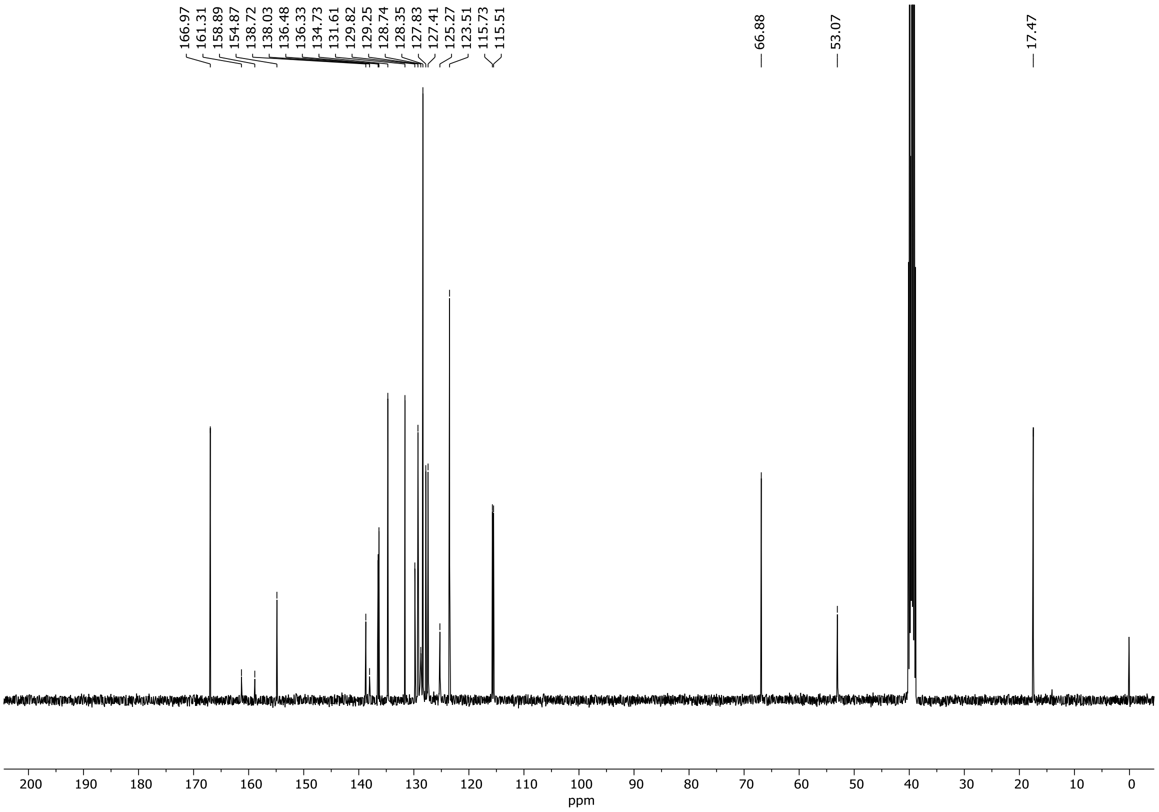

Figure S106: ^{13}C-NMR spectrum of compound **50**.

Benzyl (4-amino-3-methylbenzyl)(4-fluorophenyl)carbamate (**51**)

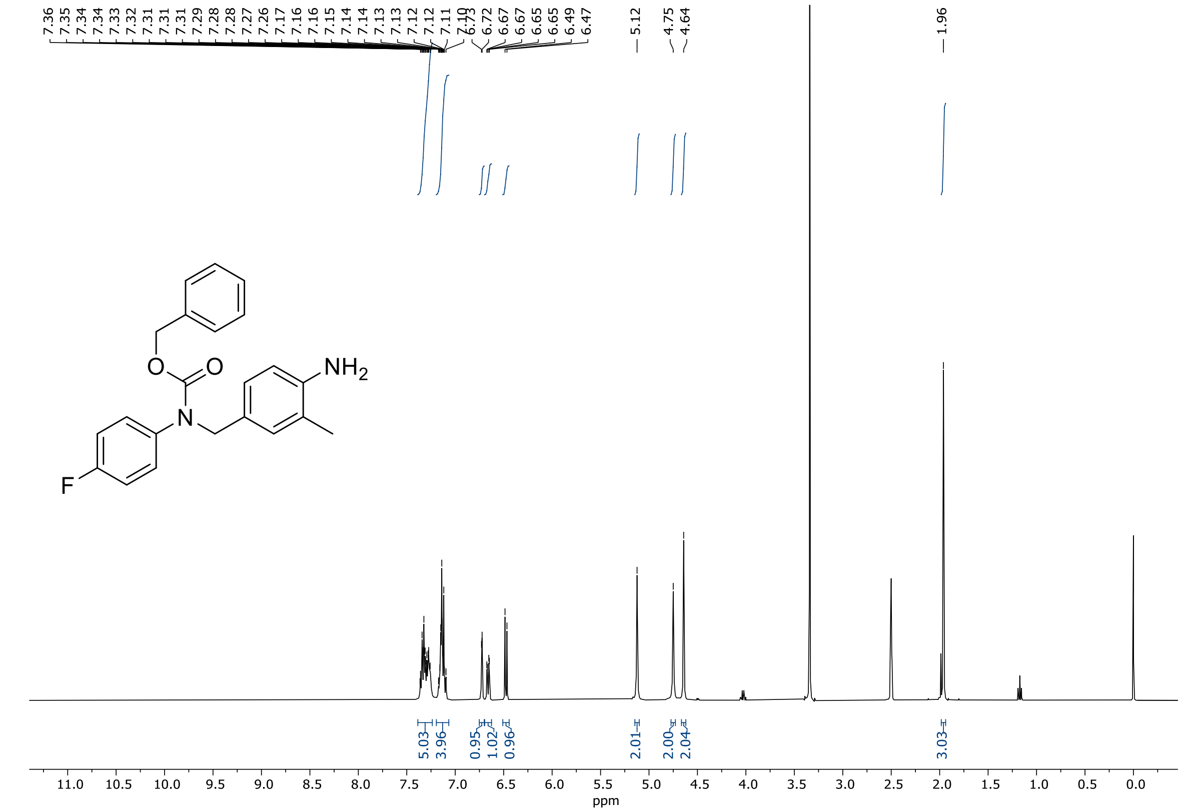

Figure S107: ^{1}H-NMR spectrum of compound **51**.

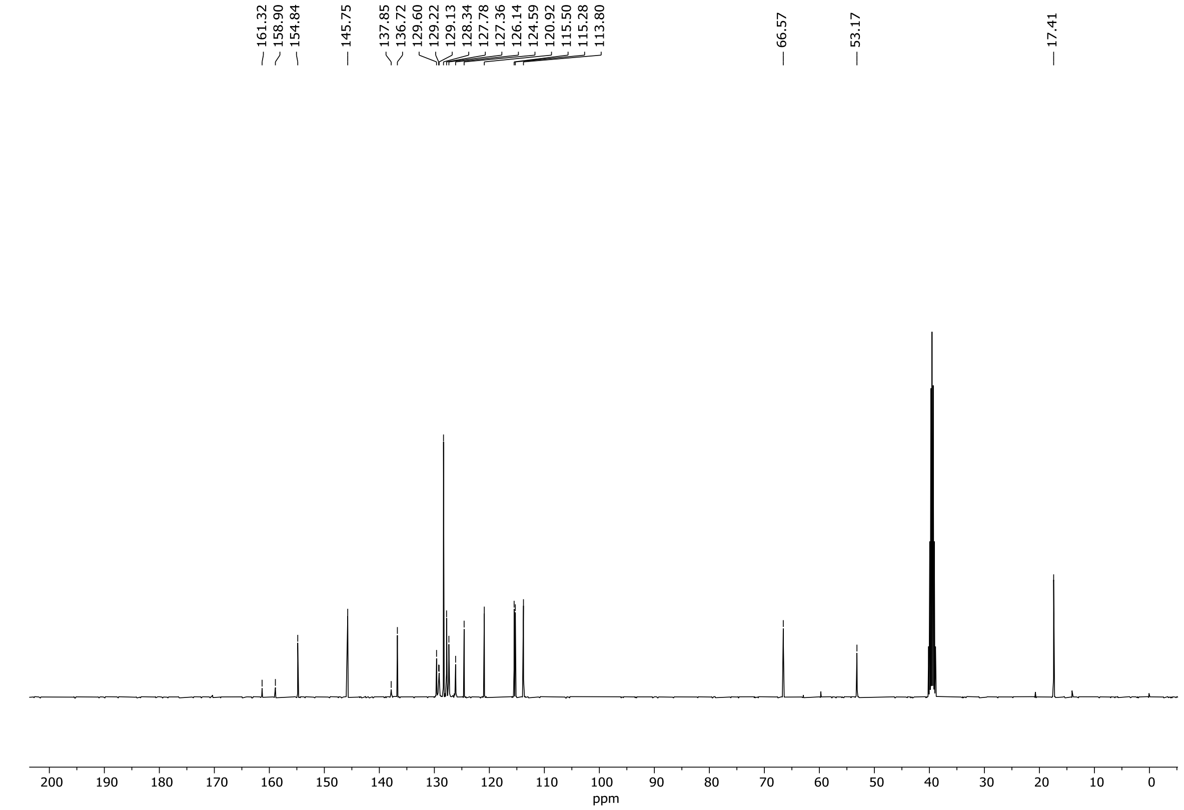

Figure S108: ^{13}C-NMR spectrum of compound **51**.

Benzyl {4-[3-(1*H*-pyrazol-1-yl)propanamido]-3-methylbenzyl}(4-fluorophenyl)carbamate (**52**)

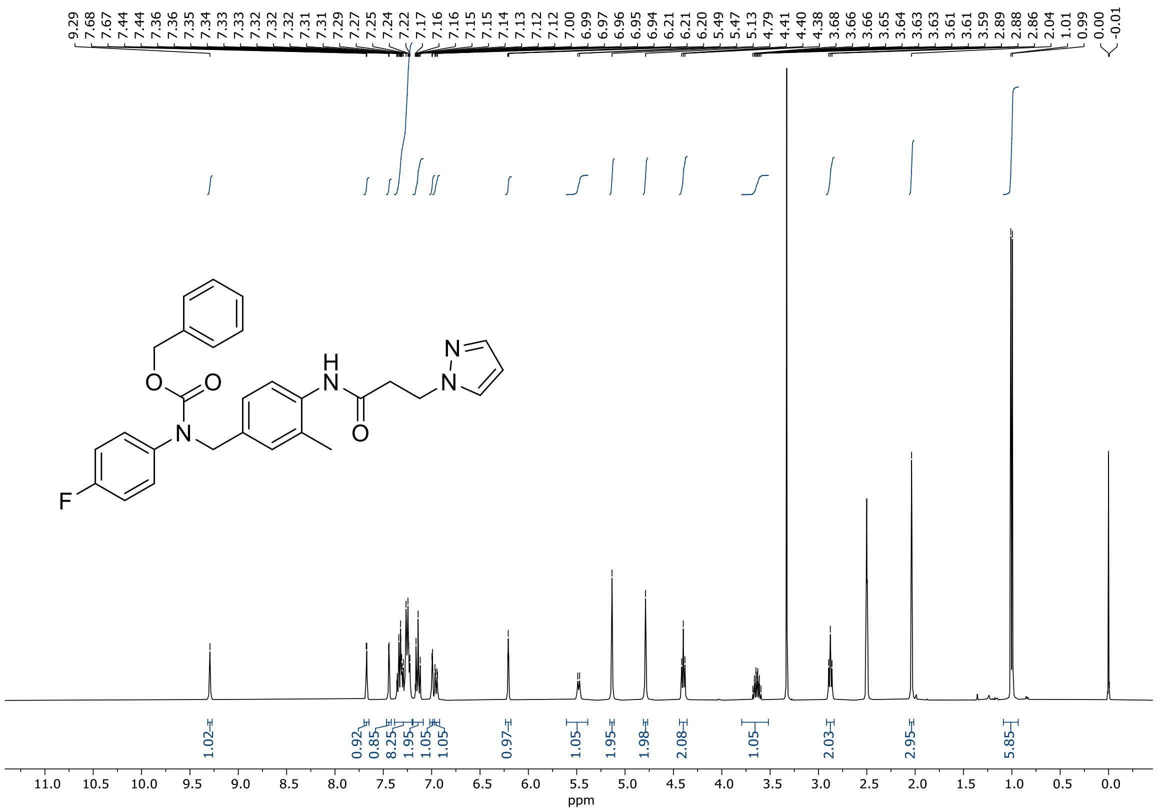

Figure S109: ^{1}H-NMR spectrum of compound **52** (spectrum contains signals of 1,3-diisopropyl urea: 5.48 (d, *J* = 7.8 Hz), 3.64 (m), 1.00 (d, *J* = 6.5 Hz)).

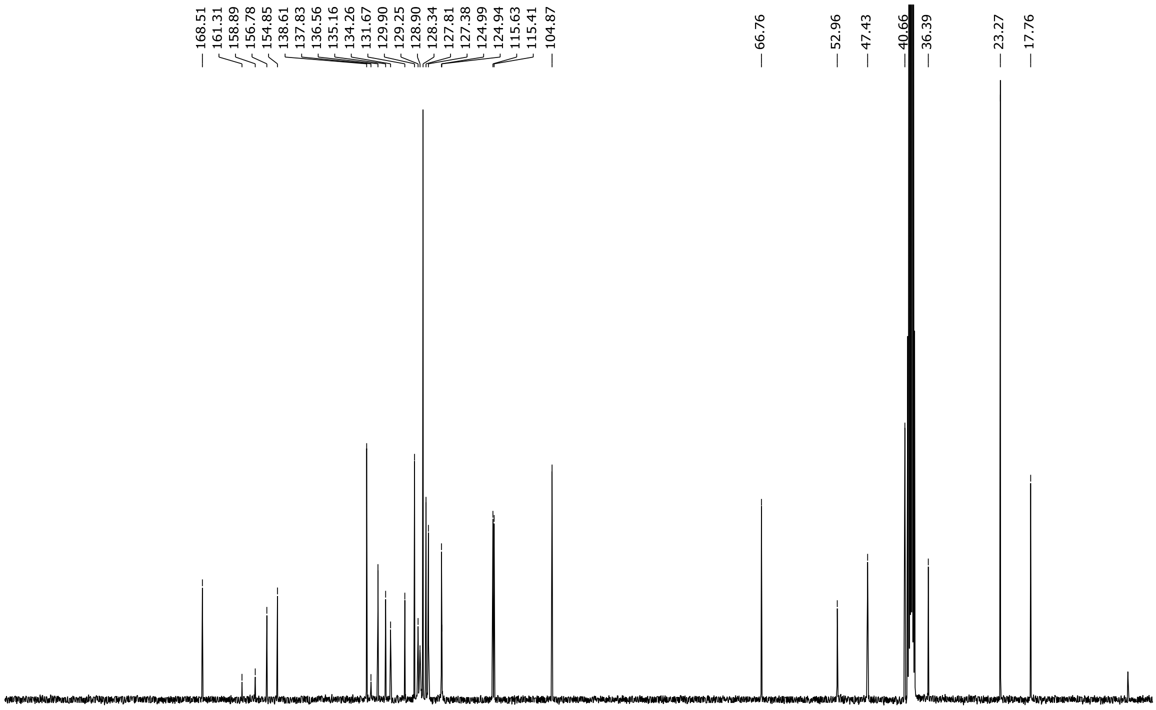

Figure S110: ^{13}C-NMR spectrum of compound **52** (spectrum contains signals of 1,3-diisopropyl urea: 156.8, 40.7, 23.3).

N-(4-{[(4-Fluorophenyl)amino]methyl}-2-methylphenyl)-3-(1*H*-pyrazol-1-yl)propanamide (**53**)

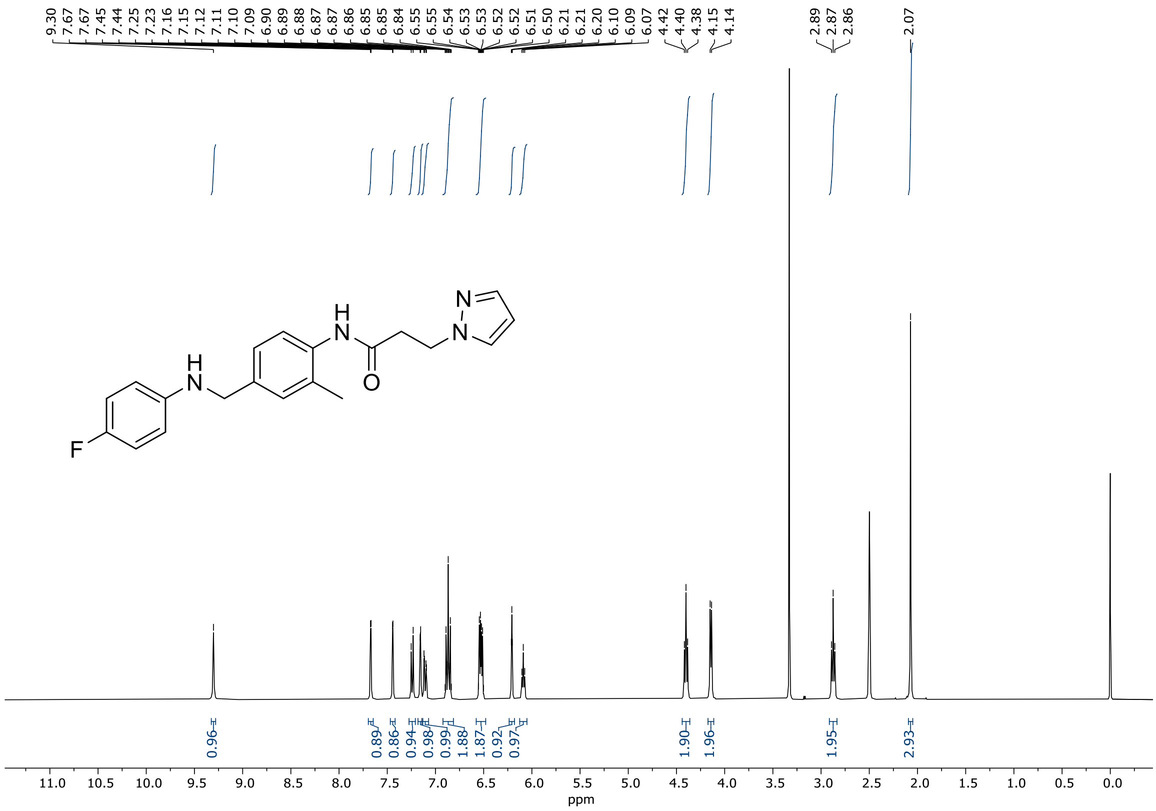

Figure S111: ^{1}H-NMR spectrum of compound **53**.

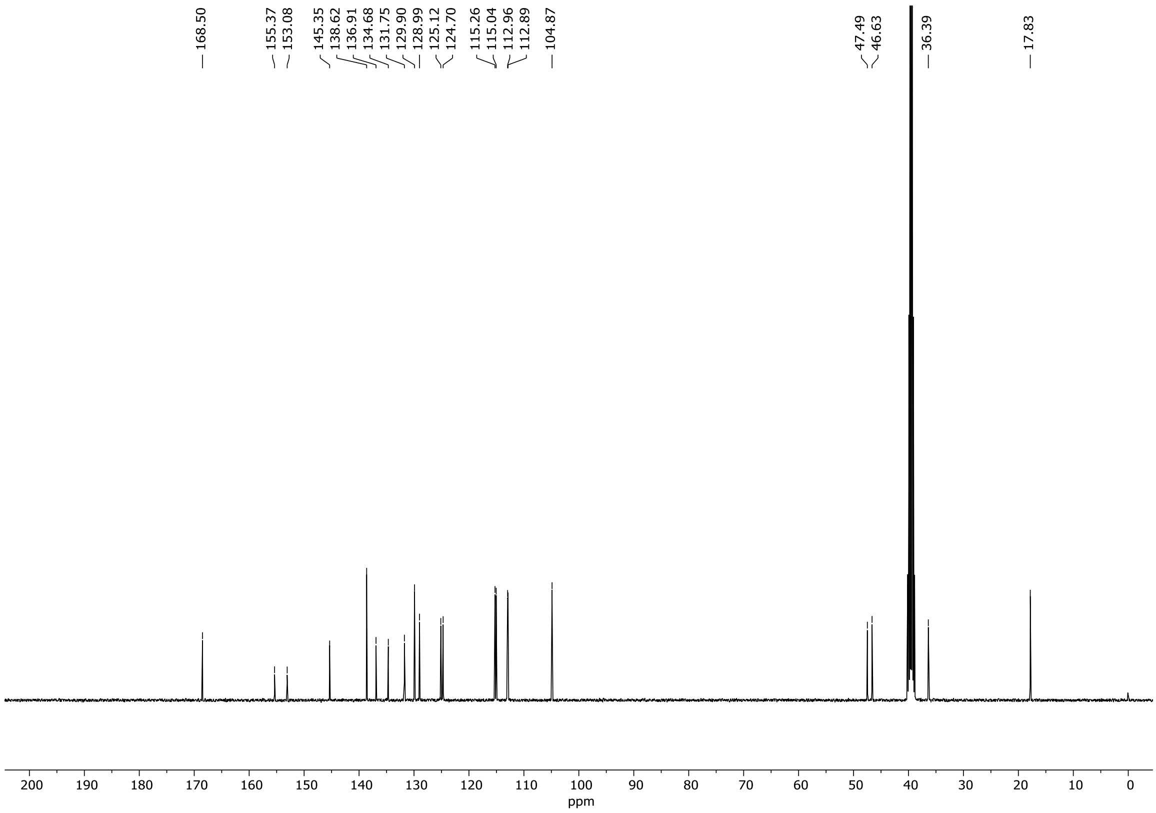

Figure S112: ^{13}C-NMR spectrum of compound **53**.

N-(2,4-Dimethylphenyl)-3-(1*H*-pyrazol-1-yl)propanamide (**54**)

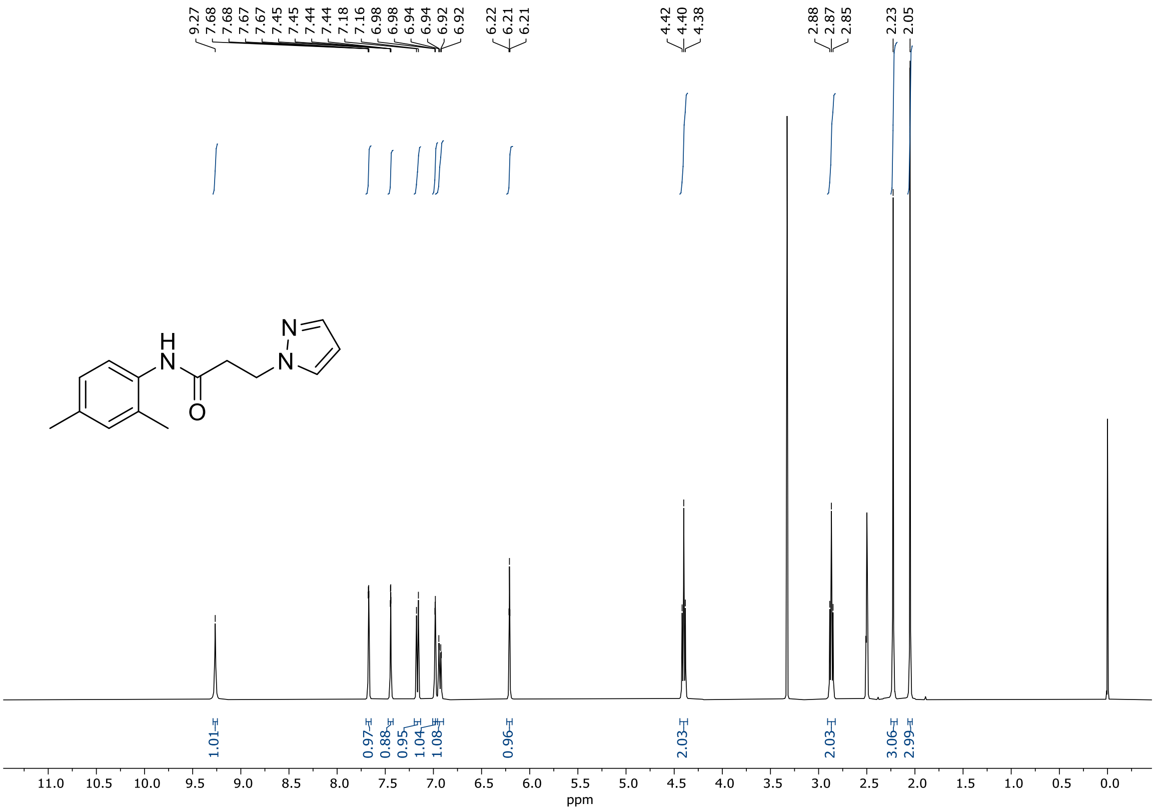

Figure S113: ^{1}H-NMR spectrum of compound **54**.

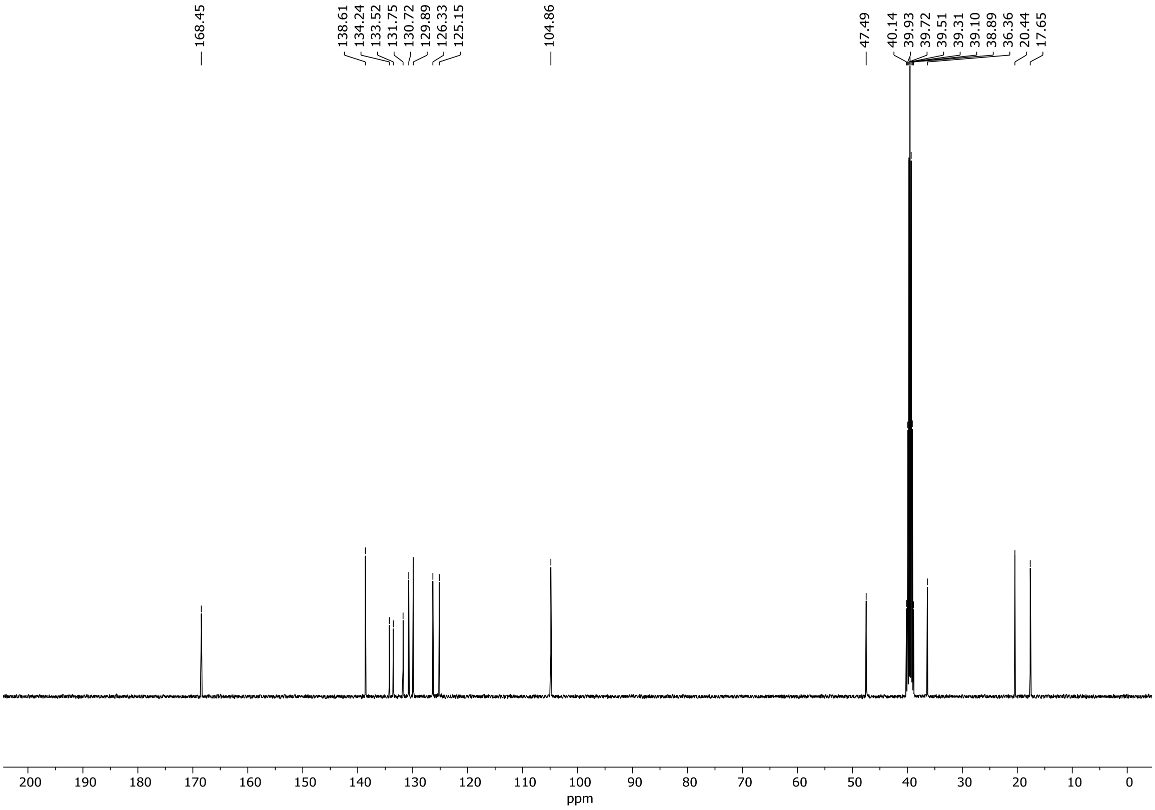

Figure S114: ^{13}C-NMR spectrum of compound **54**.

Methyl 3-(1*H*-pyrazol-1-yl)propanoate (**57**)

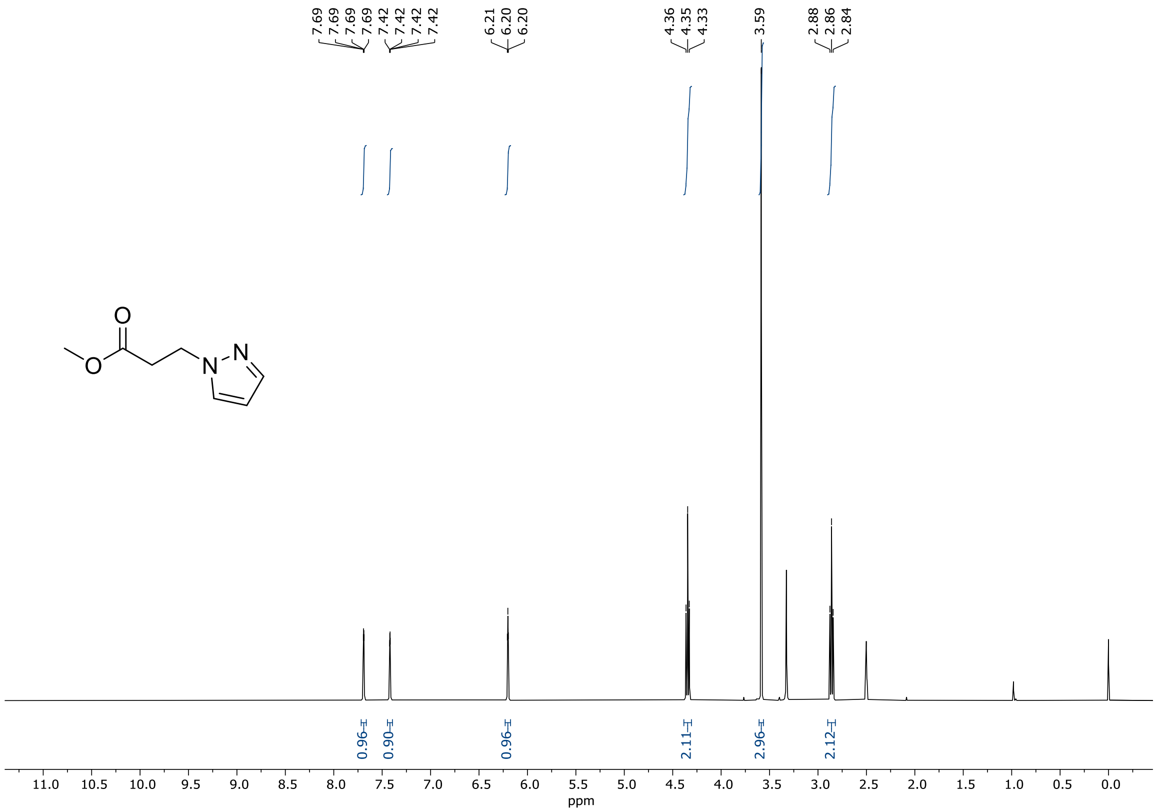

Figure S115: ^{1}H-NMR spectrum of compound **57**.

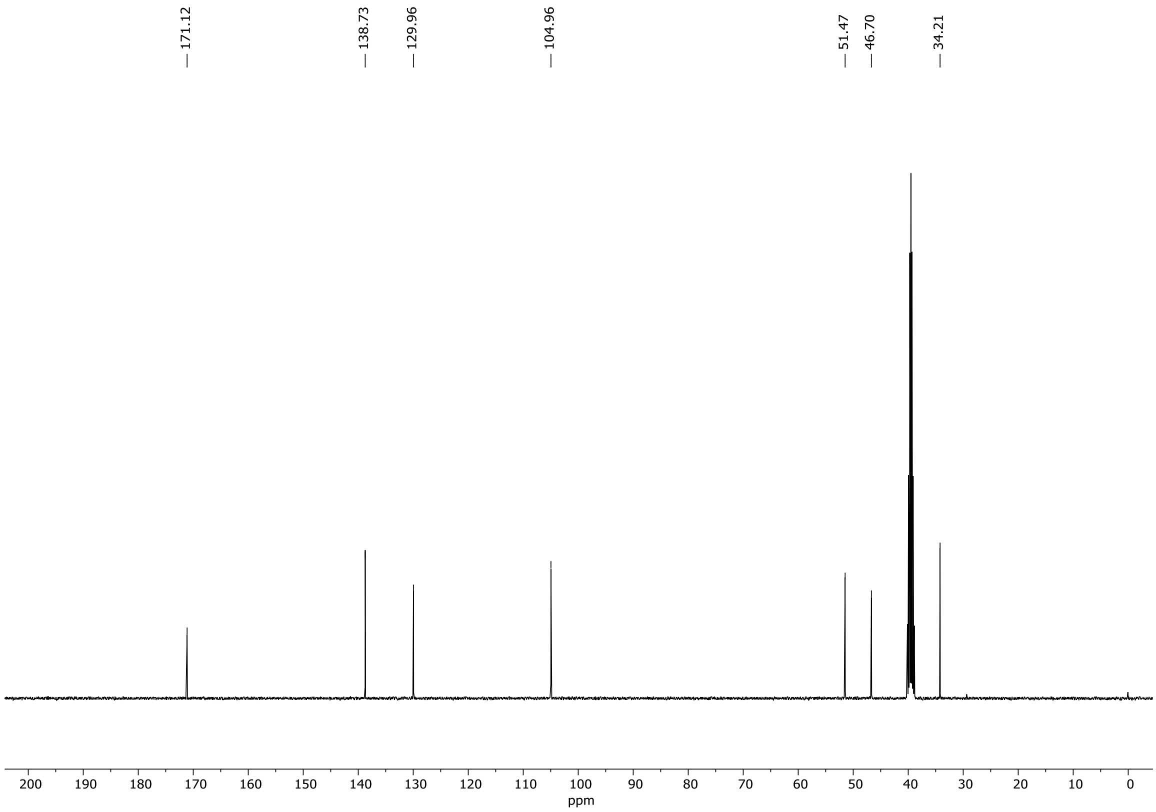

Figure S116: ^{13}C-NMR spectrum of compound **57**.

3-(1*H*-Pyrazol-1-yl)propanoic acid (**58**)

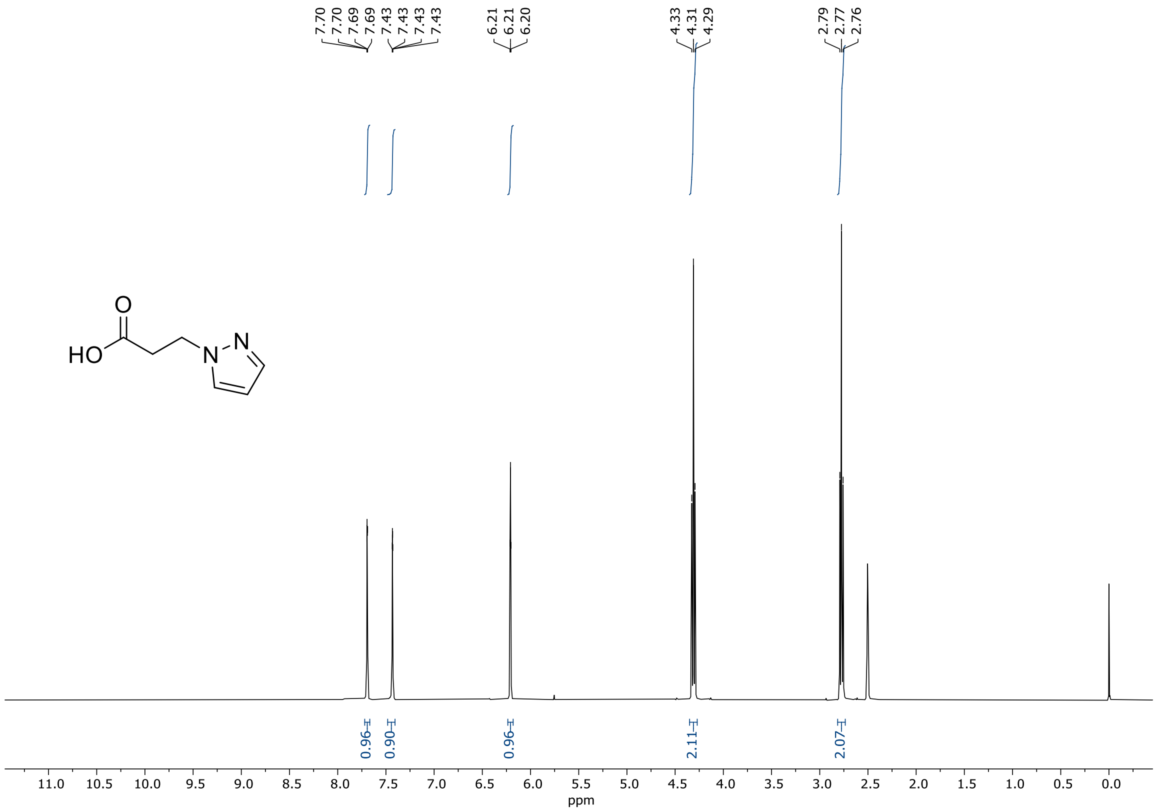

Figure S117: ^{1}H-NMR spectrum of compound **58**.

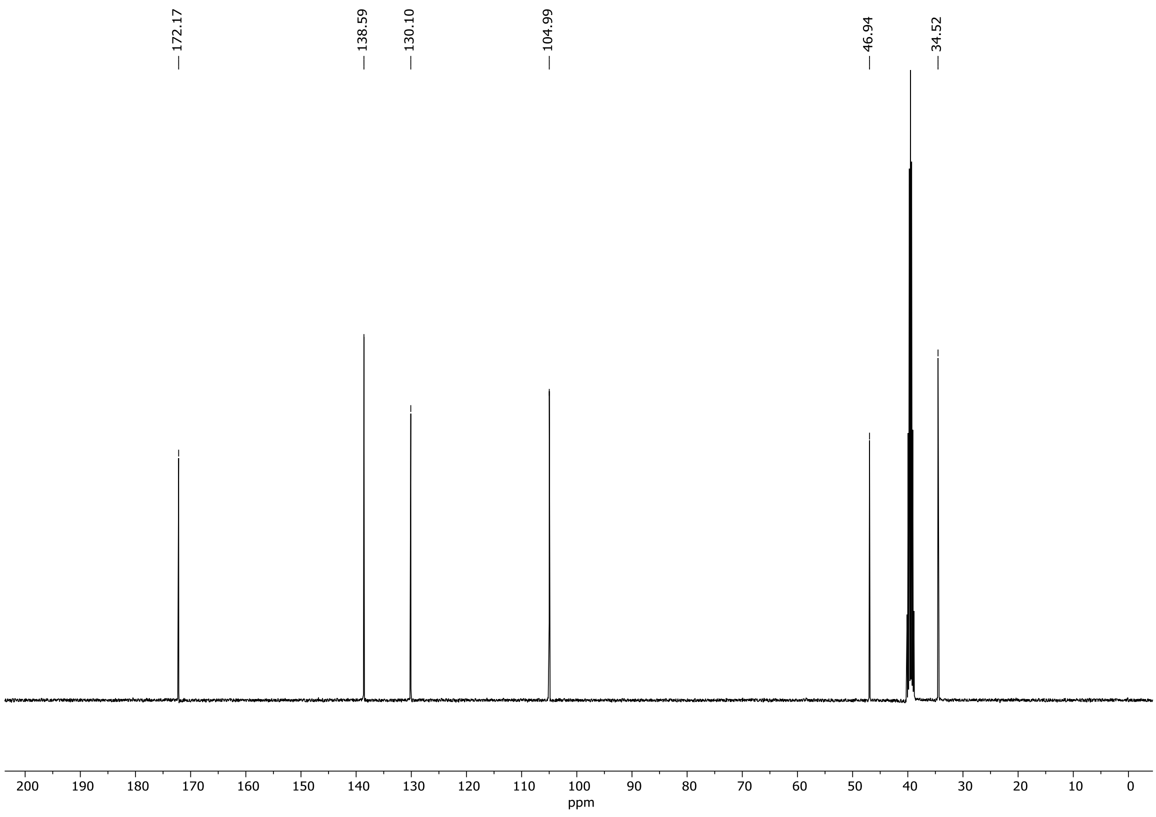

Figure S118: ^{13}C-NMR spectrum of compound **58**.

Benzyl (4-acrylamido-3-methylbenzyl)(4-fluorophenyl)carbamate (**59**)

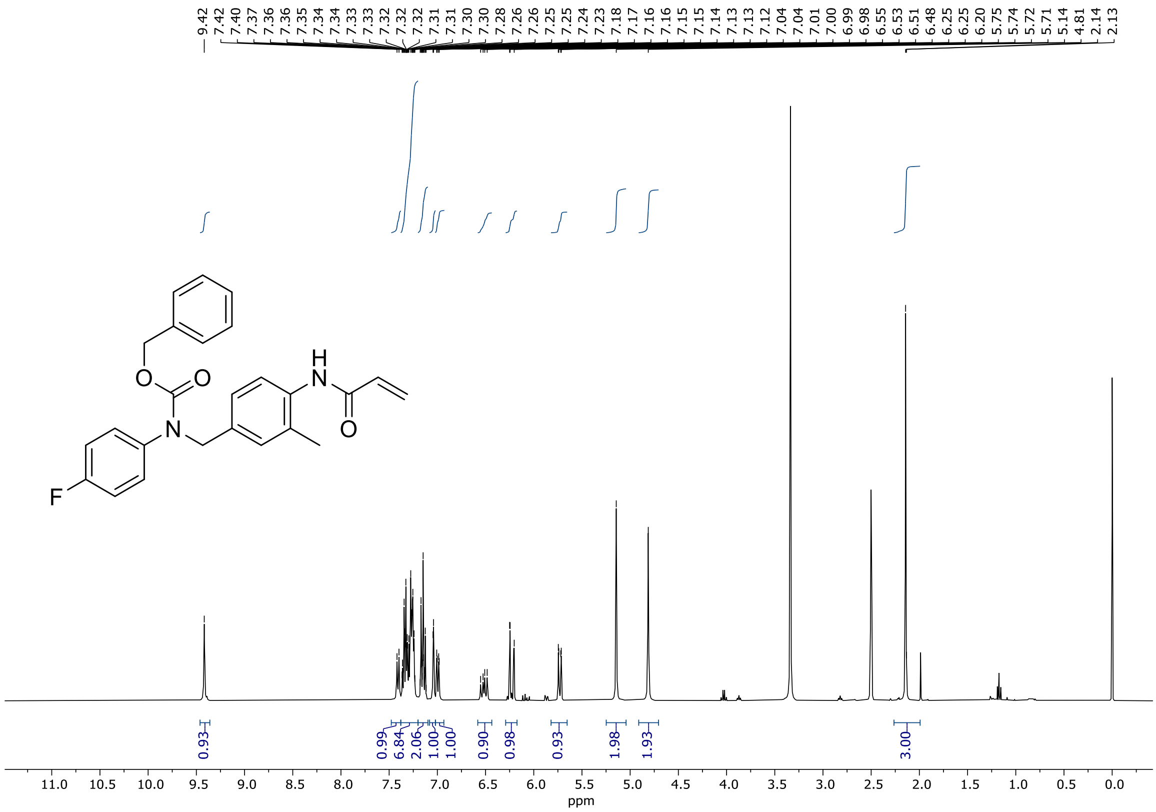

Figure S119: ^{1}H-NMR spectrum of compound **59**.

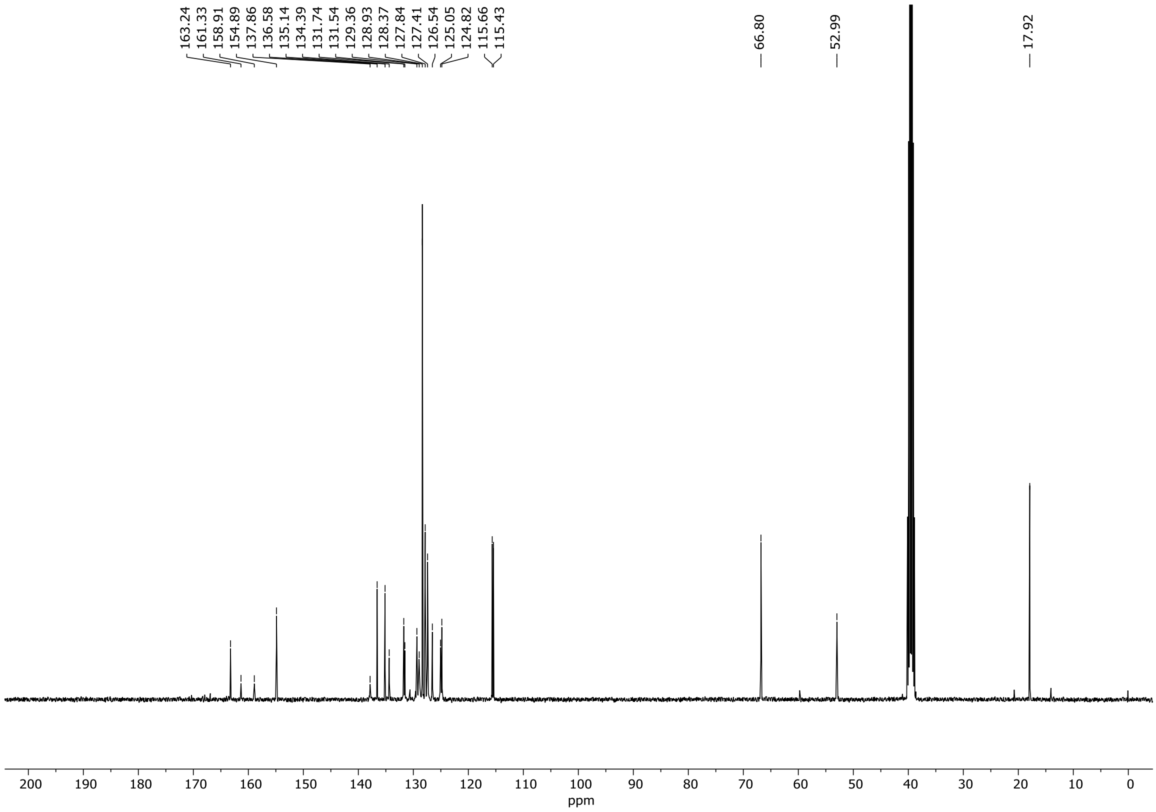

Figure S120: ^{13}C-NMR spectrum of compound **59**.

Benzyl (4-fluorophenyl){3-methyl-4-[3-(pyrrolidin-1-yl)propanamido]benzyl}carbamate (**60**)

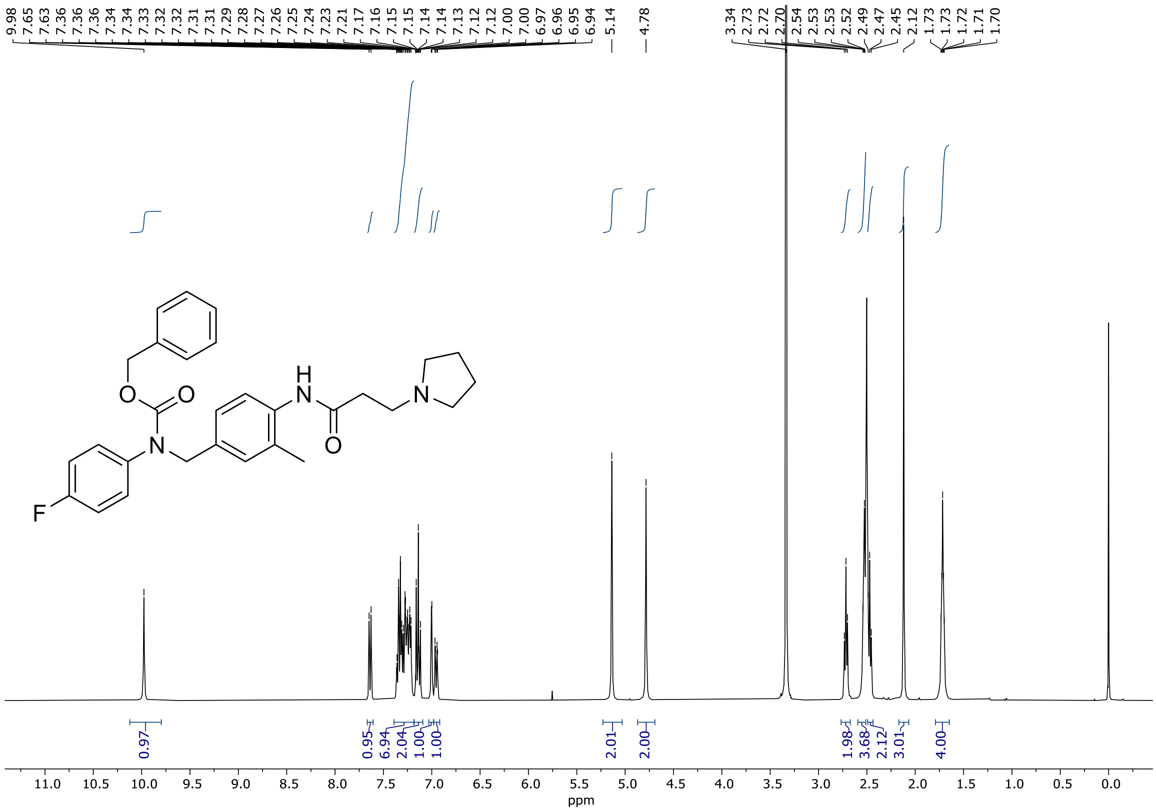

Figure S121: ^{1}H-NMR spectrum of compound **60**.

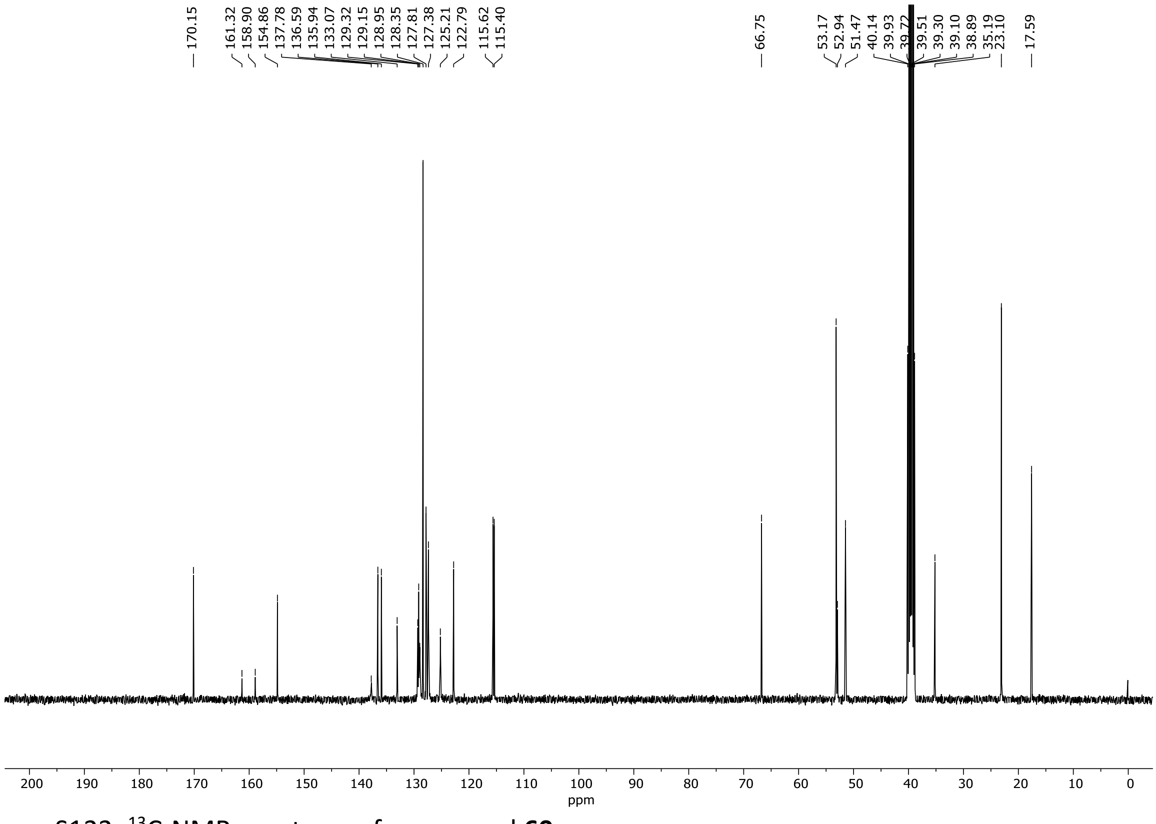

Figure S122: ^{13}C-NMR spectrum of compound **60**.

N-(4-{[(4-Fluorophenyl)amino]methyl}-2-methylphenyl)-3-(pyrrolidin-1-yl)propanamide 2,2,2-trifluoroacetate (**61**)

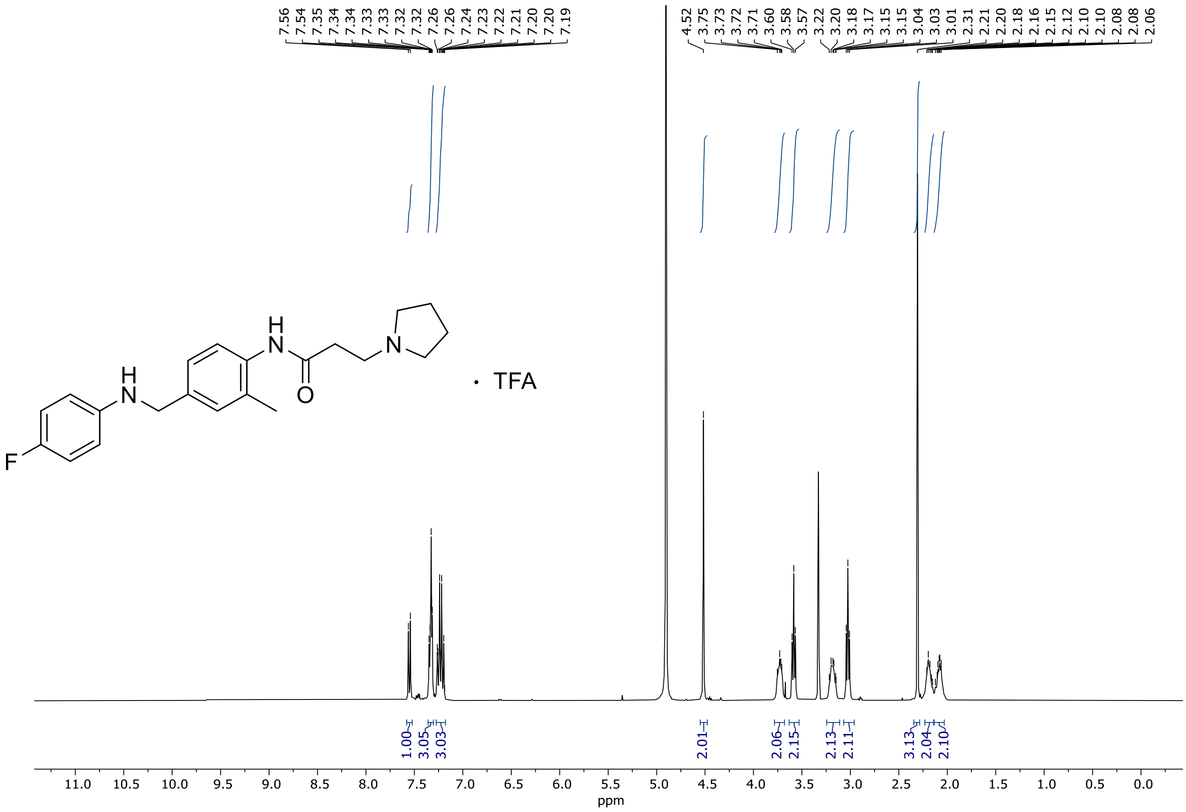

Figure S123: ^{1}H-NMR spectrum of compound **61**.

2 Assignement of NMR signals

Ethyl [2-amino-6-(4-fluorophenethyl)pyridin-3-yl]carbamate (**7**)

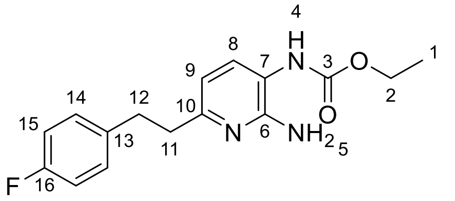

^{1}H-NMR (400 MHz, DMSO-d$_6$): δ(ppm) = 8.57 (s, 1H, 4-H), 7.44 (d, *J* = 6.6 Hz, 1H, 8-H), 7.28 – 7.18 (m, 2H, 14-H), 7.12 – 7.02 (m, 2H, 15-H), 6.41 (d, *J* = 7.8 Hz, 1H, 9-H), 5.70 (s, 2H, 5-H), 4.10 (q, *J* = 7.1 Hz, 2H, 2-H), 2.94 – 2.85 (m, 2H, 11-H), 2.80 – 2.71 (m, 2H, 12-H), 1.23 (t, *J* = 7.1 Hz, 3H, 1-H); **^{13}C-NMR (100 MHz, DMSO-d$_6$):** δ(ppm) = 160.6 (d, *J* = 241.0 Hz, 16), 154.5 (3), 152.3 (6), 137.8 (d, *J* = 3.0 Hz, 13), 131.0 (10), 130.0 (8), 130.0 (d, *J* = 7.9 Hz, 14), 116.4 (7), 114.8 (d, *J* = 20.9 Hz, 15), 110.9 (9), 60.3 (2), 38.7 (11), 34.2 (12), 14.5 (1).

[(4-Fluorophenyl)ethynyl]trimethylsilane (**10**)

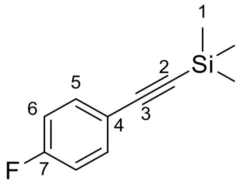

^{1}H-NMR (400 MHz, DMSO-d$_6$): δ(ppm) = 7.56 – 7.47 (m, 2H, 5-H), 7.27 – 7.16 (m, 2H, 6-H), 0.23 (s, 9H, 1-H); **^{13}C-NMR (100 MHz, DMSO-d$_6$):** δ(ppm) = 162.1 (d, *J* = 248.1 Hz, 7), 133.9 (d, *J* = 8.6 Hz, 5), 118.6 (d, *J* = 3.4 Hz, 4), 115.9 (d, *J* = 22.1 Hz, 6), 104.0 (3), 93.8 (2), -0.2 (1).

6-Chloro-3-nitropyridin-2-amine (**13**)

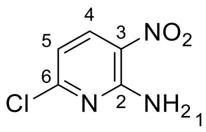

^{1}H-NMR (400 MHz, DMSO-d$_6$): δ(ppm) = 8.39 (d, *J* = 8.6 Hz, 1H, 4-H), 8.25 (s, 2H, 1-H), 6.77 (d, *J* = 8.7 Hz, 1H, 5-H); **^{13}C-NMR (100 MHz, DMSO-d$_6$):** δ(ppm) = 155.0 (6), 153.4 (2), 138.3 (4), 126.1 (3), 112.0 (5).

6-Bromo-3-nitropyridin-2-amine (**14**)

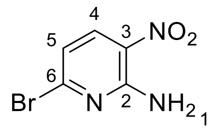

^{1}H-NMR (400 MHz, DMSO-d$_6$): δ(ppm) = 8.26 (d, *J* = 8.6 Hz, 1H, 4-H), 8.26 (s, 2H, 1-H), 6.90 (d, *J* = 8.5 Hz, 1H, 5-H); **^{13}C-NMR (100 MHz, DMSO-d$_6$):** δ(ppm) = 153.4 (2), 146.9 (6), 137.7 (4), 126.4 (3), 115.8 (5).

6-[(4-Fluorophenyl)ethynyl]-3-nitropyridin-2-amine (**15**)

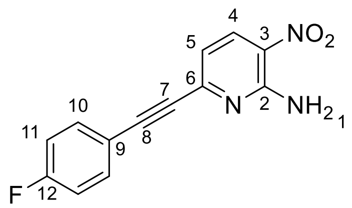

^{1}H-NMR (400 MHz, DMSO-d$_6$): δ(ppm) = 8.42 (d, *J* = 8.5 Hz, 1H, 4-H), 8.03 (s, 2H, 1-H), 7.75 – 7.67 (m, 2H, 10-H), 7.38 – 7.29 (m, 2H, 11-H), 6.96 (d, *J* = 8.5 Hz, 1H, 5-H); **^{13}C-NMR (100 MHz, DMSO-d$_6$):** δ(ppm) = 162.9 (d, *J* = 250.0 Hz, 12), 153.5 (2), 147.6 (6), 135.8 (4), 134.6 (d, *J* = 8.9 Hz, 10), 126.3 (3), 117.1 (d, *J* = 3.3 Hz, 9), 116.3 (d, *J* = 22.3 Hz, 11), 115.7 (5), 90.7 (7), 87.9 (8).

3-Nitro-6-(phenylethynyl)pyridin-2-amine (**16**)

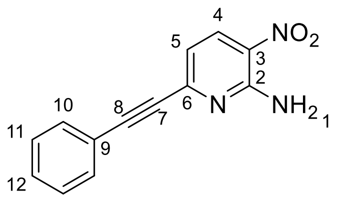

^{1}H-NMR (400 MHz, DMSO-d$_6$): δ(ppm) = 8.42 (d, *J* = 8.5 Hz, 1H, 4-H), 8.04 (s, 2H, 1-H), 7.67 – 7.60 (m, 2H, 10-H), 7.53 – 7.46 (m, 3H, 11-H, 12-H), 6.97 (d, *J* = 8.5 Hz, 1H, 5-H); **^{13}C-NMR (100 MHz, DMSO-d$_6$):** δ(ppm) = 153.7 (2), 147.7 (6), 135.7 (4), 132.0 (10), 130.2 (12), 129.0 (11), 126.3 (3), 120.6 (9), 115.7 (5), 91.7 (7), 88.1 (8).

3-Nitro-6-[(trimethylsilyl)ethynyl]pyridin-2-amine (**17**)

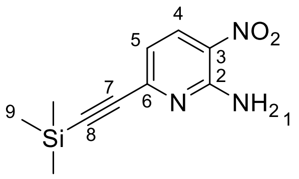

^{1}H-NMR (400 MHz, DMSO-d$_6$): δ(ppm) = 8.36 (d, *J* = 8.5 Hz, 1H, 4-H), 7.99 (s, 2H, 1-H), 6.83 (d, *J* = 8.5 Hz, 1H, 5-H), 0.25 (s, 9H, 9-H); **^{13}C-NMR (100 MHz, DMSO-d$_6$):** δ(ppm) = 153.4 (2), 147.0 (6), 135.8 (4), 126.5 (3), 115.5 (5), 103.1 (7), 97.9 (8), -0.61 (9).

6-Ethynyl-3-nitropyridin-2-amine (**18**)

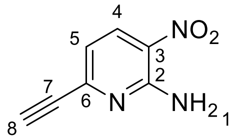

^{1}H-NMR (400 MHz, DMSO-d$_6$): δ(ppm) = 8.38 (d, *J* = 8.5 Hz, 1H, 4-H), 7.99 (s, 2H, 1-H), 6.87 (d, *J* = 8.5 Hz, 1H, 5-H), 4.63 (s, 1H, 8-H); **^{13}C-NMR (100 MHz, DMSO-d$_6$):** δ(ppm) = 153.4 (2), 147.0 (6), 135.8 (4), 126.7 (3), 115.7 (5), 83.5 (8), 82.0 (7).

Ethyl [2-amino-6-(phenylethynyl)pyridin-3-yl]carbamate (**21**)

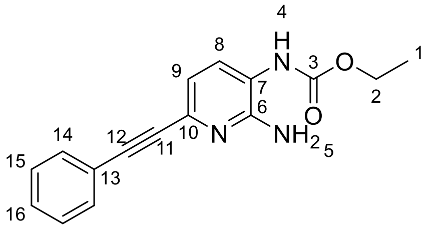

^{1}H-NMR (400 MHz, DMSO-d$_6$): δ(ppm) = 8.83 (s, 1H, 4-H), 7.75 (d, *J* = 7.9 Hz, 1H, 8-H), 7.58 – 7.49 (m, 2H, 14-H), 7.48 – 7.38 (m, 3H, 15-H, 16-H), 6.86 (d, *J* = 7.9 Hz, 1H, 9-H), 6.10 (s, 2H, 5-H), 4.14 (q, *J* = 7.1 Hz, 2H, 2-H), 1.26 (t, *J* = 7.1 Hz, 3H, 1-H); **^{13}C-NMR (100 MHz, DMSO-d$_6$):** δ(ppm) = 154.1 (3), 151.9 (10), 134.6 (6), 131.4 (14), 128.9 (16), 128.8 (15), 128.5 (8), 122.0 (13), 119.5 (7), 116.4 (9), 89.7 (11), 86.2 (12), 60.6 (2), 14.5 (1).

6-Methyl-5-nitropicolinonitrile (**23**)

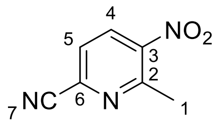

^{1}H-NMR (400 MHz, DMSO-d$_6$): δ(ppm) = 8.66 (d, *J* = 8.3 Hz, 1H, 4-H), 8.22 (dd, *J* = 8.3, 0.7 Hz, 1H, 5-H), 2.77 (s, 3H, 1-H); **^{13}C-NMR (100 MHz, DMSO-d$_6$):** δ(ppm) = 154.4 (2), 147.3 (3), 134.6 (4), 134.6 (6), 127.9 (5), 116.2 (7), 22.8 (1).

5-Amino-6-methylpicolinonitrile (**24**)

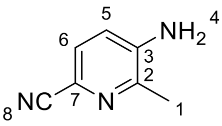

^{1}H-NMR (400 MHz, DMSO-d$_6$): δ(ppm) = 7.48 (d, *J* = 8.3 Hz, 1H, 6-H), 6.92 (d, *J* = 8.3 Hz, 1H, 5-H), 6.14 (s, 2H, 4-H), 2.28 (s, 3H, 1-H); **^{13}C-NMR (100 MHz, DMSO-d$_6$):** δ(ppm) = 146.1 (2), 144.5 (3), 128.1 (6), 119.2 (7), 117.8 (5), 116.7 (8), 20.4 (1).

Isobutyl (6-cyano-2-methylpyridin-3-yl)carbamate (**25**)

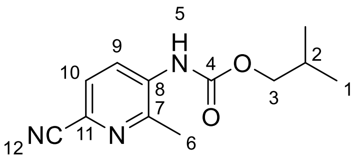

^{1}H-NMR (400 MHz, DMSO-d$_6$): δ(ppm) = 9.44 (s, 1H, 5-H), 8.16 (d, *J* = 8.4 Hz, 1H, 9-H), 7.85 (d, *J* = 8.4 Hz, 1H, 10-H), 3.93 (d, *J* = 6.7 Hz, 2H, 3-H), 2.50 (s, 3H, 6-H), 2.05 – 1.86 (m, 1H, 2-H), 0.94 (d, *J* = 6.7 Hz, 6H, 1-H); **^{13}C-NMR (100 MHz, DMSO-d$_6$):** δ(ppm) = 154.0 (4), 152.1 (7), 136.9 (8), 129.1 (10), 127.2 (9), 125.8 (11), 117.7 (12), 70.9 (3), 27.5 (2), 21.1 (6), 18.8 (1).

Isobutyl (6-formyl-2-methylpyridin-3-yl)carbamate (**26**)

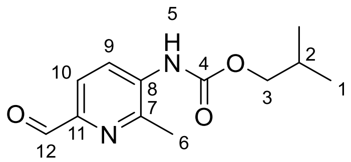

^{1}H-NMR (400 MHz, DMSO-d$_6$): δ(ppm) = 9.87 (d, *J* = 0.8 Hz, 1H, 12-H), 9.39 (s, 1H, 5-H), 8.17 (d, *J* = 8.3 Hz, 1H, 9-H), 7.80 (d, *J* = 8.4 Hz, 1H, 10-H), 3.93 (d, *J* = 6.7 Hz, 2H, 3-H), 2.56 (s, 3H, 6-H), 2.03 – 1.89 (m, 1H, 2-H), 0.95 (d, *J* = 6.7 Hz, 6H, 1-H); **^{13}C-NMR (100 MHz, DMSO-d$_6$):** δ(ppm) = 192.6 (12), 154.1 (4), 150.5 (7), 147.0 (11), 137.3 (8), 129.3 (9), 120.2 (10), 70.8 (3), 27.5 (2), 21.1 (6), 18.9 (1).

Isobutyl (6-{[(4-fluorophenyl)amino]methyl}-2-methylpyridin-3-yl)carbamate (**27**)

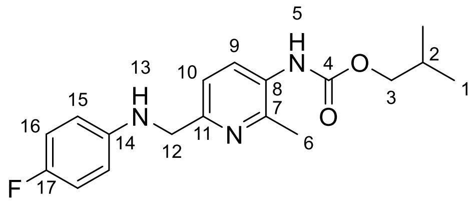

^{1}H-NMR (400 MHz, DMSO-d$_6$): δ(ppm) = 8.99 (s, 1H, 5-H), 7.63 (d, *J* = 8.2 Hz, 1H, 9-H), 7.14 (d, *J* = 8.2 Hz, 1H, 10-H), 6.93 – 6.82 (m, 2H, 16-H), 6.57 – 6.48 (m, 2H, 15-H), 6.22 (t, *J* = 6.1 Hz, 1H, 13-H), 4.24 (d, *J* = 6.1 Hz, 2H, 12-H), 3.85 (d, *J* = 6.7 Hz, 2H, 3-H), 2.41 (s, 3H, 6-H), 1.98 – 1.83 (m, 1H, 2-H), 0.91 (d, *J* = 6.7 Hz, 6H, 1-H); **^{13}C-NMR (100 MHz, DMSO-d$_6$):** δ(ppm) = 155.0 (4), 154.6 (8), 154.3 (d, *J* = 229.0 Hz, 17), 151.4 (7), 145.1 (14), 132.5 (10), 130.9 (11), 118.5 (9), 115.2 (d, *J* = 21.9 Hz, 16), 112.9 (d, *J* = 7.3 Hz, 15), 70.3 (3), 48.6 (12), 27.6 (2), 20.9 (6), 18.9 (1).

4-Nitro-3-(trifluoromethyl)benzonitrile (**29**)

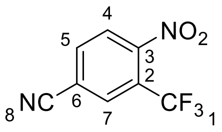

^{1}H-NMR (400 MHz, DMSO-d$_6$): δ(ppm) = 8.69 (d, *J* = 1.8 Hz, 1H, 7-H), 8,52 (dd, *J* = 8.4, 1.8 Hz, 1H, 5-H), 8.38 (d, *J* = 8.4 Hz, 1H, 4-H); **^{13}C-NMR (100 MHz, DMSO-d$_6$):** δ(ppm) = 149.3 (3), 139.1 (5), 132.6 (q, *J* = 5.1 Hz, 7), 126.2 (4), 122.2 (q, *J* = 34.3 Hz, 2), 121.3 (q, *J* = 273.3 Hz, 1), 116.2 (8), 116.2 (6).

4-Bromo-2,6-dimethylaniline (**31a**)

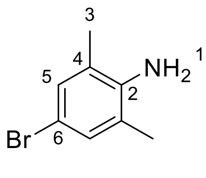

^{1}H-NMR (400 MHz, DMSO-d$_6$): δ(ppm) = 6.96 (s, 2H, 5-H), 4.69 (s, 2H, 1-H), 2.06 (s, 6H, 3-H); **^{13}C-NMR (100 MHz, DMSO-d$_6$):** δ(ppm) = 143.7 (4), 129.6 (5), 123.0 (2), 106.1 (6), 17.5 (3).

4-Amino-3,5-dimethylbenzonitrile (**32a**)

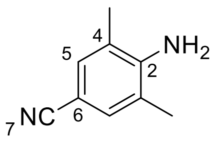

^{1}H-NMR (400 MHz, DMSO-d$_6$): δ(ppm) = 7.21 (s, 2H, 5-H), 5.57 (s, 2H, 1-H), 2.09 (s, 6H, 3-H); **^{13}C-NMR (100 MHz, DMSO-d$_6$):** δ(ppm) = 149.2 (4), 131.4 (5), 120.8 (2), 120.8 (6), 95.6 (7), 17.4 (3).

4-Amino-3-methylbenzonitrile (**32b**)

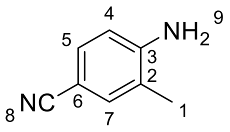

^{1}H-NMR (400 MHz, DMSO-d$_6$): δ(ppm) = 7.33 – 7.24 (m, 2H, 5-H, 7-H), 6.64 (d, *J* = 8.2 Hz, 1H, 4-H), 5.89 (s, 2H, 9-H), 2.05 (s, 3H, 1-H); **^{13}C-NMR (100 MHz, DMSO-d$_6$):** δ(ppm) = 151.2 (3), 133.5 (5), 131.1 (7), 121.2 (2), 120.7 (8), 113.4 (4), 95.8 (6), 17.0 (1).

4-Amino-3-fluorobenzonitrile (**32c**)

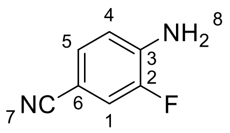

^{1}H-NMR (400 MHz, DMSO-d$_6$): δ(ppm) = 7.48 (dd, *J* = 11.7, 1.9 Hz, 1H, 1-H), 7.29 (dd, *J* = 8.3, 1.8 Hz, 1H, 5-H), 6.78 (dd, *J* = 9.0, 8.4 Hz 1H, 4-H), 6.21 (s, 2H, 8-H); **^{13}C-NMR (100 MHz, DMSO-d$_6$):** δ(ppm) = 148.9 (d, *J* = 239.4 Hz, 2), 141.8 (d, *J* = 12.7 Hz, 3), 129.9 (d, *J* = 2.5 Hz, 5), 119.4 (d, *J* = 2.6 Hz, 7), 118.5 (d, *J* = 21.3 Hz, 1), 115.6 (d, *J* = 5.5 Hz, 4), 95.4 (d, *J* = 8.5 Hz, 6).

4-Amino-3-(trifluoromethyl)benzonitrile (**32d**)

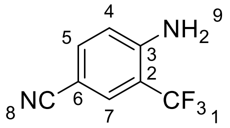

^{1}H-NMR (400 MHz, DMSO-d$_6$): δ(ppm) = 9.50 (s, 1H, 9-H), 9.25 (s, 1H, 9-H), 7.90 (d, *J* = 1.9 Hz, 1H, 7-H), 7.85 (dd, *J* = 8.7, 1.9 Hz, 1H, 5-H), 7.33 (d, *J* = 8.7 Hz, 1H, 4-H); **^{13}C-NMR (100 MHz, DMSO-d$_6$):** δ(ppm) = 150.8 (3), 137.1 (5), 130.7 (q, *J* = 5.7 Hz, 7), 123,3 (q, *J* = 270.3 Hz, 1), 118.9 (8), 112.8 (4), 110.1 (q, *J* = 31.7 Hz, 2), 98.2 (6).

N-(4-Cyano-2,6-dimethylphenyl)butyramide (**33a**)

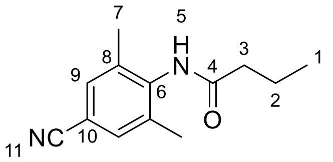

^{1}H-NMR (400 MHz, DMSO-d$_6$): δ(ppm) = 9.47 (s, 1H, 5-H), 7.59 – 7.54 (m, 2H, 9-H), 2.33 (t, *J* = 7.2 Hz, 2H, 3-H), 2.17 (s, 6H, 7-H), 1.71 – 1.57 (m, 2H, 2-H), 0.95 (t, *J* = 7.4 Hz, 3H, 1-H); **^{13}C-NMR (100 MHz, DMSO-d$_6$):** δ(ppm) = 170.7 (4), 140.3 (8), 136.8 (6), 131.2 (9), 118.8 (10), 108.7 (11), 37.3 (3), 18.7 (2), 17.9 (7), 13.7 (1).

N-(4-Cyano-2-methylphenyl)butyramide (**33b**)

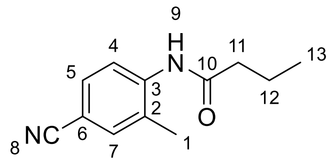

^{1}H-NMR (400 MHz, DMSO-d$_6$): δ(ppm) = 9.39 (s, 1H, 9-H), 7.81 (d, *J* = 8.3 Hz, 1H, 4-H), 7.68 (dd, *J* = 1.8, 0.9 Hz, 1H, 7-H), 7.61 (dd, *J* = 8.4, 2.0 Hz, 1H, 5-H), 2.39 (t, *J* = 7.3 Hz, 2H, 11-H), 2.26 (s, 3H, 1-H), 1.70 – 1.56 (m, 2H, 12-H), 0.93 (t, *J* = 7.4 Hz, 3H, 13-H); **^{13}C-NMR (100 MHz, DMSO-d$_6$):** δ(ppm) = 171.6 (10), 141.2 (3), 134.0 (7), 131.4 (2), 130.1 (5), 124.0 (4), 119.0 (6), 106.3 (8), 37.9 (11), 18.6 (12), 17.6 (1), 13.6 (13).

N-(4-Cyano-2-fluorophenyl)butyramide (**33c**)

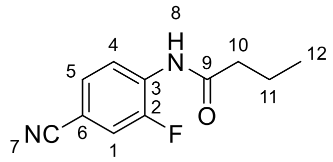

^{1}H-NMR (400 MHz, DMSO-d$_6$): δ(ppm) = 10.03 (s, 1H, 8-H), 8.26 (pseudo-t, *J* = 8.2 Hz, 1H, 4-H), 7.86 (dd, *J* = 11.1, 1.9 Hz, 1H, 1-H), 7.66 – 7.60 (m, 1H, 5-H), 2.41 (t, *J* = 7.3 Hz, 2H, 10-H), 1.64 – 1.52 (m, 2H, 11-H), 0.89 (t, *J* = 7.4 Hz, 3H, 12-H); **^{13}C-NMR (100 MHz, DMSO-d$_6$):** δ(ppm) = 172.3 (9), 151.7 (d, *J* = 247.3 Hz, 2), 131.6 (d, *J* = 11.1 Hz, 3), 129.3 (d, *J* = 3.5 Hz, 5), 123.0 (d, *J* = 2.7 Hz, 4), 119.3 (d, *J* = 23.4 Hz, 1), 117.9 (d, *J* = 2.6 Hz, 7), 105.7 (d, *J* = 9.4 Hz, 6), 37.8 (10), 18.4 (11), 13.5 (12).

N-[4-Cyano-2-(trifluoromethyl)phenyl]butyramide (**33d**)

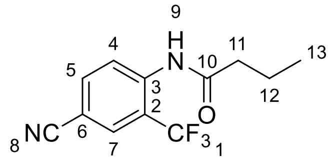

^{1}H-NMR (400 MHz, DMSO-d$_6$): δ(ppm) = 9.71 (s, 1H, 9-H), 8.28 (d, *J* = 1.9 Hz, 1H, 7-H), 8.13 (dd, *J* = 8.4, 1.9 Hz, 1H, 5-H), 7.81 (d, *J* = 8.2 Hz, 1H, 4-H), 2.38 (t, *J* = 7.3 Hz, 2H, 11-H), 1.68 – 1.54 (m, 2H, 12-H), 0.92 (t, *J* = 7.4 Hz, 3H, 13-H); **^{13}C-NMR (100 MHz, DMSO-d$_6$):** δ(ppm) = 172.2 (10), 139.9 (3), 136.6 (5), 131.0 (q, *J* = 5.0 Hz, 7), 130.0 (4), 124.2 (q, *J* = 30.0 Hz, 2), 122.6 (q, *J* = 272.0 Hz, 1), 117.5 (8), 108.7 (6), 37.5 (11), 18.5 (12), 13.4 (13).

N-(4-Cyano-2-methylphenyl)nicotinamide (**33e**)

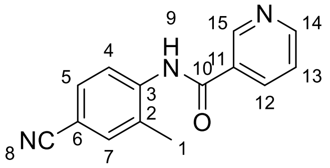

^{1}H-NMR (400 MHz, DMSO-d$_6$): δ(ppm) = 10.25 (s, 1H, 9-H), 9.14 (dd, *J* = 2.4, 0.9 Hz, 1H, 15-H), 8.80 (dd, *J* = 4.8, 1.7 Hz, 1H, 14-H), 8.32 (ddd, *J* = 7.9, 2.3, 1.7 Hz, 1H, 12-H), 7.83 – 7.77 (m, 1H, 5-H), 7.76 – 7.67 (m, 2H, 4-H, 7-H), 7.60 (ddd, *J* = 7.9, 4.8, 0.9 Hz, 1H, 13-H), 2.34 (d, *J* = 0.8 Hz, 3H, 1-H); **^{13}C-NMR (100 MHz, DMSO-d$_6$):** δ(ppm) = 164.7 (10), 152.9 (14), 149.3 (15), 141.2 (3), 136.1 (12), 134.7 (5), 134.6 (2), 130.6 (11), 130.3 (7), 126.7 (4), 124.0 (13), 119.3 (8), 108.5 (6), 18.1 (1).

N-(4-Formyl-2,6-dimethylphenyl)butyramide (**34a**)

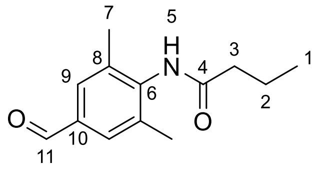

^{1}H-NMR (400 MHz, DMSO-d$_6$): δ(ppm) = 9.92 (s, 1H, 11-H), 9.45 (s, 1H, 5-H), 7.61 (s, 2H, 9-H), 2.34 (t, *J* = 7.3 Hz, 2H, 3-H), 2.22 (s, 6H, 7-H), 1.72 – 1.58 (m, 2H, 2-H), 0.96 (t, *J* = 7.4 Hz, 3H, 1-H); **^{13}C-NMR (100 MHz, DMSO-d$_6$):** δ(ppm) = 192.5 (11), 170.6 (4), 141.4 (8), 136.1 (6), 134.0 (10), 128.8 (9), 37.4 (3), 18.8 (2), 18.2 (7), 13.7 (1).

N-(4-Formyl-2-methylphenyl)butyramide (**34b**)

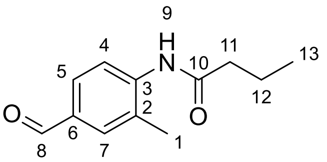

^{1}H-NMR (400 MHz, DMSO-d$_6$): δ(ppm) = 9.89 (s, 1H, 8-H), 9.37 (s, 1H, 9-H), 7.85 (d, *J* = 8.2 Hz, 1H, 4-H), 7.74 (d, *J* = 1.6 Hz, 1H 7-H), 7.71 (dd, J = 8.2, 2.0 Hz, 1H, 5-H), 2.40 (t, *J* = 7.3 Hz, 2H, 11-H), 2.31 (s, 3H, 1-H), 1.71 – 1.57 (m, 2H, 12-H), 0.94 (t, *J* = 7.4 Hz, 3H, 13-H); **^{13}C-NMR (100 MHz, DMSO-d$_6$):** δ(ppm) = 192.4 (8), 172.0 (10), 142.9 (3), 132.7 (6), 132.1 (7), 131.1 (2), 128.2 (5), 124.1 (4), 38.4 (11), 19.1 (12), 18.3 (1), 14.1 (13).

N-(2-Fluoro-4-formylphenyl)butyramide (**34c**)

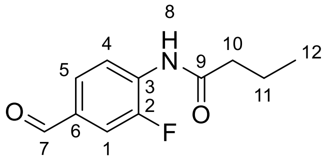

^{1}H-NMR (400 MHz, DMSO-d$_6$): δ(ppm) = 9.99 (s, 1H, 8-H), 9.88 (d, *J* = 1.9 Hz, 1H, 7-H), 8.32 (dd, *J* = 8.6, 7.5 Hz, 1H, 5-H), 7.77 – 7.68 (m, 2H, 1-H, 4-H), 2.43 (t, *J* = 7.3 Hz, 2H, 10-H), 1.67 – 1.55 (m, 2H, 11-H), 0.90 (t, *J* = 7.4 Hz, 3H, 12-H); **^{13}C-NMR (100 MHz, DMSO-d$_6$):** δ(ppm) = 191.0 (d, *J* = 2.1 Hz, 7), 172.2 (9), 152.5 (d, *J* = 247.5 Hz, 2), 132.4 (d, *J* = 11.5 Hz, 6), 132.1 (d, *J* = 5.5 Hz, 3), 126.8 (d, *J* = 2.9 Hz, 4), 122.5 (d, *J* = 2.1 Hz, 5), 115.2 (d, *J* = 19.8 Hz, 1), 37.9 (10), 18.4 (11), 13.5 (12).

N-[4-Formyl-2-(trifluoromethyl)phenyl]butyramide (**34d**)

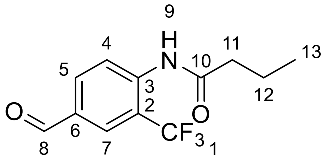

^{1}H-NMR (400 MHz, DMSO-d$_6$): δ(ppm) = 10.04 (s, 1H, 8-H), 9.68 (s, 1H, 9-H), 8.25 (d, *J* = 1.9 Hz, 1H, 7-H), 8.16 (dd, *J* = 8.1, 1.7 Hz, 1H, 5-H), 7.84 (d, *J* = 8.2 Hz, 1H, 4-H), 2.39 (t, *J* = 7.3 Hz, 2H, 11-H), 1.69 – 1.55 (m, 2H, 12-H), 0.93 (t, *J* = 7.4 Hz, 3H, 13-H); **^{13}C-NMR (100 MHz, DMSO-d$_6$):** δ(ppm) = 191.5 (8), 172.2 (10), 140.7 (3), 133.3 (6), 132.8 (5), 129.8 (4), 128.2 (q, *J* = 5.1 Hz, 7), 123.9 (q, *J* = 30.0 Hz, 2), 123.1 (q, *J* = 272.0 Hz, 1), 37.6 (11), 18.5 (12), 13.4 (13).

N-(4-Formyl-2-methylphenyl)nicotinamide (**34e**)

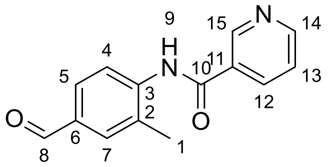

^{1}H-NMR (400 MHz, DMSO-d$_6$): δ(ppm) = 10.23 (s, 1H, 9-H), 9.97 (s, 1H, 8-H), 9.15 (dd, *J* = 2.4, 0.9 Hz, 1H, 15-H), 8.79 (dd, *J* = 4.8, 1.7 Hz, 1H, 14-H), 8.33 (ddd, *J* = 7.9, 2.4, 1.7 Hz, 1H, 12-H), 7.84 (d, *J* = 1.9 Hz, 1H, 7-H), 7.79 (dd, *J* = 8.2, 1.9 Hz, 1H, 5-H), 7.75 (d, *J* = 8.2 Hz, 1H, 4-H), 7.60 (ddd, *J* = 7.9, 4.8, 0.9 Hz, 1H, 13-H), 2.38 (s, 3H, 1-H); **^{13}C-NMR (100 MHz, DMSO-d$_6$):** δ(ppm) = 192.3 (8), 164.2 (10), 152.4 (14), 148.8 (15), 141.8 (3), 135.6 (2), 133.5 (12), 133.4 (6), 131.7 (7), 129.9 (11), 127.6 (5), 125.9 (4), 123.6 (13), 17.9 (1).

N-(4-{[(4-Fluorophenyl)amino]methyl}-2,6-dimethylphenyl)butyramide (**35a**)

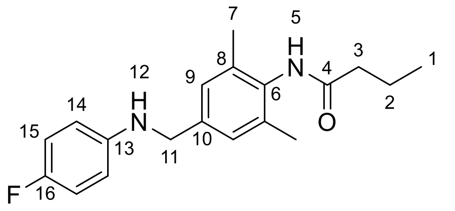

^{1}H-NMR (400 MHz, DMSO-d$_6$): δ(ppm) = 9.09 (s, 1H, 5-H), 7.03 (s, 2H, 9-H), 6.93 – 6.82 (m, 2H, 15-H), 6.58 – 6.48 (m, 2H, 14-H), 6.08 (t, *J* = 5.9 Hz, 1H, 12-H), 4.12 (d, *J* = 5.9 Hz, 2H, 11-H), 2.27 (t, *J* = 7.3 Hz, 2H, 3-H), 2.10 (s, 6H, 7-H), 1.70 – 1.56 (m, 2H, 2-H), 0.94 (t, *J* = 7.4 Hz, 3H, 1-H); **^{13}C-NMR (100 MHz, DMSO-d$_6$):** δ(ppm) = 170.7 (4), 154.2 (d, *J* = 230.7 Hz, 16), 145.4 (13), 137.8 (10), 134.9 (6), 134.0 (8), 126.4 (9), 115.1 (d, *J* = 21.8 Hz, 15), 112.9 (d, *J* = 7.3 Hz, 14), 46.7 (11), 37.4 (3), 18.9 (2), 18.2 (7), 13.7 (1).

N-(4-{[(4-Fluorophenyl)amino]methyl}-2-methylphenyl)butyramide (**35b**)

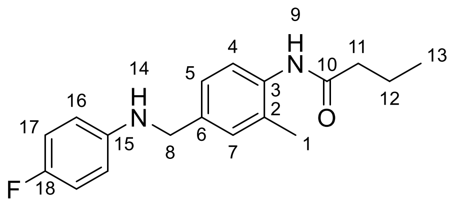

^{1}H-NMR (400 MHz, DMSO-d$_6$): δ(ppm) = 9.17 (s, 1H, 9-H), 7.27 (d, *J* = 8.1 Hz, 1H, 4-H), 7.17 (d, *J* = 2.1 Hz, 1H, 7-H), 7.11 (dd, *J* = 8.2, 2.1 Hz, 1H, 5-H), 6.92 – 6.81 (m, 2H, 16-H), 6.58 – 6.48 (m, 2H, 17-H), 6.09 (t, *J* = 6.0 Hz, 1H, 14-H), 4.15 (d, *J* = 5.9 Hz, 2H, 8-H), 2.28 (t, *J* = 7.3 Hz, 2H, 11-H), 2.15 (s, 3H, 1-H), 1.68 – 1.54 (m, 2H, 12-H), 0.93 (t, *J* = 7.4 Hz, 3H, 13-H); **^{13}C-NMR (100 MHz, DMSO-d$_6$):** δ(ppm) = 171.0 (10), 154.2 (d, *J* = 230.7 Hz, 18), 145.4 (d, *J* = 1.6 Hz, 15), 136.7 (6), 135.0 (3), 131.8 (2), 129.0 (7), 125.3 (4), 124.7 (5), 115.1 (d, *J* = 22.0 Hz, 17), 112.9 (d, *J* = 7.3 Hz, 16), 46.6 (8), 37.7 (11), 18.8 (12), 18.0 (1), 13.6 (13).

N-(2-Fluoro-4-{[(4-fluorophenyl)amino]methyl}phenyl)butyramide (**35c**)

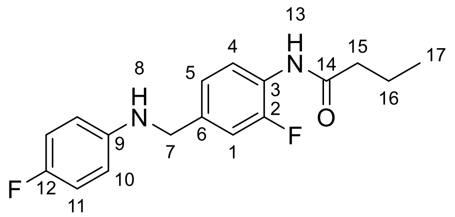

^{1}H-NMR (400 MHz, DMSO-d$_6$): δ(ppm) = 9.55 (s, 1H, 13-H), 7.71 (pseudo-t, *J* = 8.2 Hz, 1H, 5-H), 7.16 (dd, *J* = 11.8, 1.9 Hz, 1H, 1-H), 7.10 (dd, *J* = 8.3, 1.9 Hz, 1H, 4-H), 6.91 – 6.81 (m, 2H, 11-H), 6.57 – 6.47 (m, 2H, 10-H), 6.16 (t, *J* = 6.1 Hz, 1H, 8-H), 4.19 (d, *J* = 6.1 Hz, 2H, 7-H), 2.30 (d, *J* = 14.6 Hz, 1H, 15-H),

1.62 – 1.50 (m, 2H, 16-H), 0.89 (t, *J* = 7.4 Hz, 3H, 17-H); **^{13}C-NMR (100 MHz, DMSO-d$_6$):** δ(ppm) = 171.4 (14), 154.4 (d, *J* = 230 Hz, 2), 153.9 (d, *J* = 244 Hz, 12), 145.1 (d, *J* = 1.5 Hz, 9), 138.0 (d, *J* = 6.4 Hz, 6), 124.6 (5), 124.4 (3), 122.7 (d, *J* = 3.1 Hz, 4), 115.2 (d, *J* = 22.0 Hz, 11), 113.9 (d, *J* = 20.2 Hz, 1), 113.1 (d, *J* = 7.3 Hz, 10), 46.1 (7), 37.6 (15), 18.6 (16), 13.5 (17).

N-(4-{[(4-Fluorophenyl)amino]methyl}-2-(trifluoromethyl)phenyl)butyramide hydrochloride (**35d**)

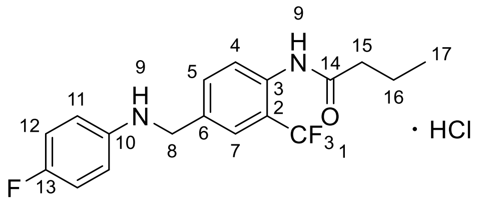

^{1}H-NMR (400 MHz, MeOH-d$_4$): δ(ppm) = 7.83 (d, *J* = 2.0 Hz, 1H, 7-H), 7.74 (dd, *J* = 8.3, 2.1 Hz, 1H, 5-H), 7.66 (d, *J* = 8.3 Hz, 1H, 4-H), 7.53 – 7.43 (m, 2H, 11-H), 7.37 – 7.27 (m, 2H, 12-H), 4.71 (s, 2H, 8-H), 2.44 (t, *J* = 7.4 Hz, 2H, 15-H), 1.83 – 1.69 (m, 2H, 16-H), 1.04 (t, *J* = 7.4 Hz, 3H, 17-H); **^{13}C-NMR (100 MHz, MeOH-d$_4$):** δ(ppm) = 175.9 (14), 164.2 (d, *J* = 250.2 Hz, 13), 137.8 (3), 136.0 (5), 132.8 (10), 131.8 (4), 131.2 (6), 129.9 (7), 127.4 (q, *J* = 30.3 Hz, 2), 126.4 (11), 124.8 (q, *J* = 271.3 Hz, 1), 118.4 (d, *J* = 23.7 Hz, 12), 55.7 (8), 39.2 (15), 20.3 (16), 14.1 (17).

N-(4-{[(2,4-Difluorophenyl)amino]methyl}-2-methylphenyl)nicotinamide (**35e**)

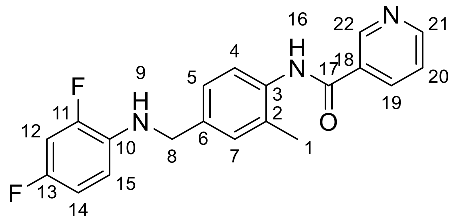

^{1}H-NMR (400 MHz, DMSO-d$_6$): δ(ppm) = 10.01 (s, 1H, 16-H), 9.15 – 9.09 (m, 1H, 22-H), 8.76 (dd, *J* = 4.8, 1.7 Hz, 1H, 21-H), 8.29 (dt, *J* = 8.1, 2.0 Hz, 1H, 19-H), 7.56 (ddd, *J* = 7.9, 4.8, 0.9 Hz, 1H, 20-H), 7.32 – 7.24 (m, 2H, 4-H, 7-H), 7.20 (dd, *J* = 8.1, 2.0 Hz, 1H, 5-H), 7.07 (ddd, *J* = 11.9, 8.9, 2.9 Hz, 1H, 12-H), 6.78 (tdd, *J* = 8.8, 2.9, 1.4 Hz, 1H, 14-H), 6.54 (ddd, *J* = 10.1, 9.0, 5.6 Hz, 1H, 15-H), 6.08 (td, *J* = 6.2, 2.0 Hz, 1H, 9-H), 4.30 (d, *J* = 6.1 Hz, 2H, 8-H), 2.22 (s, 3H, 1-H); **^{13}C-NMR (100 MHz, DMSO-d$_6$):** δ(ppm) = 163.9 (17), 152.1 (21), 152.8 (dd, *J* = 233.7, 11.0 Hz, 11), 150.0 (dd, *J* = 241.2, 11.9 Hz, 13), 148.6 (22), 137.7 (6), 135.4 (19), 134.5 (2), 133.6 (3), 133.4 (dd, *J* = 11.7, 2.6 Hz, 10), 130.1 (18), 128.9 (7), 126.5 (4), 124.6 (5), 123.5 (20), 112.1 (dd, *J* = 8.8, 5.2 Hz, 15), 110.6 (dd, *J* = 21.2, 3.5 Hz, 14), 103.4 (dd, *J* = 26.8, 22.7 Hz, 12), 45.9 (8), 18.0 (1).

N-(2-Methyl-4-{[(5-methylpyridin-2-yl)amino]methyl}phenyl)butyramide (**35f**)

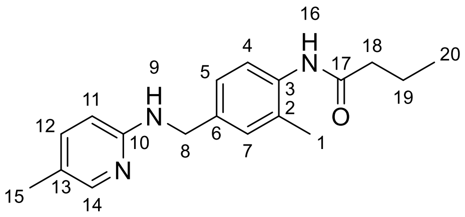

^{1}H-NMR (400 MHz, DMSO-d$_6$): δ(ppm) = 9.17 (s, 1H, 16-H), 7.80 – 7.74 (m, 1H, 14-H), 7.23 (d, *J* = 8.1 Hz, 1H, 4-H), 7.19 (dd, *J* = 8.5, 2.4 Hz, 1H, 12-H), 7.13 (d, *J* = 2.0 Hz, 1H, 7-H), 7.07 (dd, *J* = 8.1, 2.1 Hz, 1H, 5-H), 6.72 (t, *J* = 6.0 Hz, 1H, 9-H), 6.41 (d, *J* = 8.4 Hz, 1H, 11-H), 4.36 (d, *J* = 6.0 Hz, 2H, 8-H), 2.27 (t, *J* = 7.3 Hz, 2H, 18-H), 2.14 (s, 3H, 1-H), 2.07 (s, 3H, 15-H), 1.67 – 1.54 (m, 2H, 19-H), 0.92 (t, *J* = 7.4 Hz, 3H, 20-H); **^{13}C-NMR (100 MHz, DMSO-d$_6$):** δ(ppm) = 170.9 (17), 156.9 (10), 146.9 (14), 137.6 (12), 137.5 (3), 134.8 (2), 131.7 (7), 129.0 (6), 125.2 (4), 124.7 (5), 119.7 (13), 107.7 (11), 44.0 (8), 37.7 (18), 18.8 (19), 18.0 (1), 17.0 (15), 13.6 (20).

Ethyl 6-aminonicotinate (**37**)

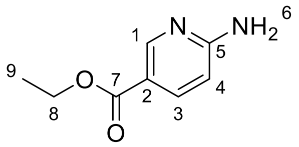

^{1}H-NMR (400 MHz, DMSO-d$_6$): δ(ppm) = 8.48 (dd, *J* = 2.4, 0.8 Hz, 1H, 1-H), 7.80 (dd, *J* = 8.7, 2.4 Hz, 1H, 3-H), 6.79 (s, 2H, 6-H), 6.43 (dd, *J* = 8.8, 0.8 Hz, 1H, 4-H), 4.21 (q, *J* = 7.1 Hz, 2H, 8-H), 1.26 (t, *J* = 7.1 Hz, 3H, 9-H); **^{13}C-NMR (100 MHz, DMSO-d$_6$):** δ(ppm) = 165.2 (7), 162.5 (5), 151.0 (1), 137.5 (3), 113.5 (2), 107.0 (4), 59.8 (8), 14.3 (9).

Ethyl 6-amino-5-bromonicotinate (**38**)

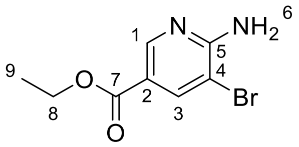

^{1}H-NMR (400 MHz, DMSO-d$_6$): δ(ppm) = 8.48 (d, *J* = 2.0 Hz, 1H, 1-H), 8.06 (d, *J* = 2.0 Hz, 1H, 3-H), 7.13 (s, 2H, 6-H), 4.23 (q, *J* = 7.1 Hz, 2H, 8-H), 1.27 (t, *J* = 7.1 Hz, 3H, 9-H); **^{13}C-NMR (100 MHz, DMSO-d$_6$):** δ(ppm) = 164.0 (7), 159.1 (5), 149.4 (1), 140.1 (3), 115.3 (2), 101.9 (4), 60.3 (8), 14.2 (9).

(6-Amino-5-bromopyridin-3-yl)methanol (**39**)

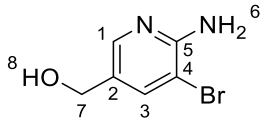

^{1}H-NMR (400 MHz, DMSO-d$_6$): δ(ppm) = 7.86 (d, *J* = 2.0 Hz, 1H, 1-H), 7.64 (d, *J* = 2.0 Hz, 1H, 3-H), 6.07 (s, 2H, 6-H), 5.02 (t, *J* = 5.7 Hz, 1H, 8-H), 4.29 (d, *J* = 5.7 Hz, 2H, 7-H); **^{13}C-NMR (100 MHz, DMSO-d$_6$):** δ(ppm) = 155.4 (4), 145.7 (1), 139.4 (3), 127.7 (2), 102.8 (5), 59.9 (7).

6-Amino-5-bromonicotinaldehyde (**40**)

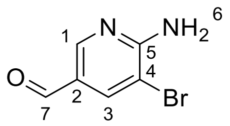

^{1}H-NMR (400 MHz, DMSO-d$_6$): δ(ppm) = 9.67 (s, 1H, 7-H), 8.47 (d, *J* = 1.9 Hz, 1H, 1-H), 8.06 (d, *J* = 1.9 Hz, 1H, 3-H), 7.40 (s, 2H, 6-H); **^{13}C-NMR (100 MHz, DMSO-d$_6$):** δ(ppm) = 188.5 (7), 159.7 (5), 152.6 (1), 138.8 (3), 123.5 (2), 103.2 (4).

N-(3-Bromo-5-formylpyridin-2-yl)butyramide (**41a**)

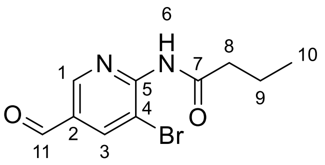

^{1}H-NMR (400 MHz, DMSO-d$_6$): δ(ppm) = 10.37 (s, 1H, 6-H), 10.00 (s, 1H, 11-H), 8.88 (d, *J* = 1.9 Hz, 1H, 1-H), 8.48 (d, *J* = 1.9 Hz, 1H, 3-H), 2.37 (t, *J* = 7.3 Hz, 2H, 8-H), 1.76 – 1.42 (m, 2H, 9-H), 0.93 (t, *J* = 7.4 Hz, 3H, 10-H); **^{13}C-NMR (100 MHz, DMSO-d$_6$):** δ(ppm) = 190.4 (11), 171.3 (6), 153.6 (2), 149.1 (1), 141.7 (3), 130.1 (4), 115.8 (5), 37.5 (8), 18.3 (9), 13.6 (10).

N-(3-Bromo-5-formylpyridin-2-yl)-3,3-dimethylbutanamide (**41b**)

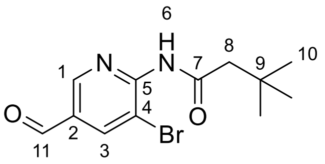

^{1}H-NMR (400 MHz, DMSO-d$_6$): δ(ppm) = 10.34 (s, 1H, 6-H), 10.01 (s, 1H, 11-H), 8.89 (d, *J* = 2.0 Hz, 1H, 1-H), 8.48 (d, *J* = 1.9 Hz, 1H, 3-H), 2.27 (s, 2H, 8-H), 1.04 (s, 9H, 10-H); **^{13}C-NMR (100 MHz, DMSO-d$_6$):** δ(ppm) = 190.4 (11), 169.9 (7), 153.6 (5), 149.0 (1), 141.8 (3), 130.2 (2), 116.0 (4), 48.6 (8), 31.0 (9), 29.7 (10).

N-(3-Bromo-5-{[(4-fluorophenyl)amino]methyl}pyridin-2-yl)butyramide (**42a**)

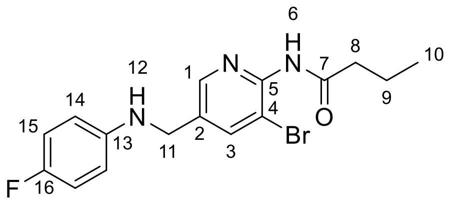

¹H-NMR (400 MHz, DMSO-d$_6$): δ(ppm) = 10.03 (s, 1H, 6-H), 8.40 (d, *J* = 2.0 Hz, 1H, 1-H), 8.05 (d, *J* = 2.0 Hz, 1H, 3-H), 6.96 – 6.85 (m, 2H, 15-H), 6.63 – 6.54 (m, 2H, 14-H), 6.22 (t, *J* = 6.3 Hz, 1H, 12-H), 4.28 (d, *J* = 6.2 Hz, 2H, 11-H), 2.28 (t, *J* = 7.3 Hz, 2H, 8-H), 1.65 – 1.54 (m, 2H, 9-H), 0.93 (t, *J* = 7.4 Hz, 3H, 10-H); **¹³C-NMR (100 MHz, DMSO-d$_6$):** δ(ppm) = 171.2 (7), 154.5 (d, *J* = 231.7 Hz, 16), 148.1 (5), 146.5 (1), 144.7 (d, *J* = 1.5 Hz, 13), 140.6 (3), 135.6 (2), 117.3 (4), 115.3 (d, *J* = 22.0 Hz, 15), 113.2 (d, *J* = 7.4 Hz, 14), 43.4 (11), 37.2 (8), 18.4 (9), 13.6 (10).

N-(3-Bromo-5-{[(4-fluorophenyl)amino]methyl}pyridin-2-yl)-3,3-dimethylbutanamide (**42b**)

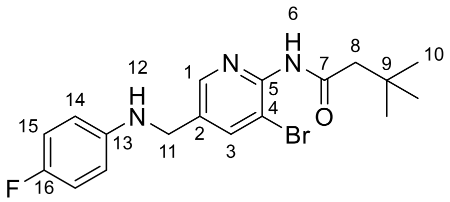

¹H-NMR (400 MHz, DMSO-d$_6$): δ(ppm) = 9.96 (s, 1H, 6-H), 8.38 (d, *J* = 2.1 Hz, 1H, 1-H), 8.03 (d, *J* = 2.0 Hz, 1H, 3-H), 6.94 – 6.83 (m, 2H, 15-H), 6.61 – 6.52 (m, 2H, 14-H), 6.20 (t, *J* = 6.3 Hz, 1H, 12-H), 4.26 (d, *J* = 6.2 Hz, 2H, 11-H), 2.17 (s, 2H, 8-H), 1.03 (s, 9H, 10-H); **¹³C-NMR (100 MHz, DMSO-d$_6$):** δ(ppm) = 169.8 (7), 154.5 (d, *J* = 231.4 Hz, 16), 148.2 (5), 146.5 (1), 144.7 (13), 140.6 (3), 135.6 (2), 117.2 (4), 115.3 (d, *J* = 22.0 Hz, 15), 113.2 (d, *J* = 7.3 Hz, 14), 48.5 (8), 43.3 (11), 30.8 (9), 29.7 (10).

N-(5-{[(4-Fluorophenyl)amino]methyl}-3-methylpyridin-2-yl)butyramide (**43a**)

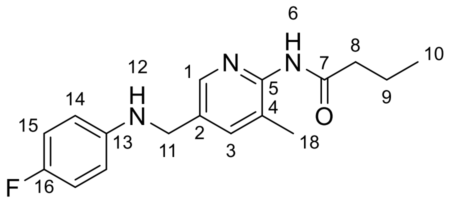

¹H-NMR (400 MHz, DMSO-d$_6$): δ(ppm) = 9.87 (s, 1H, 6-H), 8.21 (d, *J* = 2.2 Hz, 1H, 1-H), 7.60 (d, *J* = 2.3 Hz, 1H, 3-H), 6.97 – 6.80 (m, 2H, 15-H), 6.69 – 6.49 (m, 2H, 14-H), 6.13 (t, *J* = 6.0 Hz, 1H, 12-H), 4.21 (d, *J* = 6.0 Hz, 2H, 11-H), 2.29 (t, *J* = 7.3 Hz, 2H, 8-H), 2.12 (s, 3H, 18-H), 1.74 – 1.49 (m, 2H, 9-H), 0.92 (t, *J* = 7.4 Hz, 3H, 10-H); **¹³C-NMR (100 MHz, DMSO-d$_6$):** δ(ppm) = 171.2 (7), 154.4 (d, *J* = 231.1 Hz, 16),

149.1 (4), 145.1 (d, J = 1.6 Hz, 13), 144.7 (1), 138.3 (3), 133.2 (2), 128.3 (5), 115.2 (d, J = 22.0 Hz, 15), 113.1 (d, J = 7.3 Hz, 14), 44.1 (11), 37.3 (8), 18.5 (9), 17.7 (18), 13.6 (10).

N-(5-{[(4-Fluorophenyl)amino]methyl}-3-methylpyridin-2-yl)-3,3-dimethylbutanamide (**43b**)

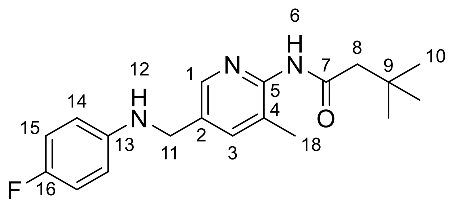

^{1}H-NMR (400 MHz, DMSO-d$_6$): δ(ppm) = 9.82 (s, 1H, 6-H), 8.21 (d, J = 2.2 Hz, 1H, 1-H), 7.61 (d, J = 2.3 Hz, 1H, 3-H), 6.95 – 6.84 (m, 2H, 14-H), 6.63 – 6.53 (m, 2H, 15-H), 6.14 (t, J = 6.0 Hz, 1H, 12-H), 4.22 (d, J = 6.0 Hz, 2H, 11-H), 2.21 (s, 2H, 8-H), 2.15 (s, 3H, 18-H), 1.04 (s, 9H, 10-H); **^{13}C-NMR (100 MHz, DMSO-d$_6$):** δ(ppm) = 169.9 (7), 154.4 (d, J = 231.3 Hz, 16-H), 149.1 (4), 145.1 (d, J = 1.6 Hz, 13-H), 144.8 (1), 138.3 (3), 133.2 (2), 128.4 (5), 115.2 (d, J = 21.9 Hz, 15-H), 113.1 (d, J = 7.3 Hz, 14-H), 48.6 (8), 44.1 (11), 30.7 (9), 29.7 (10), 18.0 (18).

N-(3-Cyclopropyl-5-{[(4-fluorophenyl)amino]methyl}pyridin-2-yl)butyramide (**43c**)

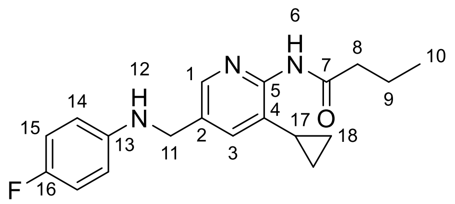

^{1}H-NMR (400 MHz, DMSO-d$_6$): δ(ppm) = 9.83 (s, 1H, 6-H), 8.17 (d, J = 2.2 Hz, 1H, 1-H), 7.34 (d, J = 2.3 Hz, 1H, 3-H), 6.95 – 6.84 (m, 2H, 15-H), 6.62 – 6.52 (m, 2H, 14-H), 6.09 (t, J = 6.2 Hz, 1H, 12-H), 4.19 (d, J = 6.1 Hz, 2H, 11-H), 2.30 (d, J = 7.3 Hz, 2H, 8-H), 1.95 – 1.84 (m, 1H, 17-H), 1.65 – 1.53 (m, 2H, 9-H), 0.92 (t, J = 7.4 Hz, 3H, 10-H), 0.89 – 0.84 (m, 2H, 18-H), 0.58 – 0.49 (m, 2H, 18-H); **^{13}C-NMR (100 MHz, DMSO-d$_6$):** δ(ppm) = 171.5 (7), 154.4 (d, J = 231.2 Hz, 16), 149.3 (5), 145.0 (d, J = 1.6 Hz, 13), 144.3 (1), 133.8 (2), 133.3 (4), 133.3 (3), 115.2 (d, J = 22.0 Hz, 15), 113.2 (d, J = 7.3 Hz, 14), 44.2 (11), 37.3 (8), 18.6 (9), 13.7 (10), 11.1 (17), 7.8 (18).

N-(5-{[(4-Fluorophenyl)amino]methyl}pyridin-2-yl)butyramide (**44**)

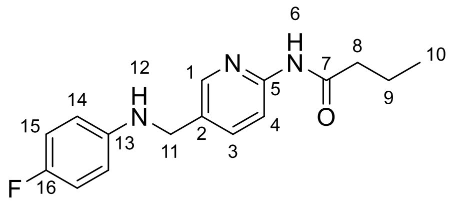

¹H-NMR (400 MHz, DMSO-d₆): δ(ppm) = 10.37 (s, 1H, 6-H), 8.28 (d, *J* = 2.5 Hz, 1H, 1-H), 8.04 (d, *J* = 8.5 Hz, 1H, 4-H), 7.72 (dd, *J* = 8.5, 2.4 Hz, 1H, 3-H), 6.95 – 6.84 (m, 2H, 15-H), 6.62 – 6.52 (m, 2H, 14-H), 6.10 (t, *J* = 6.1 Hz, 1H, 12-H), 4.19 (d, *J* = 6.0 Hz, 2H, 11-H), 2.34 (t, *J* = 7.3 Hz, 2H, 8-H), 1.66 – 1.54 (m, 2H, 9-H), 0.89 (t, *J* = 7.4 Hz, 3H, 10-H); **¹³C-NMR (100 MHz, DMSO-d₆):** δ(ppm) = 171.9 (7), 154.4 (d, *J* = 231.1 Hz, 16), 151.0 (5), 146.9 (1), 145.1 (d, *J* = 1.5 Hz, 13), 137.3 (3), 130.5 (2), 115.2 (d, *J* = 21.9 Hz, 15), 113.2 (d, *J* = 7.3 Hz, 14), 113.0 (4), 44.2 (11), 37.9 (8), 18.4 (9), 13.5 (10).

4-(1,3-Dioxoisoindolin-2-yl)-3-methylbenzonitrile (**45**)

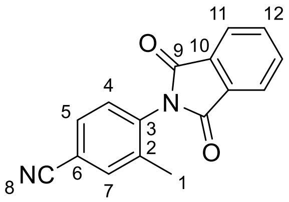

¹H-NMR (400 MHz, DMSO-d₆): δ(ppm) = 8.05 – 7.98 (m, 2H, 12-H), 7.98 – 7.89 (m, 3H, 7-H, 11-H), 7.85 (ddd, *J* = 8.1, 2.0, 0.7 Hz, 1H, 5-H), 7.63 (d, *J* = 8.1 Hz, 1H, 4-H), 2.21 (s, 3H, 1H); **¹³C-NMR (100 MHz, DMSO-d₆):** δ(ppm) = 166.4 (9), 138.3 (3), 135.5 (2), 134.9 (11), 134.5 (7), 131.7 10), 130.5 (5), 130.4 (4), 123.7 (12), 118.3 (6), 111.8 (8), 17.3 (1).

4-(1,3-Dioxoisoindolin-2-yl)-3-methylbenzaldehyde (**46**)

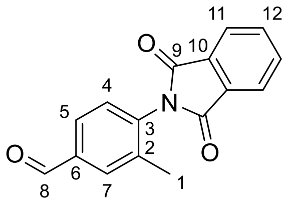

¹H-NMR (400 MHz, DMSO-d₆): δ(ppm) = 10.06 (s, 1H, 8-H), 8.03 – 7.99 (m, 2H, 11-H), 7.98 – 7.92 (m, 3H, 7-H, 12-H), 7.91 – 7.87 (m, 1H, 5-H), 7.63 (d, *J* = 8.0 Hz, 1H, 4-H), 2.25 (s, 3H, 1-H); **¹³C-NMR (100 MHz, DMSO-d₆):** δ(ppm) = 192.6 (8), 166.6 (9), 137.6 (3), 136.4 (6), 136.3 (2), 134.9 (12), 131.7 (7), 131.6 (10), 130.1 (4), 127.5 (5), 123.7 (11), 17.5 (1).

N-(4-{[(4-Fluorophenyl)amino]methyl}-2-methylphenyl)-2-(hydroxymethyl)benzamide (**48**)

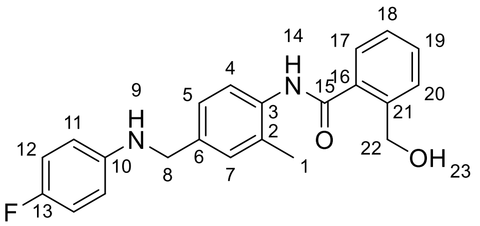

^{1}H-NMR (400 MHz, DMSO-d$_{6}$): δ(ppm) = 9.82 (s, 1H, 14-H), 7.63 – 7.55 (m, 2H, 17-H 19-H), 7.53 – 7.45 (m, 1H, 20-H), 7.42 – 7.33 (m, 2H, 4-H, 18-H), 7.24 (d, *J* = 2.0 Hz, 1H, 7-H), 7.18 (dd, *J* = 8.3, 2.1 Hz, 1H, 5-H), 6.94 – 6.83 (m, 2H, 12-H), 6.60 – 6.50 (m, 2H, 11-H), 6.14 (t, *J* = 6.0 Hz, 1H, 9-H), 5.36 (t, *J* = 5.5 Hz, 1H, 23-H), 4.69 (d, *J* = 5.5 Hz, 2H, 22-H), 4.19 (d, *J* = 5.9 Hz, 2H, 8-H), 2.24 (s, 3H, 1-H); **^{13}C-NMR (100 MHz, DMSO-d$_{6}$):** δ(ppm) = 167.3 (15), 154.3 (d, *J* = 230.9 Hz, 13), 145.3 (16), 140.2 (10), 137.5 (21), 135.2 (6), 134.8 (2), 132.7 (3), 129.9 (20), 129.1 (7), 127.6 (19), 127.6 (17), 126.7 (18), 125.9 (4), 124.8 (5), 115.2 (d, *J* = 22.0 Hz, 12), 113.0 (d, *J* = 7.3 Hz, 11), 61.0 (8), 46.7 (22), 18.1 (1).

2-(4-{[(4-Fluorophenyl)amino]methyl}-2-methylphenyl)isoindoline-1,3-dione (**49**)

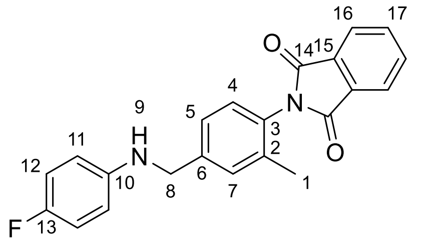

^{1}H-NMR (400 MHz, DMSO-d$_{6}$): δ(ppm) = 8.01 – 7.94 (m, 2H, 17-H), 7.94 – 7.87 (m, 2H, 16-H), 7.40 – 7.34 (m, 1H, 7-H), 7.33 – 7.23 (m, 2H, 4-H, 5-H), 6.94 – 6.85 (m, 2H, 12-H), 6.61 – 6.53 (m, 2H, 11-H), 6.21 (t, *J* = 6.0 Hz, 1H, 9-H), 4.25 (d, *J* = 6.0 Hz, 2H, 8-H), 2.09 (s, 3H, 1-H); **^{13}C-NMR (100 MHz, DMSO-d$_{6}$):** δ(ppm) = 167.1 (14), 154.3 (d, *J* = 231.0 Hz, 13), 145.3 (d, *J* = 1.3 Hz, 10), 141.3 (6), 136.1 (3), 134.7 (16), 131.6 (15), 129.4 (2), 129.3 (1), 129.1 (4), 125.4 (5), 123.5 (17), 115.2 (d, *J* = 22.0 Hz, 12), 112.9 (d, *J* = 7.3 Hz, 11), 46.6 (8), 17.5 (1).

Benzyl [4-(1,3-dioxoisoindolin-2-yl)-3-methylbenzyl](4-fluorophenyl)carbamate (**50**)

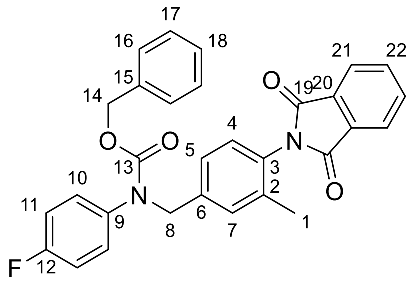

^{1}H-NMR (400 MHz, DMSO-d$_6$): δ(ppm) = 8.01 – 7.95 (m, 2H, 22-H), 7.95 – 7.89 (m, 2H, 21-H), 7.42 – 7.14 (m, 12H, 4-H, 5-H, 7-H, 10-H, 11-H, 16-H, 17-H, 18-H), 5.16 (s, 2H, 8-H), 4.92 (s, 2H, 14-H), 2.07 (s, 3H, 1-H); **^{13}C-NMR (100 MHz, DMSO-d$_6$):** δ(ppm) = 167.0 (19), 160.1 (d, *J* = 243.7 Hz, 12), 154.9 (13), 138.7 (2), 138.0 (9), 136.5 (15), 136.3 (6), 134.7 (21), 131.6 (20), 129.8 (3), 129.3 (4), 128.7 (7), 128.6 (d, *J* = 8.9 Hz, 12), 128.4 (17), 127.8 (18), 127.4 (16), 125.3 (5), 123.5 (22), 115.6 (d, *J* = 22.5 Hz, 11), 66.9 (8), 53.1 (14), 17.47 (1).

Benzyl (4-amino-3-methylbenzyl)(4-fluorophenyl)carbamate (**51**)

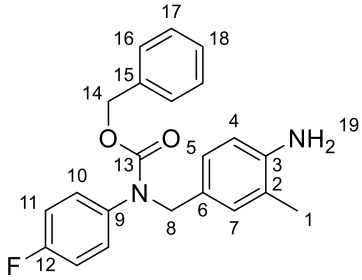

^{1}H-NMR (400 MHz, DMSO-d$_6$): δ(ppm) = 7.39 – 7.24 (m, 5H, 16-H, 17-H, 18-H), 7.19 – 7.07 (m, 4H, 10-H, 11-H), 6.73 (d, *J* = 2.4 Hz, 1H, 7-H), 6.66 (dd, *J* = 8.1, 2.1 Hz, 1H, 5-H), 6.48 (d, *J* = 8.0 Hz, 1H, 4-H), 5.12 (s, 2H, 8-H), 4.75 (s, 2H, 19-H), 4.64 (s, 2H, 14-H), 1.96 (s, 3H, 1-H); **^{13}C-NMR (100 MHz, DMSO-d$_6$):** δ(ppm) = 160.1 (*J* = 243.2 Hz, 12), 154.8 (13), 145.8 (2), 137.9 (15), 136.7 (9), 129.6 (7), 129.2 (d, *J* = 8.4 Hz, 10), 128.3 (17), 127.8 (18), 127.4 (16), 126.1 (5), 124.6 (6), 120.9 (3), 115.4 (d, *J* = 22.5 Hz, 11), 113.8 (4), 66.6 (14), 53.2 (8), 17.4 (1).

Benzyl {4-[3-(1*H*-pyrazol-1-yl)propanamido]-3-methylbenzyl}(4-fluorophenyl)carbamate (**52**)

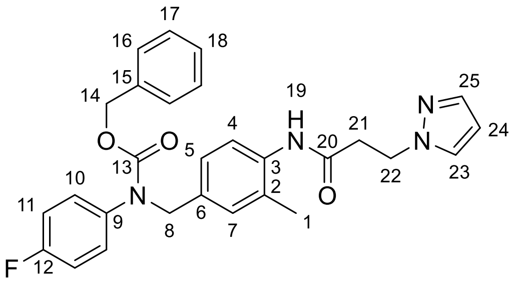

^{1}H-NMR (400 MHz, DMSO-d$_6$): δ(ppm) = 9.29 (s, 1H, 19-H), 7.67 (d, *J* = 2.3 Hz, 1H, 23-H), 7.44 (d, *J* = 2.0 Hz, 1H, 25-H), 7.38 – 7.20 (m, 8H, 4-H, 10-H, 16-H, 17-H, 18-H), 7.19 – 7.09 (m, 2H, 11-H), 6.99 (d, *J* = 2.1 Hz, 1H, 7-H), 6.95 (dd, *J* = 8.1, 2.1 Hz, 1H, 5-H), 6.21 (t, *J* = 2.0 Hz, 1H, 24-H), 5.13 (s, 2H, 8-H), 4.79 (s, 2H, 14-H), 4.40 (t, *J* = 6.8 Hz, 2H, 22-H), 2.88 (t, *J* = 6.8 Hz, 2H, 21-H), 2.04 (s, 3H, 1-H); **^{13}C-NMR (100 MHz, DMSO-d$_6$):** δ(ppm) = 168.5 (20), 160.1 (d, *J* = 242.8 Hz, 12), 154.9 (13), 138.6 (25), 137.8 (9), 136.6 (6), 135.2 (3), 134.3 (2), 131.7 (15), 129.9 (23), 129.3 (7), 128.9 (10), 128.3 (17), 127.8 (18), 127.4 (16), 125.0 (5), 124.9 (4), 115.5 (d, *J* = 22.6 Hz, 11), 104.9 (24), 66.8 (8), 53.0 (14), 47.4 (22), 36.4 (21), 17.8 (1).

N-(4-{[(4-Fluorophenyl)amino]methyl}-2-methylphenyl)-3-(1*H*-pyrazol-1-yl)propanamide (**53**)

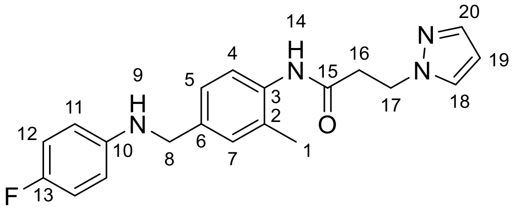

^{1}H-NMR (400 MHz, DMSO-d$_6$): δ(ppm) = 9.30 (s, 1H, 14-H), 7.67 (d, *J* = 2.2 Hz, 1H, 18-H), 7.44 (d, *J* = 1.8 Hz, 1H, 20-H), 7.24 (d, *J* = 8.1 Hz, 1H, 4-H), 7.16 (d, *J* = 2.1 Hz, 1H, 7-H), 7.11 (dd, *J* = 8.1, 2.1 Hz, 1H, 5-H), 6.92 – 6.81 (m, 2H, 12-H), 6.58 – 6.48 (m, 2H, 11-H), 6.21 (t, *J* = 2.0 Hz, 1H, 19-H), 6.09 (t, *J* = 6.0 Hz, 1H, 9-H), 4.40 (t, *J* = 6.8 Hz, 2H, 17-H), 4.14 (d, *J* = 5.9 Hz, 2H, 8-H), 2.87 (t, *J* = 6.8 Hz, 2H, 16-H), 2.07 (s, 3H, 1-H); **^{13}C-NMR (100 MHz, DMSO-d$_6$):** δ(ppm) = 168.5 (15), 154.2 (d, *J* = 230.7 Hz, 13), 145.4 (10), 138.6 (20), 136.9 (6), 134.7 (3), 131.8 (2), 129.9 (18), 129.0 (7), 125.1 (4), 124.7 (5), 115.2 (d, *J* = 22.0 Hz, 12), 112.9 (d, *J* = 7.3 Hz, 11), 104.9 (19), 47.5 (17), 46.6 (8), 36.4 (16), 17.8 (1).

N-(2,4-Dimethylphenyl)-3-(1*H*-pyrazol-1-yl)propanamide (**54**)

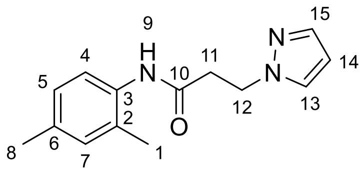

^{1}H-NMR (400 MHz, DMSO-d$_6$): δ(ppm) = 9.27 (s, 1H, 9-H), 7.67 (d, *J* = 1.6 Hz, 1H, 13-H), 7.44 (d, *J* = 1.1 Hz, 1H, 15-H), 7.17 (d, *J* = 8.0 Hz, 1H, 4-H), 6.98 (d, *J* = 2.0 Hz, 1H, 7-H), 6.93 (dd, *J* = 8.0, 2.1 Hz, 1H, 5-H), 6.21 (t, *J* = 2.0 Hz, 1H, 14-H), 4.40 (t, *J* = 6.8 Hz, 2H, 12-H), 2.87 (t, *J* = 6.8 Hz, 2H, 11-H), 2.23 (s, 3H, 8-H), 2.05 (s, 3H, 1-H); **^{13}C-NMR (100 MHz, DMSO-d$_6$):** δ(ppm) = 168.5 (10), 138.6 (15), 134.2 (2), 133.5 (3), 131.8 (6), 130.7 (7), 129.9 (13), 126.3 (5), 125.2 (4), 104.9 (14), 47.5 (12), 36.4 (11), 20.4 (8), 17.7 (1).

Methyl 3-(1*H*-pyrazol-1-yl)propanoate (**57**)

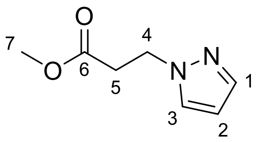

^{1}H-NMR (400 MHz, DMSO-d$_6$): δ(ppm) = 7.69 (dd, *J* = 2.3, 0.8 Hz, 1H, 3-H), 7.42 (dd, *J* = 1.9, 0.7 Hz, 1H, 1-H), 6.20 (t, *J* = 2.1 Hz, 1H, 2-H), 4.35 (t, *J* = 6.7 Hz, 2H, 4-H), 3.59 (s, 3H, 7-H), 2.86 (t, *J* = 6.7 Hz, 2H, 5-H); **^{13}C-NMR (100 MHz, DMSO-d$_6$):** δ(ppm) = 171.1 (6), 138.7 (1), 130.0 (3), 105.0 (2), 51.5 (7), 46.7 (4), 34.2 (5).

3-(1*H*-Pyrazol-1-yl)propanoic acid (**58**)

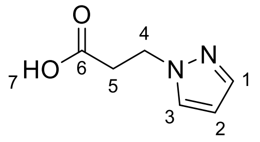

^{1}H-NMR (400 MHz, DMSO-d$_6$): δ(ppm) = 11.14 (s, 1H, 7-H), 7.69 (dd, *J* = 2.3, 0.7 Hz, 1H, 3-H), 7.43 (dd, *J* = 1.8, 0.7 Hz, 1H, 1-H), 6.21 (t, *J* = 2.1 Hz, 1H, 2-H), 4.31 (t, *J* = 6.8 Hz, 2H, 4-H), 2.77 (t, *J* = 6.8 Hz, 2H, 5-H); **^{13}C-NMR (100 MHz, DMSO-d$_6$):** δ(ppm) = 172.2 (6), 138.6 (1), 130.1 (3), 105.0 (2), 46.9 (4), 34.5 (5).

Benzyl (4-acrylamido-3-methylbenzyl)(4-fluorophenyl)carbamate (**59**)

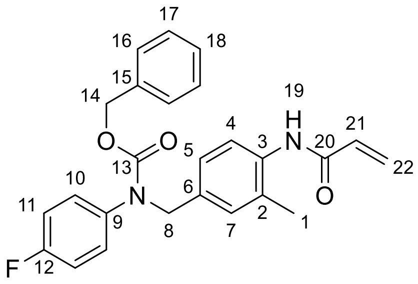

^{1}H-NMR (400 MHz, DMSO-d$_6$): δ(ppm) = 9.42 (s, 1H, 19-H), 7.41 (d, *J* = 8.1 Hz, 1H, 4-H), 7.38 – 7.20 (m, 7H, 10-H, 16-H, 17-H, 18-H), 7.20 – 7.10 (m, 2H, 11-H), 7.04 (d, *J* = 2.1 Hz, 1H, 7-H), 6.99 (dd, *J* = 8.1, 2.1 Hz, 1H, 5-H), 6.52 (dd, *J* = 17.0, 10.2 Hz, 1H, 21-H), 6.23 (dd, *J* = 17.1, 2.0 Hz, 1H, 22-H), 5.73 (dd, *J* = 10.2, 2.1 Hz, 1H, 22-H), 5.14 (s, 2H, 8-H), 4.81 (s, 2H, 14-H), 2.14 (s, 3H, 1-H); **^{13}C-NMR (100 MHz, DMSO-d$_6$):** δ(ppm) = 163.2 (20), 160.1 (d, *J* = 243.5 Hz, 12), 154.9 (13), 137.9 (6), 136.6 (15), 135.1 (9), 134.4 (2), 131.7 (21), 131.5 (3), 129.4 (7), 128.9 (10), 128.4 (17), 127.8 (18), 127.4 (16), 126.5 (22), 125.1 (5), 124.8 (4), 115.5 (d, *J* = 22.6 Hz, 11), 66.8 (8), 53.0 (14), 17.9 (1).

Benzyl (4-fluorophenyl){3-methyl-4-[3-(pyrrolidin-1-yl)propanamido]benzyl}carbamate (**60**)

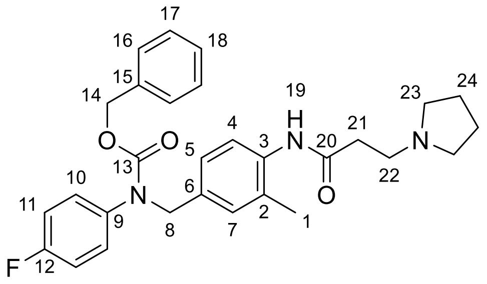

^{1}H-NMR (400 MHz, DMSO-d$_6$): δ(ppm) = 9.98 (s, 1H, 19-H), 7.64 (d, *J* = 8.2 Hz, 1H, 4-H), 7.39 – 7.18 (m, 7H, 10-H, 16-H, 17-H, 18-H), 7.18 – 7.09 (m, 2H, 11-H), 7.00 (d, *J* = 2.1 Hz, 1H, 7-H), 6.95 (dd, *J* = 8.2, 2.1 Hz, 1H, 5-H), 5.14 (s, 2H, 8-H), 4.78 (s, 2H, 14-H), 2.72 (t, *J* = 6.5 Hz, 2H, 22-H), 2.61 – 2.41 (m, 6H, 21-H, 24-H), 2.12 (s, 3H, 1-H), 1.79 – 1.65 (m, 4H, 23-H); **^{13}C-NMR (100 MHz, DMSO-d$_6$):** δ(ppm) = 170.2 (20), 160.1 (d, *J* = 243.4 Hz, 12), 154.9 (13), 137.8 (9), 136.6 (6), 135.9 (15), 133.1 (3), 129.3 (7), 129.2 (2), 129.0 (10), 128.4 (17), 127.8 (18), 127.4 (16), 125.2 (5), 122.8 (4), 115.5 (d, *J* = 22.6 Hz, 11), 66.8 (8), 53.2 (23), 52.9 (14), 51.5 (22), 35.2 (21), 23.1 (24), 17.6 (1).

N-(4-{[(4-Fluorophenyl)amino]methyl}-2-methylphenyl)-3-(pyrrolidin-1-yl)propanamide 2,2,2-trifluoroacetate (**61**)

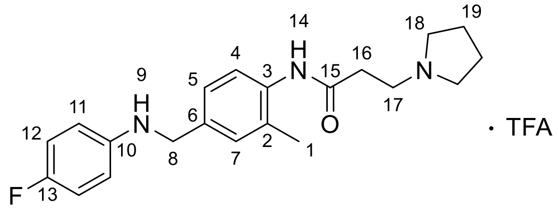

^{1}H-NMR (400 MHz, MeOH-d$_4$): δ(ppm) = 7.55 (d, *J* =8.2 Hz, 1H, 4-H), 7.20–7.35 (m, 7H, 5-H, 7-H 11-H, 12-H), 4.52 (s, 2H, 8-H), 3.78 – 3.68 (m, 2H, 18-H), 3.58 (t, *J* =6.8 Hz, 2H, 17-H), 3.24 – 3.11 (m, 2H, 18-H), 3.30 (t, *J* =6.8 Hz, 2H, 16-H), 2.31 (s, 3H, 1-H), 2.23 – 2.14 (m, 2H, 19-H), 2.14 – 2.03 (m, 2H, 19-H).

3 Log$D_{7.4}$ estimation

Table S1: Values used for Calibration A.

Reference	t_R mean [min]	k′	logk′	log*D* (Lit.)
Uracil	1.803			
Acetophenone	2.907	0.612	-0.213	1.7
Benzene	3.399	0.885	-0.053	2.1
Ethyl benzoate	4.077	1.261	0.101	2.6
Benzophenone	5.279	1.928	0.285	3.2
Phenyl benzoate	6.126	2.398	0.380	3.6
Diphenyl ether	7.818	3.336	0.523	4.2
Bibenzyl	12.013	5.663	0.753	4.8

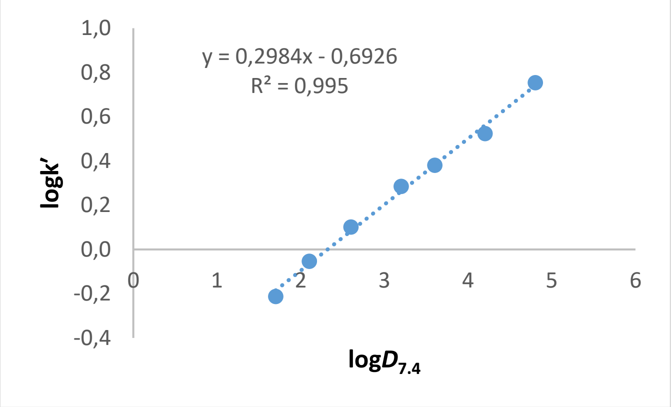

Figure S124: Calibration function A.

Table S2: Values used for Calibration B.

Reference	t_R mean [min]	k'	logk'	log*D* (Lit.)
Uracil	1.7965			
Acetophenone	2.857	0.585	-0.233	1.7
Benzene	3.323	0.843	-0.074	2.1
Ethyl benzoate	3.955	1.194	0.077	2.6
Benzophenone	5.0765	1.816	0.259	3.2
Phenyl benzoate	5.8465	2.243	0.351	3.6
Diphenyl ether	7.3915	3.100	0.491	4.2
Bibenzyl	11.2105	5.218	0.717	4.8

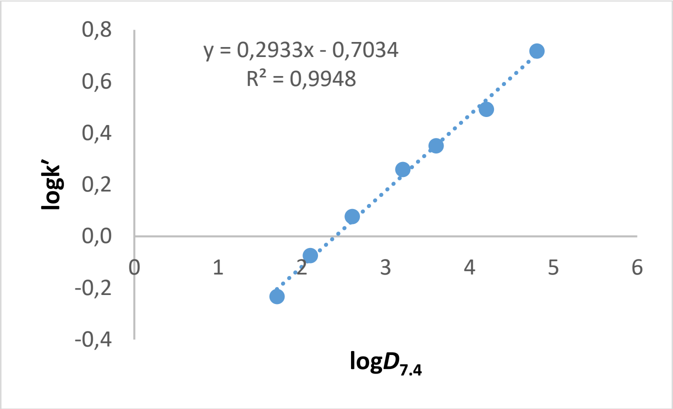

Figure S125: Calibration function B.

Table S3: Values used for Calibration C.

Reference	t_R mean [min]	k'	logk'	log*D* (Lit.)
Uracil	1.850			
Acetophenone	2.968	0.604	-0.219	1.7
Benzene	3.471	0.876	-0.057	2.1
Ethyl benzoate	4.156	1.246	0.096	2.6
Benzophenone	5.377	1.906	0.280	3.2
Phenyl benzoate	6.226	2.365	0.374	3.6
Diphenyl ether	7.938	3.291	0.517	4.2
Bibenzyl	12.214	5.602	0.748	4.8

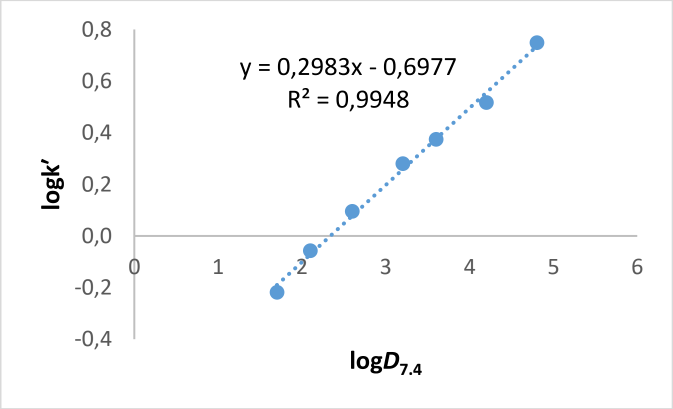

Figure S126: Calibration function C.

Table S4: Values used for Calibration D.

Reference	t_R mean [min]	k′	logk′	log*D* (Lit.)
Uracil	1.885			
Acetophenone	2.965	0.603	-0.220	1.7
Benzene	3.471	0.876	-0.057	2.1
Ethyl benzoate	4.155	1.246	0.095	2.6
Benzophenone	5.371	1.903	0.279	3.2
Phenyl benzoate	6.219	2.362	0.373	3.6
Diphenyl ether	7.928	3.285	0.517	4.2
Bibenzyl	12.186	5.587	0.747	4.8

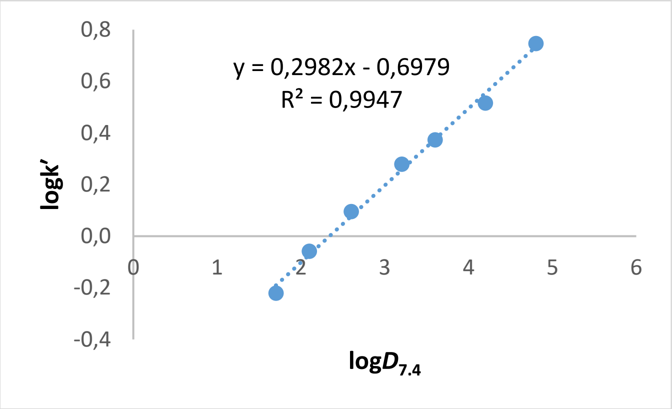

Figure S127: Calibration function D.

Table S5: Values used for Calibration E.

Reference	t_R mean [min]	k′	logk′	log*D* (Lit.)
Uracil	1.785			
Acetophenone	2.901	0.626	-0.204	1.7
Benzene	3.428	0.921	-0.036	2.1
Ethyl benzoate	4.054	1.272	0.104	2.6
Benzophenone	5.305	1.973	0.295	3.2
Phenyl benzoate	6.224	2.488	0.396	3.6
Diphenyl ether	7.924	3.440	0.537	4.2
Bibenzyl	12.286	5.885	0.770	4.8

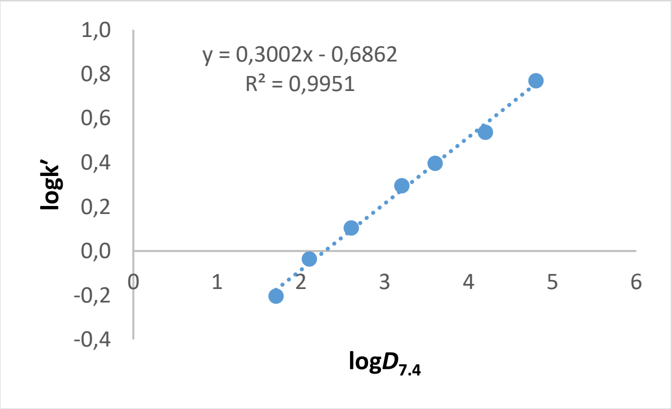

Figure S128: Calibration function E.

Table S6: Values used for Calibration F.

Reference	t_R mean [min]	k′	logk′	log*D* (Lit.)
Uracil	1.784			
Acetophenone	2.912	0.632	-0.199	1.7
Benzene	3.446	0.931	-0.031	2.1
Ethyl benzoate	4.086	1.290	0.110	2.6
Benzophenone	5.363	2.005	0.302	3.2
Phenyl benzoate	6.304	2.532	0.404	3.6
Diphenyl ether	8.043	3.507	0.545	4.2
Bibenzyl	12.521	6.016	0.779	4.8

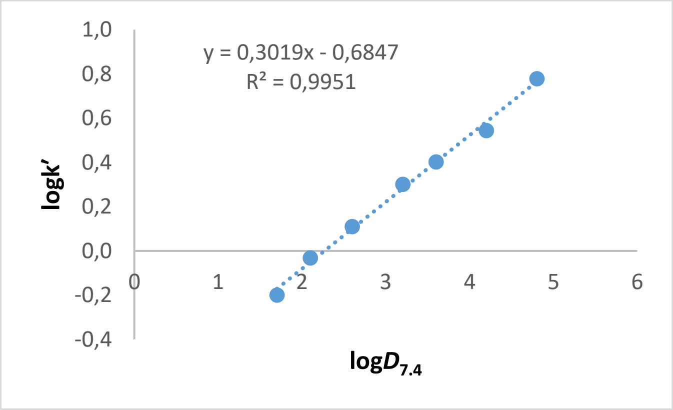

Figure S129: Calibration function F.

Log$D_{7.4}$ values

Table S7: Measured retention times and calculated log$D_{7.4}$ values.

Cmpd.	t_R [min]	k'	logk'	cali	log$D_{7.4}$	log$D_{7.4}$ (mean)
**Flupirtine**	3.351	0.859	-0.066	A	2.099	2.1
	3.242	0.798	-0.098	B	2.064	
**Retigabine**	3.369	0.821	-0.086	C	2.052	2.1
	3.372	0.823	-0.085	D	2.056	
**7**	4.581	1.541	0.188	A	2.950	3.0
	4.479	1.484	0.171	B	2.983	
**21**	4.661	1.585	0.200	A	2.992	3.0
	4.480	1.485	0.172	B	2.983	
**27**	5.638	2.127	0.328	A	3.419	3.4
	5.369	1.978	0.296	B	3.408	
**35a**	4.754	1.637	0.214	A	3.038	3.0
	4.552	1.525	0.183	B	3.023	
**35b**	4.444	1.465	0.166	A	2.877	2.9
	4.282	1.375	0.138	B	2.870	
**35c**	4.639	1.600	0.204	E	2.965	3.0
	4.712	1.641	0.215	F	2.980	
**35d**	4.687	1.600	0.204	A	3.005	3.0
	4.494	1.493	0.174	B	2.991	
**35e**	4.616	1.560	0.193	A	2.968	3.0
	4.423	1.453	0.162	B	2.952	
**35f**	3.566	0.978	-0.010	A	2.288	2.3
	3.449	0.913	-0.040	B	2.263	
**42a**	4.171	1.337	0.126	E	2.706	2.7
	4.217	1.363	0.135	F	2.714	
**43a**	3.628	1.033	0.014	E	2.333	2.3
	3.662	1.052	0.022	F	2.341	
**43b**	4.774	1.675	0.224	E	3.032	3.0
	4.835	1.709	0.233	F	3.039	
**43c**	4.272	1.394	0.144	E	2.766	2.8
	4.318	1.420	0.152	F	2.772	
**48**	4.546	1.521	0.182	A	2.932	2.9
	4.332	1.403	0.147	B	2.899	
**53**	4.577	1.565	0.194	E	2.934	2.9
	4.601	1.578	0.198	F	2.924	
